# The Effects of the Medium’s pH Value on the Efficiency of the Enzymatic Transglycosylation Catalyzed by *Escherichia coli* Purine Nucleoside Phosphorylase and *Lactobacillus leichmanii* Nucleoside Deoxyribosyltransferase Type II

**DOI:** 10.3390/ijms27219901

**Published:** 2026-11-05

**Authors:** Cyril S. Alexeev, Maria A. Konkina, Ivan A. Oskolsky, Dmitrii A. Platov, Mikhail S. Drenichev

**Affiliations:** 1 Laboratory of the Design and Synthesis of Biologically Active Compounds, Engelhardt Institute of Molecular Biology, Russian Academy of Sciences, Vavilova Str. 32, 119991 Moscow, Russiamdrenichev@mail.ru (M.S.D.); 2 Laboratory of Molecular Basis of Action of Physiologically Active Compounds, Engelhardt Institute of Molecular Biology, Russian Academy of Sciences, Vavilova Str. 32, 119991 Moscow, Russia

**Keywords:** biocatalysts, biochemistry, enzymatic transglycosylation, deoxyribosyltransferase, modified nucleosides, *Lactobacillus leichmanii* nucleoside deoxyribosyltransferase type II, *Escherichia coli* purine nucleoside phosphorylase

## Abstract

The effect of the reaction medium pH value on the rate and yield of enzymatic transglycosylation catalyzed by *Escherichia coli* purine nucleoside phosphorylase (*Ec*PNP) and *Lactobacillus leichmanii* type II nucleoside deoxyribosyltransferase (*Ll*NDTII) was investigated. In the presence of *Ec*PNP at pH 5.5, a significant reduction in the inosine formation rate was observed, compared with that of adenosine. A substituent at the purine *O*^6^ position neutralizes the negative effect of the 6-oxo group on the reaction rate. A substituent at the purine *N*^6^ position has virtually no effect on the transglycosylation rate. In reactions catalyzed by *Ll*NDTII, the rate of nucleoside formation from 4-oxopyrimidines was higher at pH 5.5, but the yield decreased by approximately 10% compared with that at pH 7.0; an unusual dependence of the yield of the target nucleoside on the enzyme concentration used was also revealed.

## 1. Introduction

Nucleosides are an important class of natural compounds that serve as the basis for the development of new drugs possessing a broad spectrum of biological activity. Currently, effective antiviral and antitumour drugs can have a modified nucleoside or similar fragment in their structure, which acts by mimicking the natural components of nucleic acids in the cell, thereby becoming included in cellular metabolism. The spectrum of biological activity of nucleoside analogues is diverse and includes antiviral, antibacterial, antifungal, and antitumour activity [1,2,3,4]. Nucleosides hold great potential for combating drug-resistant microorganisms [5]. Technologies for the production of practically significant modified nucleosides based on enzymatic transglycosylation reactions are currently being widely developed. Biocatalytic reactions, providing regio- and stereoselectivity as well as tunability, can complement, enhance or partially replace some steps of chemical methods of synthesis. Combining individual enzymatic reactions into multistep enzyme-catalyzed cascades brings additional advantages. Cascade enzymatic reactions are also eco-friendly, eliminating the time- and reagent-costly steps of isolation and purification of intermediates, as well as the functional groups’ protection/deprotection steps. Numerous enzymes such as glycosyltransferases, phosphoribosyltransferases, kinases, and reductases can participate in the cascade enzymatic synthesis of modified biologically active nucleosides [6,7,8]. However, enzymatic transglycosylation (consisting of two coupled phosphorolysis reactions) remains one of the primary enzymatic reactions for producing biologically active nucleosides. This reaction is typically catalyzed by enzymes of the glycosyltransferase class (EC 2.4) from various natural sources. Nucleoside phosphorylases (NPs, EC 2.4.2.x) and nucleoside-2′-deoxyribosyltransferases (NDTs, EC 2.4.2.6), which play an important role in nucleoside metabolism are primarily used as biocatalysts. NPs participate in the secondary pathway of nucleoside synthesis in the cell and catalyze the reversible phosphorolysis of (2′-deoxy)ribonucleosides, releasing the corresponding nucleobase and (2-deoxy)ribose-1-phosphate. NDTs participate in the recycling of nucleobases and 2′-deoxyribonucleosides in the DNA synthesis pathway in microorganisms, directly transferring a 2-deoxyribose residue between purine and/or pyrimidine bases.

**Figure 1:**
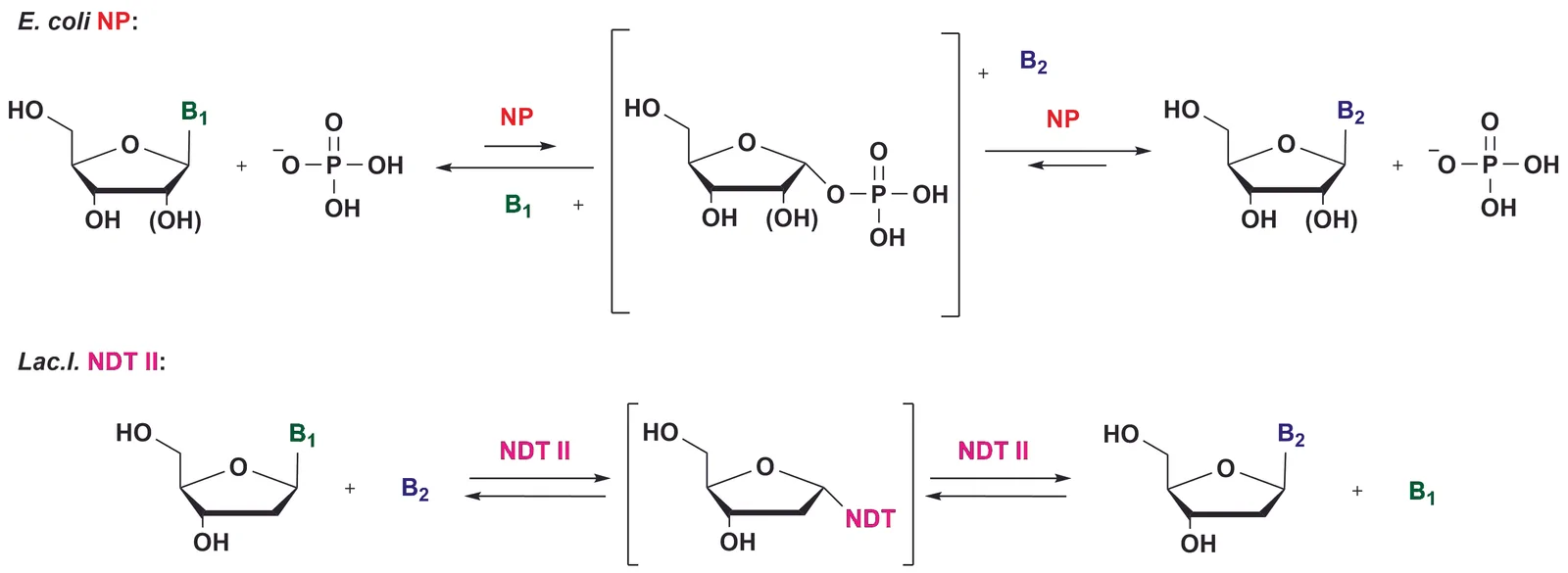
Enzymatic transglycosylation reactions catalyzed by NP and NDT II. B1 and B2 are heterocyclic bases.

Enzymatic transglycosylation is the reaction of the N-glycoside bond of the reversible cleavage in ribonucleosides/2′-deoxyribonucleosides forming the corresponding heterocyclic base and pentafuranose phosphate- or pentafuranose-enzyme complex derivative followed by the transfer of the latter to another heterocyclic base (Figure 1). The nucleoside phosphorylases (NPs) that are the most commonly used in the biotechnological industry are purine nucleoside phosphorylase (PNP, EC 2.4.2.1), pyrimidine-nucleoside phosphorylase (PyNP, EC 2.4.2.2), uridine phosphorylase (UP, EC 2.4.2.3), and thymidine phosphorylase (TP, EC 2.4.2.4). They require the presence of a phosphate anion, which is needed to form the intermediate pentafuranose-1-phosphate releasing the heterocyclic base from the initial nucleoside (Figure 1). Nucleoside phosphorylases are ubiquitous and found in almost all organisms.

Other pentosyltransferases used in the nucleoside derivatives synthesis comprised nucleoside deoxyribosyltransferases (NDTs), enzymes catalyzing the transfer of a 2-deoxyribose moiety from one purine/or pyrimidine nucleoside to another pyrimidine/purine base [8,9] forming pentafuranose-enzyme intermediate complex. Unlike nucleoside phosphorylases, these enzymes seem to be limited in their distribution to the bacterial genus *Lactobacillus* [10,11] and also found in parasitic unicellular organisms [12]. NDTs comprise two types of enzymes, according to their substrate specificity: enzymes belonging to type I are specific for purine nucleosides and heterocyclic bases, and type II enzymes utilize and catalyze reactions of both purine and pyrimidine nucleosides and bases [8,13]. The catalytic use of both types is limited to the synthesis of 2′-deoxyribonucleosides. The use of both types of enzymes—NPs and NDTs—has certain advantages and disadvantages. When NPs are used as catalysts, the reaction outcome is contingent upon the equilibrium shift toward nucleoside-donor phosphorolysis. Additionally, a pair of NPs (purine and pyrimidine) must be used if purine and pyrimidine nucleobases/nucleosides are present in the reaction medium. The inability to obtain cytosine/cytidine derivatives is a limitation. Finally, the presence of a labile intermediate α-*D*-(deoxy)ribose-1-phosphate in the reaction medium is a potential issue that could negatively affect the yield of target nucleosides. The advantages of NPs are as follows: (a) their widespread availability and accessibility, (b) their specificity for both deoxy- and ribo-derivatives of the carbohydrate residue, and (c) their sufficiently broad specificity to heterocyclic bases. The direct transfer of the deoxyribose residue to the acceptor base, the possibility of obtaining 2′-deoxycytidine/cytosine derivatives (such as cytarabine having an arabinose residue as the carbohydrate moiety), and the ability to use a single enzyme for purine and pyrimidine bases/nucleosides are benefits of NDTs. Disadvantages include the scarcity of sources for obtaining NDT enzymes and the specificity to 2′-deoxynucleosides only. The use of NDTs and NPs in biotechnological production could significantly increase the number of biologically active nucleosides and pharmaceutical substances that can be synthesized via enzymatic glycosylation. Despite the aforementioned deficiencies of both types of enzymes, some of them have been successfully resolved or are on the brink of being resolved. Thus, to shift the equilibrium when using NPs, a nucleoside donor undergoing irreversible phosphorolysis can be used [14,15,16]. The use of the minimum concentration of the second substrate, the phosphate anion, also has a significant effect on shifting the equilibrium towards the heterocyclic base and α-*D*-(deoxy)ribose-1-phosphate. This was first observed by Krenitsky [17] and was later confirmed by a proposed mathematical model [18] of enzymatic transglycosylation describing this effect [19,20]. The problem of the low prevalence of NDTs can be solved using genetic engineering methods to obtain highly purified recombinant enzymes in large quantities [8,9,21,22].

Currently, there is a lot of information concerning the influence of pH on enzyme activity. However, we have not been able to find any studies aimed at determining the dependence of the enzymatic transglycosylation yield (rate, outcome, efficiency) on the reaction medium pH value and the structure of the base acceptor, namely the configuration of the 6th position of the purine cycle (6-oxo- and 6-amino) for purines and the 4th position (4-oxo- and 4-amino-) for pyrimidines. We believe that the results of our study may be useful and applicable in the pharmacological and biotechnological industry.

## 2. Results and Discussion

To date, there is little or no literature data describing the effect of pH on the enzymatic activity of *Ec*PNP and *Ll*NDTII in the enzymatic transglycosylation process. Thus, there are some literature sources describing the effect of pH only on PNP activity, *i.g.* mammalian PNP from chicken liver [23,24], Chinese hamster [25], and bovine brain [26]. These works are mainly devoted to characterizing the enzyme—determining the pH optimum (its physicochemical properties)—with only a few of them studying catalytic activity as a function of pH. We could not find any studies examining the impact of pH on the yield and course of enzymatic transglycosylation depending on the structure of the target nucleoside. Thus, we found only one investigation that examined the phosphorolysis of adenosine and inosine in the presence of *Ec*PNP at varied pH values. It was devoted to studying the mechanism of PNP catalysis and the substrate-enzyme complex transition state having considerable oxocarbonium ion-like character using alpha-deuterium isotope effects [27]. Determining enzyme activity at different pH values has become a standard mandatory procedure in the characterization of newly discovered enzymes—the pH optimum is determined, as well as the pH values at which enzyme activity is minimal and, less commonly, the range of pH values at which the enzyme shows some residual activity before its inactivation.

In the present work, we investigated the effect of pH on the course of enzymatic transglycosylation of 6(4)-oxo- or 6(4)-amino purine (pyrimidine) derivatives in the presence of *Escherichia coli* purine nucleoside phosphorylase (*Ec*PNP) and *Lactobacillus leichmanii* Nucleoside deoxyribosyltransferase type II (*Ll*NDTII).

**Figure 2:**
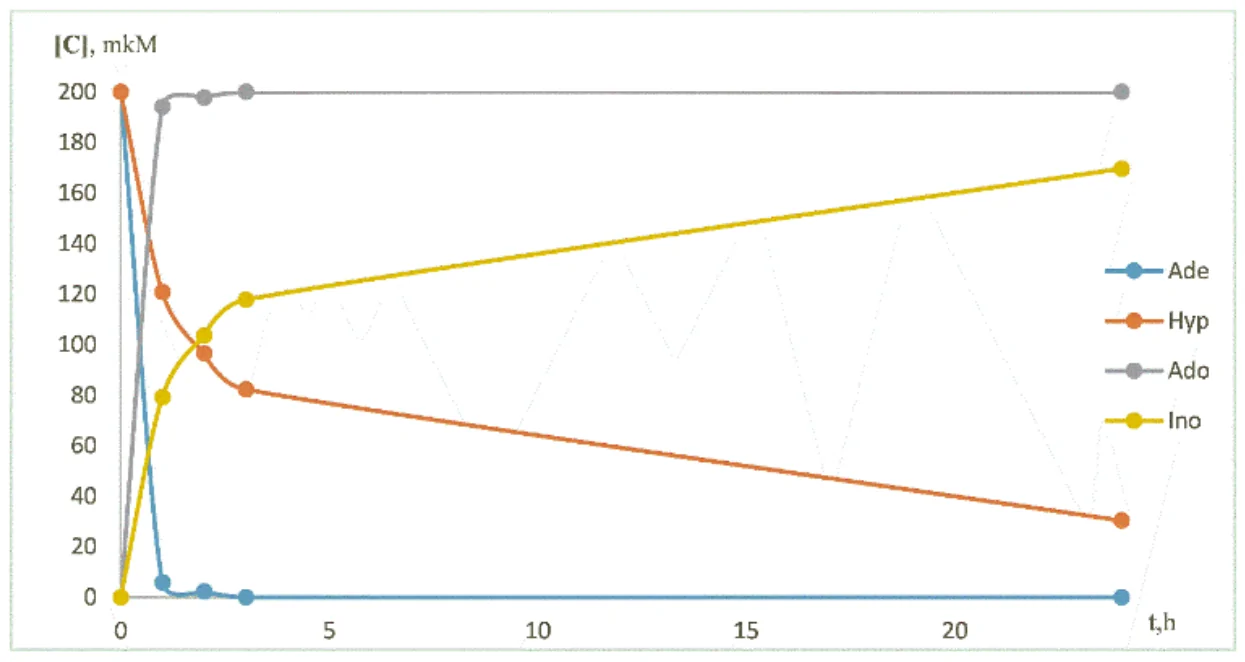
Conversion of Ade and Hyp and synthesis of Ado and Ino. Conditions: 2 μL *Ec*PNP (100 U/mL), 50 mM NaOAc, pH 5.5, 37 °C.

**Scheme 1:**
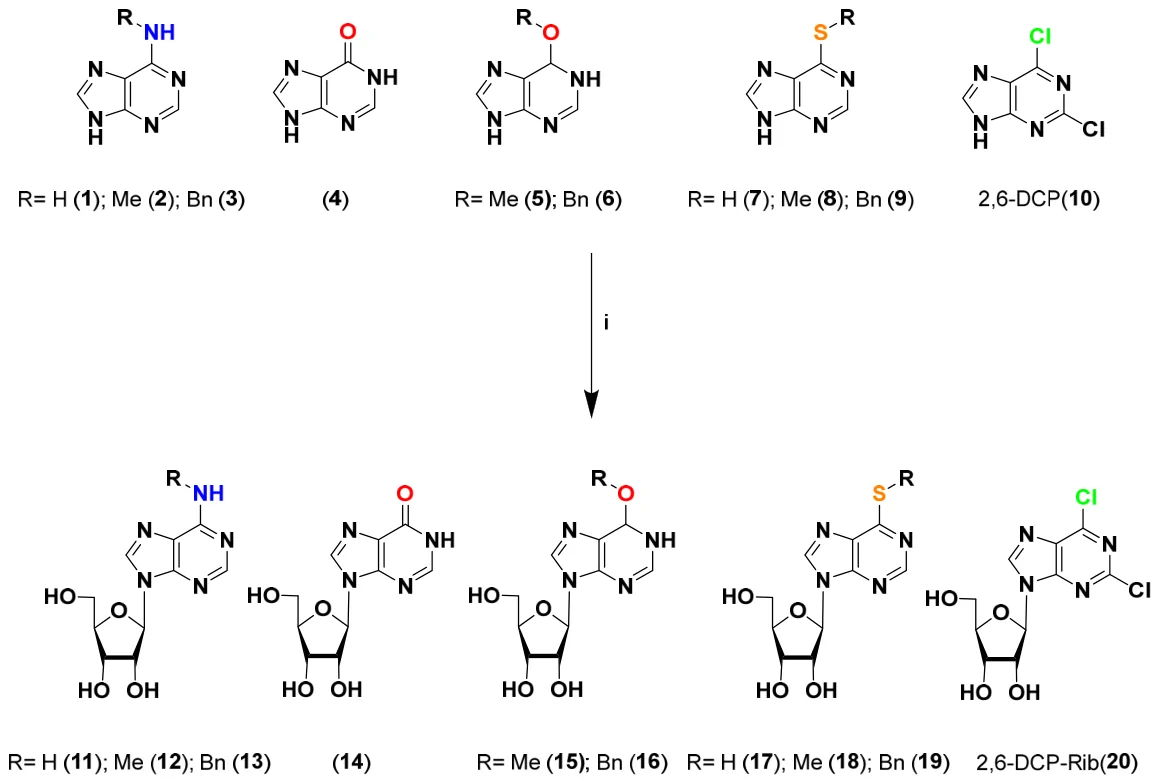
NP-catalyzed reactions of nucleoside formation. Reagents and conditions: (i) *Ec*PNP (0.3 mM 7-mGuo: 0.2 mM Base: 0.1 Pi (1.5:1:0.5)), 37 °C, 50 mM NaOAc (pH 5.5); 50 mM Tris-HCl, (pH 7.0); 50 mM Tris-HCl, (pH 8.5). Me: –CH_3_, Bn: –CH_2_C_6_H_5_.

To study the effect of pH, adenine (Ade, **1**), hypoxanthine (Hyp, **4**), 6-mercaptopurine (6-MP, **7**) and 2,6-dichloropurine (2,6-DCP, **10**) (Scheme 1) were selected, containing an amino, carbonyl, sulfur or halogen substituent in the 6th position, respectively. The change in pH value had virtually no effect on the adenosine (Ado, **11**) synthesis rate, except at pH 5.5, where a slight decrease in the rate of synthesis was observed. But even in this case, the conversion of Ade was complete within 3 h. We observed the opposite outcome in the case of Hyp: a rather significant rate drop at pH 5.5 (Figure 2), after 24 h the conversion of Hyp was about 85% (Table 1).

Nevertheless, at pH 7.0 and 8.5, the rate of inosine (Ino, **14**) synthesis was practically equal to the rate of Ado synthesis. To verify the discriminating effect (or lack thereof) of the 6-oxo group on the enzymatic glycosylation rate, we obtained a series of *O*^6^-, *N*^6^- and *S*^6^-substituted (methyl- and benzyl-) derivatives of Hyp (**5**, **6**), Ade (**2**, **3**), and 6-MP (**8**, **9**), respectively, with which we performed transglycosylation. During the synthesis of *O*^6^-benzyl-inosine (*O*^6^-BnIno, **16**) we observed a significant increase in its formation rate at pH 5.5 compared to that of Hyp. Thus, complete conversion of *O*^6^-BnHyp (**6**) occurred in approximately 40 min (while in the case of *N*^6^-BnAdo (**13**), it took only approx. 3 h). Transglycosylation of the *O*^6^-methyl-hypoxanthine (*O*^6^-mHyp, **5**), which has the smallest possible alkyl element, also showed a positive effect of the substituent on the reaction rate. In this case, the rate of *O*^6^-mIno (**15**) formation was higher than that of *N*^6^-mAdo: approx. 68% in 20 min and 81% in 1 h, respectively (at pH 7.0 and 8.5, complete *O*^6^-mHyp conversion occurred in 1.5 h, and *N*^6^-mAde conversion in 2.5 h, Tables S3 and S5). The proposed mechanism of catalysis by *Ec*PNP suggests [28] that the hydroxyl group of the Asp204 residue must be protonated so that this proton is subsequently transferred to the N7 atom of the heterocyclic base. According to other data [29], an additional hydrogen bond between the carbonyl group of the Asp204 residue and the carbonyl oxygen atom of the 6-keto group is also required for the productive orientation of the heterocyclic moiety of the substrate. It is possible that, at a reaction pH of 5.5, the stabilization of hypoxanthine in the active site is impeded, which may explain the sharp drop in the rate of its glycosylation. Conversely, the introduction of a substituent at the *O*^6^-position of hypoxanthine, by preventing binding or other interactions between the O6 of hypoxanthine and Asp204 at a reaction pH of 5.5, may contribute to the further stabilization of the heterocyclic base in the active site through the formation of non-specific π–π interactions involving Phe159 and Tyr160, leading to a sharp increase in the reaction rate (Table 1).

Studies conducted with 6-MP and its methyl (**8**) and benzyl (**9**) derivatives showed only a slight increase in the *S*^6^-benzyl-mercaptopurine riboside (**17**) formation rate compared to the *S*^6^-methyl derivative (**18**) at pH 5.5 and 7.0 (Table S4).

Similar to guanosine, several tautomeric forms are possible in inosine: three amino-oxo, and one imino-hydroxy. The change in the tautomeric form of the Hyp moiety does not affect the conformation of its nucleoside. The rate constant of the water-assisted proton transfer in inosine from the keto-enamine to enol-imine form is significantly large to generate imino-hydroxy tautomer (k = 5.64 × 10^1^ s^−1^), whilst the time necessary to reach the equilibrium concentration of the 6-keto and 6-hydroxy tautomeric forms is 2.10 × 10^−6^ s [30].

As azole prototropic tautomerism, which creates N7H and N9H forms, is typical of purine base derivatives [31,32,33], we used the B3LYP-D4/6-311++G(d,p)/CPCM(water) method to find the differences in electronic energy (ΔE) and Gibbs free energy (ΔG) between the two tautomeric forms of the Hyp and 6-MP derivatives we were studying. Binding within the active site of *Ec*PNP and, as a result, the rate of enzymatic glycosylation can be influenced by the relative thermodynamic stability of these forms [33]. For Ade, which reacts efficiently with *Ec*PNP, the calculated relative energy of the N9H form is lower than that of the N7H form [34]. Both forms are energetically similar [33,35] in the case of Hyp, which manifests a significantly reduced conversion rate. The conversion rates of Hyp and 6-MP into their corresponding ribosides by *Ec*PNP are lower at pH 5.5 than those of the *O*^6^-/*S*^6^-derivatives studied, as shown in Table 1 and Table S4. The methyl and benzyl substituents stabilize the N9H form, according to the computation results (Table 2), and could enhance the effectiveness of enzymatic glycosylation. Mercaptopurine was the sole compound for which the calculated relative energy of the N7H tautomer was lower than that of the N9H form. This may be one of the reasons why it demonstrates the lowest efficiency in enzymatic glycosylation among all the compounds studied. These results suggest a correlation between the relative stability of the N7H and N9H tautomers of purine bases and the efficiency of their enzymatic glycosylation by *Ec*PNP. For 6-mercaptopurine, calculations carried out considering both the aqueous solution and the gold nanoparticles show that the experimental Raman spectrum is dominated by two tautomeric forms of the thione—thione (N1,N7) and thione (N1,N9), which are approximately equal in energy, while the authors did not require the thiol forms of 6-mercaptopurine to explain the observed spectral pattern [36].

When conducting studies with 2,6-DCP, as expected, the lowest synthesis rate of 2,6-dichloropurine riboside (2,6-DCP-Rib, **20**) was observed at pH 5.5: 20 h after the initiation of the reaction, the yield of the target nucleoside was only 11%, while after 2.5 h at pH 7.0 and 8.5, the yield was 96.7% and 71.6%, respectively. The rate of the glycosyl donor 7-methylguanosine (7-mGuo) concentration decrease in all reactions performed rises from pH 5.5 to 8.5. This fact is apparently associated with the occurrence of side reactions of the nucleoside’s imidazole cycle non-specific cleavage [37,38]. Based on the obtained results, it can be concluded that conducting the reaction at pH 5.5 allows for almost 100% of the glycosyl donor target consumption for the target nucleoside formation, despite the reaction rate decreasing (in the case of hypoxanthine, more significantly). When transglycosylation is carried out at pH 7.0, the partial non-specific consumption of the nucleoside donor takes place due to the destruction of its imidazole cycle, and at pH 8.5 the donor is destroyed quite rapidly. If we disregard the consumption and/or non-targeted destruction of the donor, it does not seem advisable to carry out enzymatic transglycosylation at pH 5.5, especially in the case of hypoxanthine derivatives with a free carbonyl group at the 6th position of the heterocycle.

**Scheme 2:**
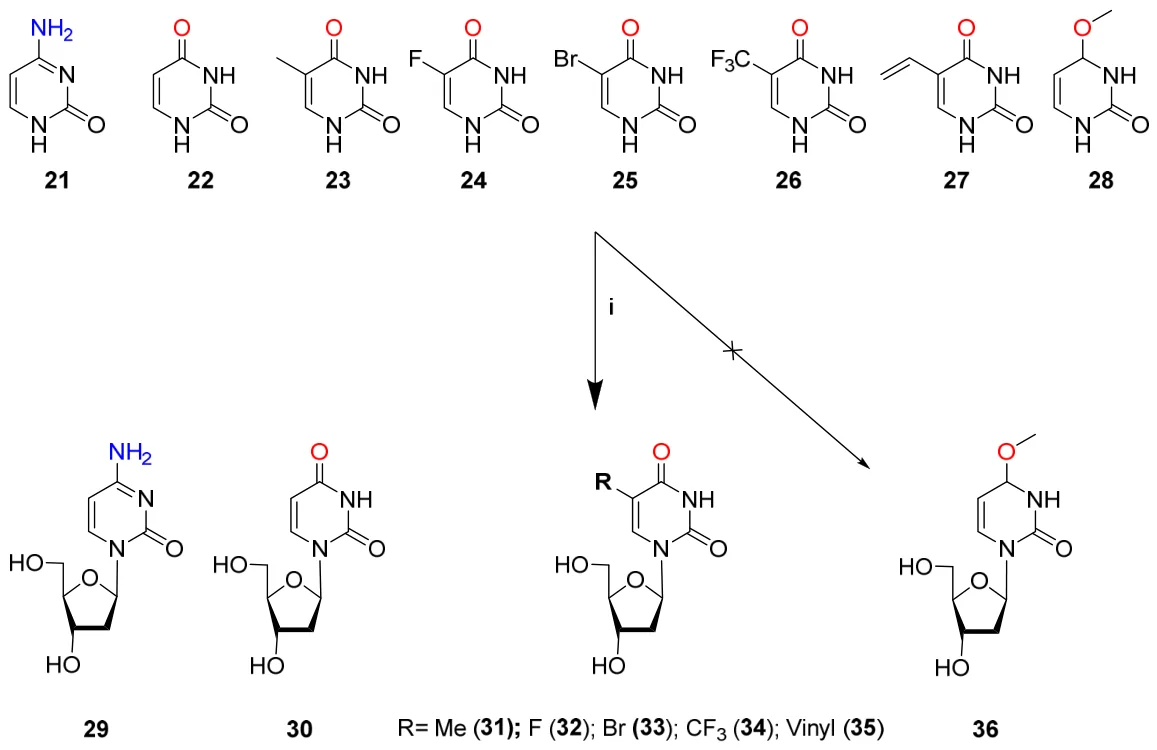
NDT II-catalyzed reactions of nucleoside formation. Reagents and conditions: (i) *Ll*NDTII, 0.1 mM Base: 0.3 mM 7-mdGuo (1:3), 20 °C, 50 mM NaOAc (pH 5.5), 50 mM Tris-HCl, (pH 7.0) or 50 mM Tris-HCl, (pH 8.5).

Natural nucleobases cytosine (Cyt, **21**), uracil (Ura, **22**), and thymine (Thy, **23**) and modified heterocyclic bases 5-fluorouracil (5-FUra, **24**), 5-bromouracil (5-BrUra, **25**), 5-trifluoromethyluracil (5-CF_3_Ura, **26**), 5-vinyluracil (5-VinUra, **27**), and *O*^4^-methyluracil (*O*^4^-mUra, **28**) (Table 2, Scheme 2) were used as acceptors. These heterocyclic bases were chosen to study the influence of the size and nature of the substituents at the *O*^4^- and 5th positions of uracil on the course of enzymatic transglycosylation at different pH values, as well as for their practical importance: some synthetic analogues of 2-deoxyuridine are currently used in clinical practice as drugs [39,40] and may be used as nucleoside labels [41].

The results in Table 2 show that the pH of the reaction mixture can affect the reaction rate and product yields. For Cyt, the maximum reaction rate was achieved at pH 7.0, the reaction proceeded more slowly at pH 5.5, in both cases quantitative yields were obtained. At pH 8.5, the slowest reaction rate was observed, as well as a slight decrease in yield. For uracil derivatives, which contain a carbonyl group at the 4th position, the highest transglycosylation rate is observed at pH 5.5, and the lowest at pH 8.5. An effect of pH on the reaction rate and yield was also found in the presence of a substituent at the 5-position of uracil.

Maximum yields were observed at pH 7.0, with yields decreasing by approximately 10% at pH 5.5 and significantly decreasing at pH 8.5. The results of the study of the influence of pH on the transglycosylation reaction carried out with purine heterocyclic bases (Table 2) showed a similar trend as with pyrimidine compounds, but not as with transglycosylation catalyzed by *Ec*PNP. For Hyp, the reaction proceeded faster at pH 5.5 than at pH 7.0, and no reaction was observed at pH 8.5. However, unlike *O*^4^-mUra, the methylated carbonyl group of Hyp does not hinder the reaction. This can be explained by the ability of the terminal carboxylate of Tyr157′ to form hydrogen bonds with the N7 atom of the purine. In the case of pyrimidine bases, a hydrogen bond is formed between the carboxyl group at the C-terminus and O4 [42]. The absence of changes in the rate and yield of reactions carried out with adenine at different pH values is additional evidence that the presence of a carbonyl group in the structure of the purine heterocyclic base-acceptor makes the rate and yield of enzymatic transglycosylation dependent on the pH value of the reaction medium.

During the experiments to determine the concentration of *Ll*NDTII, it was discovered that the reaction yields at pH 8.5 were unusually dependent on the concentration of enzyme used, in addition to the expected change in the enzymatic transglycosylation rate at pH 5.5 and 7.0 (Table 2). This fact was also revealed by our colleagues [43] while this article was being prepared. Therefore, additional studies of transglycosylation reactions catalyzed by *Ll*NDTI were carried out with the previously considered heterocyclic bases-acceptors and donor at pH 8.5, but with an increased enzyme concentration of 26 × 10^−3^ U (Table 3). The enzyme concentration was selected based on the obtained data (entry 5, Table S7) showing that this is the lowest concentration at which the maximum yield of the test reaction of 5-hmUra with 7-mdGuo is achieved at pH 8.5.

The obtained data at pH 8.5 (Table 4) indicate that at an enzyme concentration of 26 × 10^−3^ U, in contrast to 7.8 × 10^−3^ U, quantitative yields are achieved in reactions with heterocyclic bases Cyt, Ura, Thy, 5-BrUra, Hyp, and *O*^6^-mHyp. Under these conditions, the conversion of 5-VinUra reached 88%, which is higher than the conversion of this base (using 7.8 × 10^−3^ U of enzyme) at both pH 5.5 and 7.0 (entry 16, Table 2). This may be due to the very low reaction rate, since monitoring its progress and determining the equilibrium state are complicated. Therefore, the lower yields at pH 5.5 and 7.0 may be the result of the reverse hydrolysis reaction. The reaction at pH 8.5 completely consumed the donor, so it is possible that increasing the acceptor:donor ratio could lead to even higher yields. In this case, the difference in yields at different pH values is independent of the reaction rate and target nucleoside hydrolysis.

**Table 4:** Enzymatic synthesis of 2′-deoxynucleosides using *Ll*NDTII at pH 8.5 ^(a)^.

Base Acceptor	Enzyme, U	t, min	Yield (HPLC), % ^(b)^
Cyt	26 × 10^−3^	15	100 ^(c)^
Ura	26 × 10^−3^	35	100 ^(c)^
Thy	26 × 10^−3^	50	100 ^(c)^
5-FUra	26 × 10^−3^	15	31
	52 × 10^−3^	70	100 ^(c)^
5-BrUra	26 × 10^−3^	35	100 ^(c)^
5-CF_3_Ura	26 × 10^−3^	150	25
	52 × 10^−3^	1 day	86 ^(d)^
5-VinUra	26 × 10^−3^	300	88 ^(d)^
Hyp	26 × 10^−3^	190	100 ^(c)^
*O*^6^-mHyp	26 × 10^−3^	15	100 ^(c)^

^(a)^ Reaction conditions: *Ll*NDT II, 0.1 mM Base: 0.3 mM 7-mdGuo (1:3), 20 °C, 50 mM NaOAc (pH 5.5), 50 mM Tris-HCl, (pH 7.0), or 50 mM Tris-HCl, (pH 8.5); ^(b)^ The yield (HPLC) was calculated as the ratio of the target nucleoside equilibrium concentration to the limiting substrate initial concentration; ^(c)^ The peak signal of the heterocyclic base was not detected; ^(d)^ The yield is determined by the complete depletion of the donor.

In contrast to the heterocyclic base-acceptors conversions previously studied at an enzyme concentration of 26 × 10^−3^ U (Table 3), reactions with 5-FUra and 5-CF_3_Ura yielded modest yields of 31% and 25%, respectively. Doubling the enzyme activity in the reaction mixture to 52 × 10^−3^ units resulted in a quantitative yield for the reaction with 5-FUra and an 86% yield for 5-CF_3_Ura. In the case of the latter, complete consumption of the donor nucleoside was observed, as in the case of the reaction with 5-VinUra. Thus, when performing transglycosylation reactions at pH 8.5, a decrease in the reaction rate is observed. This favourably affects the ability to study the course of analytical reactions, since the reverse hydrolysis reaction is also slowed. At the same time, a higher enzyme concentration at pH 8.5 contributes to achieving the maximum possible yields. However, when performing preparative transglycosylation reactions catalyzed by *Ll*NDTII, the forward and reverse reactions are already slower, especially with pyrimidine bases as acceptors, so it is not advisable to use buffer solutions with a pH of 8.5. Enzymatic transglycosylation at pH 5.5 is also not preferable, since reduced yields are observed in many studied reactions with different heterocyclic bases. We suppose the optimal pH value for scaling up transglycosylation reactions is 7.0.

## 3. Materials and Methods

The solvents and materials were reagent grade and were used without additional purification. Column chromatography was performed on silica gel (Kieselgel 60 Merck KGaA, Darmstadt, Germany, 0.040–0.063 mm). All reagents and solvents were purchased from Sigma Aldrich (Sigma-Aldrich, Inc., St. Louis, MO, USA) and used without additional purification. TLC was performed on an Alugram SIL G/UV254 (Macherey-Nagel, Düren, Germany) with UV visualization. Purine and pyrimidine bases were obtained from the Engelhardt Institute of Molecular Biology nucleoside collection. 7-mdGuo was obtained according to the previously elaborated procedure [14]. Thymidine (Sigma-Aldrich #89270), purine nucleoside phosphorylase *E. coli* (Sigma-Aldrich #N2415, EC 2.4.2.1), and *Lactobacillus leichmannii* nucleoside deoxyribosyltransferase II (Sigma-Aldrich #N2665, EC 2.4.2.6) were purchased from Sigma-Aldrich. 

### 3.1. Selecting a Nucleoside Donor, Initial Concentrations and Molar Ratios of Substrates

Enzymatic transglycosylation is an equilibrium process; therefore, the reaction conditions were selected to maximize the shift in equilibrium towards the target nucleoside. For *Ec*PNP-catalyzed reactions, 7-methylguanosine (7-mGuo) and a molar ratio of substrates of 1.5:1:0.5 (Nuc:Base:Pi) were selected, conditions that had previously shown excellent results [16]. For *Ll*NDTII, we previously considered various donors (Thd, 2′-dIno and 7-methyl-2′-deoxyguanosine (7-mdGuo)) and donor-acceptor ratios of 1:1, 1:3, and 1:6 [44]. The use of 7-mdGuo also allows transglycosylation reactions with 5-substituted uracil derivatives and purine bases to be carried out with high yields, so 7-mdGuo was chosen for the study as the optimal carbohydrate residue donor in a 3-fold excess (Table S6).

### 3.2. Enzymatic Synthesis of Nucleosides (For Analysis, General Method)

The reactions were carried out in 1.5 mL plastic tubes (Eppendorf, Hamburg, Germany), the volume of the reaction mixture was 1 mL in 50 mM Tris—HCl or NaOAc (at pH 5.5, 7.0, and 8.5) buffer at 37 °C, with substrate concentrations of Base:Donor:Phosphate in the range 0.1–0.6 mM. The molar concentration ratio was 1:1.5:0.5 (Base:Donor:Phosphate) for *Ec*PNP and 1:1 or 1:3 (Base:Donor) for *Ll*NDTII. The reactions were catalyzed by the addition of 0.12 units (U) *Lactobacillus leichmanii* NDT II (*Ll* NDT II) or 0.3 U of *Ec*PNP.

Enzymatic transglycosylation reactions were performed until equilibrium was established.

### 3.3. Choosing pH Values

When choosing pH values for the study, we were guided by literature data, which determined optimal pH values, as well as values at which enzyme activity was practically undetectable. Thus, pH values of 5.5, 7.0, and 8.5 were selected, lying within the minimum and optimum activity ranges for *Ec*PNP [45] and *Ll*NDTII [46]. When selecting a buffer system, we were guided by reliability and the minimal (or negligible) effect on the enzymatic activity of the enzymes used. The Tris-HCl buffer has a limited pH range (app. pH 7.0–9.2) within which it exhibits satisfactory buffer capacity. For pH 5.5 we used sodium acetate buffer (app. pH 3.6–5.6), which is also widely used in work relating to purification and precipitation of nucleic acids, enzyme crystallization and catalysis. To further check the possible influence (or its exclusion) of the buffer components on the enzyme activity at pH 5.5, a citrate buffer was also used to carry out the transglycosylation of Hyp + 7mGuo at pH 5.5.

### 3.4. Adjusting of Enzyme Quantity for Transglycosylation Catalyzed by LlNDTII

According to a prior study [44] on enzymatic transglycosylation catalyzed by *Ll*NDTII, the synthesis of target nucleosides proceeds at varying rates with the same quantity of enzyme in the reaction mixture, contingent on the acceptor base’s structure. Therefore, to allow convenient monitoring of reactions and determination of the time difference to reach equilibrium at different pH values, the optimal amount of enzyme was selected using the example reaction of 5-hydroxymethyluracil (5-hmUra) with 7-mdGuo. This reaction occurs at an average rate (not too fast and not too slow), relative to other heterocyclic bases (Table S7). Since transglycosylation is a reversible reaction, the reverse reaction of the target nucleoside glycosidic bond hydrolysis begins immediately after equilibrium is reached. The data from Table S2 show that the amount of enzyme, added to the reaction mixture, predictably affects the enzymatic reaction rate. Increasing enzyme concentration increases both the rate of transglycosylation and the hydrolysis rate. Therefore, for analytical reactions (with low substrate concentrations) to capture the equilibrium state, it is preferable to use lower enzyme concentrations. However, at the lowest enzyme activity studied by us (2.6 × 10^−3^ U), transglycosylation proceeds too slowly, which makes it difficult to determine the heterocyclic base maximum conversion and reach the reaction plateau. Moreover, a lower yield was observed 24 h after the reaction was initiated; thus, it can be assumed that by this time the target nucleoside had already been partially hydrolyzed. Hence, for further studies of transglycosylation reactions with other heterocyclic bases, an enzyme activity of 7.8 × 10^−3^ U was selected, i.e., the minimum activity that allows for the most accurate determination of the equilibrium state. To ensure that pH does not affect the efficiency of the nucleoside donor used, a series of reactions was performed also with Thd as the glycosyl donor and 5-hmUra, varying the enzyme concentration. The data obtained (Table S8) showed that donor efficiency is independent of pH, and the same reaction patterns are observed regardless of the nucleoside donor used.

### 3.5. HPLC Analytical Experiments

An HPLC analysis was performed using an Akvilon (Moscow, Russia) HPLC system (2 × Stayer pumps (2nd series), a Stayer MS16 dynamic mixer and a Stayer 104 M UV-Vis detector. Enzymatic reactions were assessed by HPLC analysis (elution conditions and details can be found in the Supplementary Material). HPLC analysis was performed with a 20 μL analytical volume. HPLC analysis was run in a linear acetonitrile gradient in 0.06% (*v*/*v*) TFA/deionized water. The detailed analytical methods, the columns, and sorbents used, as well as the gradient rates and retention times of the compounds, are described in the Supplementary Material.

### 3.6. Software and Calculating

The Molecular Operating Environment (MOE, version 2019.0102; Chemical Computing Group, Montreal, QC, Canada) software package was used for molecular structure preparation, preliminary energy minimization using the PM3 method, and conformational searches (gradient convergence criterion: 0.01 kcal·mol^−1^·Å^−1^) [47]. The maximum number of iterations for the conformational search was 1000, while the maximum number of iterations for molecular mechanics minimization was 500. The lowest-energy conformers of the N7H and N9H tautomers were searched for using the LowModeMD method, with energy minimization employing the Amber10:EHT force field and the R-field solvent model.

Quantum chemical calculations were performed using the ORCA software package (ORCA, version 6.1.1; FACCTs GmbH, Recklinghausen, Germany) [48] within the framework of density functional theory (DFT) [49]. Molecular geometry optimization and the calculation of thermodynamic properties were carried out using the B3LYP hybrid functional with the D4 dispersion correction [50] and the 6-311++G(d,p) basis set. The effect of the aqueous environment was accounted for using the conductor-like polarizable continuum model (CPCM) [51]. Geometry optimization and convergence of the self-consistent field procedure were controlled using the TightOpt and TightSCF settings, respectively. Following geometry optimization, vibrational frequencies and thermochemical properties were calculated.

## 4. Conclusions

Based on the results, it can be concluded that the discriminatory effect of the hypoxanthine *O*^6^-oxo group on the rate of enzymatic transglycosylation in the reaction catalyzed by *Ec*PNP at pH 5.5 is essentially eliminated when a substituent is added. A bulky lipophilic substituent may affect binding in the active centre of *Ec*PNP and influence the reaction rate, as in the case of *O*^6^-BnHyp and *S*^6^-BnMP-Rib. Studies conducted with 6-MP and its derivatives showed an increase in *S*^6^-benzyl-mercaptopurine riboside formation rate compared to the *S*^6^-methyl derivative at pH 5.5 and 7.0. When the acceptor base is adenine, however, we observe only a negligible effect of substituents at the *N*^6^ position on the rate of transglycosylation, compared with a free amino group.

The results obtained from *Ll*NDTII-catalyzed transglycosylation also indicate that the pH of the reaction mixture affects the reaction rate and product yield. For Cyt, which has a free amino group at the *N*^4^ position, the maximum reaction rate was achieved at pH 7.0; at pH 5.5, the reaction proceeded more slowly; nevertheless, quantitative yields were obtained in both cases. At pH 8.5, the lowest rate of cytidine formation was observed, along with a slight decrease in yield. For uracil derivatives containing a free carbonyl group at the 4th position, the highest rate of transglycosylation is observed at pH 5.5, and the lowest at pH 8.5. A change in the reaction rate was also observed depending on the nature of the substituent at the 5-position of uracil. Furthermore, a dependence of the yield of the target nucleoside on the concentration of *Ll*NDTII was found.

Conducting enzymatic transglycosylation at pH 5.5 allows for almost 100% consumption of the target glycosyl donor for target nucleoside synthesis, despite the reaction rate decreasing (which is more significant in the case of hypoxanthine). The rate of the glycosyl donor 7-methylguanosine (7-mGuo) concentration decrease in all reactions performed rises from pH 5.5 to 8.5. If we disregard the consumption and/or non-targeted depletion of the donor, it does not seem advisable to carry out enzymatic transglycosylation at 5.5, especially in the case of hypoxanthine derivatives with a free carbonyl group at the 6th position of the heterocycle.

## Figures and Tables

**Table 1:** Enzymatic synthesis of nucleosides using *Ec*PNP at different pH values ^(a)^.

Entry	Base Acceptor/Nucleoside Product	HPLC Defined Yield,% ^(b)^			
		t, h	pH 5.5	pH 7.0	pH 8.5
1	Ade/Ado (**1**)/(**11**)	0	-	-	-
2		1	97.1	100 ^(c)^	100 ^(c)^
3		2	98.9		
4		3	100 ^(c)^		
5		24.5			
6		50			
7	Hyp/Ino (**4**)/(**14**)	0	-	-	-
8		1	39.6	88.3	90.2
9		2	51.8	97.4	95.6
10		3	58.9	100 ^(c)^	100 ^(c)^
11		24.5	84.8		
12		50	91.3 ^(d)^		
13	*N*^6^-BnAde/*N*^6^-BnAdo (**3**)/(**13**)	0	-	-	-
14		1 h 20 min	97.6	100 ^(c)^	100 ^(c)^
15		2 h 50 min	99.4		
16		4 h 20 min	100 ^(c)^		
17	*O*^6^-BnHyp/*O*^6^-BnIno (**6**)/(**16**)	0	-	-	-
18		2 h 40 min	100 ^(c,e)^	100 ^(c)^	100 ^(c)^
19	2,6-DCP/2,6-DCP-Rib (**10**)/(**20**)	0	-	-	-
20		1 h 20 min	6.2	68.5	49.3
21		2.5	8.5	96.6	71.6
22		20	11.0	100 ^(c)^	100 ^(c)^

^(a)^ Reaction conditions: *Ec*PNP 100 U/mL (3 μL for *N*^6^-BnAde, *O*^6^-BnHyp, 2,6-DCP; 2 μL for Ade, Hyp) (0.3 mM 7-mGuo: 0.2 mM Base: 0.1 Pi (1.5:1:0.5)), 37 °C, 50 mM NaOAc (pH 5.5); 50 mM Tris-HCl, (pH 7.0); 50 mM Tris-HCl, (pH 8.5); ^(b)^ The yield (HPLC) was calculated as the ratio of the target nucleoside equilibrium concentration to the limiting substrate initial concentration; ^(c)^ The peak signal of the heterocyclic base was not detected; ^(d)^ After 48 h, the residual *Ec*PNP activity was approximately 5%; ^(e)^ At pH 5.5, the reaction proceeded quantitatively in less than 40 min. For more detailed table containing equilibrium concentrations see the Supplementary Material.

**Table 2:** Electronic energy differences (Δ*E* = *E*^N7H^ − *E*^N9H^) and Gibbs free energy differences (Δ*G* = *G*^N7H^ − *G*^N9H^) between the N7H and N9H forms of Hyp/*O*^6^-Hyp derivatives, 6-MP/*S*^6^-MP derivatives, and Ade ^(a)^.

Heterocyclic Base	Δ*E* (kcal/mol)	Δ*G* (kcal/mol)
Ade	1.46	1.72
Hyp	0.19	0.16
*O*^6^-mHyp	0.62	0.56
*O*^6^-BnHyp	0.61	0.40
6-MP	−0.63	−0.68
*S*^6^-mMP	1.05	1.03
*S*^6^-BnMP	1.09	1.08

^(a)^ All calculations were performed at the B3LYP-D4/6-311++G(d,p) level of theory using the conductor-like polarizable continuum model (CPCM) for aqueous solvation.

**Table 3:** Enzymatic synthesis of 2′-deoxynucleosides using *Ll*NDTII at different pH values ^(a)^.

Entry	Base Acceptor/Nucleoside Product	HPLC Defined Yield,% ^(b)^			
		t, min	pH 5.5	pH 7.0	pH 8.5
1	Cyt/dCyd (**21**)/(**29**)	20	-	100 ^(c)^	-
2		40	100 ^(c)^	-	-
3		60	-	-	94
4	Ura/dUrd (**22**)/(**30**)	20	100 ^(c)^	-	19
5		40	-	100 ^(c)^	-
6	Thy/dThd (**23**)/(**31**)	20	-	-	13
7		60	91	100 ^(c)^	-
8	5-FUra/5-FdUrd (**24**)/(**32**)	15	-	-	26
9		20	88	-	-
10		40	-	100 ^(c)^	-
11	5-BrUra/5-BrdUrd (**25**)/(**33**)	20	-	-	14
12		40	91	-	-
13		70	-	100 ^(c)^	-
14	5-CF_3_Ura/5-CF_3_dUrd (**26**)/(**34**)	160	61	-	No reaction
15		220	-	57	
16	5-VinUra/5-VindUrd (**27**)/(**35**)	1 day	25	59	9
17	*O*^4^-mUra/*O*^4^-mdUra (**28**)/(**36**)	-	No reaction		
18	Ade/dAdo	20	100 ^(c)^	100 ^(c)^	100 ^(c)^
19	Hyp/dIno	80	100 ^(c)^	-	No reaction
20		160	-	100 ^(c)^	
21	*O*^6^-mHyp/*O*^6^-mdIno	40	-	-	53
22		70	100 ^(c)^	100 ^(c)^	-

^(a)^ Reaction conditions: *Ll*DTII 7.8 × 10^−3^ U, (0.1 mM Base: 0.3 mM 7-mdGuo (1:3)), 20 °C, 50 mM NaOAc (pH 5.5); 50 mM Tris-HCl, (pH 7.0); 50 mM Tris-HCl, (pH 8.5); ^(b)^ The yield (HPLC) was calculated as the ratio of the target nucleoside equilibrium concentration to the limiting substrate initial concentration; ^(c)^ The peak signal of the heterocyclic base was not detected. For a more detailed table containing equilibrium concentrations, see the Supplementary Material.

## Data Availability

The original contributions presented in this study are included in the article/Supplementary Material. Further inquiries can be directed to the corresponding author(s).

## Supplementary Materials

The following supporting information can be downloaded at: https://www.mdpi.com/article/10.3390/ijms27219901/s1.
