# Supplementary material for: The Effects of the Medium’s pH Value on the Efficiency of the Enzymatic Transglycosylation Catalyzed by Escherichia coli Purine Nucleoside Phosphorylase and Lactobacillus leichmanii Nucleoside Deoxyribosyltransferase Type II
Source: Int J Mol Sci. 2026 Nov 5;27(21):9901. doi: 10.3390/ijms27219901 (PMC13763734; doi:10.3390/ijms27219901)
Supplement: Supplementary file 1 [file ijms-27-09901-s001.zip › ijms-4634383-supplementary.pdf]

Supporting Information

The Effects of the Medium's pH Value on the Efficiency of the Enzymatic Transglycosylation Catalysed by *Escherichia coli* Purine Nucleoside Phosphorylase and *Lactobacillus leichmanii* Nucleoside Deoxyribosyltransferase Type II

Cyril S. Alexeev*, Maria A. Konkina, Ivan A. Oskolsky, Dmitrii A. Platov and Mikhail S. Drenichev

Reagents and solutions

All chemicals and solvents were of analytical grade or higher and purchased, if not stated otherwise, from Sigma–Aldrich (United States).

All solutions were prepared in water purified using Milli-Q® water ultrafiltration station (Merck Millipore, United States). pH values were determined with microprocessor-based pH (mV- C) bench meter 211 (Hanna Instruments, Germany), equipped with HI1131B double junction combination pH electrode and HI7662 stainless steel temperature sensor for pH compensation.

K,Na-phosphate buffer solution (50 mM, pH 7.5). 50 mL of 0.1 M KH_2PO_4 and 40.9 mL of 0.1 M NaOH were mixed in 100 mL volumetric flask. The pH value of the solution was adjusted to 7.5 by careful addition of 0.1 M NaOH. The overall volume was brought to 100 mL by addition of water.

K,Na-phosphate buffer solution (5 mM, pH 7.5). For analytical reactions was used 50 mM Tris-HCl buffer diluted 10 times.

Tris – HCl buffer solution (50 mM, pH 7.0/8.5). 3,025 g of tris(hydroxymethyl)aminomethane was dissolved in deionized water in 500 mL volumetric flask. The pH value was adjusted to 7.0 and 8.5 by careful addition of 0.1 M HCl solution. The overall volume was brought to 500 mL by addition of deionized water.

NaOAc buffer solution (50 mM, pH 5.5). 3,4 g of sodium acetate trihydrate was dissolved in deionized water in 500 mL volumetric flask. The pH value was adjusted to 5.5 by careful addition of 0,05 M acetic acid solution. The overall volume was brought to 500 mL by addition of deionized water.

Stock 1/2 mM solutions of compounds were prepared by dissolving 0.025/0.05 mmol of compound in deionized water in 25 mL volumetric flask.

Enzymes. A purine nucleoside phosphorylase *E. coli* (PNP, Sigma-Aldrich N2415) and nucleoside deoxyribosyltransferase type II *Lac. l.* (NDT II, Sigma-Aldrich N2665) were purchased from Sigma–Aldrich (United States).

To a reaction sample solution (1 mL, Table 1) were added 100 U/ml of PNP. The reaction mixture was carried out at 37°C, at one of three different pH-system (5.5/7.0/8.5), and was monitored by HPLC.

Table S1. Volumes of stock and buffer solutions taken for 1 mL of a rection mixture.

1 mM 7-MeGuo stock solution	2 mM acceptor stock solution	5 mM phosphate	50 mM Tris – HCl/NaOAc	$C_{7\text{-MeGuo}}$: C_{acceptor}:C_{Pi}
300 μL	100 μL	20 μL	580 μL	1.5:1:0.5

To a reaction sample solution (1 mL, Table 2) were added NDT II. The reaction mixture was carried out at room temperature, at one of three different pH-system (5.5/7.0/8.5), and was monitored by HPLC.

Table S2. Volumes of stock and buffer solutions taken for 1 mL of a rection mixture.

1 mM acceptor stock solution	1 mM 7-MedGuo/Thd stock solution	50 mM Tris – HCl/NaOAc	C_{acceptor}: $C_{7\text{-MedGuo/Thd}}$
200 μL	200 μL	600 μL	1:1
100 μL	300 μL	600 μL	1:3

HPLC analysis: Analytical HPLC analysis was run using Akvilon (Russia) HPLC system (2×Stayer pumps (2nd series), Stayer MS16 dynamic mixer and Stayer 104M UV-Vis detector).

Important!: The product was eluted after flushing step in gradient program.

Calibration coefficients (α) were calculated using equation:

$$C_{t=0} = \alpha \times S_{t=0},$$

were t = 0 min refers to HPLC run immediately after mixing a substrates sample solution with the enzyme(s) solution. The HPLC data at t = 0 was used for calibration coefficients (α) calculations. Using these values, the equilibrium concentrations were calculated, and the data are presented below, in figures.

Transglycosylation reactions catalysed by PNP.

Adenosine, inosin from 7-methylguanosine (7-MeGuo+Ade, Hyp +P_i, analytical method)

HPLC-analysis of transglycosylation: Cosmosil 5C_{18}-MS-II, 4.6*150 mm, 5μm, 120 Å, (Nacalai Tesque, Inc. (Japan)), 20ºC, elution in modified mobile phase: linear gradient of acetonitrile in 0.06% TFA/deionized water from 2 to 12% for 10 min (flushing with 12-80 % acetonitrile-0.06% TFA/deionized water for 10-10.1 min, then 80-2% for 10.1-10.8 min) at flow rate 1 ml/min with UV detection at wavelength 260 nm, injection volume 20 μL.

Fugure S1.1. pH = 5.5

a) $t_{initial}$

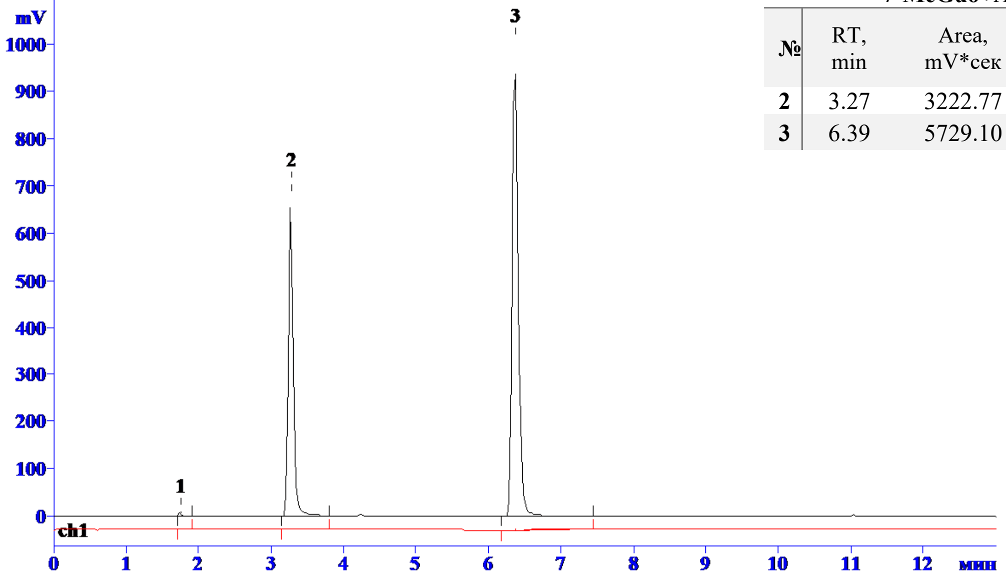

7-MeGuo+Ade+P_i –1.5:1:0.5 – t_0

№	RT, min	Area, mV*сек	Equilibrium concentration, µM	Name
2	3.27	3222.77	200.00	Ade
3	6.39	5729.10	300.00	7-MeGuo

$t_{equilibrium}$

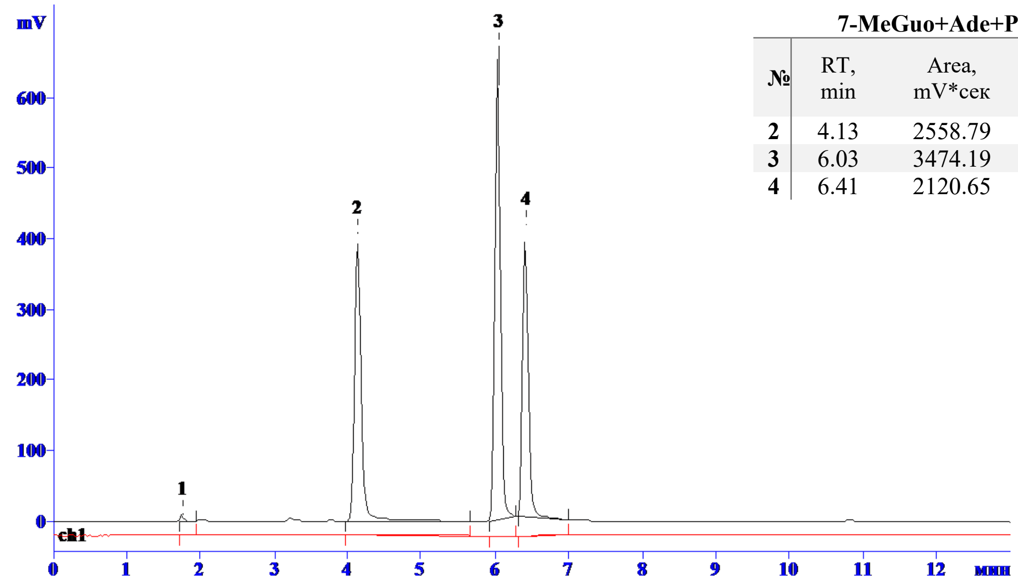

7-MeGuo+Ade+P_i – 1.5:1:0.5– $t_{equilibrium}$

№	RT, min	Area, mV*сек	Equilibrium concentration, µM	Name
2	4.13	2558.79	188.95	7-MeGua
3	6.03	3474.19	200	Ado
4	6.41	2120.65	111.05	7-MeGuo

b) $t_{initial}$

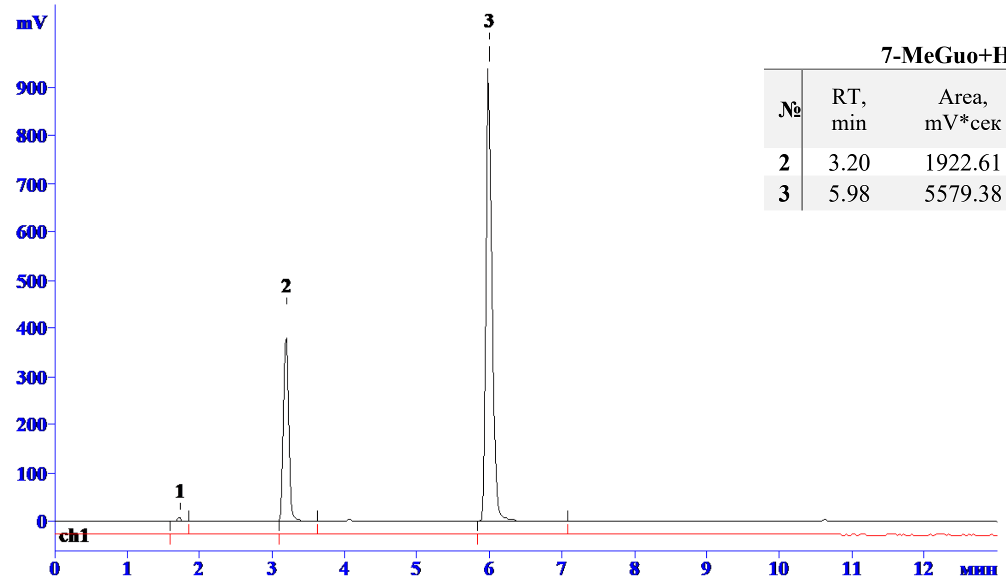

7-MeGuo+Hyp+P_i –1.5:1:0.5 – t_0

№	RT, min	Area, mV*сек	Equilibrium concentration, µM	Name
2	3.20	1922.61	200.00	Hyp
3	5.98	5579.38	300.00	7-MeGuo

tequilibrium

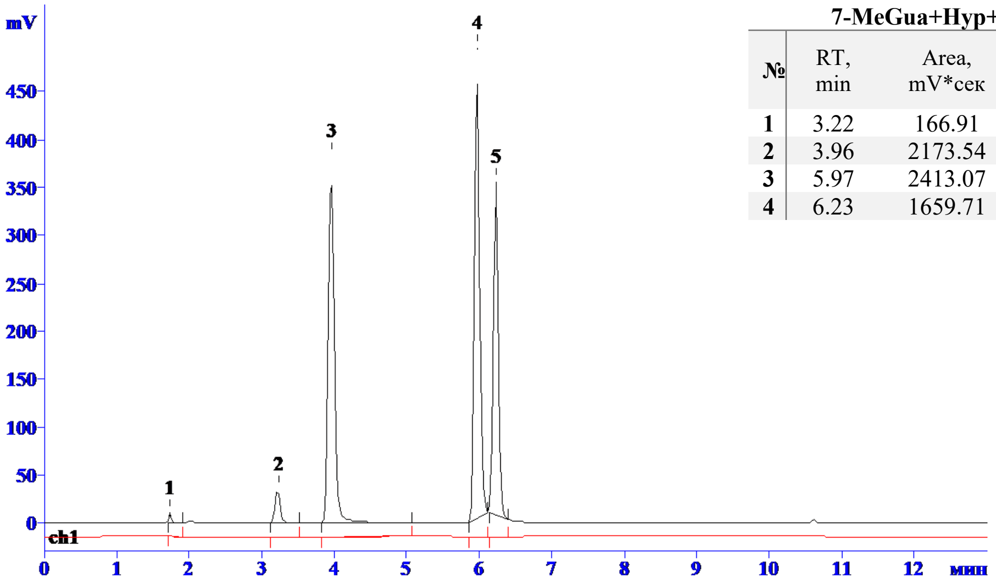

7-MeGua+Hyp+Pᵢ – 1.5:1:0.5– t equilibrium

№	RT, min	Area, mV*сек	Equilibrium concentration, μM	Name
1	3.22	166.91	17.36	Hyp
2	3.96	2173.54	170.25	7-MeGua
3	5.97	2413.07	129.75	7-MeGuo
4	6.23	1659.71	182.64	Ino

Fugure S1.2. pH = 7.0

a) tinitial

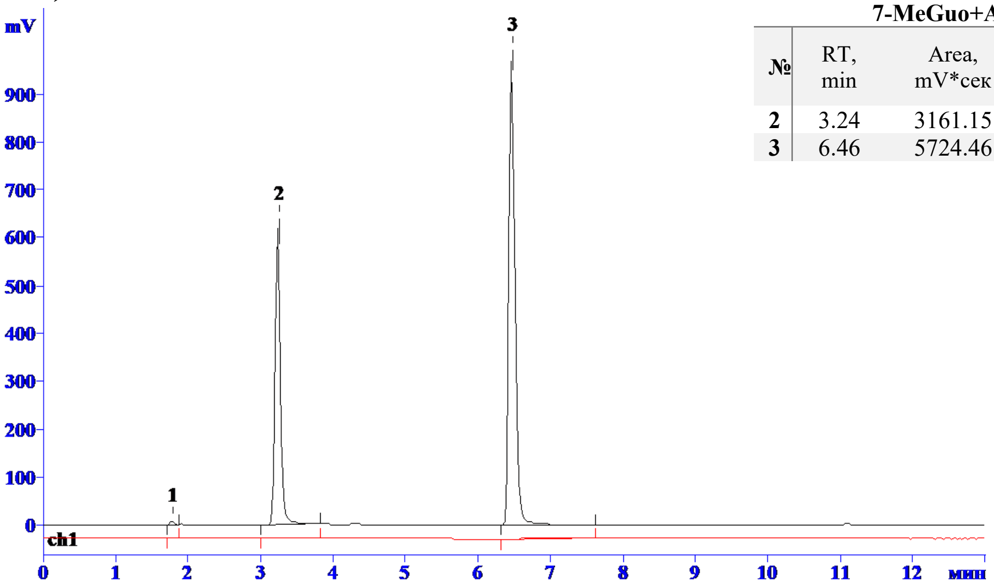

7-MeGuo+Ade+Pᵢ –1.5:1:0.5 – t0

№	RT, min	Area, mV*сек	Equilibrium concentration, μM	Name
2	3.24	3161.15	200.00	Ade
3	6.46	5724.46	300.00	7-MeGuo

tequilibrium

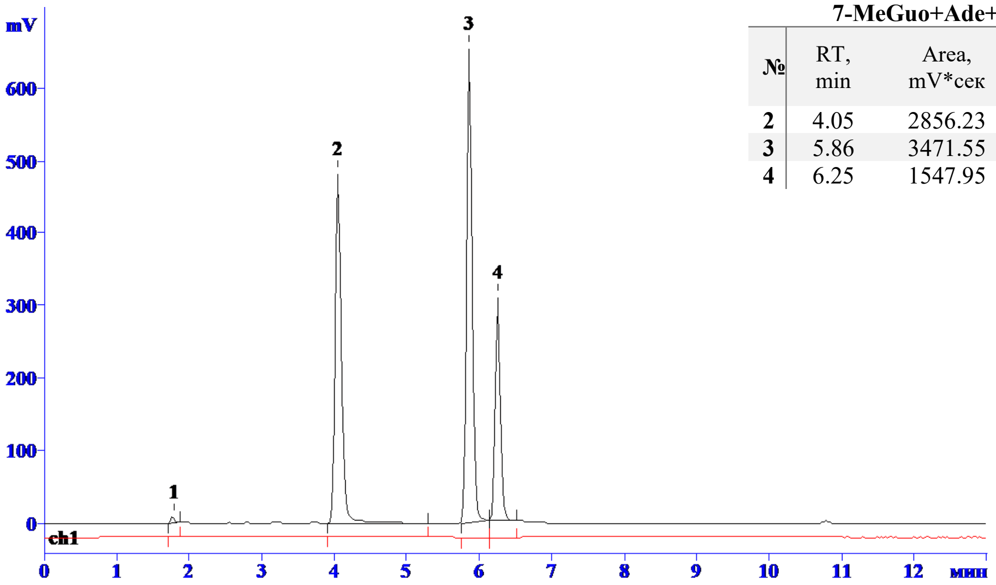

7-MeGuo+Ade+Pᵢ – 1.5:1:0.5– t equilibrium

№	RT, min	Area, mV*сек	Equilibrium concentration, μM	Name
2	4.05	2856.23	218.88	7-MeGua
3	5.86	3471.55	200	Ado
4	6.25	1547.95	81.12	7-MeGuo

b) $t_{initial}$

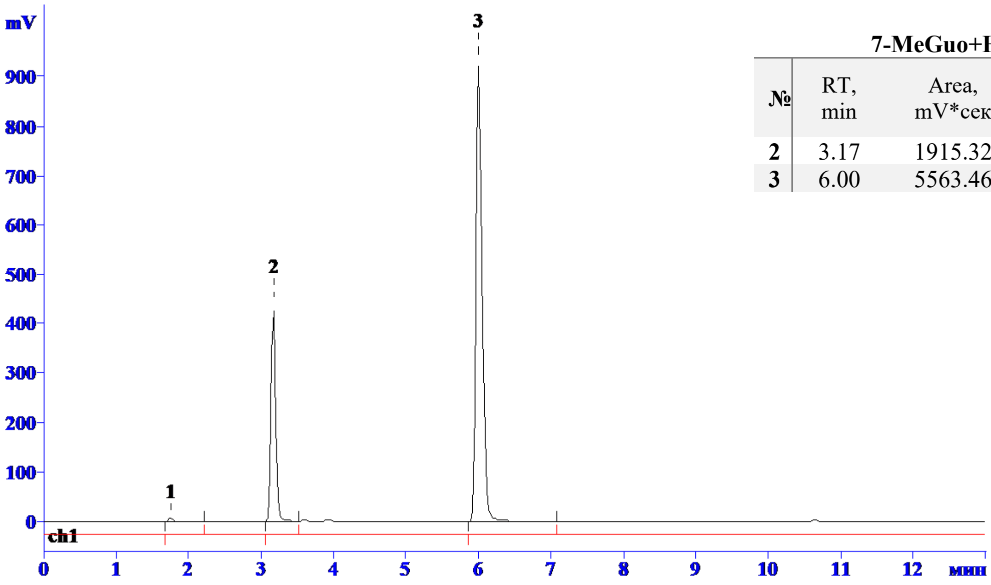

7-MeGuo+Hyp+P_i –1.5:1:0.5 – t_0

№	RT, min	Area, mV*сек	Equilibrium concentration, μM	Name
2	3.17	1915.32	200.00	Hyp
3	6.00	5563.46	300.00	7-MeGuo

$t_{equilibrium}$

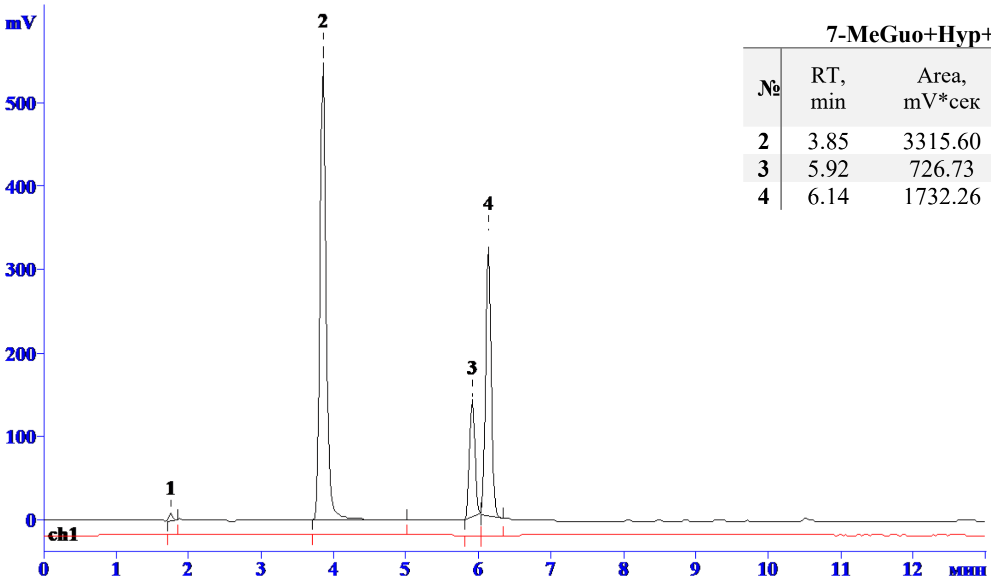

7-MeGuo+Hyp+P_i – 1.5:1:0.5– $t_{equilibrium}$

№	RT, min	Area, mV*сек	Equilibrium concentration, μM	Name
2	3.85	3315.60	137.33	7-MeGua
3	5.92	726.73	39.19	7-MeGuo
4	6.14	1732.26	200	Ino

Fugure S1.3. <u>pH = 8.5</u>

a) $t_{initial}$

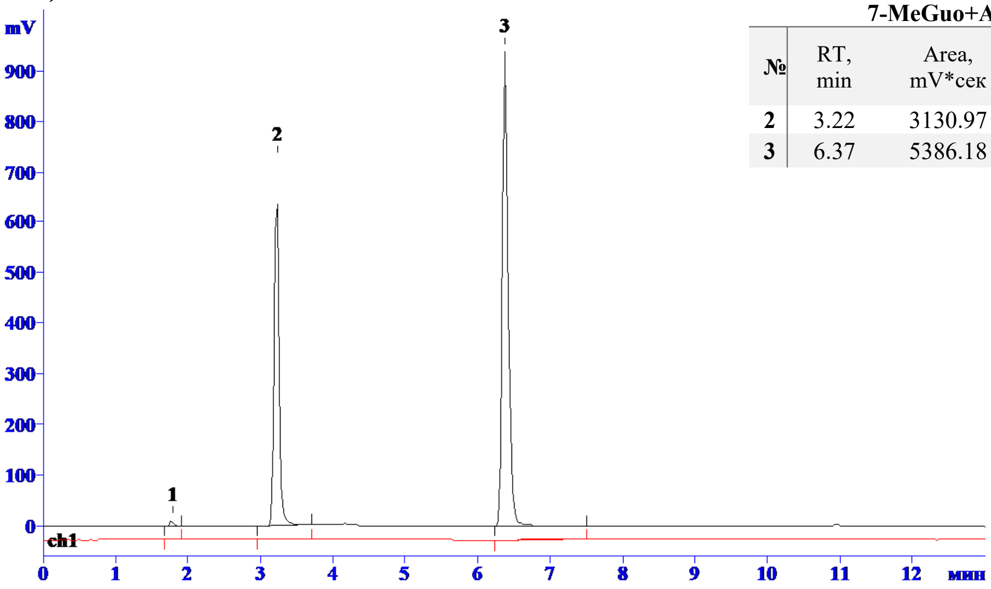

7-MeGuo+Ade+P_i –1.5:1:0.5 – t_0

№	RT, min	Area, mV*сек	Equilibrium concentration, μM	Name
2	3.22	3130.97	200.00	Ade
3	6.37	5386.18	300.00	7-MeGuo

$t_{equilibrium}$

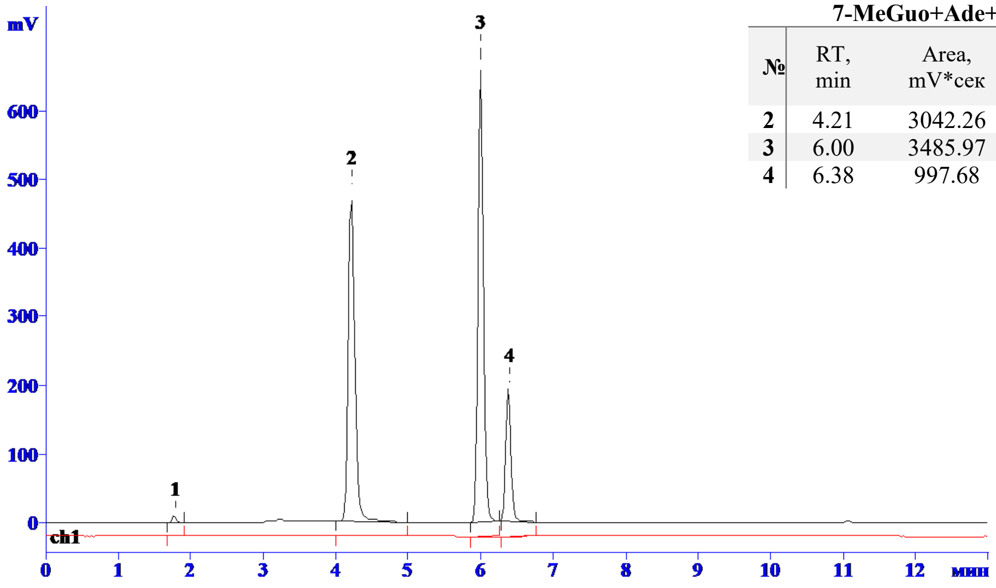

7-MeGuo+Ade+P_i – 1.5:1:0.5– t $_{equilibrium}$

№	RT, min	Area, mV*сек	Equilibrium concentration, µM	Name
2	4.21	3042.26	244.43	7-MeGua
3	6.00	3485.97	200	Ado
4	6.38	997.68	55.57	7-MeGuo

b) $t_{initial}$

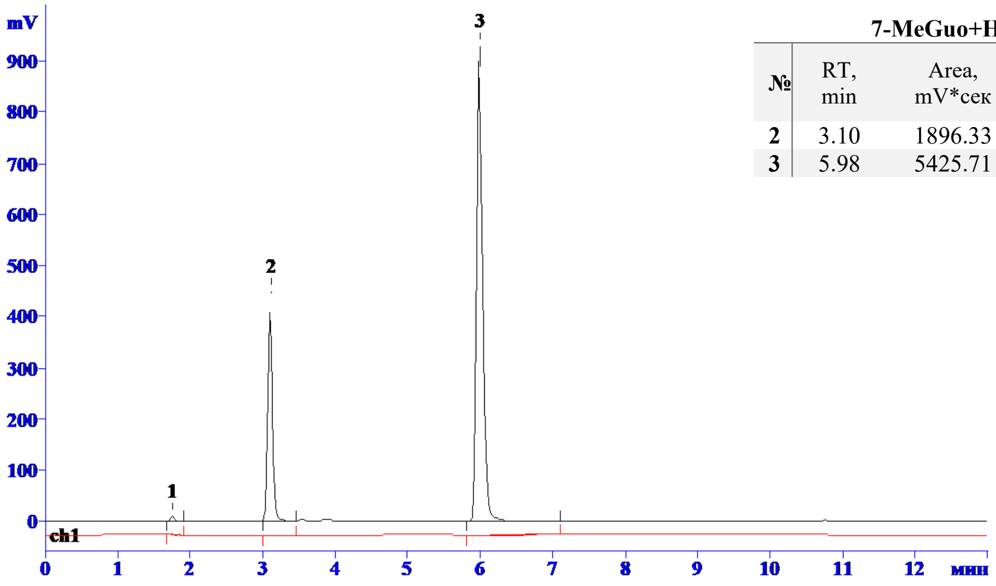

7-MeGuo+Hyp+P_i –1.5:1:0.5 – t_0

№	RT, min	Area, mV*сек	Equilibrium concentration, µM	Name
2	3.10	1896.33	200.00	Hyp
3	5.98	5425.71	300.00	7-MeGuo

$t_{equilibrium}$

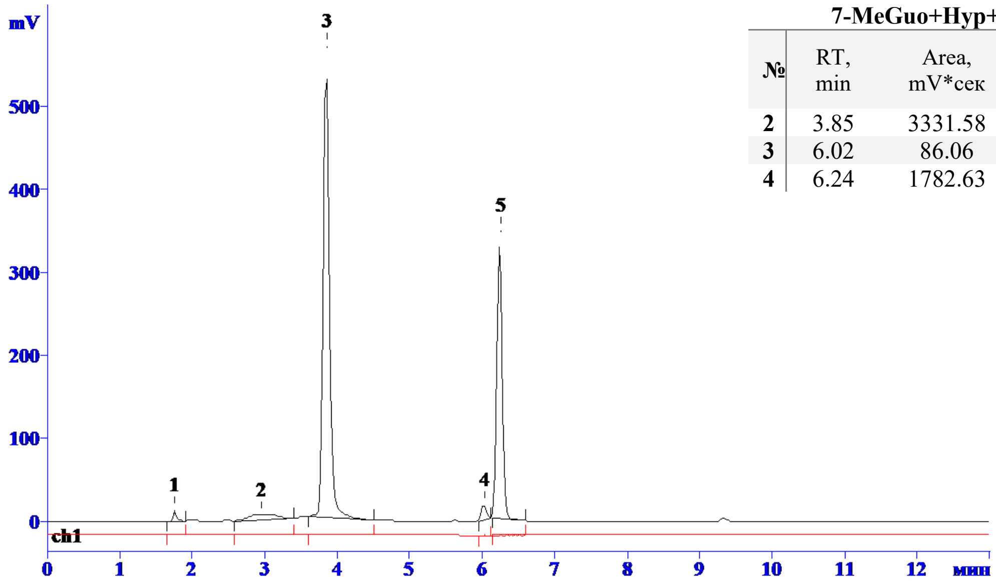

7-MeGuo+Hyp+P_i – 1.5:1:0.5– t $_{equilibrium}$

№	RT, min	Area, mV*сек	Equilibrium concentration, µM	Name
2	3.85	3331.58	295.24	7-MeGua
3	6.02	86.06	4.76	7-MeGuo
4	6.24	1782.63	200	Ino

N⁶-methyl-adenosine from 7-methylguanosine (7-MeGuo+ N⁶-Me-Ade+P_i, analytical method)

HPLC-analysis of transglycosylation: Phenomenex Hydro-RP, 150 × 4.6 мм, 80 Å, 4 мкм, 20ºC, elution in modified mobile phase: linear gradient of acetonitrile in 0.06% TFA/deionized water from 2 to 12% for 10 min (flushing with 12-80 % acetonitrile-0.06% TFA/deionized water for 10-10.1 min, then 80-2% for 10.1-10.8 min) at flow rate 1 ml/min with UV detection at wavelength 270 nm, injection volume 20 µL.

Fugure S2.1. pH = 5.5

$t_{initial}$

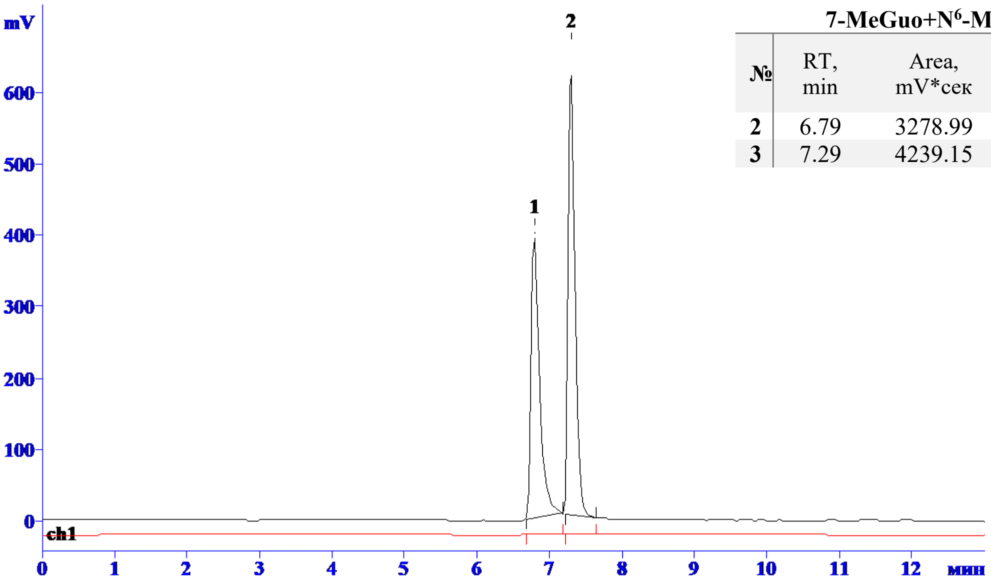

7-MeGuo+N⁶-Me-Ade+P_i –1.5:1:0.5 – t_0

№	RT, min	Area, mV*сек	Equilibrium concentration, µM	Name
2	6.79	3278.99	200.00	N⁶-Me-Ade
3	7.29	4239.15	300.00	7-MeGuo

$t_{equilibrium}$

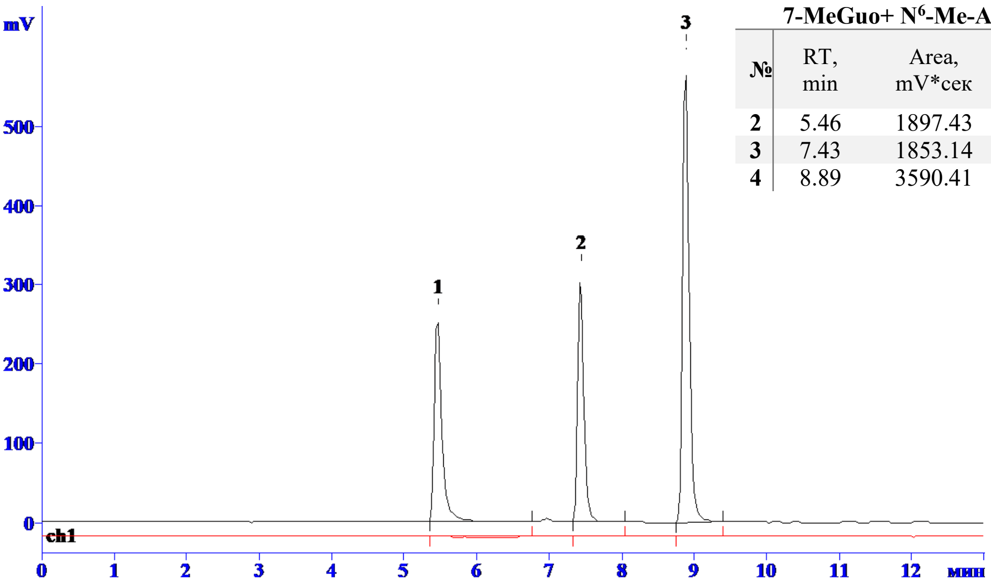

7-MeGuo+ N⁶-Me-Ade+P_i – 1.5:1:0.5– $t_{equilibrium}$

№	RT, min	Area, mV*сек	Equilibrium concentration, µM	Name
2	5.46	1897.43	168.86	7-MeGua
3	7.43	1853.14	131.14	7-MeGuo
4	8.89	3590.41	200	N⁶-Me-Ado

Fugure S2.2. pH = 7.0

$t_{initial}$

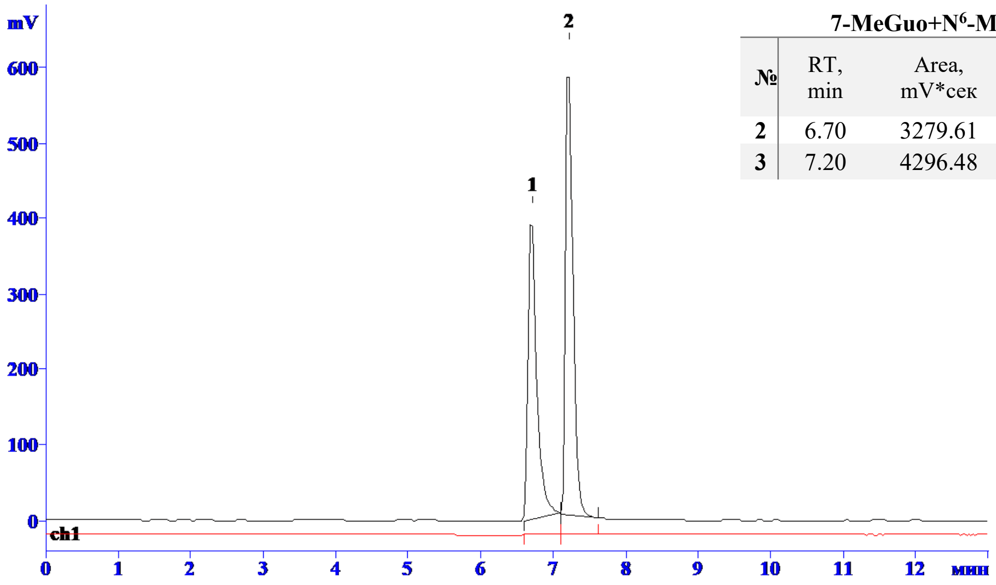

7-MeGuo+N^6-Me-Ade+P_i –1.5:1:0.5 – t_0

№	RT, min	Area, mV*сек	Equilibrium concentration, μM	Name
**2**	6.70	3279.61	200.00	N^6-Me-Ade
**3**	7.20	4296.48	300.00	7-MeGuo

$t_{equilibrium}$

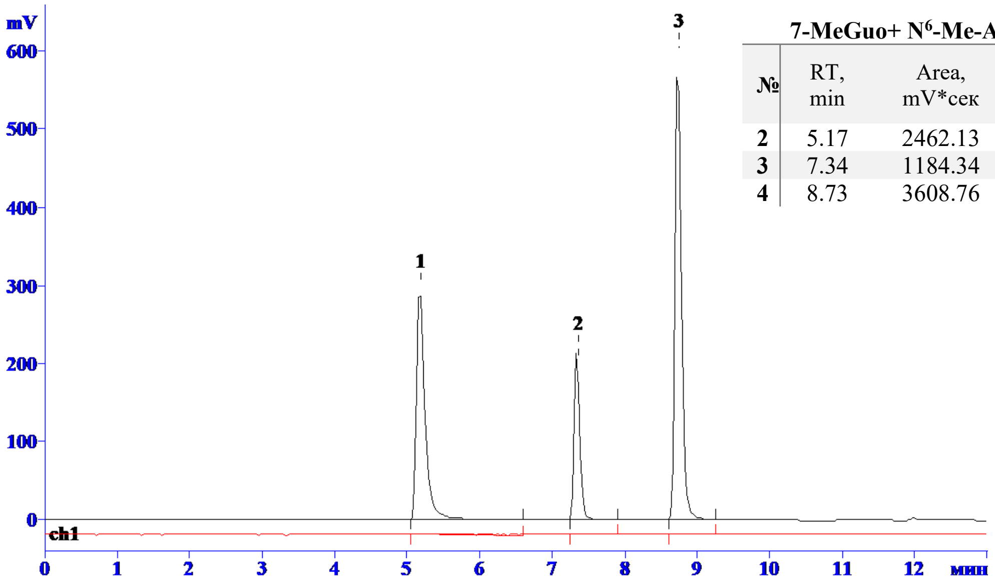

7-MeGuo+ N^6-Me-Ade+P_i – 1.5:1:0.5– t $_{equilibrium}$

№	RT, min	Area, mV*сек	Equilibrium concentration, μM	Name
**2**	5.17	2462.13	217.32	7-MeGua
**3**	7.34	1184.34	82.68	7-MeGuo
**4**	8.73	3608.76	200	N^6-Me-Ado

Fugure S2.3. pH = 8.5

$t_{initial}$

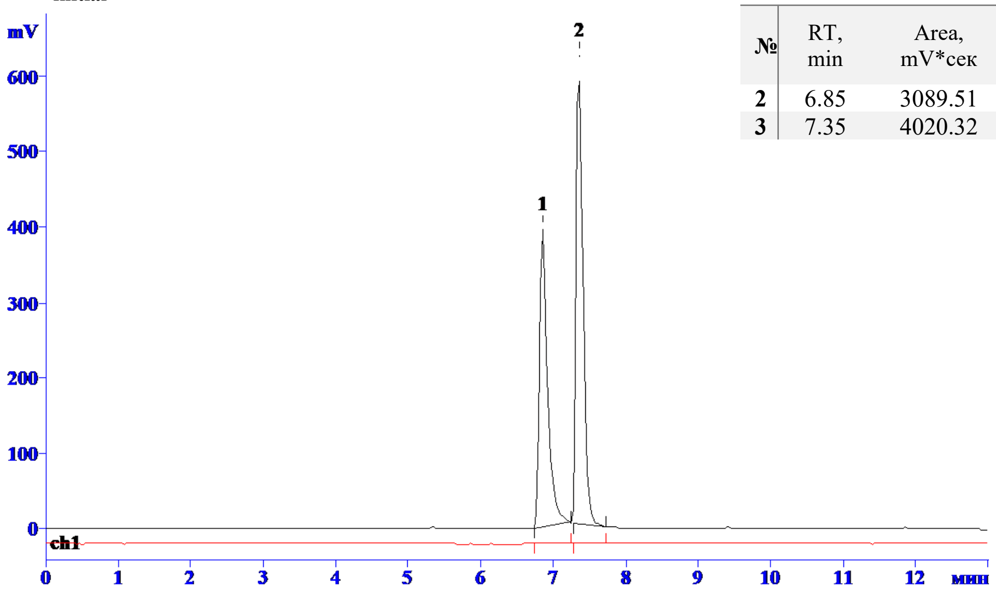

7-MeGuo+N^6-Me-Ade+P_i –1.5:1:0.5 – t_0

№	RT, min	Area, mV*сек	Equilibrium concentration, μM	Name
**2**	6.85	3089.51	200.00	N^6-Me-Ade
**3**	7.35	4020.32	300.00	7-MeGuo

$t_{equilibrium}$

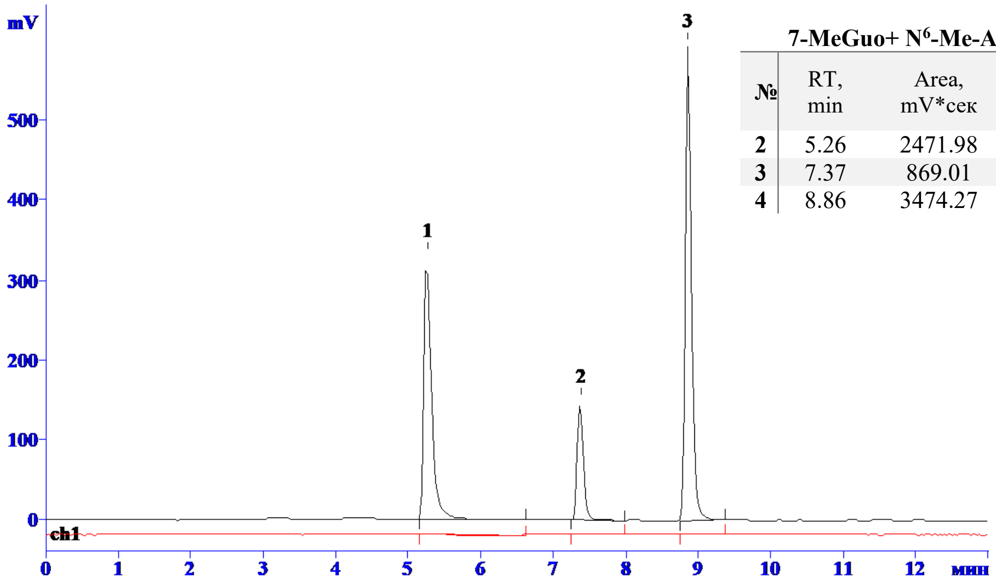

7-MeGuo+ N6-Me-Ade+P_i – 1.5:1:0.5– t $_{equilibrium}$

№	RT, min	Area, mV*сек	Equilibrium concentration, µM	Name
2	5.26	2471.98	235.15	7-MeGua
3	7.37	869.01	64.85	7-MeGuo
4	8.86	3474.27	200	N6-Me-Ado

N6-isopropyl-adenosine from 7-methylguanosine (7-MeGuo+ N6-iPr-Ade+P_i, analytical method)

HPLC-analysis of transglycosylation Luna LC-Column C18(2), 4.6 × 150 мм, 5 мкм, 20ºC, elution in modified mobile phase: linear gradient of acetonitrile in 0.06% TFA/deionized water from 2 to 30% for 15 min (flushing with 12-80 % acetonitrile-0.06% TFA/deionized water for 10-10.1 min, then 80-2% for 10.1-10.8 min) at flow rate 1 ml/min with UV detection at wavelength 260 nm, injection volume 20 µL.

Fugure S3.1. pH = 5.5

$t_{initial}$

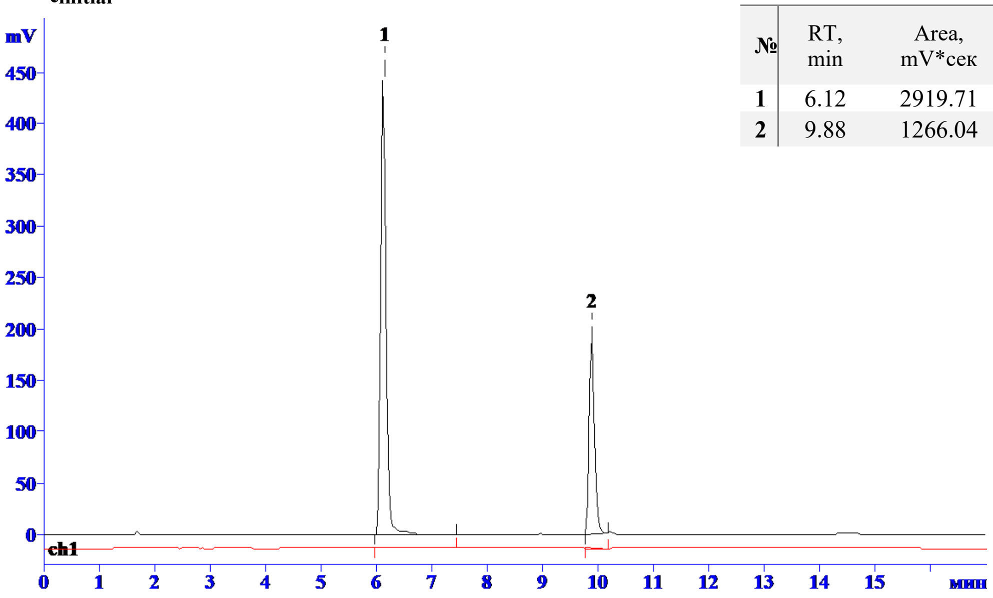

7-MeGuo+N6-iPr-Ade+P_i –1.5:1:0.5 – t_0

№	RT, min	Area, mV*сек	Equilibrium concentration, µM	Name
1	6.12	2919.71	300.00	7-MeGuo
2	9.88	1266.04	200.00	N6-iPR-Ade

tequilibrium

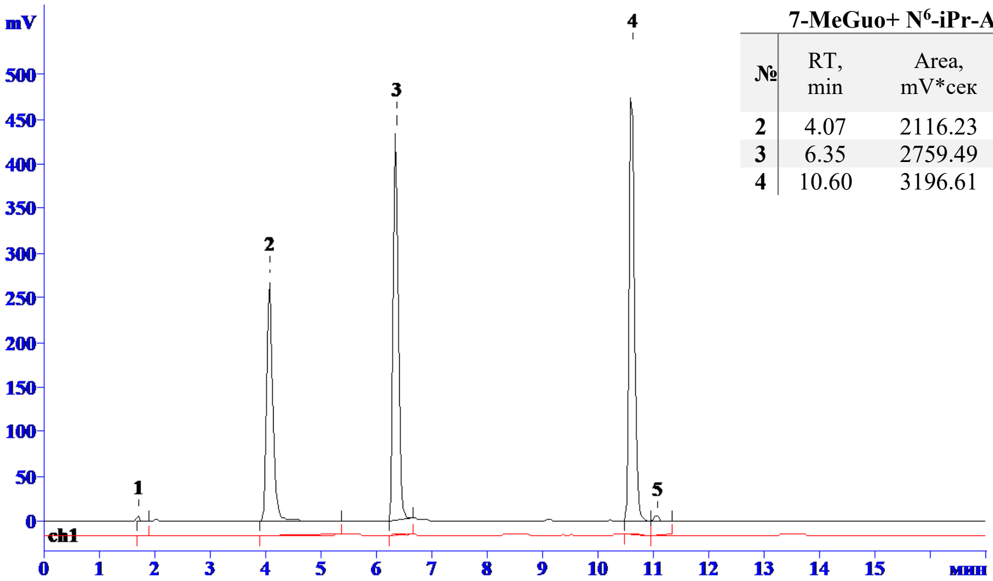

7-MeGuo+ N⁶-iPr-Ade+Pᵢ – 1.5:1:0.5– t equilibrium

№	RT, min	Area, mV*сек	Equilibrium concentration, µM	Name
2	4.07	2116.23	49.14	7-MeGua
3	6.35	2759.49	250.86	7-MeGuo
4	10.60	3196.61	200	N⁶-iPR-Ado

Fugure S3.2. pH = 7.0

tinitial

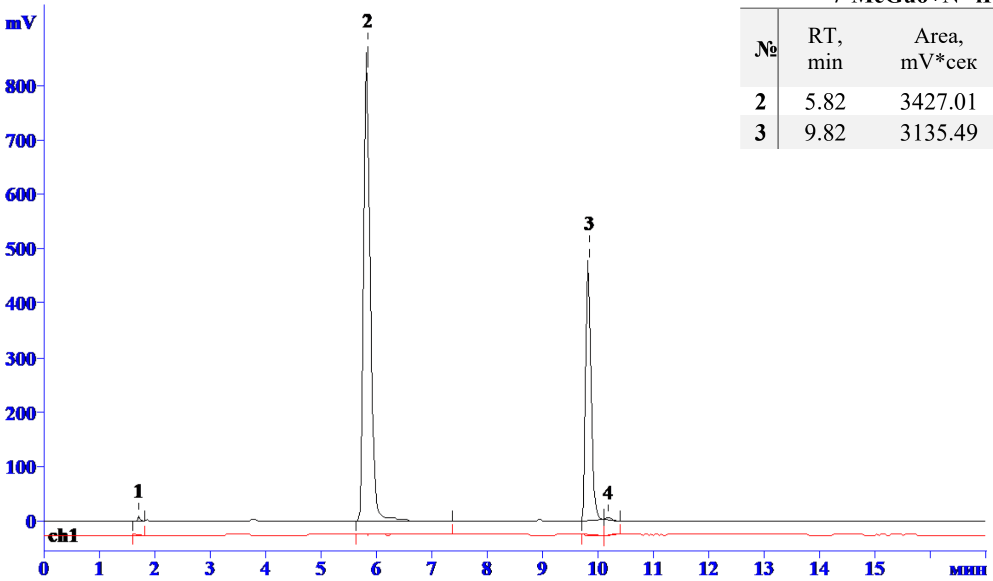

7-MeGuo+N⁶-iPr-Ade+Pᵢ –1.5:1:0.5 – t₀

№	RT, min	Area, mV*сек	Equilibrium concentration, µM	Name
2	5.82	3427.01	300.00	7-MeGuo
3	9.82	3135.49	200.00	N⁶-iPR-Ade

tequilibrium

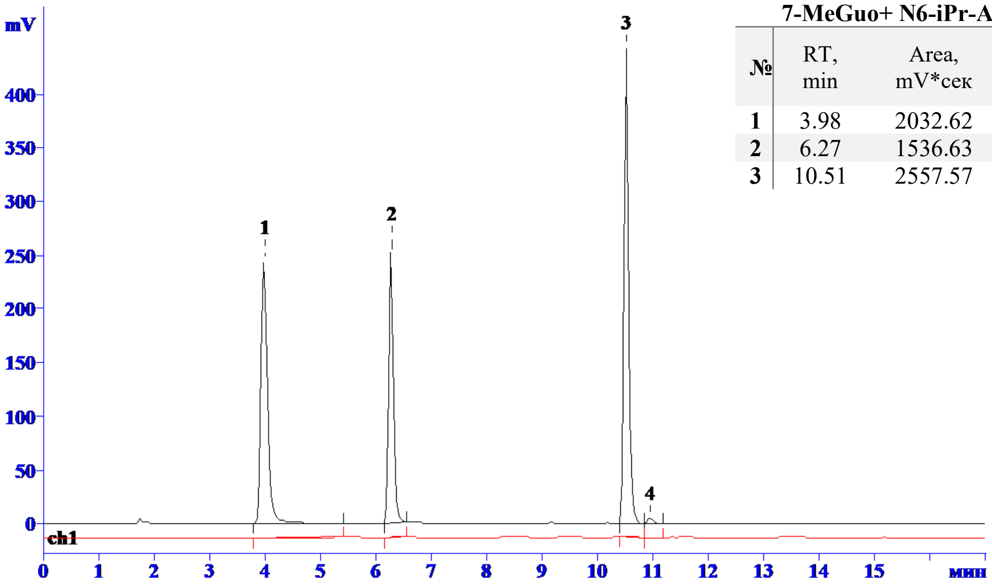

7-MeGuo+ N6-iPr-Ade+Pᵢ – 1.5:1:0.5– t equilibrium

№	RT, min	Area, mV*сек	Equilibrium concentration, µM	Name
1	3.98	2032.62	165.48	7-MeGua
2	6.27	1536.63	134.52	7-MeGuo
3	10.51	2557.57	200	N⁶-iPR-Ado

Fugure S3.3. <u>pH = 8.5</u>

$t_{initial}$

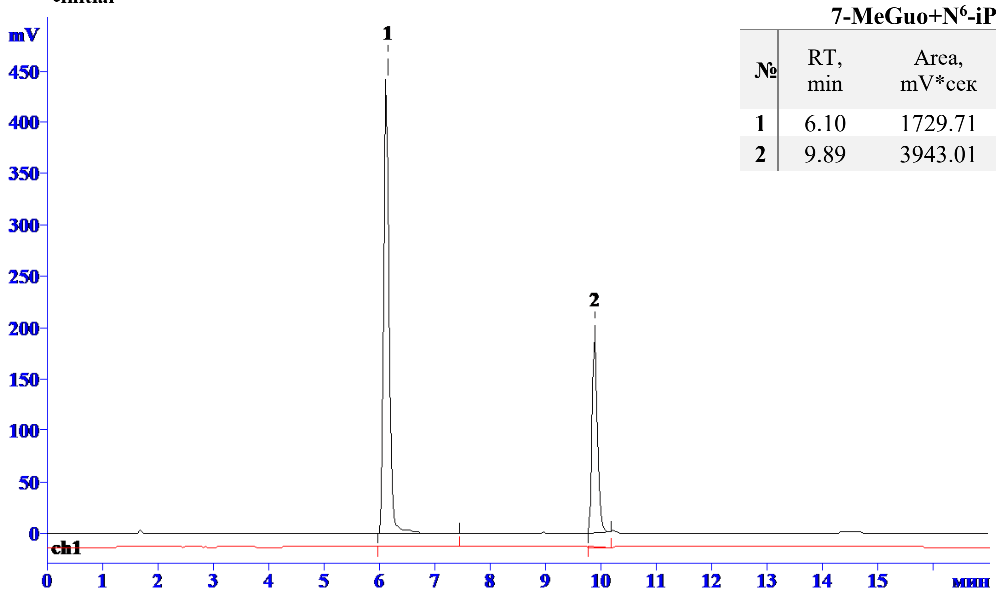

7-MeGuo+N⁶-iPr-Ade+P$_i$ –1.5:1:0.5 – t$_0$

№	RT, min	Area, mV*сек	Equilibrium concentration, μM	Name
1	6.10	1729.71	300.00	7-MeGuo
2	9.89	3943.01	200.00	N^6-iPR-Ade

$t_{equilibrium}$

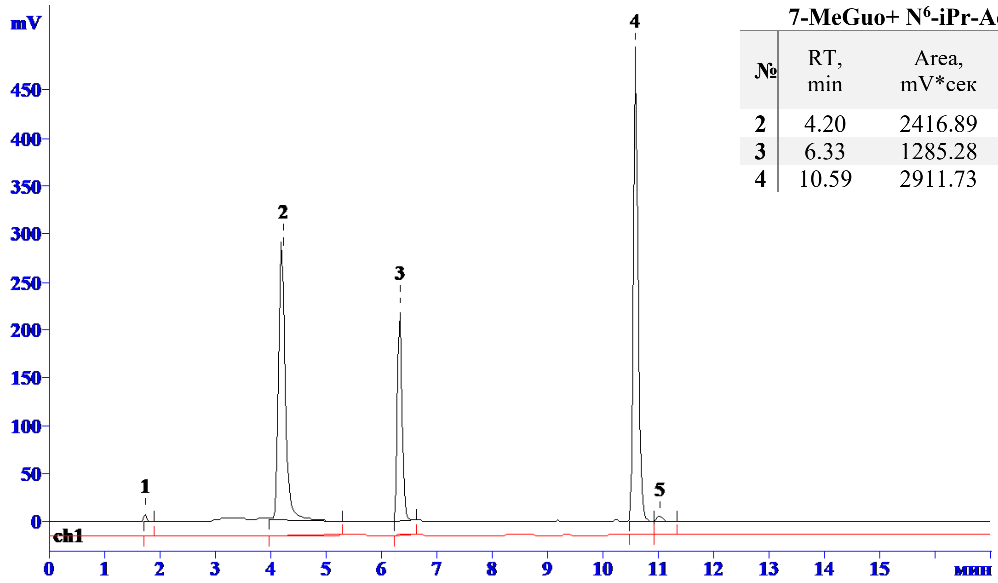

7-MeGuo+ N^6-iPr-Ade+P$_i$ – 1.5:1:0.5– t $_{equilibrium}$

№	RT, min	Area, mV*сек	Equilibrium concentration, μM	Name
2	4.20	2416.89	202.21	7-MeGua
3	6.33	1285.28	97.79	7-MeGuo
4	10.59	2911.73	200	N^6-iPR-Ado

<u>N^6-benzyl-adenosine from 7-methylguanosine (7-MeGuo+ N^6-Bn-Ade+P$_i$, analytical method)</u>

HPLC-analysis of transglycosylation Luna LC-Column C18(2), 4.6 × 150 мм, 5 мкм, 20ºC, elution in modified mobile phase: linear gradient of acetonitrile in 0.06% TFA/deionized water from 2 to 24 to 42 to 50% for 7-15 min (flushing with 12-80 % acetonitrile-0.06% TFA/deionized water for 10-10.1 min, then 80-2% for 10.1-10.8 min) at flow rate 1 ml/min with UV detection at wavelength 269 nm, injection volume 20 μL.

Fugure S4.1. pH = 5.5

$t_{initial}$

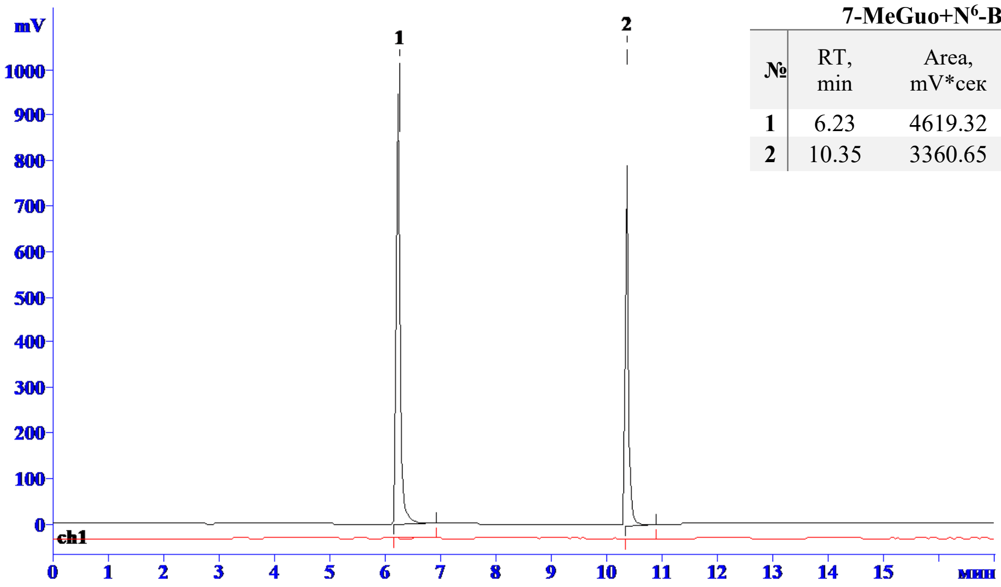

7-MeGuo+N^6-Bn-Ade+P$_i$ –1.5:1:0.5 – t_0

№	RT, min	Area, mV*сек	Equilibrium concentration, µM	Name
1	6.23	4619.32	300.00	7-MeGuo
2	10.35	3360.65	200.00	N^6-Bn-Ade

$t_{equilibrium}$

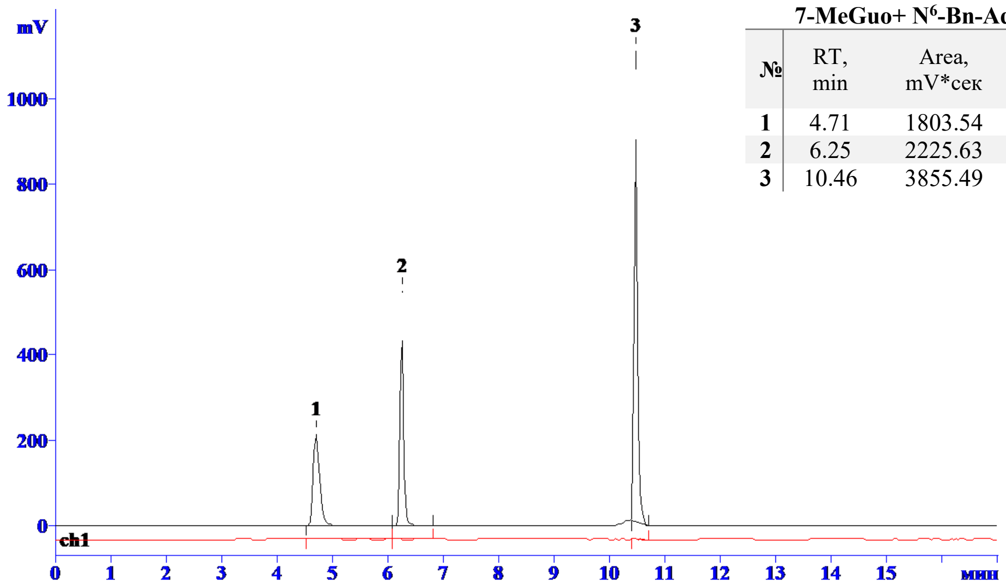

7-MeGuo+ N^6-Bn-Ade+P$_i$ – 1.5:1:0.5– $t_{equilibrium}$

№	RT, min	Area, mV*сек	Equilibrium concentration, µM	Name
1	4.71	1803.54	155.46	7-MeGua
2	6.25	2225.63	144.54	7-MeGuo
3	10.46	3855.49	200	N^6-Bn-Ado

Fugure S4.2. pH = 7.0

$t_{initial}$

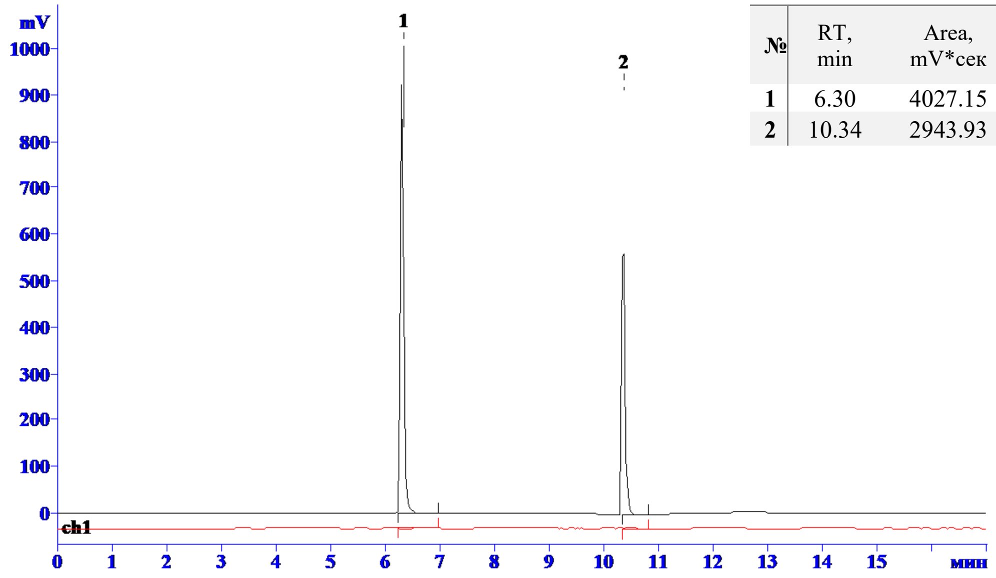

7-MeGuo+N^6-Bn-Ade+P$_i$ –1.5:1:0.5 – t_0

№	RT, min	Area, mV*сек	Equilibrium concentration, µM	Name
1	6.30	4027.15	300.00	7-MeGuo
2	10.34	2943.93	200.00	N^6-Bn-Ade

$t_{equilibrium}$

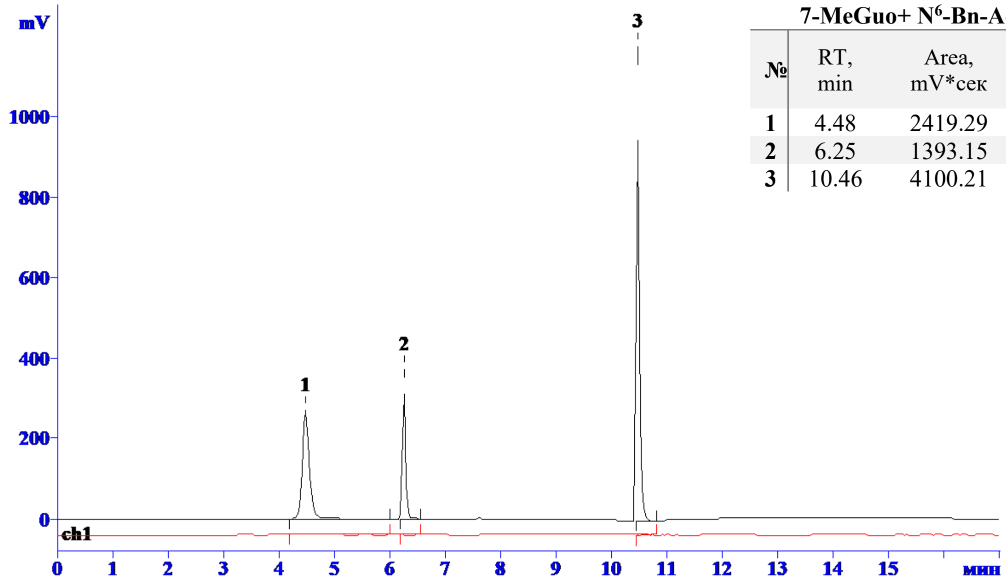

7-MeGuo+ N⁶-Bn-Ade+Pᵢ – 1.5:1:0.5– t equilibrium

№	RT, min	Area, mV*сек	Equilibrium concentration, μM	Name
1	4.48	2419.29	158.03	7-MeGua
2	6.25	1393.15	141.97	7-MeGuo
3	10.46	4100.21	200	N^6-Bn-Ado

Fugure S4.3. pH = 8.5

$t_{initial}$

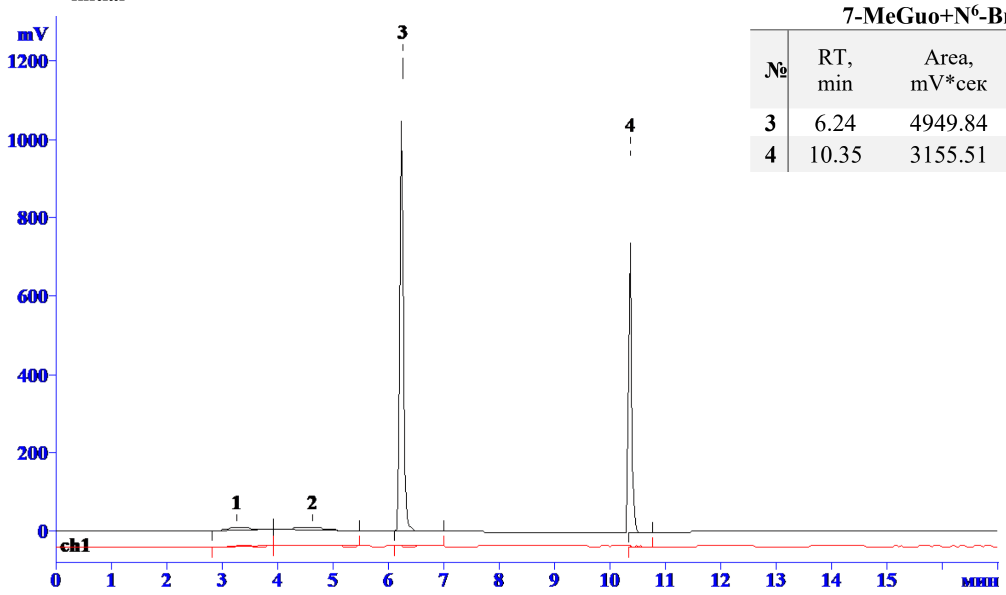

7-MeGuo+N^6-Bn-Ade+P$_i$ –1.5:1:0.5 – t$_0$

№	RT, min	Area, mV*сек	Equilibrium concentration, μM	Name
3	6.24	4949.84	300.00	7-MeGuo
4	10.35	3155.51	200.00	N^6-Bn-Ade

$t_{equilibrium}$

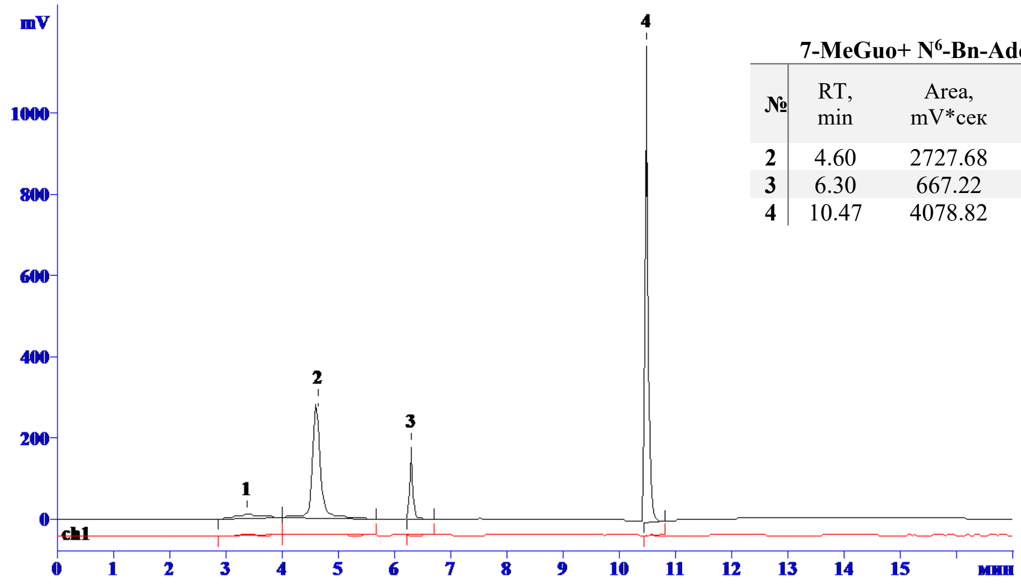

7-MeGuo+ N^6-Bn-Ade+P$_i$ – 1.5:1:0.5– t equilibrium

№	RT, min	Area, mV*сек	Equilibrium concentration, μM	Name
2	4.60	2727.68	259.56	7-MeGua
3	6.30	667.22	40.44	7-MeGuo
4	10.47	4078.82	200	N^6-Bn-Ado

O^6-benzyl-inosin from 7-methylguanosine (7-MeGuo+ O^6-Bn-Hyp+P$_i$, analytical method)

HPLC-analysis of transglycosylation: Synergy Hydro-RP, 150 × 4.6 мм, 80 Ǻ, H19, 4 мкм, 20ºC, elution in modified mobile phase: linear gradient of acetonitrile in 0.06% TFA/deionized water from 2 to 24 to 42 to 50% for 7-15 min (flushing with 12-80 % acetonitrile-0.06% TFA/deionized water for 10-10.1 min, then 80-2% for 10.1-10.8 min) at flow rate 1 ml/min with UV detection at wavelength 260 nm, injection volume 20 µL.

Fugure S5.1. pH = 5.5

$t_{initial}$

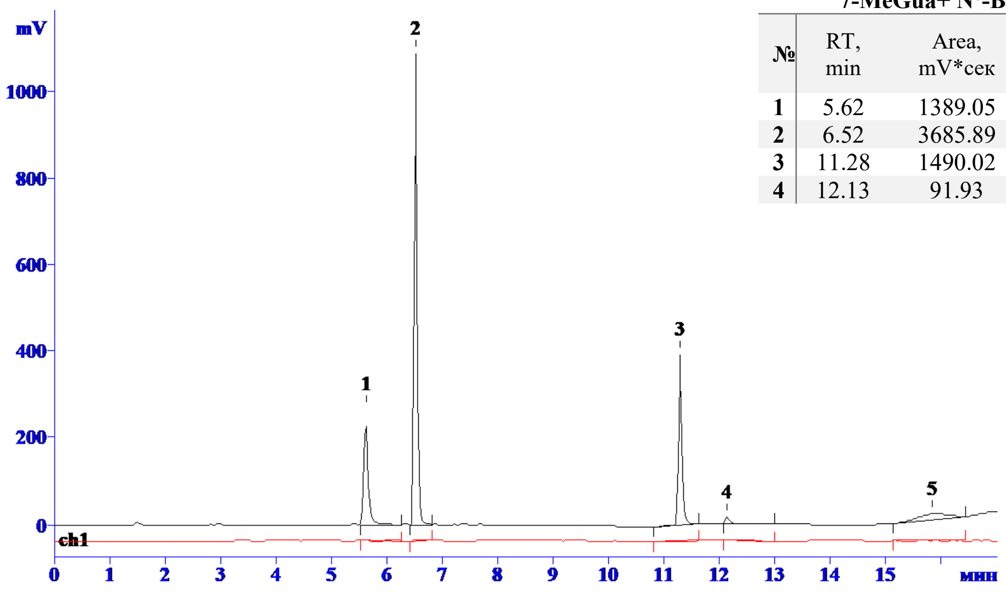

7-MeGua+ N^6-Bn-Hyp +P$_i$ – 1.5:1:0.5– t$_0$

№	RT, min	Area, mV*сек	Equilibrium concentration, µM	Name
1	5.62	1389.05	-	7-MeGua
2	6.52	3685.89	-	7-MeGuo
3	11.28	1490.02	-	N^6-Bn-Ino
4	12.13	91.93	-	N^6-Bn-Hyp

$t_{equilibrium}$

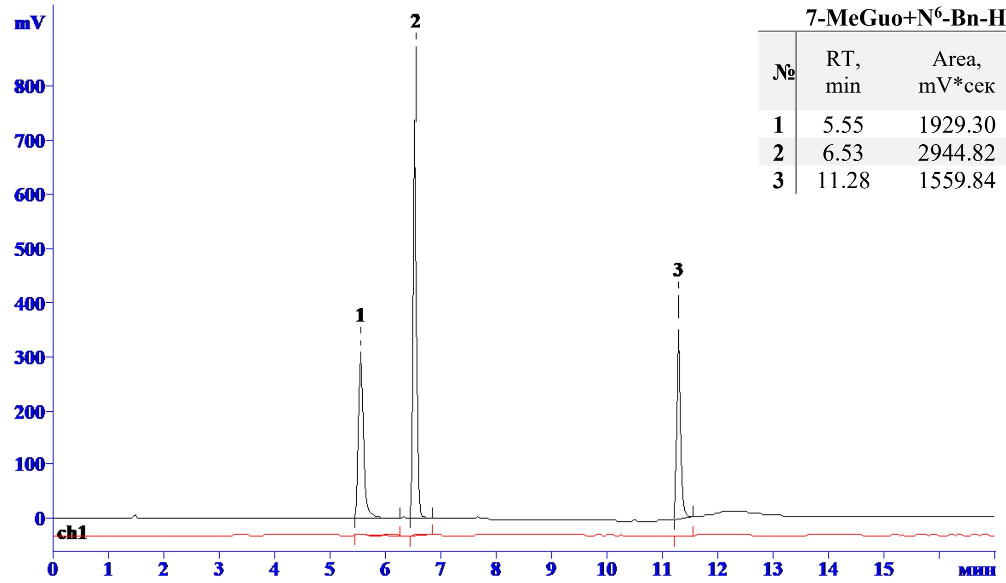

7-MeGuo+N^6-Bn-Hyp+P$_i$ – 1.5:1:0.5– t $_{equilibrium}$

№	RT, min	Area, mV*сек	Equilibrium concentration, µM	Name
1	5.55	1929.30	-	7-MeGua
2	6.53	2944.82	-	7-MeGuo
3	11.28	1559.84	-	N^6-Bn-Ino

Fugure S5.2. pH = 7.0

$t_{initial}$

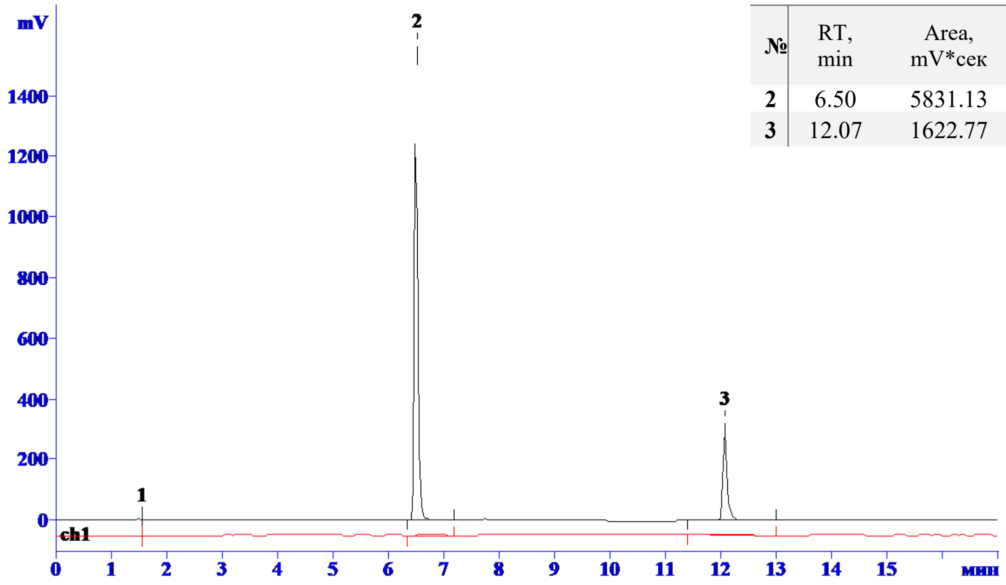

7-MeGuo+ N⁶-Bn-Hyp+Pᵢ –1.5:1:0.5 – t₀

№	RT, min	Area, mV*сек	Equilibrium concentration, µM	Name
2	6.50	5831.13	300.00	7-MeGuo
3	12.07	1622.77	200.00	N^6-Bn-Hyp

$t_{equilibrium}$

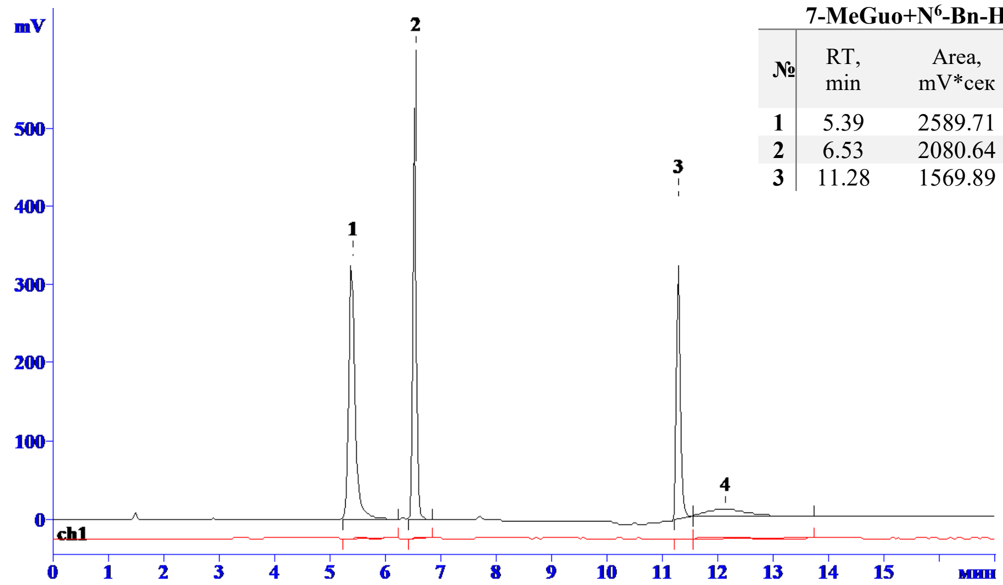

7-MeGuo+N⁶-Bn-Hyp+Pᵢ – 1.5:1:0.5– t equilibrium

№	RT, min	Area, mV*сек	Equilibrium concentration, µM	Name
1	5.39	2589.71	192.96	7-MeGua
2	6.53	2080.64	107.04	7-MeGuo
3	11.28	1569.89	200	N^6-Bn-Ino

Fugure S5.3. pH = 8.5

$t_{initial}$

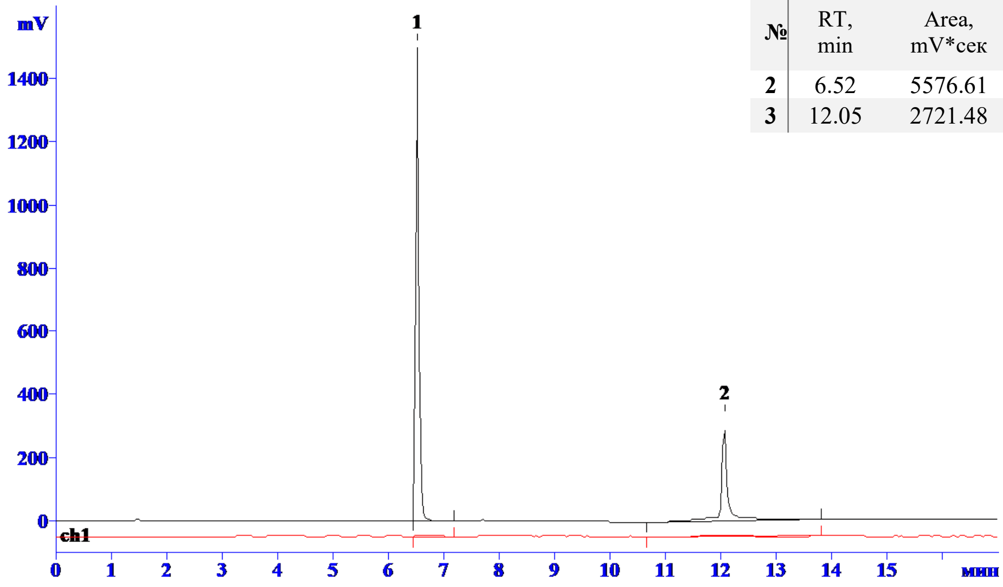

7-MeGuo+ N⁶-Bn-Hyp+Pᵢ –1.5:1:0.5 – t₀

№	RT, min	Area, mV*сек	Equilibrium concentration, µM	Name
2	6.52	5576.61	300.00	7-MeGuo
3	12.05	2721.48	200.00	N^6-Bn-Hyp

$t_{equilibrium}$

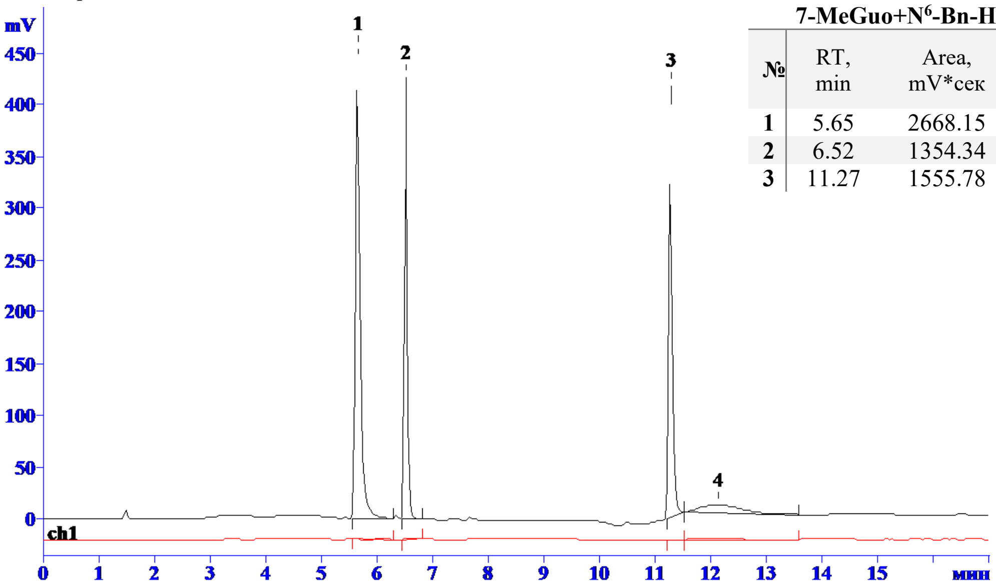

7-MeGuo+N⁶-Bn-Hyp+P_i – 1.5:1:0.5– t $_{equilibrium}$

№	RT, min	Area, mV*сек	Equilibrium concentration, μM	Name
1	5.65	2668.15	227.14	7-MeGua
2	6.52	1354.34	72.86	7-MeGuo
3	11.27	1555.78	200	N^6-Bn-Ino

6-thi-purinriboside, S^6-methyl-purinriboside, S^6-benzyl-purinriboside, O^6- methyl-inosin from 7-methylguanosine (7-MeGuo+SH-Pur, S^6-Me-Pur, S^6-Bn-Pur, O^6-Me-Hyp+P_i, analytical method)

HPLC-analysis of transglycosylation: Cosmosil 5C$_{18}$-MS-II, 4.6*150 mm, 5μm, 120 Å, (Nacalai Tesque, Inc. (Japan)), 20°C, elution in modified mobile phase: linear gradient of acetonitrile in 0.06% TFA/deionized water from from 2 to 24 to 42 to 50% for 7-15 min (flushing with 12-80 % acetonitrile-0.06% TFA/deionized water for 10-10.1 min, then 80-2% for 10.1-10.8 min) at flow rate 1 ml/min with UV detection at wavelength: SH-Pur – 323 nm, S^6-Me-Pur – 289 nm, S^6-Bn-Pur – 291 nm, O^6-Me-Hyp – 260 nm, injection volume 20 μL.

Fugure S6.1. **pH = 5.5**

a) $t_{initial}$

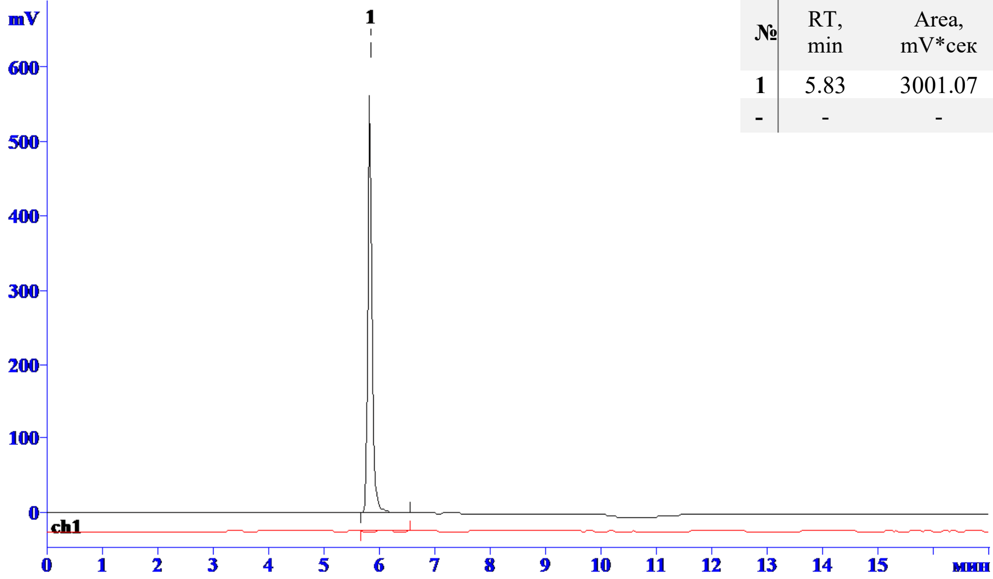

7-MeGuo+SH-Pur+P_i –1.5:1:0.5 – t_0

№	RT, min	Area, mV*сек	Equilibrium concentration, μM	Name
1	5.83	3001.07	200.00	SH-Pur
-	-	-	300.00	7-MeGuo

$t_{equilibrium}$

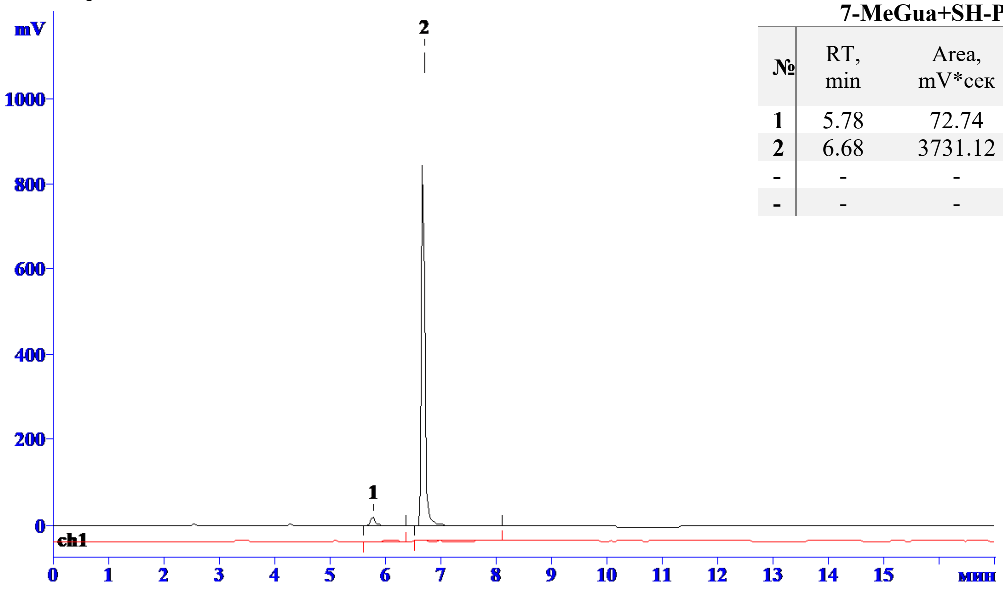

7-MeGua+SH-Pur – 1.5:1:0.5– t equilibrium

№	RT, min	Area, mV*сек	Equilibrium concentration, µM	Name
1	5.78	72.74	4.85	SH-Pur
2	6.68	3731.12	195.15	SH-PurRib
-	-	-	-	7-MeGua
-	-	-	-	7-MeGuo

b) $t_{initial}$

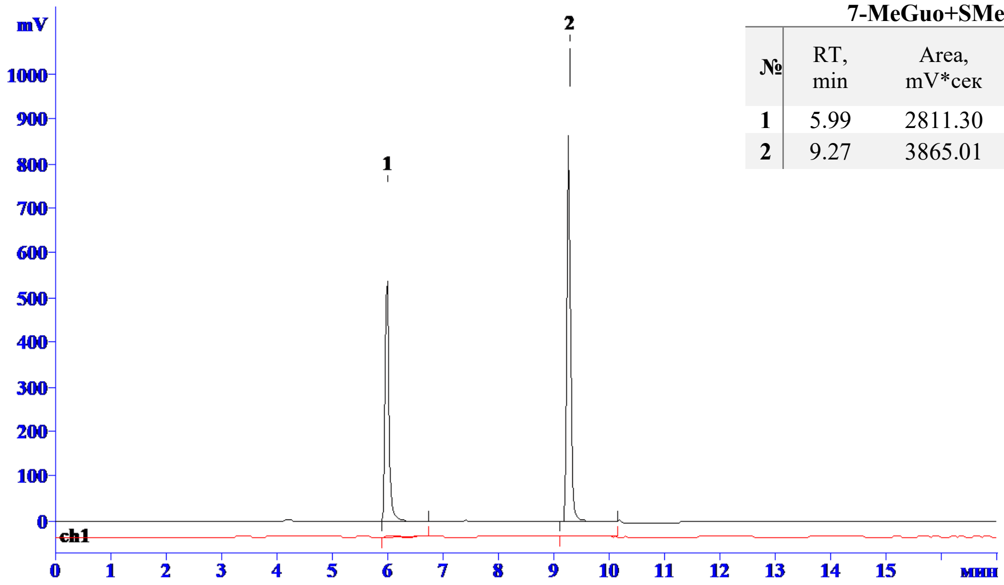

7-MeGuo+SMe-Pur+P_i –1.5:1:0.5 – t_0

№	RT, min	Area, mV*сек	Equilibrium concentration, µM	Name
1	5.99	2811.30	300.00	7-MeGuo
2	9.27	3865.01	200.00	SMe-Pur

$t_{equilibrium}$

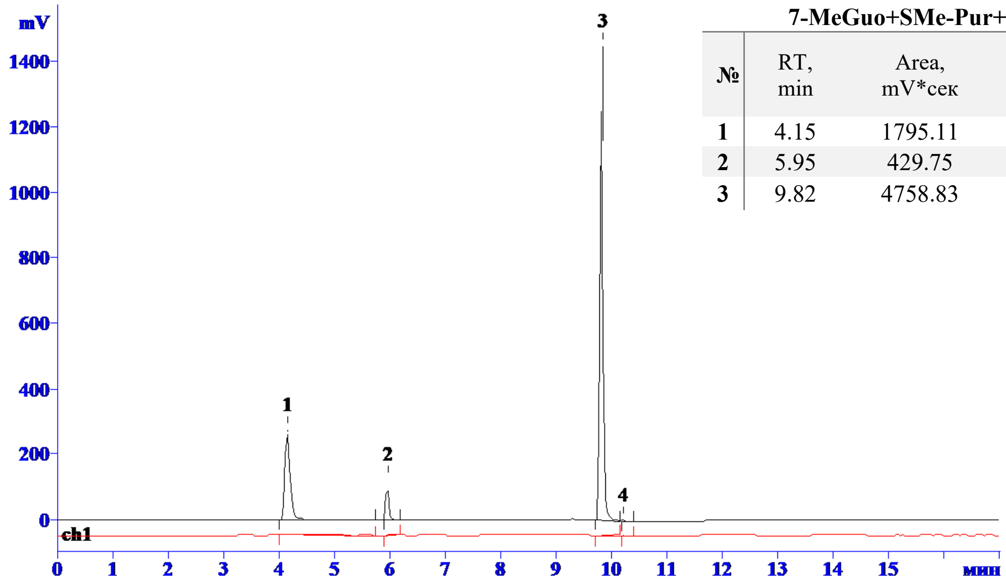

7-MeGuo+SMe-Pur+P_i – 1.5:1:0.5– t equilibrium

№	RT, min	Area, mV*сек	Equilibrium concentration, µM	Name
1	4.15	1795.11	254.14	7-MeGua
2	5.95	429.75	45.86	7-MeGuo
3	9.82	4758.83	200	SMe-PurRib

c) $t_{initial}$

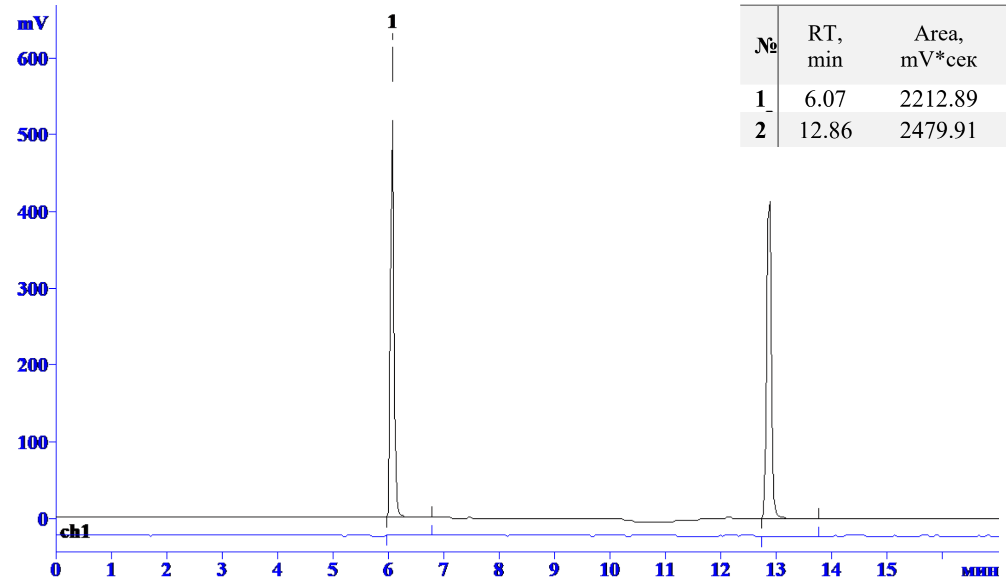

7-MeGuo+SBn-Pur+P_i –1.5:1:0.5 – t_0

№	RT, min	Area, mV*сек	Equilibrium concentration, μM	Name
1	6.07	2212.89	300.00	7-MeGuo
2	12.86	2479.91	200.00	SBn-Pur

$t_{equilibrium}$

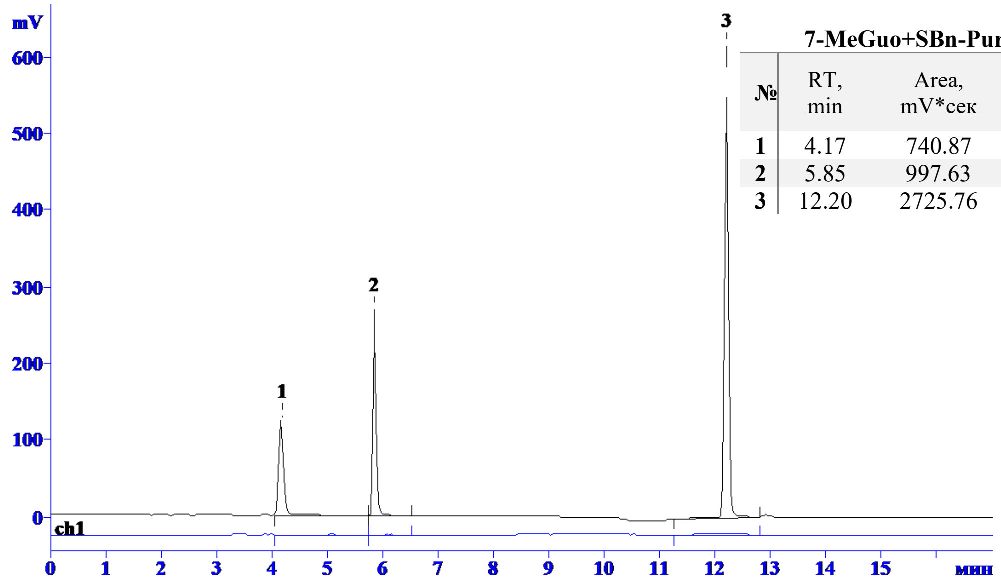

7-MeGuo+SBn-Pur+P_i – 1.5:1:0.5– $t_{equilibrium}$

№	RT, min	Area, mV*сек	Equilibrium concentration, μM	Name
1	4.17	740.87	164.75	7-MeGua
2	5.85	997.63	135.25	7-MeGuo
3	12.20	2725.76	200	SBn-PurRib

d) $t_{initial}$

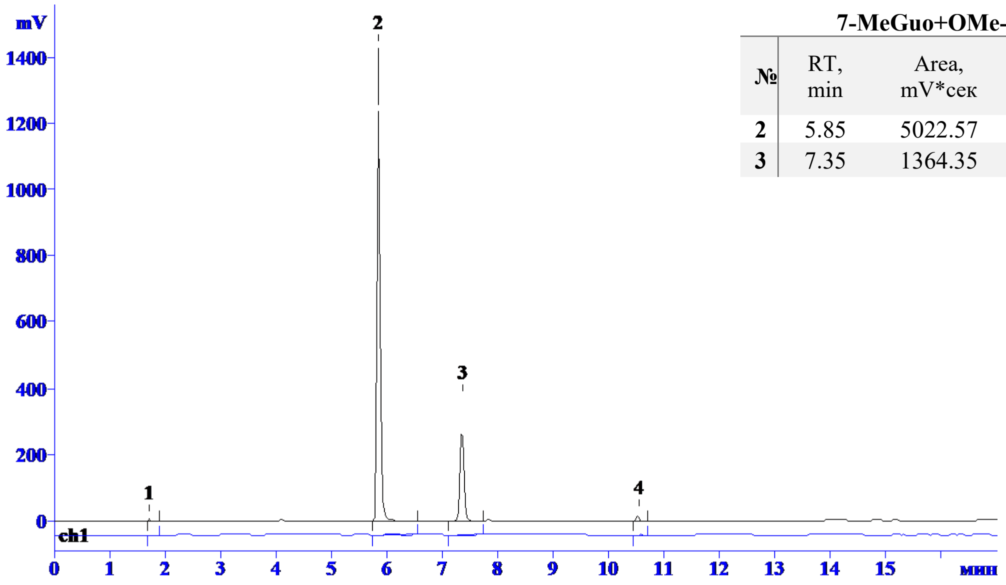

7-MeGuo+OMe-Hyp+P_i –1.5:1:0.5 – t_0

№	RT, min	Area, mV*сек	Equilibrium concentration, μM	Name
2	5.85	5022.57	300.00	7-MeGuo
3	7.35	1364.35	200.00	OMe-Hyp

$t_{equilibrium}$

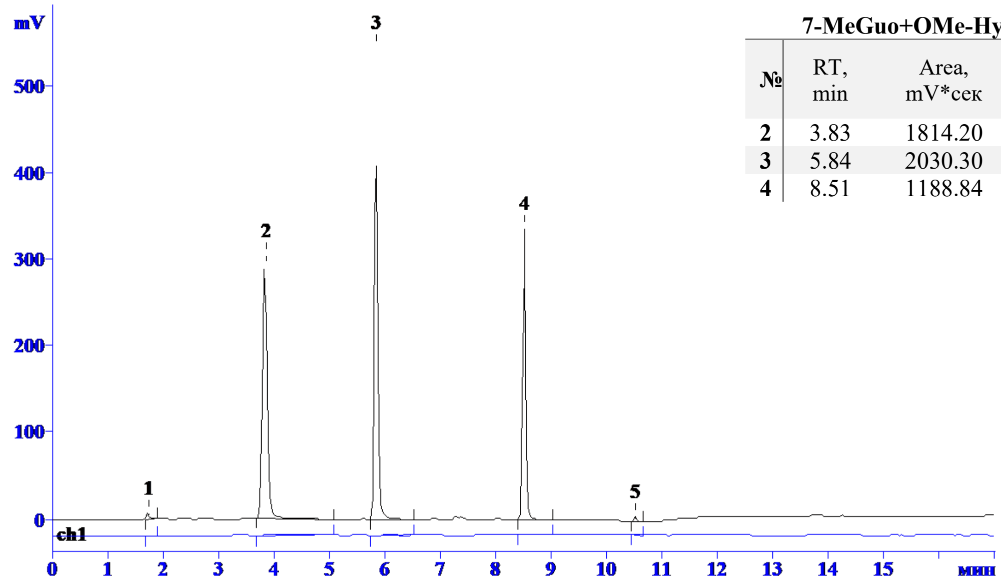

7-MeGuo+OMe-Hyp+P_i – 1.5:1:0.5– $t_{\text{equilibrium}}$

№	RT, min	Area, mV*сек	Equilibrium concentration, µM	Name
2	3.83	1814.20	178.83	7-MeGua
3	5.84	2030.30	121.27	7-MeGuo
4	8.51	1188.84	200	OMe-Ino

Fugure S6.2. pH = 7.0

a) $t_{initial}$

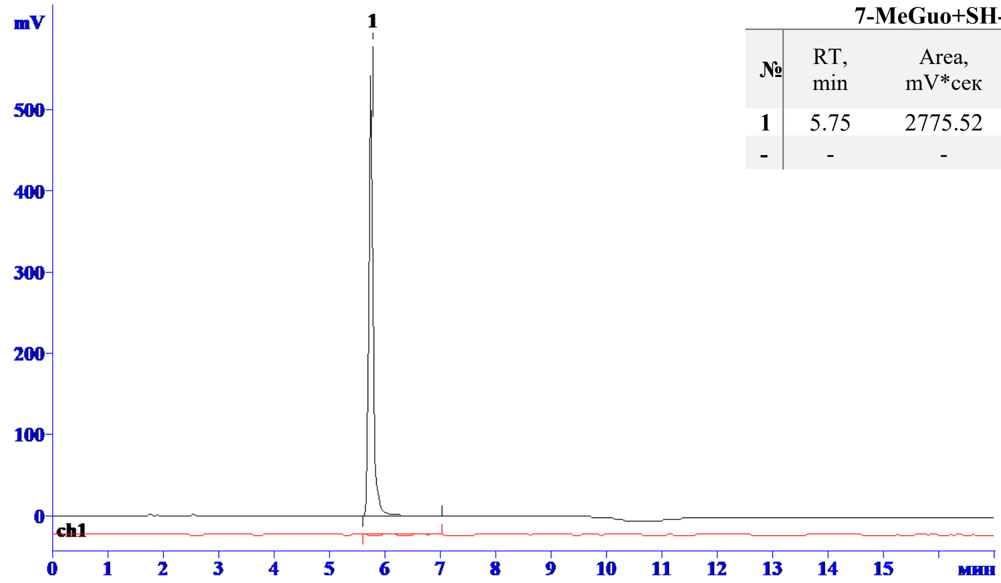

7-MeGuo+SH-Pur+P_i –1.5:1:0.5 – t_0

№	RT, min	Area, mV*сек	Equilibrium concentration, µM	Name
1	5.75	2775.52	200.00	SH-Pur
-	-	-	300.00	7-MeGuo

$t_{equilibrium}$

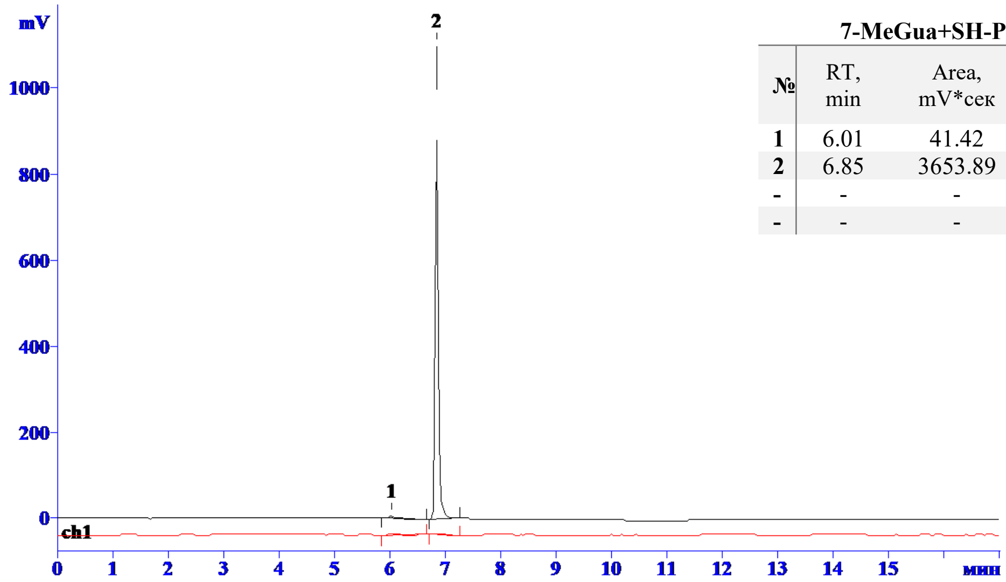

7-MeGua+SH-Pur – 1.5:1:0.5– $t_{\text{equilibrium}}$

№	RT, min	Area, mV*сек	Equilibrium concentration, µM	Name
1	6.01	41.42	2.98	SH-Pur
2	6.85	3653.89	197.02	SH-PurRib
-	-	-	-	7-MeGua
-	-	-	-	7-MeGuo

b) $t_{initial}$

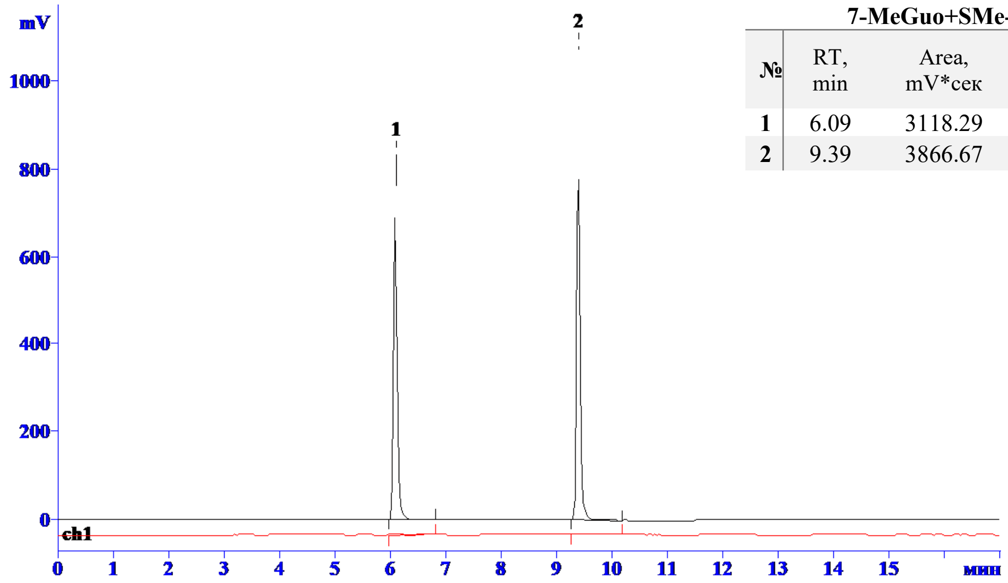

7-MeGuo+SMe-Pur+P_i –1.5:1:0.5 – t_0

№	RT, min	Area, mV*сек	Equilibrium concentration, µM	Name
1	6.09	3118.29	300.00	7-MeGuo
2	9.39	3866.67	200.00	SMe-Pur

$t_{equilibrium}$

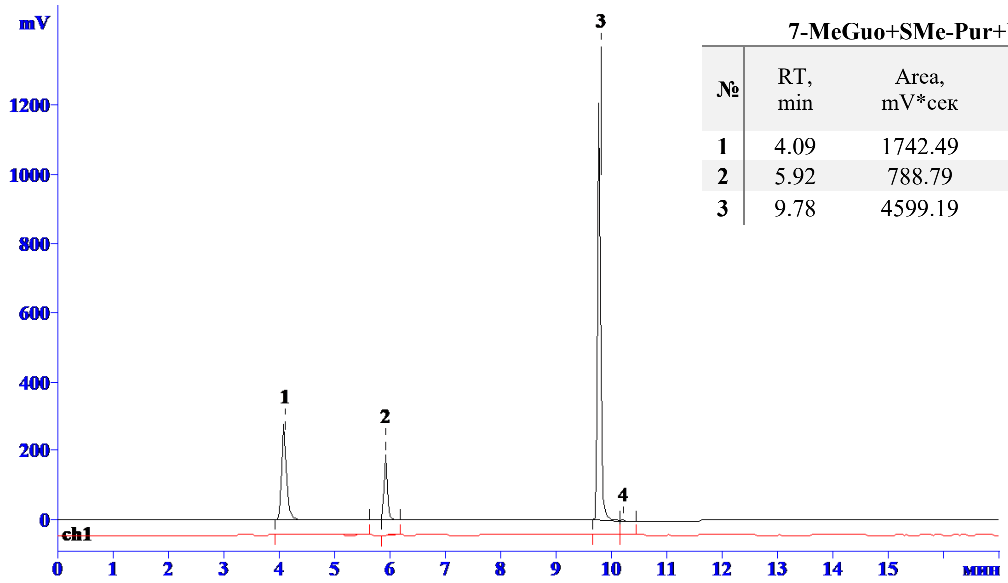

7-MeGuo+SMe-Pur+P_i – 1.5:1:0.5– $t_{equilibrium}$

№	RT, min	Area, mV*сек	Equilibrium concentration, µM	Name
1	4.09	1742.49	224.11	7-MeGua
2	5.92	788.79	75.89	7-MeGuo
3	9.78	4599.19	200	SMe-PurRib

c) $t_{initial}$

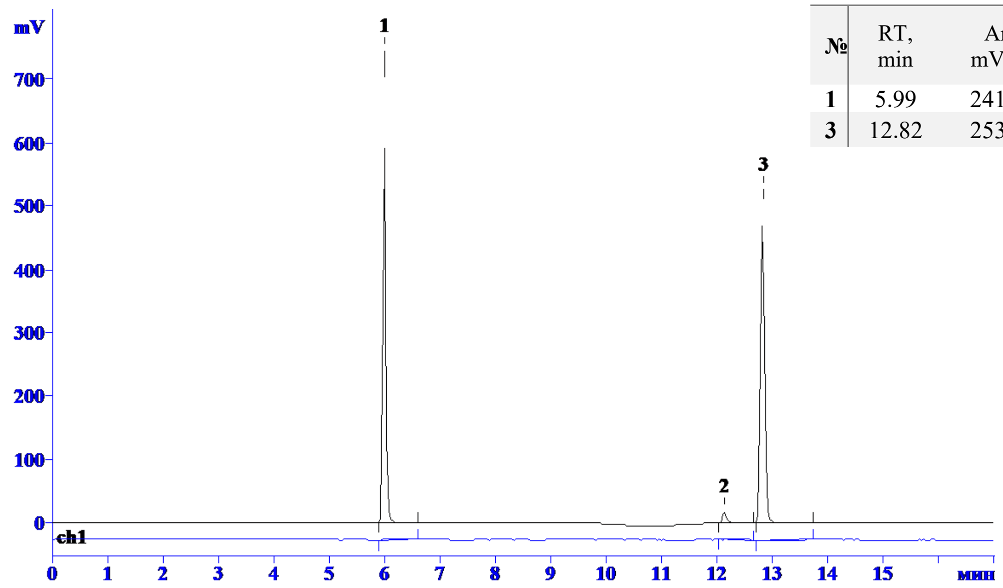

7-MeGuo+SBn-Pur+P_i –1.5:1:0.5 – t_0

№	RT, min	Area, mV*сек	Equilibrium concentration, µM	Name
1	5.99	2414.60	300.00	7-MeGuo
3	12.82	2533.69	200.00	SBn-Pur

$t_{equilibrium}$

7-MeGuo+SBn-Pur+P_i – 1.5:1:0.5– t $_{equilibrium}$

№	RT, min	Area, mV*сек	Equilibrium concentration, µM	Name
1	4.35	856.66	192.46	7-MeGua
2	6.02	865.659	107.54	7-MeGuo
3	12.27	2524.09	200	SBn-PurRib

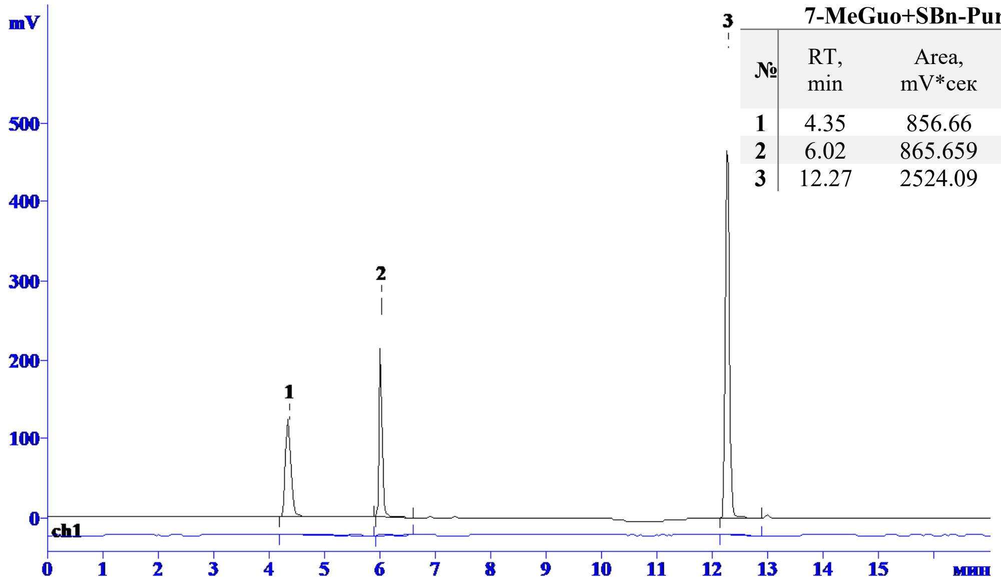

d) $t_{initial}$

7-MeGuo+OMe-Hyp+P_i –1.5:1:0.5 – t_0

№	RT, min	Area, mV*сек	Equilibrium concentration, µM	Name
2	5.60	4488.10	300.00	7-MeGuo
3	7.31	1299.38	200.00	OMe-Hyp

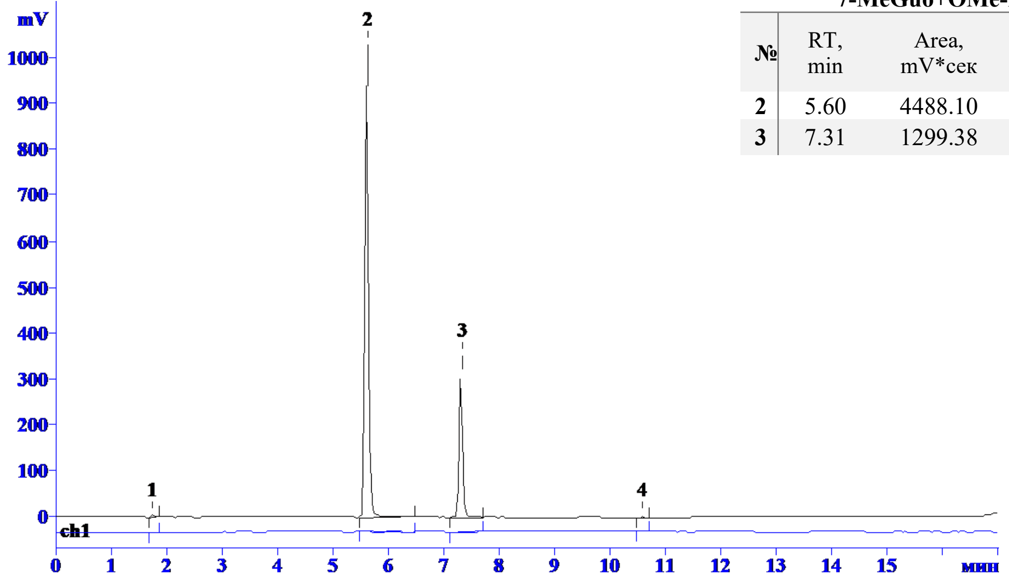

$t_{equilibrium}$

7-MeGuo+OMe-Hyp+P_i – 1.5:1:0.5– t $_{equilibrium}$

№	RT, min	Area, mV*сек	Equilibrium concentration, µM	Name
2	3.92	2190.71	200.93	7-MeGua
3	5.92	1482.11	99.07	7-MeGuo
4	8.61	1261.02	200	OMe-Ino

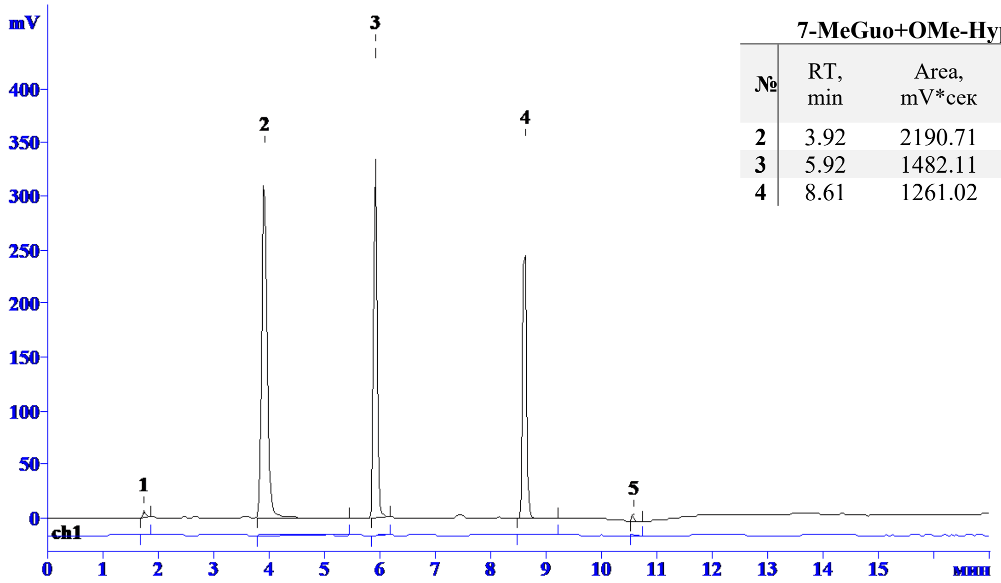

Fugure S6.3. pH = 8.5

a) $t_{initial}$

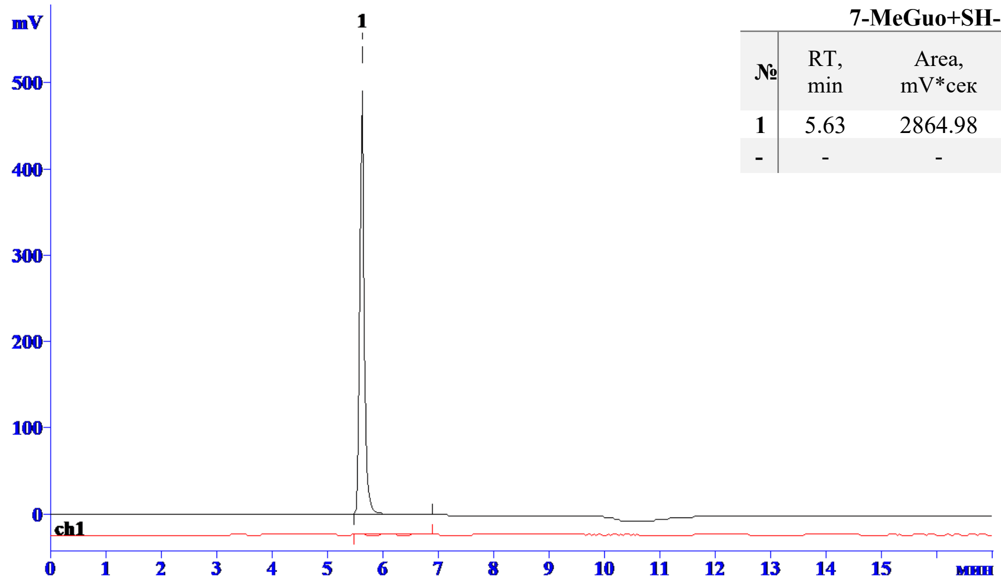

7-MeGuo+SH-Pur+P_i –1.5:1:0.5 – t_0

№	RT, min	Area, mV*сек	Equilibrium concentration, µM	Name
1	5.63	2864.98	200.00	SH-Pur
-	-	-	300.00	7-MeGuo

$t_{equilibrium}$

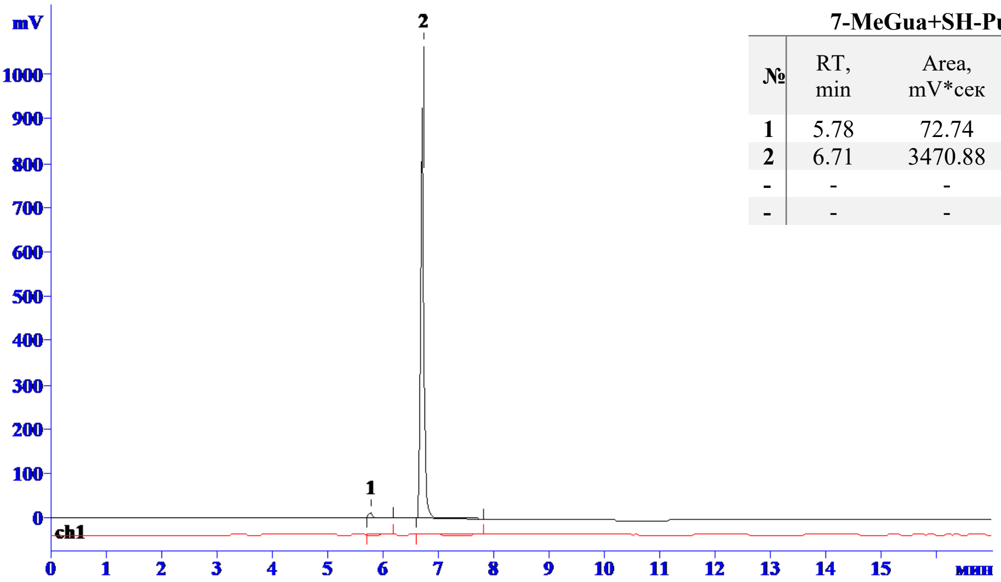

7-MeGua+SH-Pur – 1.5:1:0.5– t $_{equilibrium}$

№	RT, min	Area, mV*сек	Equilibrium concentration, µM	Name
1	5.78	72.74	5.04	SH-Pur
2	6.71	3470.88	194.96	SH-PurRib
-	-	-	-	7-MeGua
-	-	-	-	7-MeGuo

b) $t_{initial}$

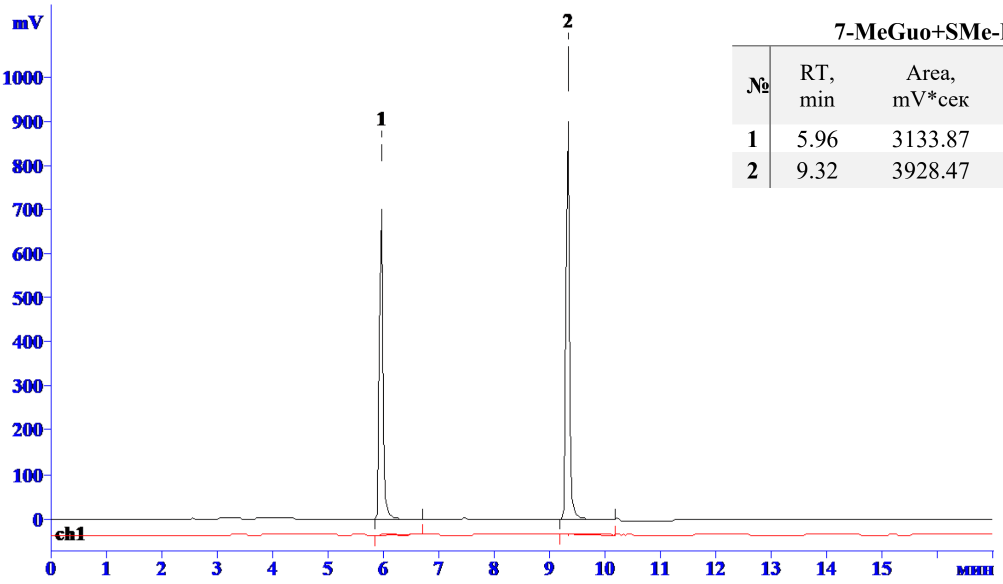

7-MeGuo+SMe-Pur+P_i –1.5:1:0.5 – t_0

№	RT, min	Area, mV*сек	Equilibrium concentration, µM	Name
1	5.96	3133.87	300.00	7-MeGuo
2	9.32	3928.47	200.00	SMe-Pur

$t_{equilibrium}$

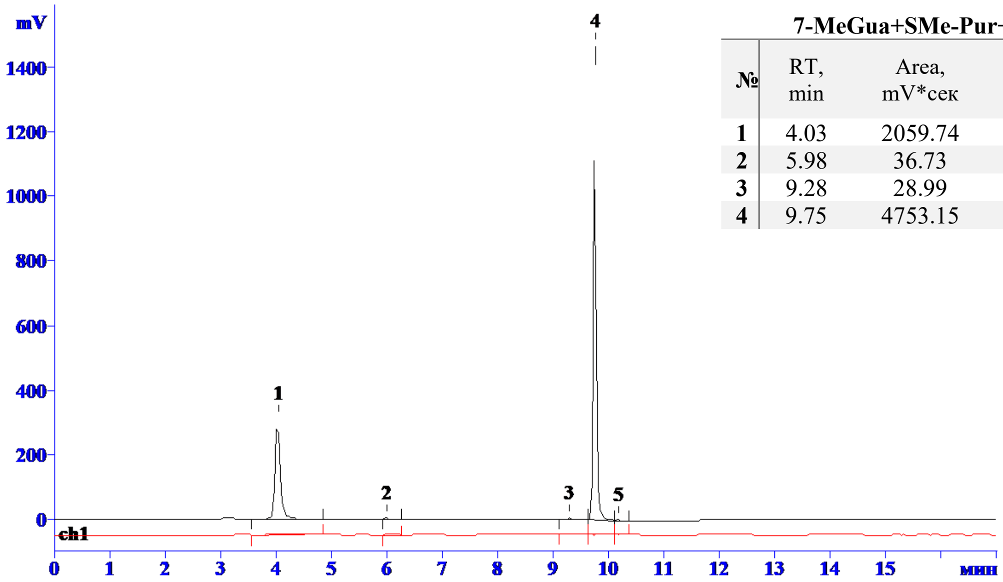

7-MeGua+SMe-Pur+P_i – 1.5:1:0.5– t $_{equilibrium}$

№	RT, min	Area, mV*сек	Equilibrium concentration, µM	Name
1	4.03	2059.74	296.48	7-MeGua
2	5.98	36.73	3.52	7-MeGuo
3	9.28	28.99	1.48	SMe-Pur
4	9.75	4753.15	198.52	SMe-PurRib

c) $t_{initial}$

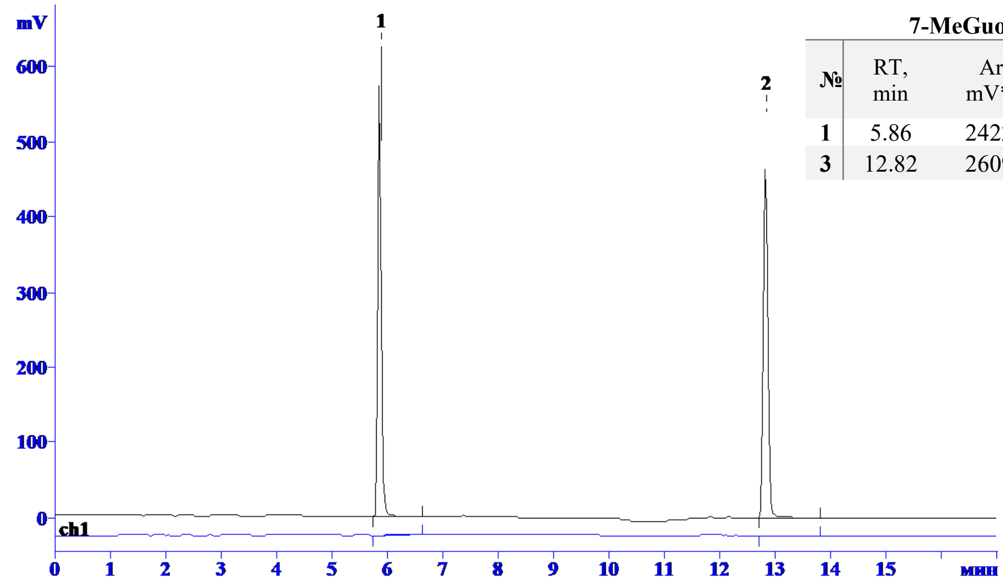

7-MeGuo+SBn-Pur+P_i –1.5:1:0.5 – t_0

№	RT, min	Area, mV*сек	Equilibrium concentration, µM	Name
1	5.86	2422.10	300.00	7-MeGuo
3	12.82	2609.22	200.00	SBn-Pur

$t_{equilibrium}$

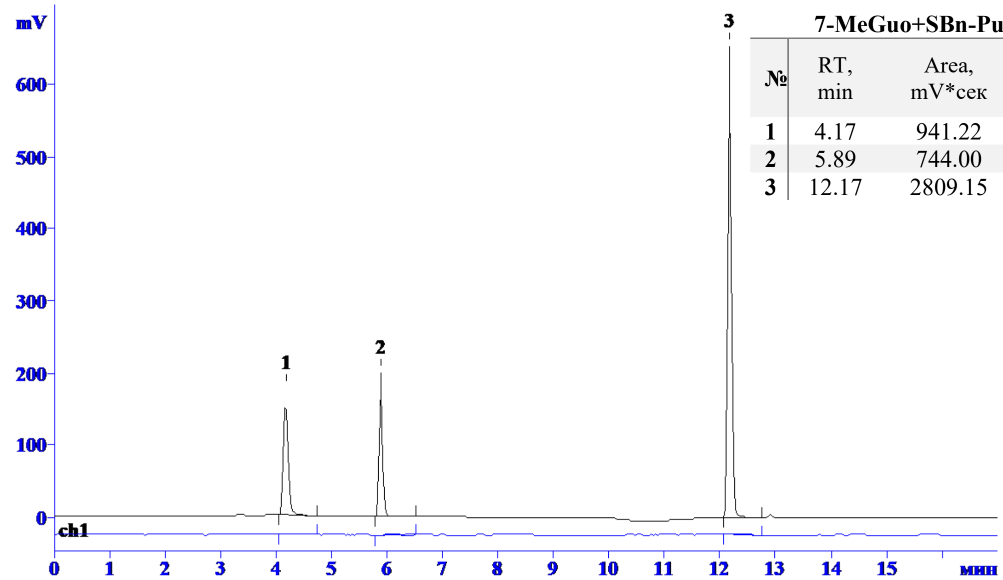

7-MeGuo+SBn-Pur+P_i – 1.5:1:0.5– t $_{equilibrium}$

№	RT, min	Area, mV*сек	Equilibrium concentration, µM	Name
1	4.17	941.22	207.85	7-MeGua
2	5.89	744.00	92.15	7-MeGuo
3	12.17	2809.15	200	SBn-PurRib

d) $t_{initial}$

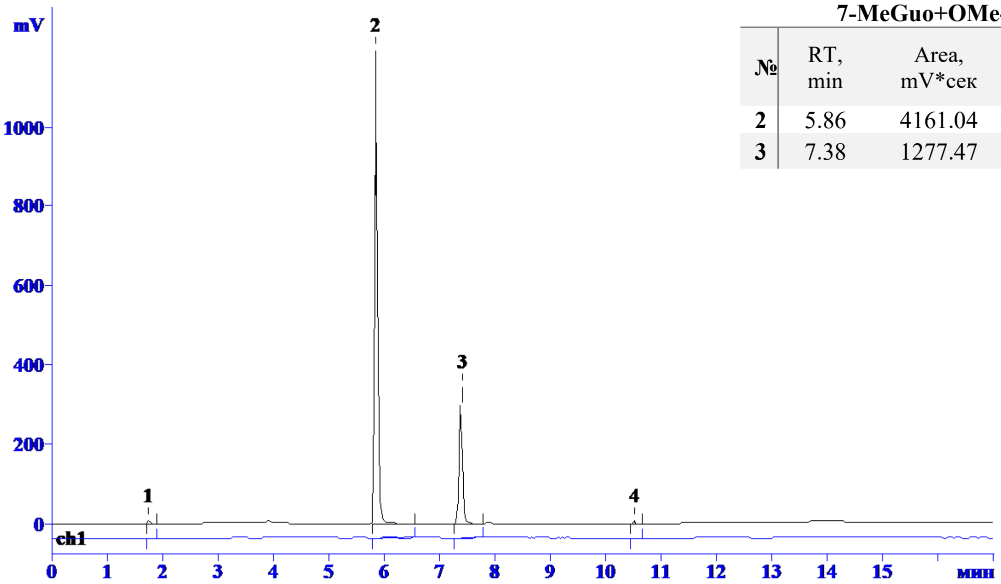

7-MeGuo+OMe-Hyp+P_i –1.5:1:0.5 – t_0

№	RT, min	Area, mV*сек	Equilibrium concentration, µM	Name
2	5.86	4161.04	300.00	7-MeGuo
3	7.38	1277.47	200.00	OMe-Hyp

$t_{equilibrium}$

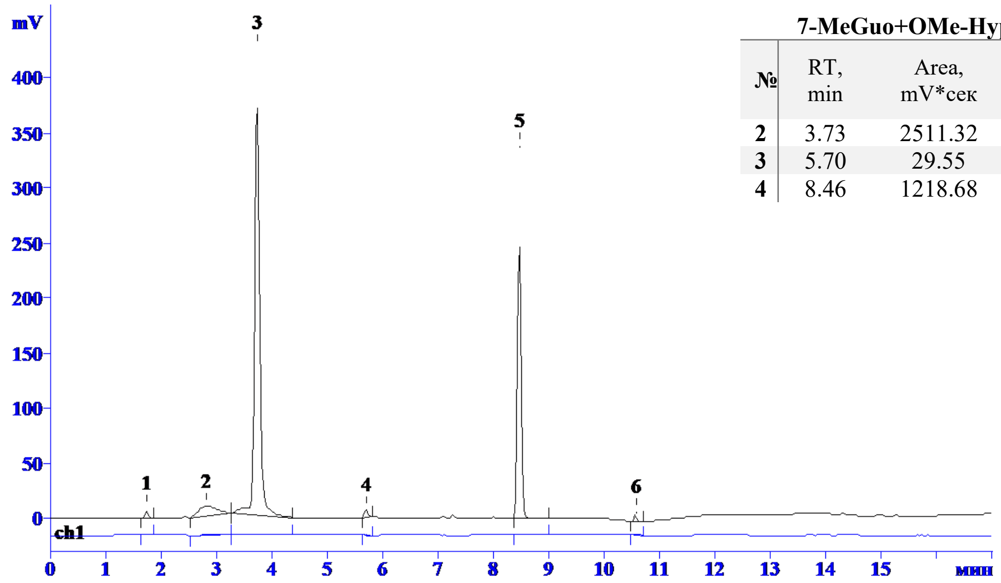

7-MeGuo+OMe-Hyp+P_i – 1.5:1:0.5– $t_{equilibrium}$

№	RT, min	Area, mV*сек	Equilibrium concentration, µM	Name
2	3.73	2511.32	297.87	7-MeGua
3	5.70	29.55	2.13	7-MeGuo
4	8.46	1218.68	200	OMe-Ino

Table S3. Enzymatic transglycosylation reactions of Ade/Ade derivatives and 7-MeGuo, catalysed by PNP *E. coli*, at different pH values[1].

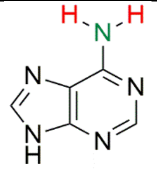	**Ade**		
	pH 5.5	*pH 7.0*	*pH 8.5*
Time, h	$[C]^{Base}$, mkM / $[C]^{Nuc}$, mkM (yield, %)[2]	$[C]^{Base}$, mkM / $[C]^{Nuc}$, mkM (yield, %)[2]	$[C]^{Base}$, mkM / $[C]^{Nuc}$, mkM (yield, %)[2]
1	5.89 / 194.11 (97.06)	0 / 200 (100)	0 / 200 (100)
2	2.26 / 197.74 (98.87)	0 / 200 (100)	0 / 200 (100)
3	0 / 200 (100)	-	-

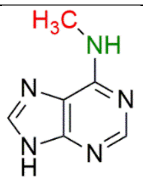

Time, h	**N^6-MeAde** *pH 5.5* $[C]^{Base}$, mkM / $[C]^{Nuc}$, mkM (yield, %)[2]	*pH 7.0* $[C]^{Base}$, mkM / $[C]^{Nuc}$, mkM (yield, %)[2]	*pH 8.5* $[C]^{Base}$, mkM / $[C]^{Nuc}$, mkM (yield, %)[2]
1	38.37 / 161.63 (80.82)	0 / 200 (100)	0 / 200 (100)
2	11.89 / 188.11 (94.05)	-	-
3	4.70 / 195.30 (97.65)	-	-
4	2.62 / 197.38 (98.69)	-	-
5	0 / 200 (100)	-	-

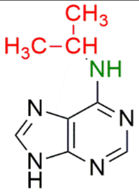

Time, h	**N^6-iPrAde** *pH 5.5* $[C]^{Base}$, mkM / $[C]^{Nuc}$, mkM (yield, %)[2]	*pH 7.0* $[C]^{Base}$, mkM / $[C]^{Nuc}$, mkM (yield, %)[2]	*pH 8.5* $[C]^{Base}$, mkM / $[C]^{Nuc}$, mkM (yield, %)[2]
1.25	48.03 / 151.97 (75.98)	22.60 / 177.40 (88.70)	8.37 / 191.63 (95.82)
2.5	0 / 200 (100)	0 / 200 (100)	0 / 200 (100)

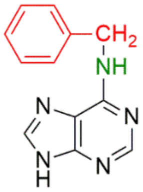

Time, h	**N^6-BnAde** *pH 5.5* $[C]^{Base}$, mkM / $[C]^{Nuc}$, mkM (yield, %)[2]	*pH 7.0* $[C]^{Base}$, mkM / $[C]^{Nuc}$, mkM (yield, %)[2]	*pH 8.5* $[C]^{Base}$, mkM / $[C]^{Nuc}$, mkM (yield, %)[2]
1.33	4.80 / 195.20 (97.60)	0 / 200 (100)	0 / 200 (100)
2.83	1.18 / 198.82 (99.41)	-	-
4.33	0 / 200 (100)	-	-

[1] Reactions were carried out in 50 мМ NaOAc pH 5.5, 50 mM Tris-HCl pH 7.0/8,5, 37°C, PNP *E. coli*, concentration of 7-MeGuo:acceptor:Pi 0.3 mM: 0.2 mM: 0.1 mM (1.5:1:0.5).

[2] The yield was calculated as the ratio of the equilibrium concentration of the target nucleoside to the initial concentration of the limiting substrate.

Table S4. Enzymatic transglycosylation reactions of 6-SHPur/6-SHPur derivatives and 7-MeGuo, catalysed by PNP *E. coli*, at different pH values[1].

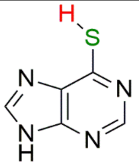	**6-SHPur**		
	pH 5.5	*pH 7.0*	*pH 8.5*
Time, h	$[C]^{Base}$, mkM / $[C]^{Nuc}$, mkM (yield, %)[2]	$[C]^{Base}$, mkM / $[C]^{Nuc}$, mkM (yield, %)[2]	$[C]^{Base}$, mkM / $[C]^{Nuc}$, mkM (yield, %)[2]
1.25	24.89 / 175.11 (87.55)	13.51 / 186.49 (93.24)	6.47 / 193.53 (96.76)
2.5	7.52 / 192.48 (96.24)	5.23 / 194.77 (97.38)	5.04 / 194.96 (97.48)
24	4.85 / 195.15 (97.58)	2.98 / 197.02 (98.51)	8.91 / 191.09 (95.55)
48	6.86 / 193.14 (96.57)	24.40 / 175.60 (87.80)	47.70 / 152.30 (76.15)
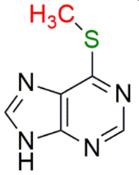	**6-SMePur**		
	pH 5.5	*pH 7.0*	*pH 8.5*
Time, h	$[C]^{Base}$, mkM / $[C]^{Nuc}$, mkM (yield, %)[2]	$[C]^{Base}$, mkM / $[C]^{Nuc}$, mkM (yield, %)[2]	$[C]^{Base}$, mkM / $[C]^{Nuc}$, mkM (yield, %)[2]
1.25	9.39 / 190.61 (95.31)	2.19 / 197.81 (98.91)	12.50 / 187.50 (93.75)
2.66	2.86 / 197.14 (98.57)	0 / 200 (100)	2.46 / 197.54 (98.77)
24	0 / 200 (100)	0 / 200 (100)	1.71 / 198.29 (99.14)
48	-	-	1.48 / 198.52 (99.26)
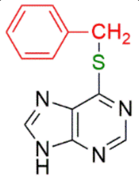	**6-SBnPur**		
	pH 5.5	*pH 7.0*	*pH 8.5*
Time, h	$[C]^{Base}$, mkM / $[C]^{Nuc}$, mkM (yield, %)[2]	$[C]^{Base}$, mkM / $[C]^{Nuc}$, mkM (yield, %)[2]	$[C]^{Base}$, mkM / $[C]^{Nuc}$, mkM (yield, %)[2]
0.33	18.81 / 181.19 (90.60)	4.72 / 195.28 (97.64)	9.98 / 190.02 (95.01)
1	0 / 200 (100)	0 / 200 (100)	0 / 200 (100)

[1] Reactions were carried out in 50 mM NaOAc pH 5.5, 50 mM Tris-HCl pH 7.0/8,5, 37°C, PNP *E. coli*, concentration of 7-MeGuo:acceptor:Pi 0.3 mM: 0.2 mM: 0.1 mM (1.5:1:0.5).

[2] The yield was calculated as the ratio of the equilibrium concentration of the target nucleoside to the initial concentration of the limiting substrate.

Table S5. Enzymatic transglycosylation reactions of Hyp/Hyp derivatives and 7-MeGuo, catalysed by PNP *E. coli*, at different pH values[1].

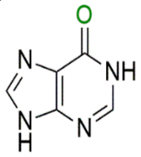	**Hyp**		
	pH 5.5	*pH 7.0*	*pH 8.5*
Time, h	$[C]^{Base}$, mkM / $[C]^{Nuc}$, mkM (yield, %)[2]	$[C]^{Base}$, mkM / $[C]^{Nuc}$, mkM (yield, %)[2]	$[C]^{Base}$, mkM / $[C]^{Nuc}$, mkM (yield, %)[2]
1	120.75 / 79.25 (39.62)	23.48 / 176.52 (88.26)	19.69 / 180.31 (90,15)
2	96.46 / 103.54 (51.77)	5.30 / 194.70 (97.35)	8.74 / 191.26 (95.63)
3	82.25 / 117.75 (58.88)	-	-
24	30.37 / 169.63 (84.82)	0 / 200 (100)	0 / 200 (100)
48	17.36 / 182.64 (91.32)	0 / 200 (100)	0 / 200 (100)
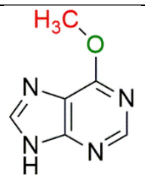	**6-OMePur**		
	pH 5.5	*pH 7.0*	*pH 8.5*
Time, h	$[C]^{Base}$, mkM / $[C]^{Nuc}$, mkM (yield, %)[2]	$[C]^{Base}$, mkM / $[C]^{Nuc}$, mkM (yield, %)[2]	$[C]^{Base}$, mkM / $[C]^{Nuc}$, mkM (yield, %)[2]
0.33	63.62 / 136.38 (68.19)	92.71 / 107.29 (53.64)	121.04 / 78.96 (39.48)
1.5	0 / 200 (100)	0 / 200 (100)	4.64 / 195.36 (97.68)
24	0 / 200 (100)	0 / 200 (100)	0 / 200 (100)
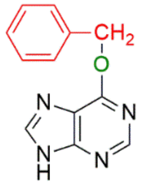	**6-OBnPur**		
	pH 5.5	*pH 7.0*	*pH 8.5*
Time, h	$[C]^{Base}$, mkM / $[C]^{Nuc}$, mkM (yield, %)[2]	$[C]^{Base}$, mkM / $[C]^{Nuc}$, mkM (yield, %)[2]	$[C]^{Base}$, mkM / $[C]^{Nuc}$, mkM (yield, %)[2]
0.5	-	-	-
2.33	-	0 / 200 (100)	0 / 200 (100)

[1] Reactions were carried out in 50 mM NaOAc pH 5.5, 50 mM Tris-HCl pH 7.0/8,5, 37°C, PNP *E. coli*, concentration of 7-MeGuo:acceptor:Pi 0.3 mM: 0.2 mM: 0.1 mM (1.5:1:0.5).

[2] The yield was calculated as the ratio of the equilibrium concentration of the target nucleoside to the initial concentration of the limiting substrate.

Transglycosylation reactions catalysed by NDT II.

2′-deoxy-5-hydroxymethyluridine from 7-methyldeoxyguanosine (7-MedGuo+5-hmUra, analytical method)

HPLC-analysis of transglycosylation: Cosmosil 5C18-MS-II, 4.6*150 mm, 5µm, 120 Å, (Nacalai Tesque, Inc. (Japan)), 20ºC, elution in modified mobile phase: linear gradient of acetonitrile in 0.06% TFA from 2 to 12% for 10 min (flushing with 12-80 % acetonitrile-0.06% TFA for 10-10.1 min, then 80-2% for 10.1-10.8 min) at flow rate 1 ml/min with UV detection at wavelength 263 nm, injection volume 20 µL.

Fugure S7.1. pH = 5.5

1) 2.6*10^{-3} U *LlNDT II*

$t_{initial}$

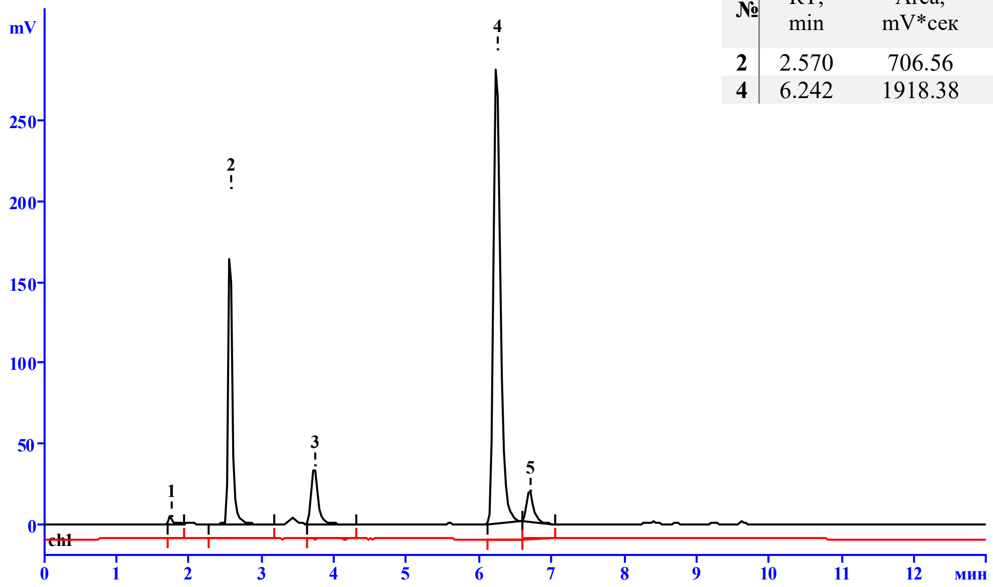

7-MedGuo+5-hmUra – 1:3 – t_0

№	RT, min	Area, mV*сек	Equilibrium concentration, µM	Name
2	2.570	706.56	100.00	5-hmUra
4	6.242	1918.38	300.00	7-MedGuo

$t_{equilibrium}$

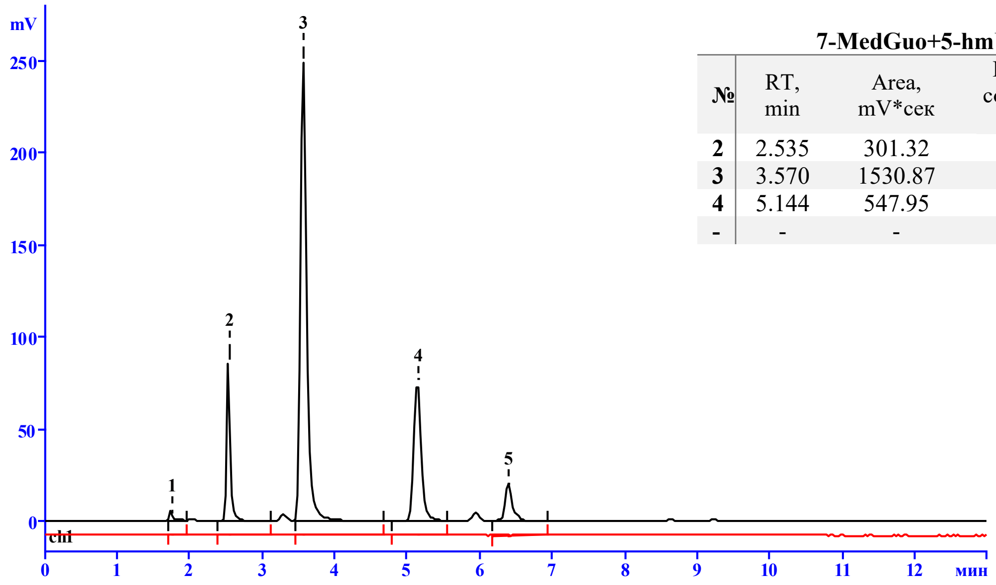

7-MedGuo+5-hmUra – 1:3– t $_{equilibrium}$

№	RT, min	Area, mV*сек	Equilibrium concentration, µM	Name
2	2.535	301.32	42.65	5-hmUra
3	3.570	1530.87	300.00	7-MeGua
4	5.144	547.95	57.35	5-hmdUrd
-	-	-	-	7-MedGuo

2) 7.8*10^{-3} U *LlNDT II*

$t_{initial}$

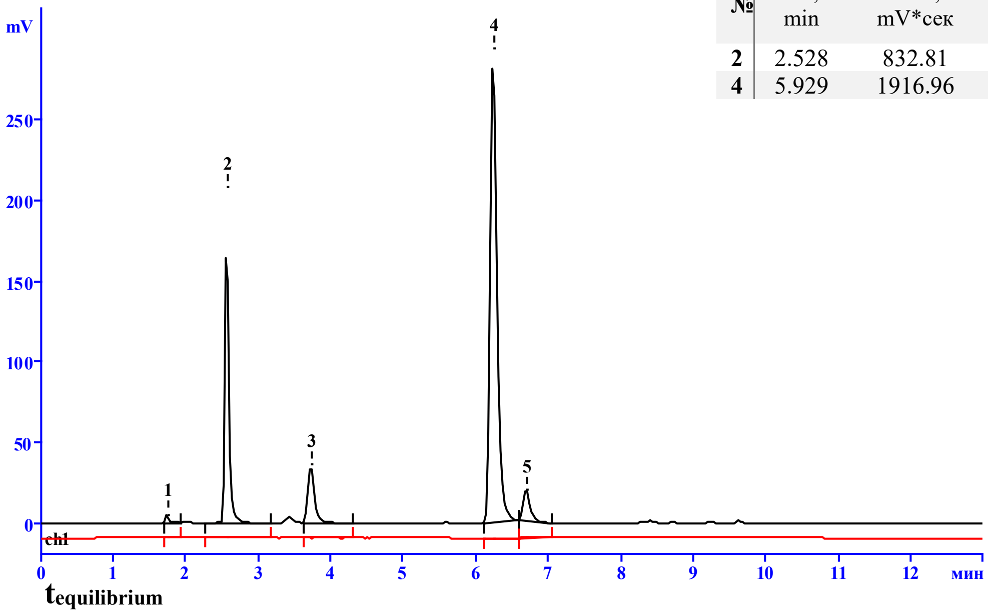

7-MedGuo+5-hmUra – 1:3 – t_0

№	RT, min	Area, mV*сек	Equilibrium concentration, µM	Name
**2**	2.528	832.81	100.00	5-hmUra
**4**	5.929	1916.96	300.00	7-MedGuo

$t_{equilibrium}$

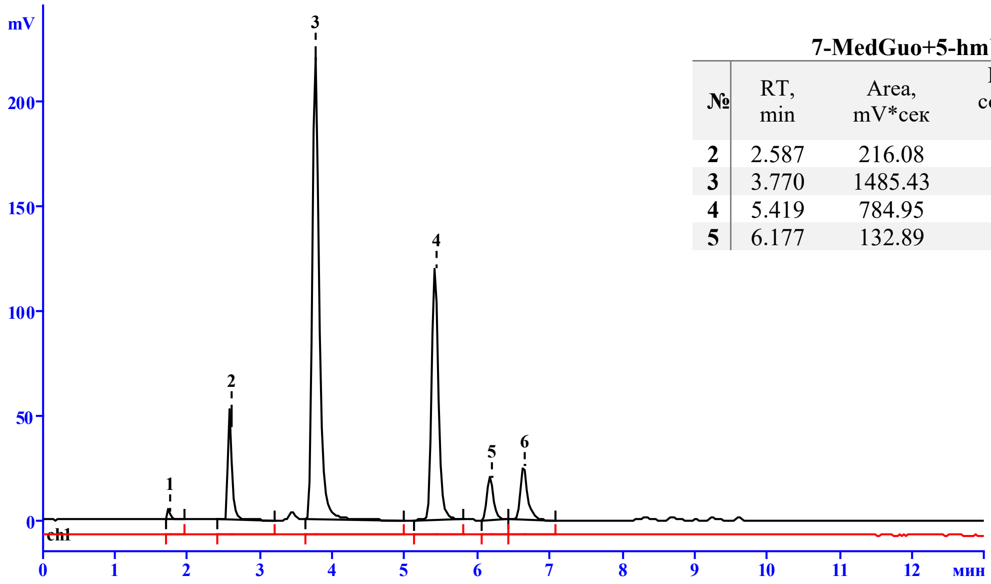

7-MedGuo+5-hmUra – 1:3– t $_{equilibrium}$

№	RT, min	Area, mV*сек	Equilibrium concentration, µM	Name
**2**	2.587	216.08	25.95	5-hmUra
**3**	3.770	1485.43	279.20	7-MeGua
**4**	5.419	784.95	74.05	5-hmdUrd
**5**	6.177	132.89	20.80	7-MedGuo

3) 13*10^{-3} U *LlNDT II*

$t_{initial}$

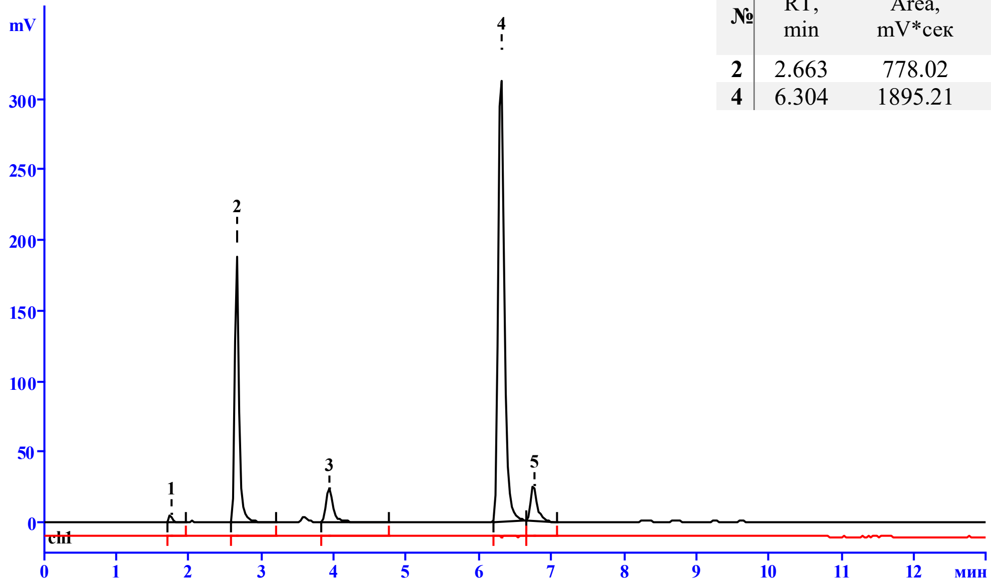

7-MedGuo+5-hmUra – 1:3 – t_0

№	RT, min	Area, mV*сек	Equilibrium concentration, µM	Name
**2**	2.663	778.02	100.00	5-hmUra
**4**	6.304	1895.21	300.00	7-MedGuo

$t_{equilibrium}$

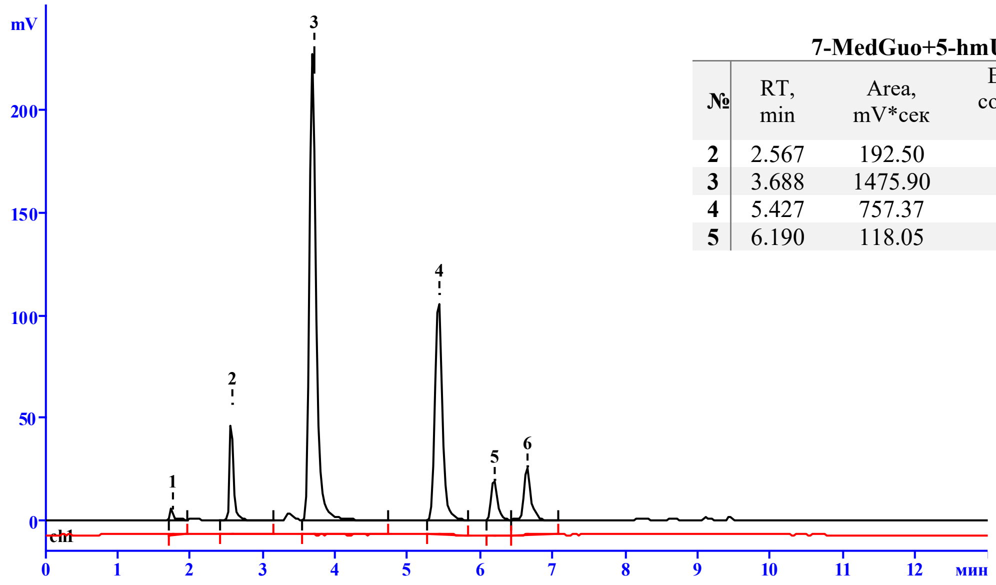

7-MedGuo+5-hmUra – 1:3– t $_{equilibrium}$

№	RT, min	Area, mV*сек	Equilibrium concentration, µM	Name
**2**	2.567	192.50	24.74	5-hmUra
**3**	3.688	1475.90	281.31	7-MeGua
**4**	5.427	757.37	75.26	5-hmdUrd
**5**	6.190	118.05	18.69	7-MedGuo

4) $26*10^{-3}$ U *LlNDT II*

7-MedGuo+5-hmUra – 1:3 – t$_0$

№	RT, min	Area, mV*сек	Equilibrium concentration, µM	Name
**2**	2.577	833.21	100.00	5-hmUra
**4**	6.055	2072.39	300.00	7-MedGuo

$t_{initial}$

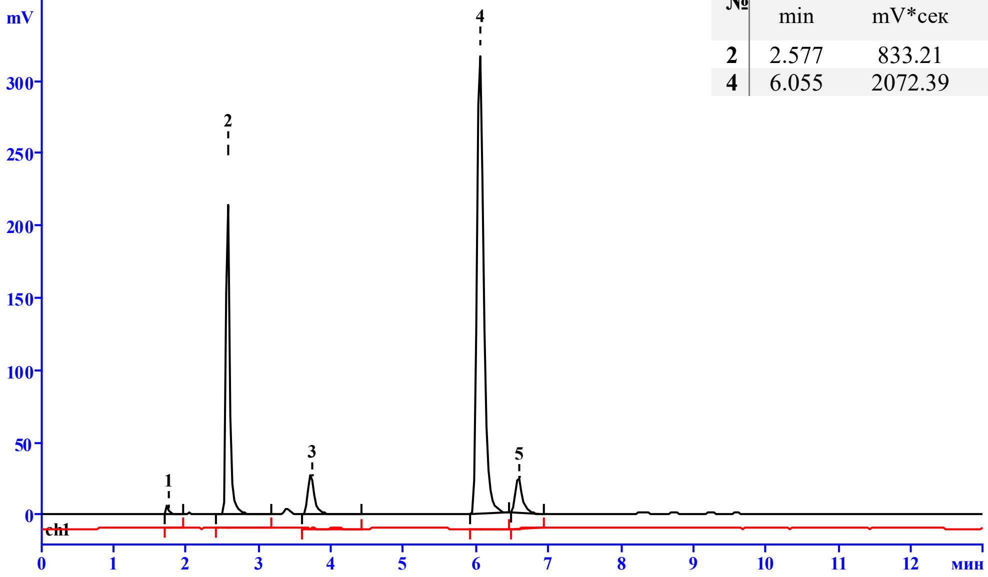

$t_{equilibrium}$

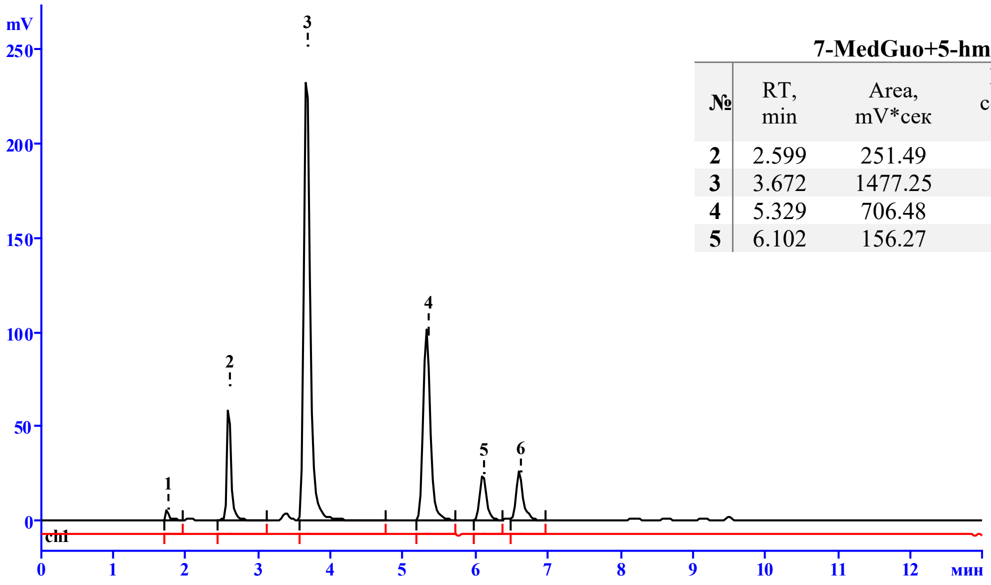

7-MedGuo+5-hmUra – 1:3– t $_{equilibrium}$

№	RT, min	Area, mV*сек	Equilibrium concentration, µM	Name
**2**	2.599	251.49	30.18	5-hmUra
**3**	3.672	1477.25	277.38	7-MeGua
**4**	5.329	706.48	69.82	5-hmdUrd
**5**	6.102	156.27	22.62	7-MedGuo

5) 52*10^{-3} U *LlNDT II*

7-MedGuo+5-hmUra – 1:3 – t_0

№	RT, min	Area, mV*сек	Equilibrium concentration, µM	Name
**2**	2.577	841.28	100.00	5-hmUra
**4**	6.055	2104.28	300.00	7-MedGuo

$t_{initial}$

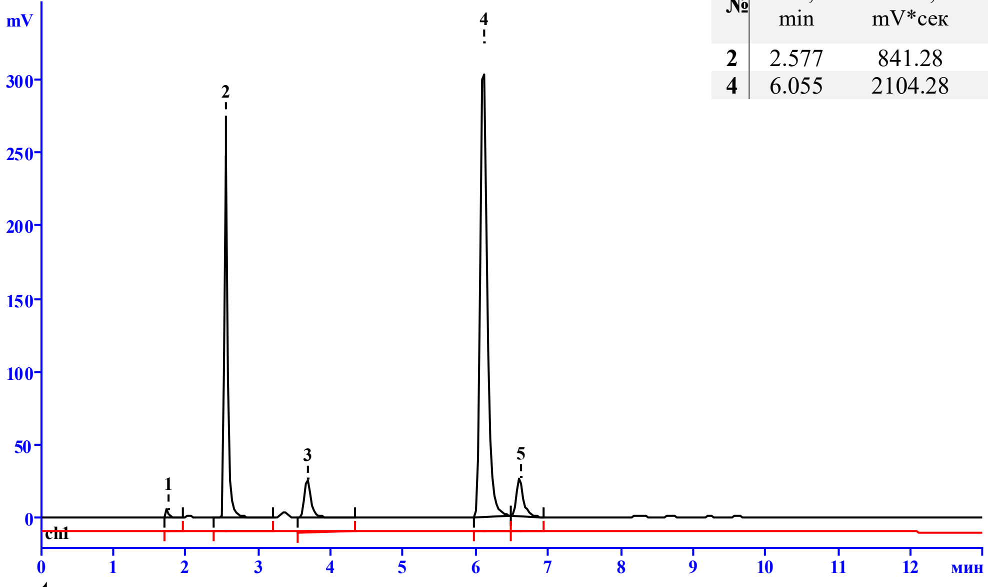

$t_{equilibrium}$

7-MedGuo+5-hmUra – 1:3– t equilibrium

№	RT, min	Area, mV*сек	Equilibrium concentration, µM	Name
**2**	2.586	221.16	26.29	5-hmUra
**3**	3.758	1399.99	260.86	7-MeGua
**4**	5.493	757.59	73.71	5-hmdUrd
**5**	6.299	274.53	39.14	7-MedGuo

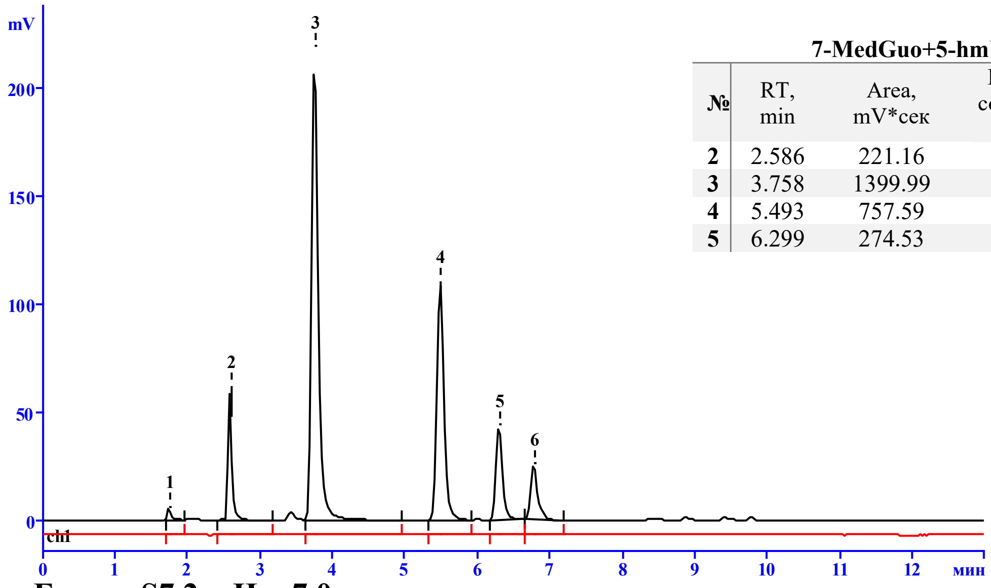

Fugure S7.2. <u>pH = 7.0</u>

1) 2.6*10^{-3} U *LlNDT II*

7-MedGuo+5-hmUra – 1:3 – t_0

№	RT, min	Area, mV*сек	Equilibrium concentration, µM	Name
**2**	2.550	641.82	100.00	5-hmUra
**4**	6.063	2143.89	300.00	7-MedGuo

$t_{initial}$

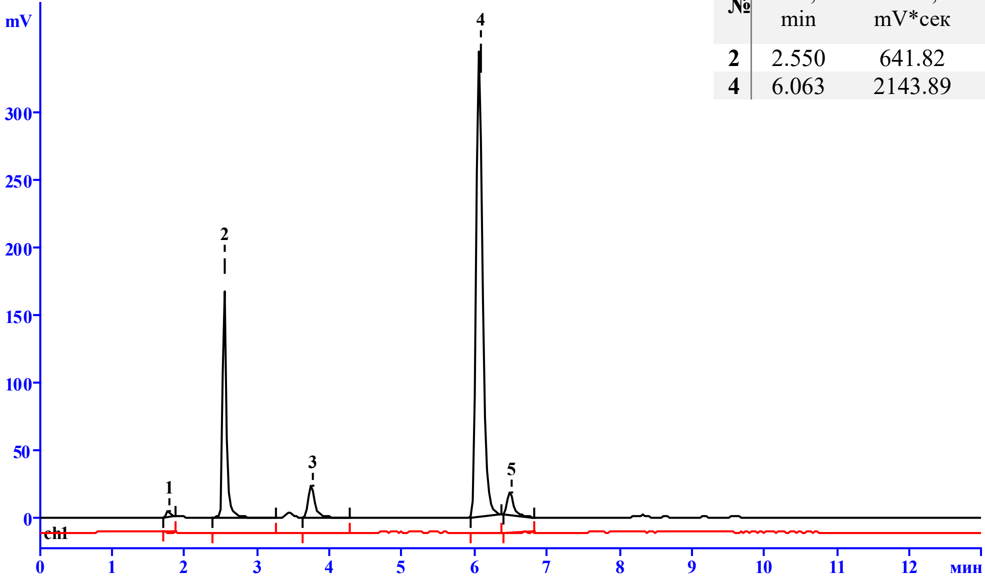

$t_{equilibrium}$

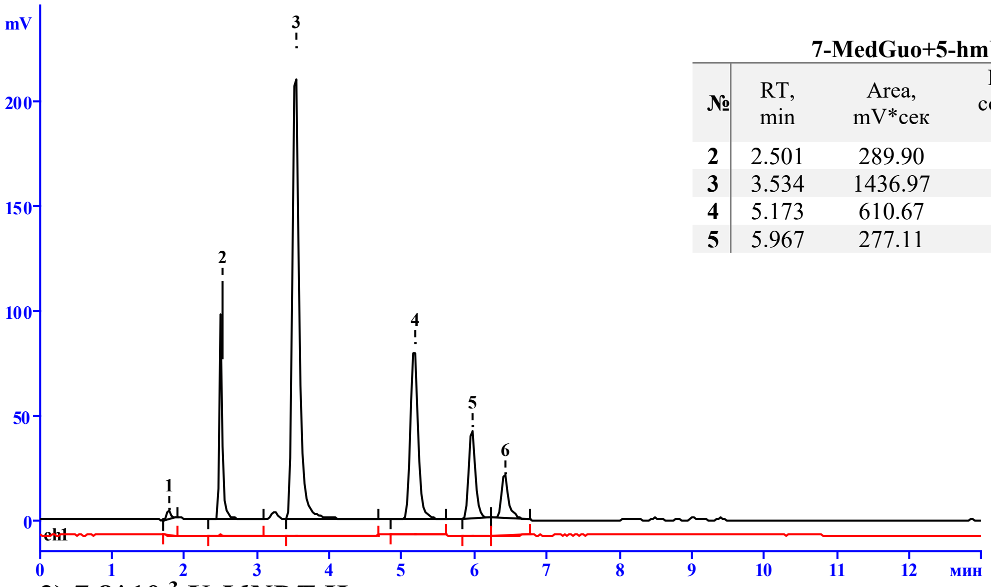

7-MedGuo+5-hmUra – 1:3– t $_{equilibrium}$

№	RT, min	Area, mV*сек	Equilibrium concentration, µM	Name
**2**	2.501	289.90	45.17	5-hmUra
**3**	3.534	1436.97	261.22	7-MeGua
**4**	5.173	610.67	54.83	5-hmdUrd
**5**	5.967	277.11	38.78	7-MedGuo

2) 7.8*10^{-3} U *LlNDT II*

$t_{initial}$

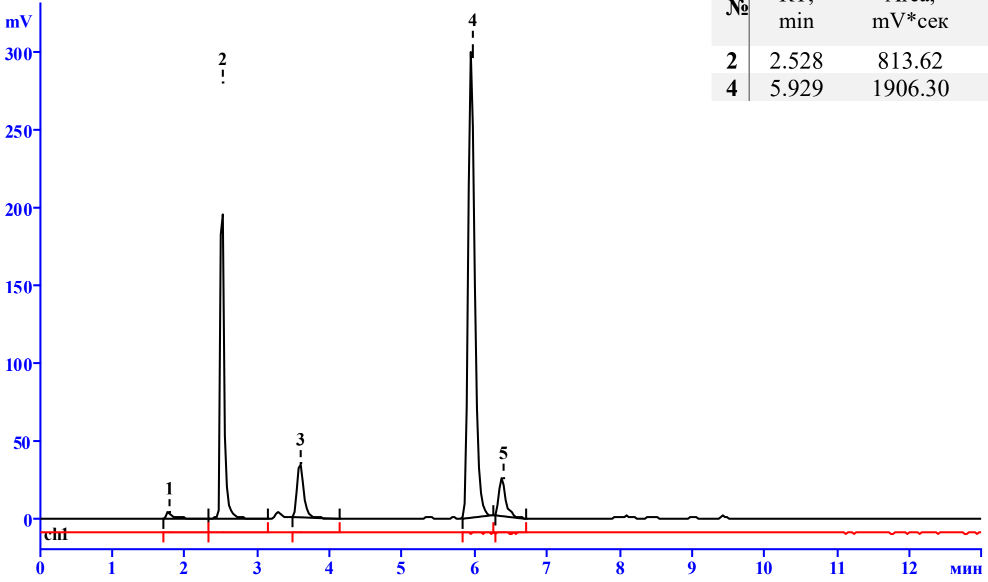

7-MedGuo+5-hmUra – 1:3 – t$_0$

№	RT, min	Area, mV*сек	Equilibrium concentration, µM	Name
**2**	2.528	813.62	100.00	5-hmUra
**4**	5.929	1906.30	300.00	7-MedGuo

$t_{equilibrium}$

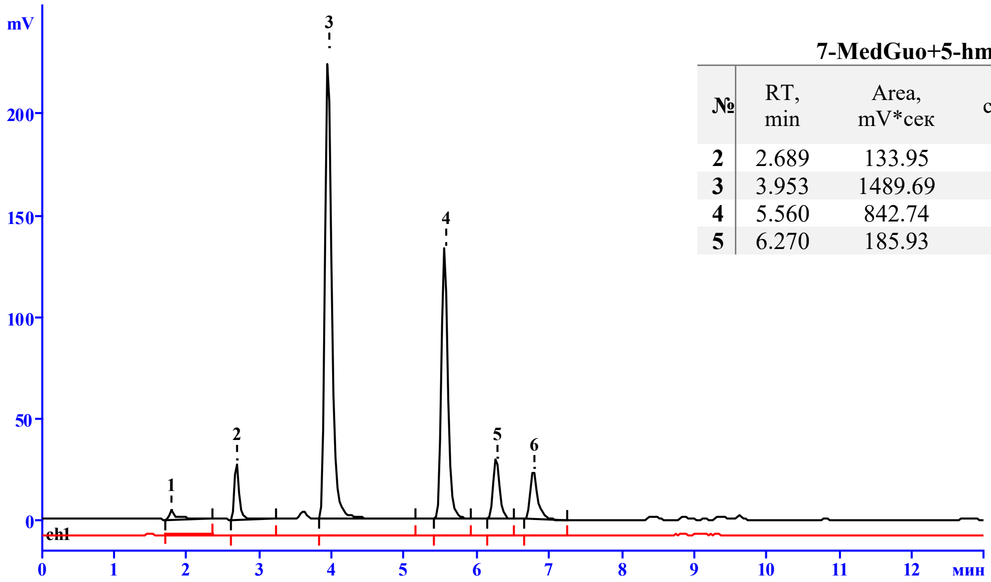

7-MedGuo+5-hmUra – 1:3– t $_{equilibrium}$

№	RT, min	Area, mV*сек	Equilibrium concentration, µM	Name
**2**	2.689	133.95	16.46	5-hmUra
**3**	3.953	1489.69	270.74	7-MeGua
**4**	5.560	842.74	83.54	5-hmdUrd
**5**	6.270	185.93	29.26	7-MedGuo

3) 13*10^{-3} U *LlNDT II*

$t_{initial}$

7-MedGuo+5-hmUra – 1:3 – t_0

№	RT, min	Area, mV*сек	Equilibrium concentration, μM	Name
**2**	2.706	705.80	100.00	5-hmUra
**3**	6.298	1735.33	300.00	7-MedGuo

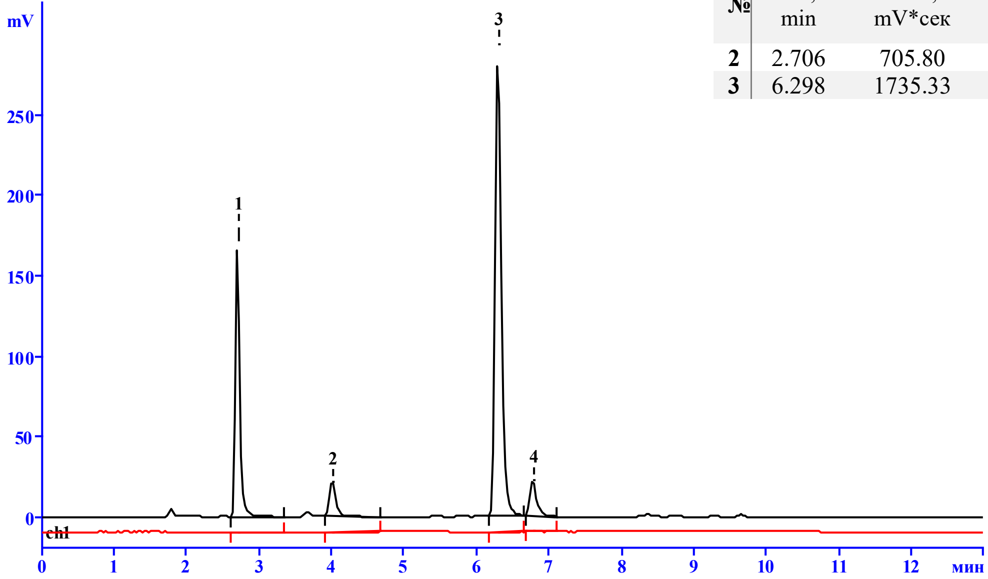

$t_{equilibrium}$

7-MedGuo+5-hmUra – 1:3– t $_{equilibrium}$

№	RT, min	Area, mV*сек	Equilibrium concentration, μM	Name
**2**	2.606	104.24	14.77	5-hmUra
**3**	3.802	1351.89	300.00	7-MeGua
**4**	5.500	733.40	85.23	5-hmdUrd
**-**	-	-	-	7-MedGuo

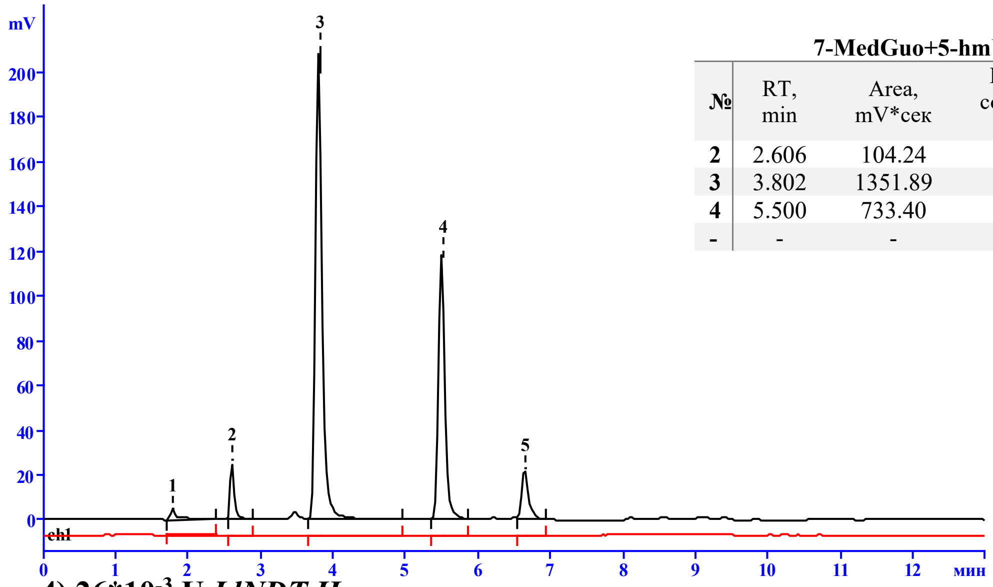

4) 26*10^{-3} U *LlNDT II*

$t_{initial}$

7-MedGuo+5-hmUra – 1:3 – t_0

№	RT, min	Area, mV*сек	Equilibrium concentration, μM	Name
**2**	2.636	789.18	100.00	5-hmUra
**4**	6.266	2000.39	300.00	7-MedGuo

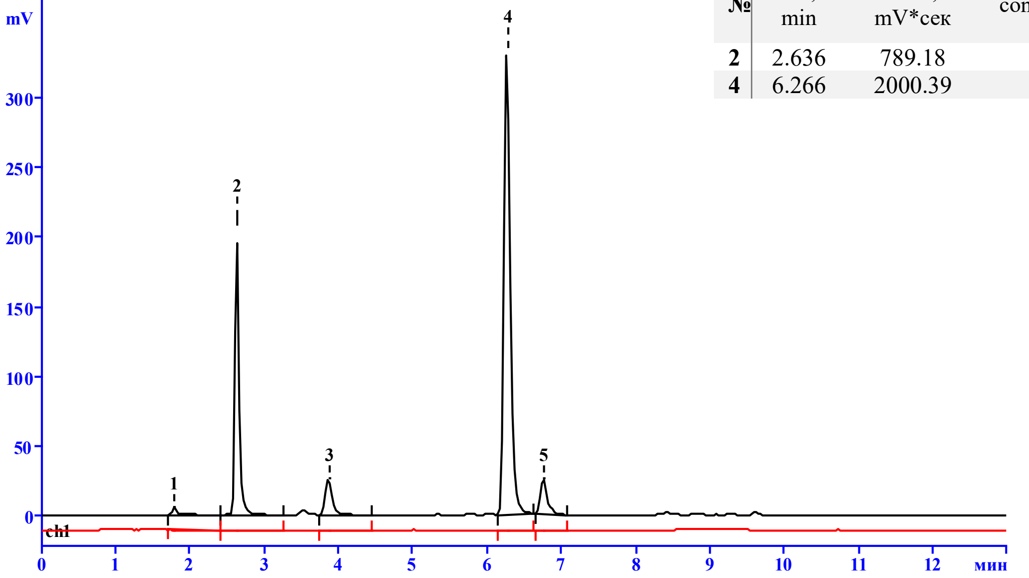

$t_{equilibrium}$

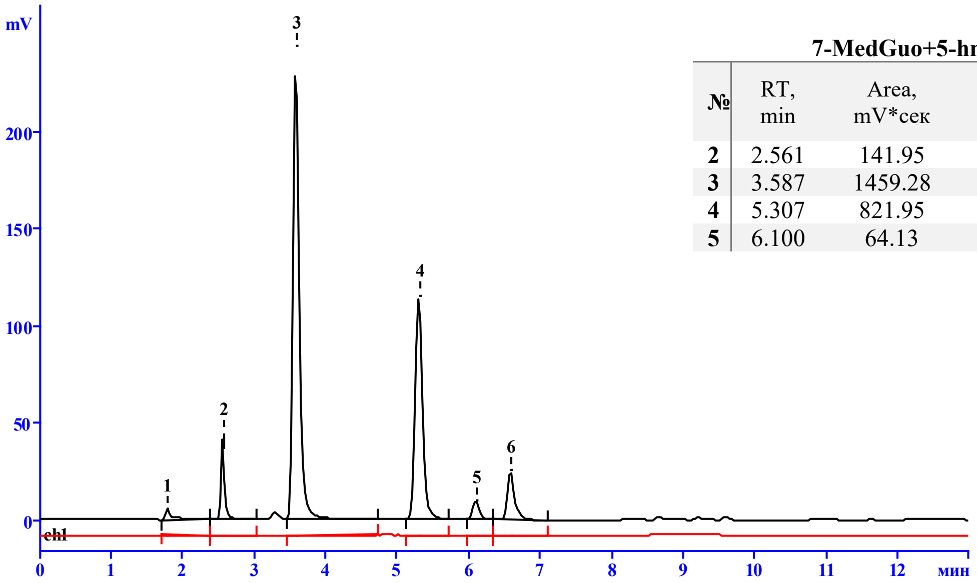

7-MedGuo+5-hmUra – 1:3– t equilibrium

№	RT, min	Area, mV*сек	Equilibrium concentration, µM	Name
**2**	2.561	141.95	17.99	5-hmUra
**3**	3.587	1459.28	290.38	7-MeGua
**4**	5.307	821.95	82.01	5-hmdUrd
**5**	6.100	64.13	9.62	7-MedGuo

5) 52*10^{-3} U *LlNDT II*

$t_{initial}$

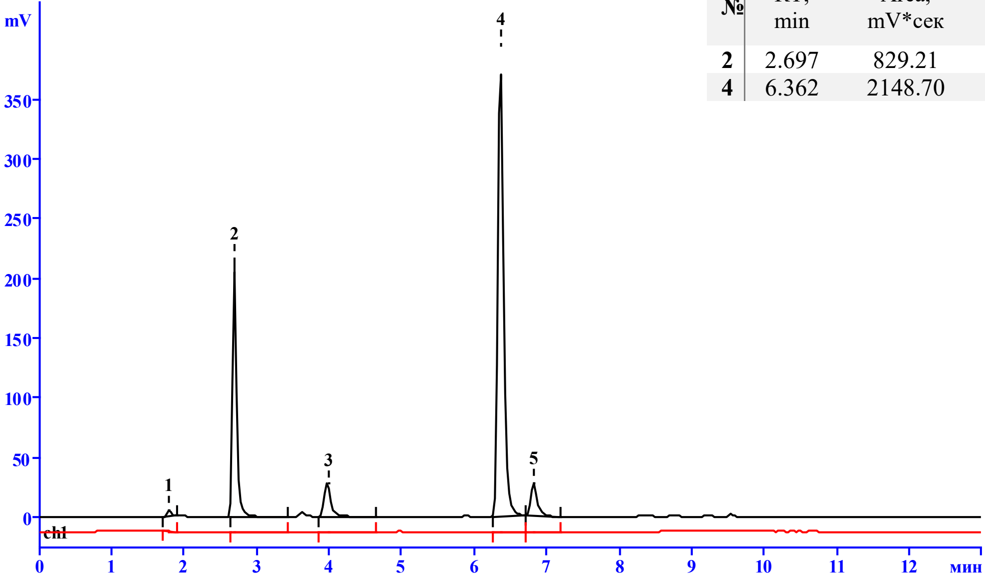

7-MedGuo+5-hmUra – 1:3 – t_0

№	RT, min	Area, mV*сек	Equilibrium concentration, µM	Name
**2**	2.697	829.21	100.00	5-hmUra
**4**	6.362	2148.70	300.00	7-MedGuo

$t_{equilibrium}$

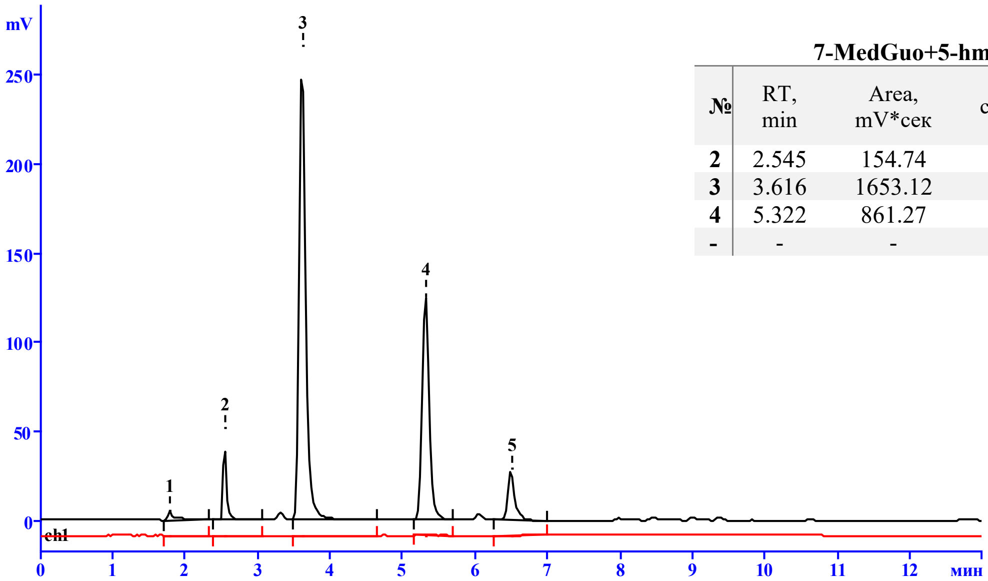

7-MedGuo+5-hmUra – 1:3– t equilibrium

№	RT, min	Area, mV*сек	Equilibrium concentration, µM	Name
**2**	2.545	154.74	18.66	5-hmUra
**3**	3.616	1653.12	300.00	7-MeGua
**4**	5.322	861.27	81.34	5-hmdUrd
**-**	-	-	-	7-MedGuo

Fugure S7.3. pH = 8.5

1) 2.6*10^{-3} U *LlNDT II*

$t_{initial}$

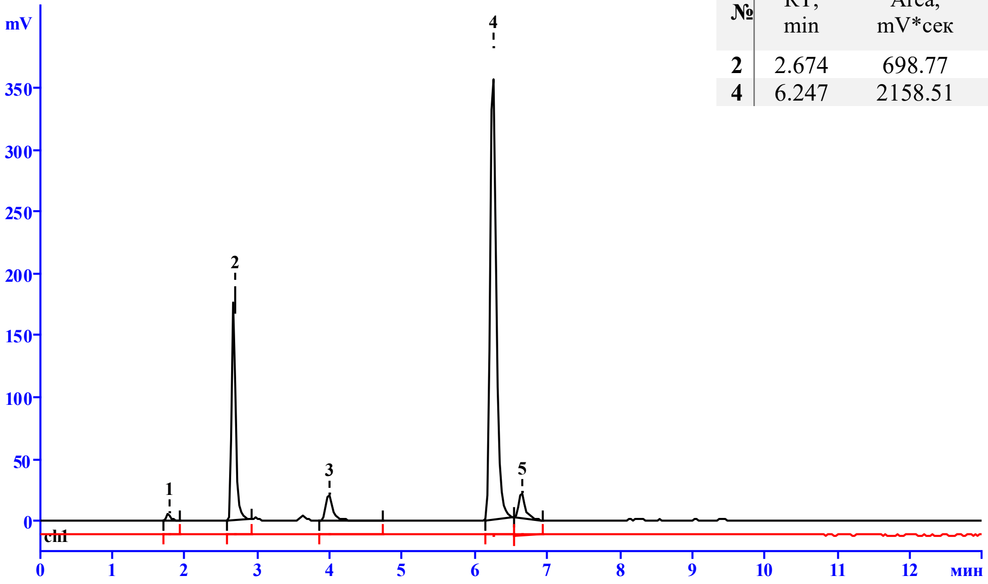

7-MedGuo+5-hmUra – 1:3 – t_0

№	RT, min	Area, mV*сек	Equilibrium concentration, µM	Name
**2**	2.674	698.77	100.00	5-hmUra
**4**	6.247	2158.51	300.00	7-MedGuo

$t_{equilibrium}$

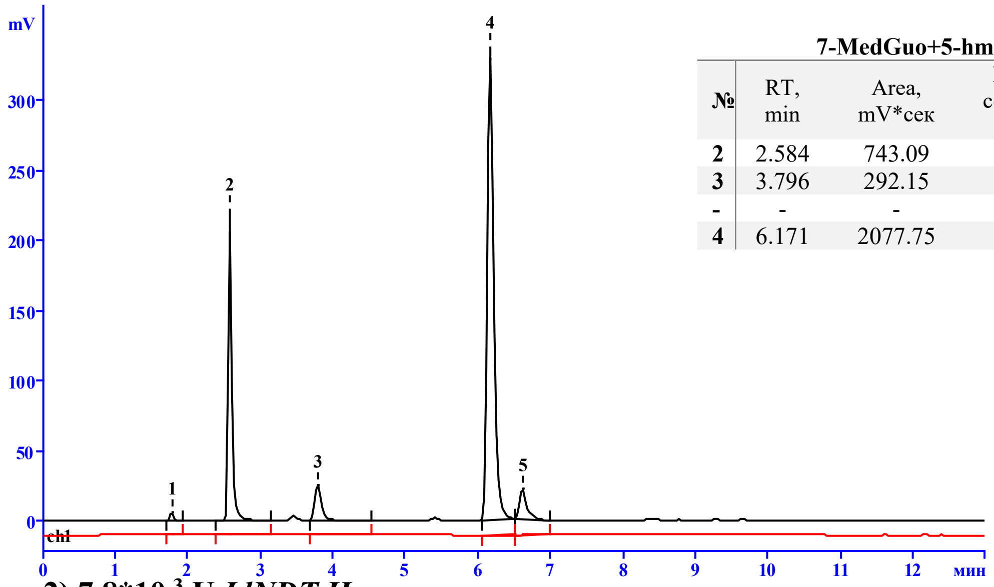

7-MedGuo+5-hmUra – 1:3– t $_{equilibrium}$

№	RT, min	Area, mV*сек	Equilibrium concentration, µM	Name
**2**	2.584	743.09	100.00	5-hmUra
**3**	3.796	292.15	11.22	7-MeGua
**-**	-	-	-	5-hmdUrd
**4**	6.171	2077.75	288.78	7-MedGuo

2) 7.8*10^{-3} U *LlNDT II*

$t_{initial}$

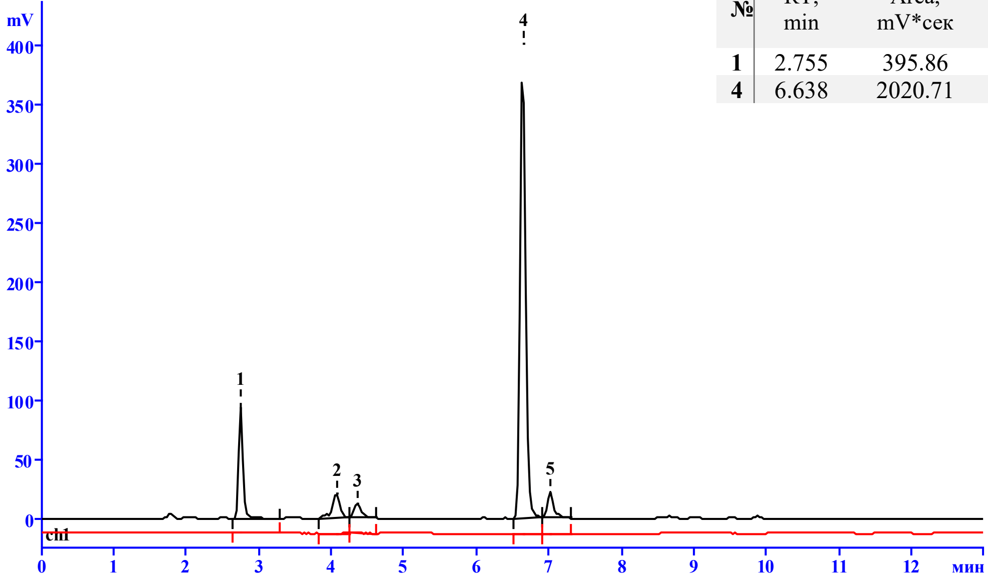

7-MedGuo+5-hmUra – 1:3 – t_0

№	RT, min	Area, mV*сек	Equilibrium concentration, µM	Name
**1**	2.755	395.86	100.00	5-hmUra
**4**	6.638	2020.71	300.00	7-MedGuo

t equilibrium

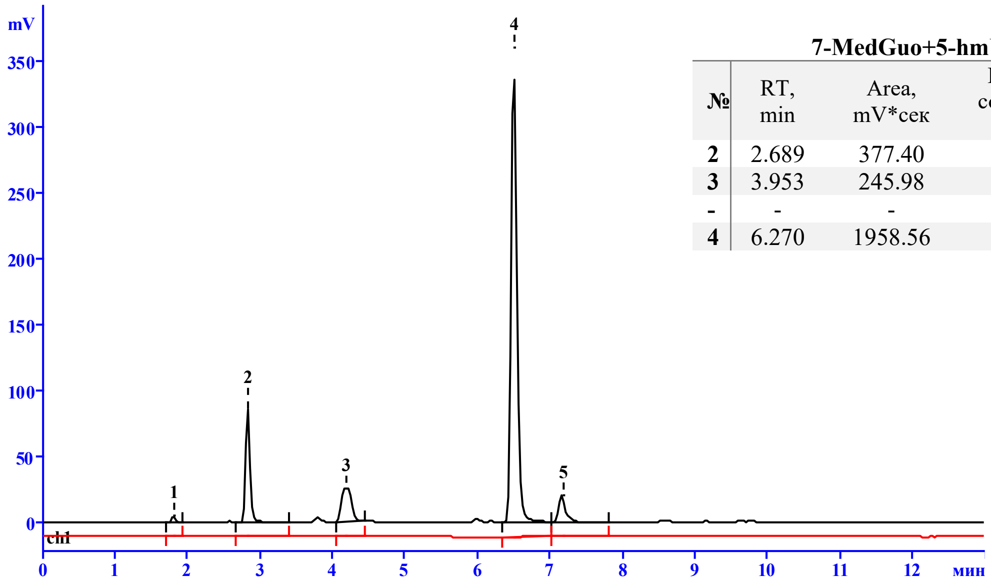

7-MedGuo+5-hmUra – 1:3– t equilibrium

№	RT, min	Area, mV*сек	Equilibrium concentration, µM	Name
**2**	2.689	377.40	100.00	5-hmUra
**3**	3.953	245.98	9.23	7-MeGua
**-**	-	-	-	5-hmdUrd
**4**	6.270	1958.56	290.77	7-MedGuo

3) 13*10^{-3} U *LlNDT II*

7-MedGuo+5-hmUra – 1:3 – t_0

№	RT, min	Area, mV*сек	Equilibrium concentration, µM	Name
**2**	2.542	843.81	100.00	5-hmUra
**4**	5.929	2086.42	300.00	7-MedGuo

t initial

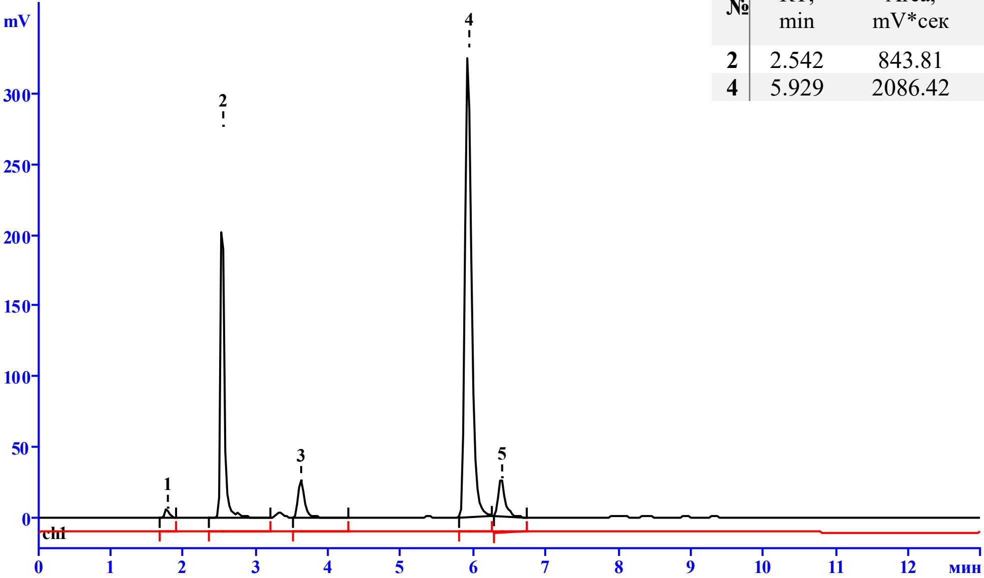

t equilibrium

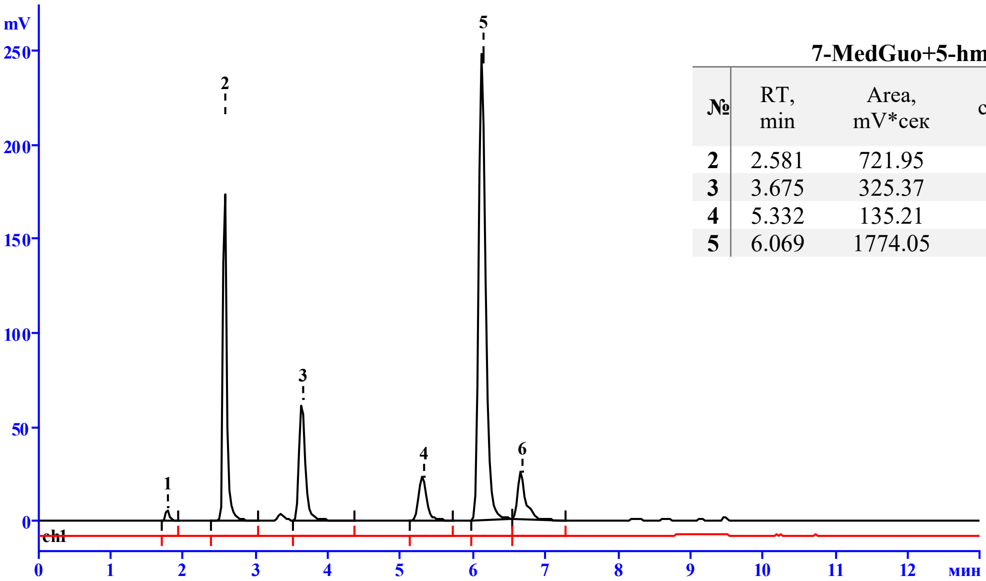

7-MedGuo+5-hmUra – 1:3– t equilibrium

№	RT, min	Area, mV*сек	Equilibrium concentration, µM	Name
**2**	2.581	721.95	85.56	5-hmUra
**3**	3.675	325.37	44.91	7-MeGua
**4**	5.332	135.21	14.44	5-hmdUrd
**5**	6.069	1774.05	255.09	7-MedGuo

4) 26*10^{-3} U *LlNDT II*

tinitial

7-MedGuo+5-hmUra – 1:3 – t_0

№	RT, min	Area, mV*сек	Equilibrium concentration, µM	Name
**2**	2.670	823.27	100.00	5-hmUra
**4**	6.369	2018.53	300.00	7-MedGuo

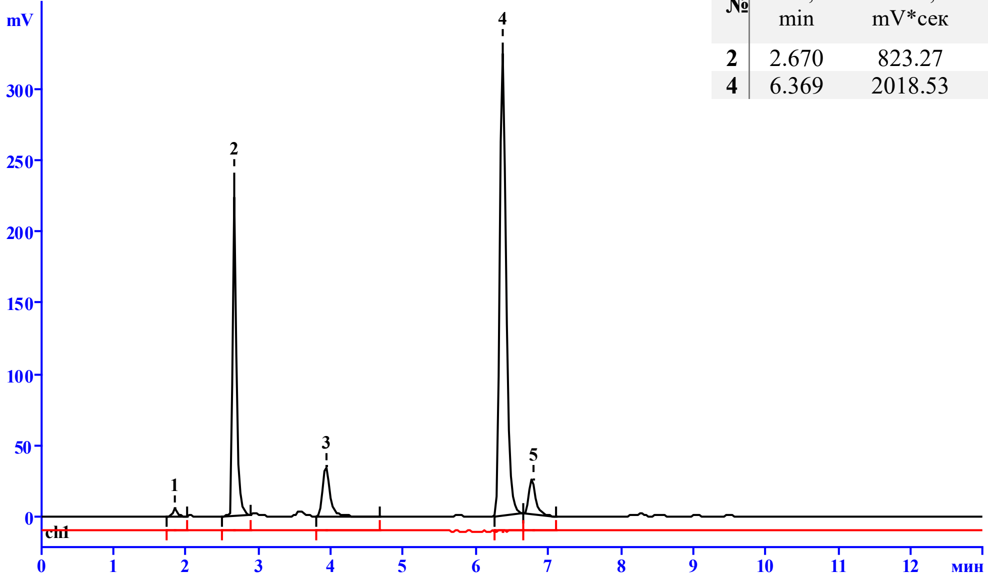

tequilibrium

7-MedGuo+5-hmUra – 1:3– t equilibrium

№	RT, min	Area, mV*сек	Equilibrium concentration, µM	Name
**2**	2.688	106.24	12.90	5-hmUra
**3**	4.020	1509.69	300.00	7-MeGua
**4**	5.632	860.09	87.10	5-hmdUrd
**-**	-	-	-	7-MedGuo

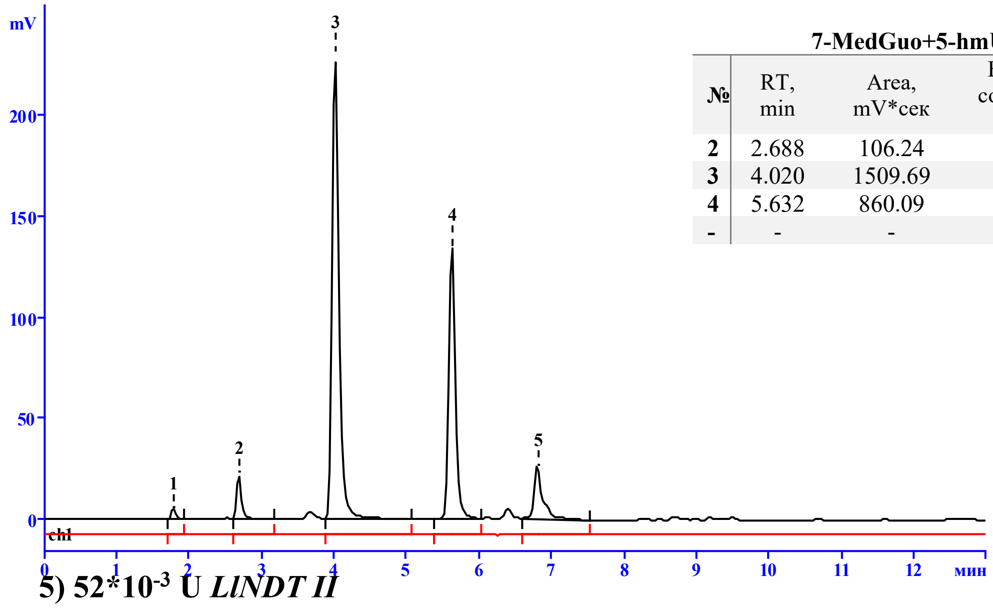

5) 52*10^{-3} U *LlNDT II*

tinitial

7-MedGuo+5-hmUra – 1:3 – t_0

№	RT, min	Area, mV*сек	Equilibrium concentration, µM	Name
**2**	2.652	700.83	100.00	5-hmUra
**4**	6.081	1846.88	300.00	7-MedGuo

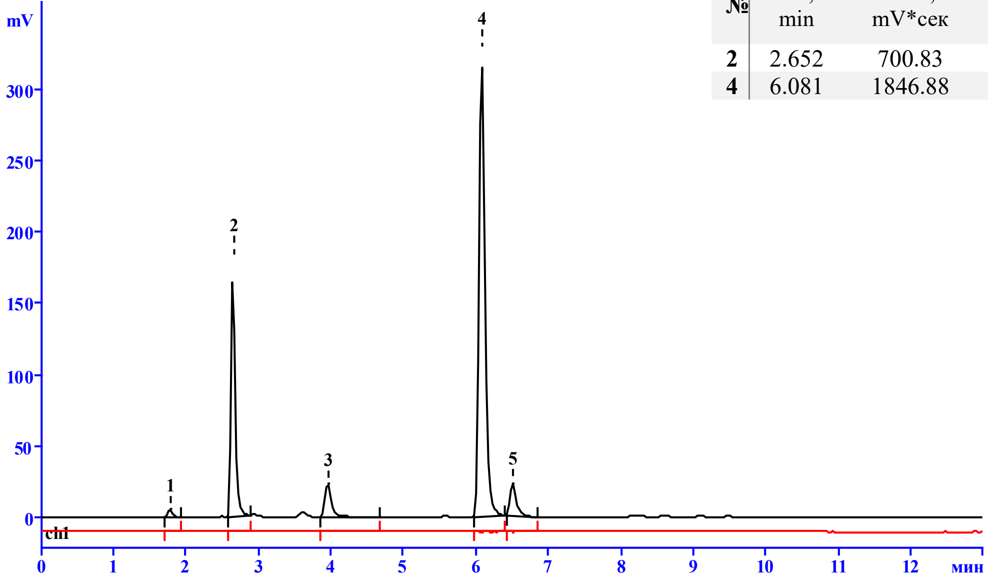

t$_{equilibrium}$

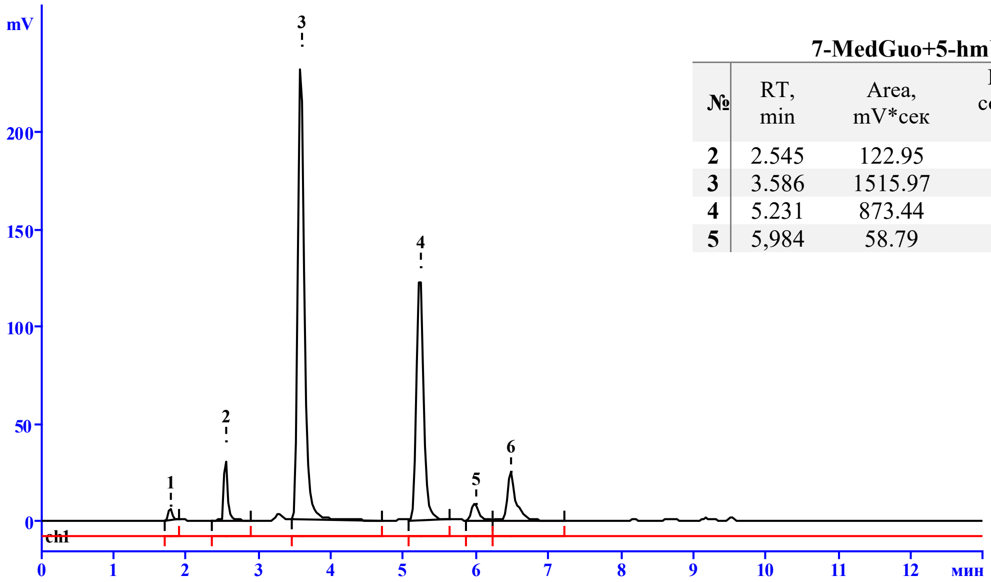

7-MedGuo+5-hmUra – 1:3– t $_{equilibrium}$

№	RT, min	Area, mV*сек	Equilibrium concentration, µM	Name
**2**	2.545	122.95	17.54	5-hmUra
**3**	3.586	1515.97	290.45	7-MeGua
**4**	5.231	873.44	82.46	5-hmdUrd
**5**	5,984	58.79	9.55	7-MedGuo

2΄-deoxy-5-hydroxymethyluridine from thymidine (Thd+5-hmUra, analytical method)

HPLC-analysis of transglycosylation: Cosmosil 5C18-MS-II, 4.6*150 mm, 5µm, 120 Å, (Nacalai Tesque, Inc. (Japan)), 20ºC, elution in modified mobile phase: linear gradient of acetonitrile in 0.06% TFA from 2 to 12% for 10 min (flushing with 12-80 % acetonitrile-0.06% TFA for 10-10.1 min, then 80-2% for 10.1-10.8 min) at flow rate 1 ml/min with UV detection at wavelength 263 nm, injection volume 20 µL.

Fugure S8.1. pH = 5.5

1) 2.6*10^{-3} U *LlNDT II*

Thd+5-hmUra – 1:3 – t$_0$

№	RT, min	Area, mV*сек	Equilibrium concentration, µM	Name
**1**	2.631	887.90	100.00	5-hmUra
**3**	8.376	3463.39	300.00	Thd

t$_{initial}$

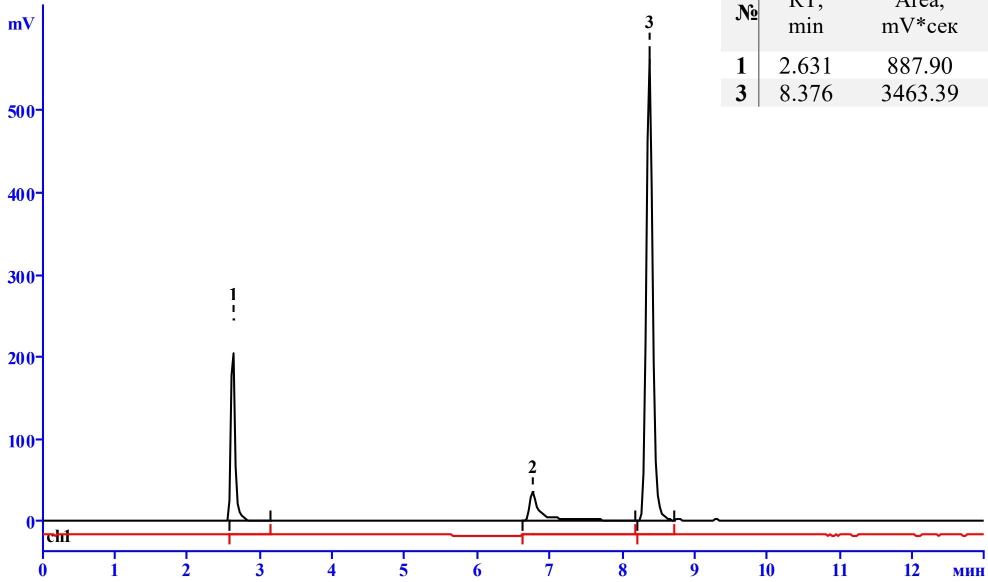

$t_{equilibrium}$

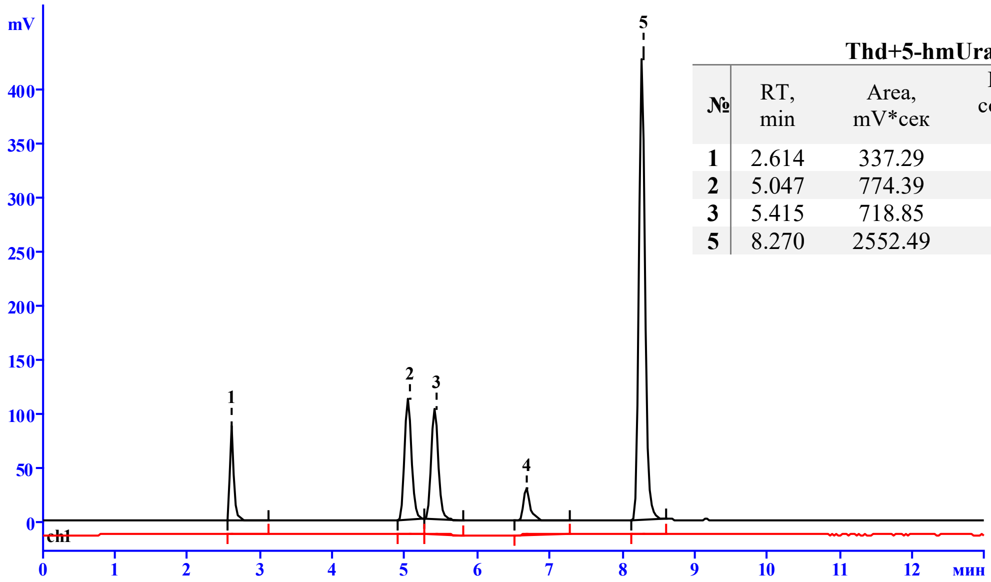

Thd+5-hmUra – 1:3– $t_{equilibrium}$

№	RT, min	Area, mV*сек	Equilibrium concentration, µM	Name
1	2.614	337.29	37.99	5-hmUra
2	5.047	774.39	78.97	Thy
3	5.415	718.85	62.01	5-hmdUrd
5	8.270	2552.49	221.03	Thd

2) 7.8*10^{-3} U *LlNDT II*

$t_{initial}$

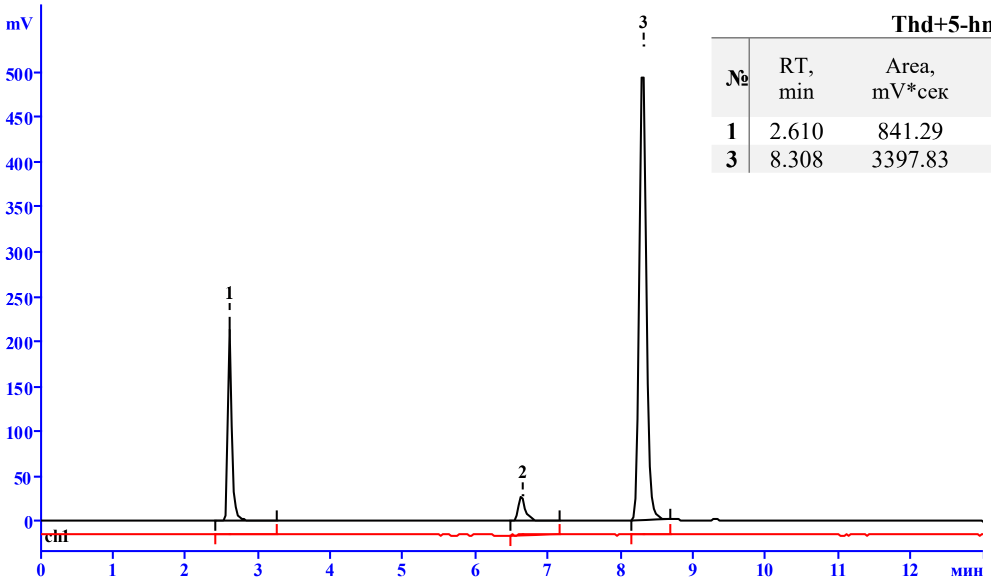

Thd+5-hmUra – 1:3 – t_0

№	RT, min	Area, mV*сек	Equilibrium concentration, µM	Name
1	2.610	841.29	100.00	5-hmUra
3	8.308	3397.83	300.00	Thd

$t_{equilibrium}$

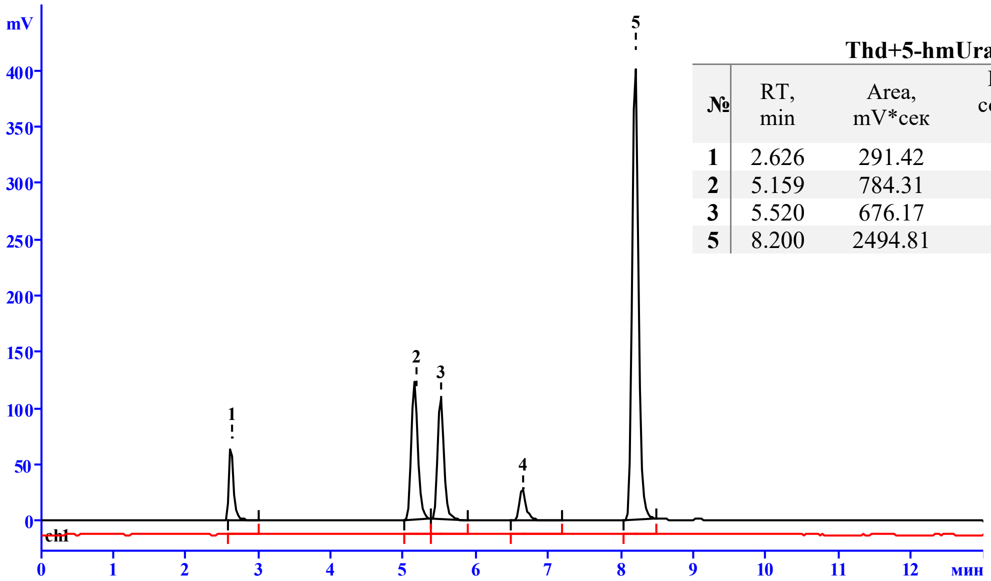

Thd+5-hmUra – 1:3– $t_{equilibrium}$

№	RT, min	Area, mV*сек	Equilibrium concentration, µM	Name
1	2.626	291.42	34.64	5-hmUra
2	5.159	784.31	79.73	Thy
3	5.520	676.17	65.36	5-hmdUrd
5	8.200	2494.81	220.27	Thd

Fugure S8.2. pH = 7.0

1) 2.6*10^{-3} U *LlNDT II*

t$_{initial}$

Thd+5-hmUra – 1:3 – t$_0$

№	RT, min	Area, mV*сек	Equilibrium concentration, µM	Name
**1**	2.589	876.34	100.00	5-hmUra
**3**	8.302	3577.31	300.00	Thd

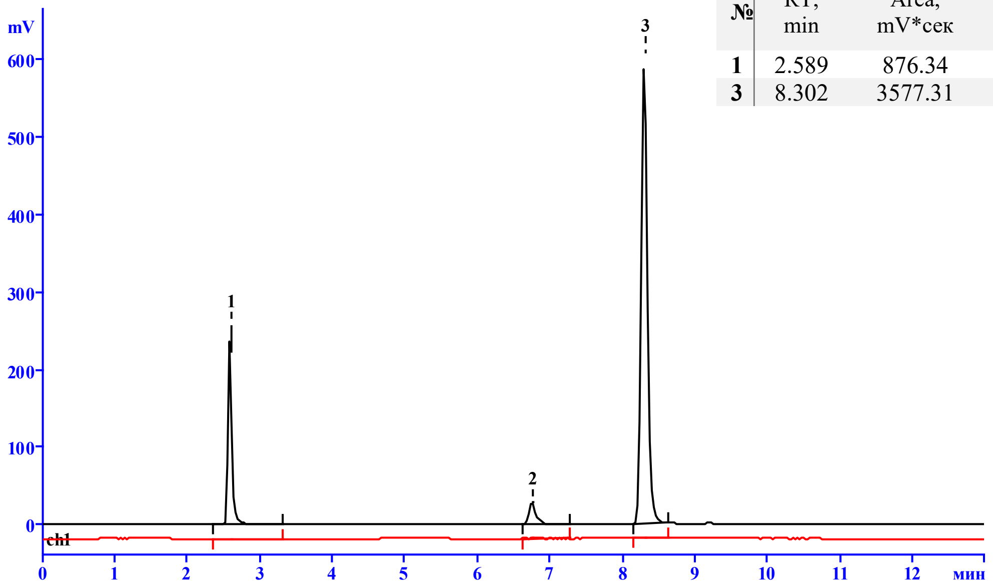

t$_{equilibrium}$

Thd+5-hmUra – 1:3– t $_{equilibrium}$

№	RT, min	Area, mV*сек	Equilibrium concentration, µM	Name
**1**	2.572	766.35	87.45	5-hmUra
**2**	5.086	180.56	24.23	Thy
**3**	5.450	210.09	12.55	5-hmdUrd
**5**	8.270	3288.37	275.77	Thd

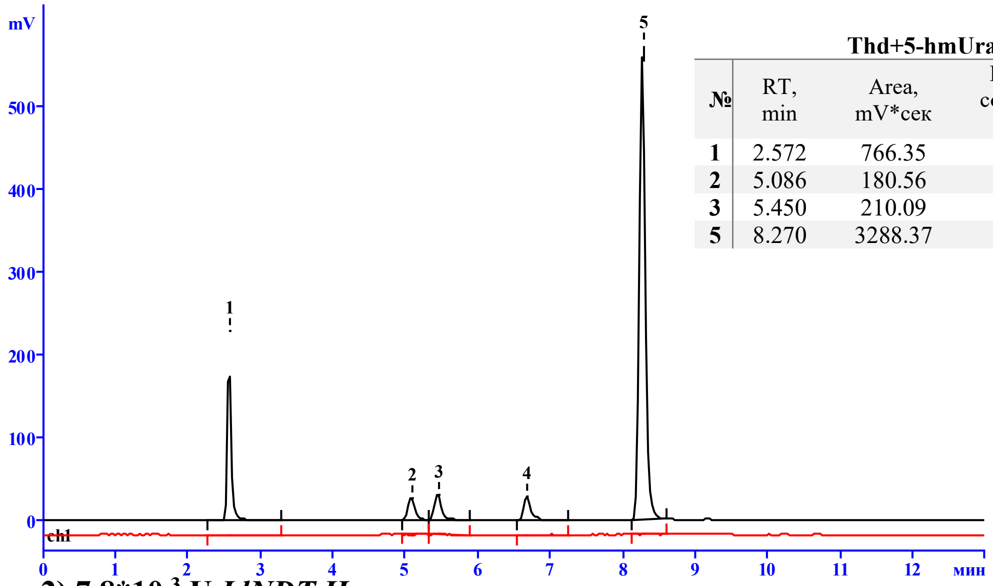

2) 7.8*10^{-3} U *LlNDT II*

t$_{initial}$

Thd+5-hmUra – 1:3 – t$_0$

№	RT, min	Area, mV*сек	Equilibrium concentration, µM	Name
**1**	2.674	550.79	100.00	5-hmUra
**3**	8.935	3464.60	300.00	Thd

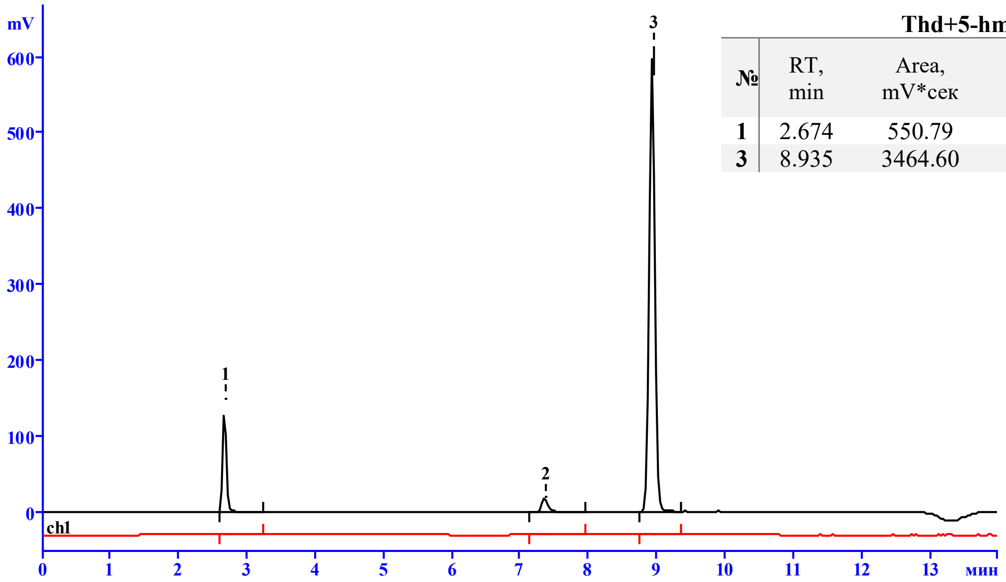

t$_{equilibrium}$

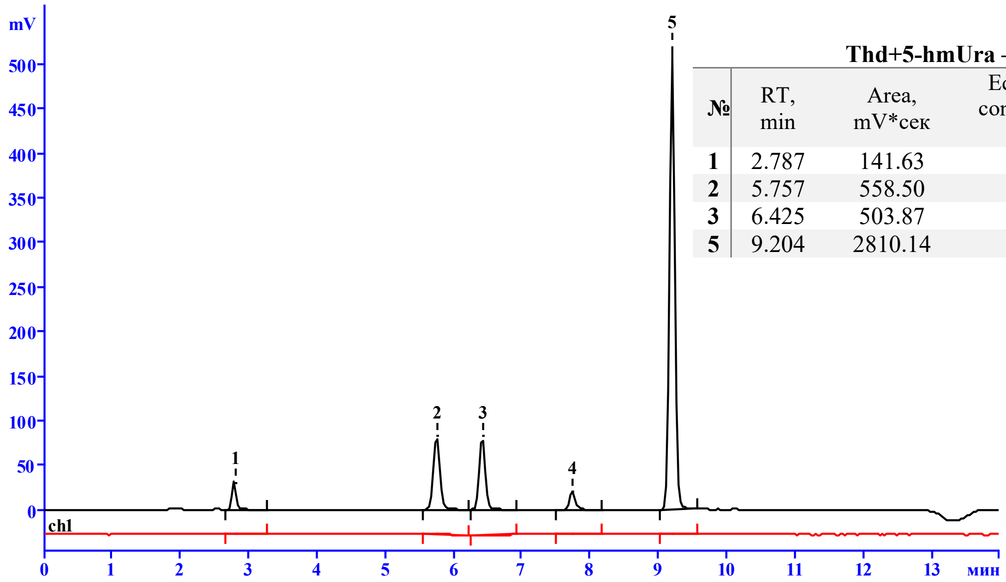

Thd+5-hmUra – 1:3– t $_{equilibrium}$

№	RT, min	Area, mV*сек	Equilibrium concentration, µM	Name
1	2.787	141.63	25.71	5-hmUra
2	5.757	558.50	56.67	Thy
3	6.425	503.87	74.29	5-hmdUrd
5	9.204	2810.14	243.33	Thd

Fugure S8.3. pH = 8.5

1) 2.6*10^{-3} U *LlNDT II*

t$_{initial}$

Thd+5-hmUra – 1:3 – t$_0$

№	RT, min	Area, mV*сек	Equilibrium concentration, µM	Name
1	2.621	876.34	100.00	5-hmUra
3	8.396	3577.31	300.00	Thd

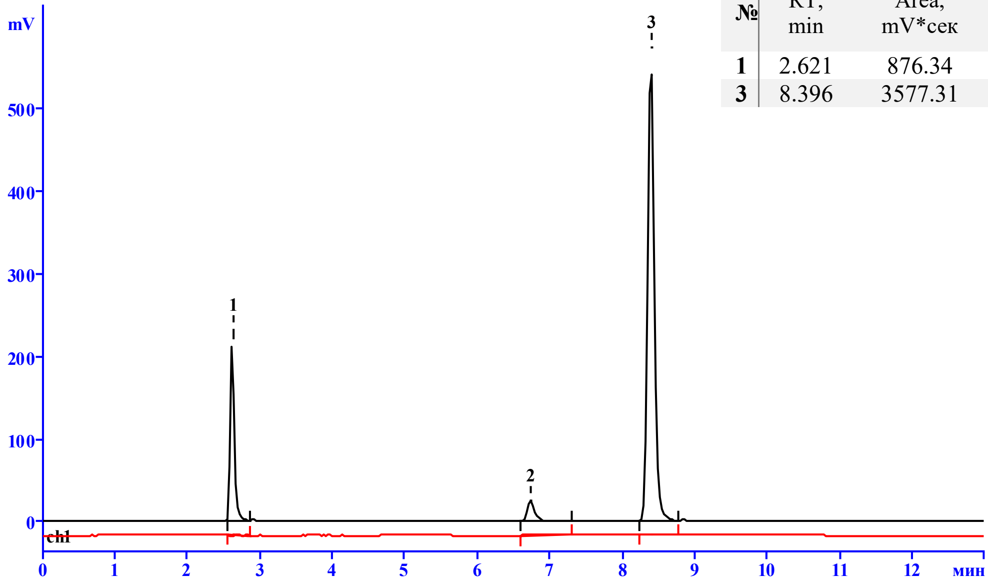

t$_{equilibrium}$

Thd+5-hmUra – 1:3– t $_{equilibrium}$

№	RT, min	Area, mV*сек	Equilibrium concentration, µM	Name
1	2.568	943.83	100.00	5-hmUra
-	-	-	-	Thy
-	-	-	-	5-hmdUrd
3	8.286	3558.42	300.00	Thd

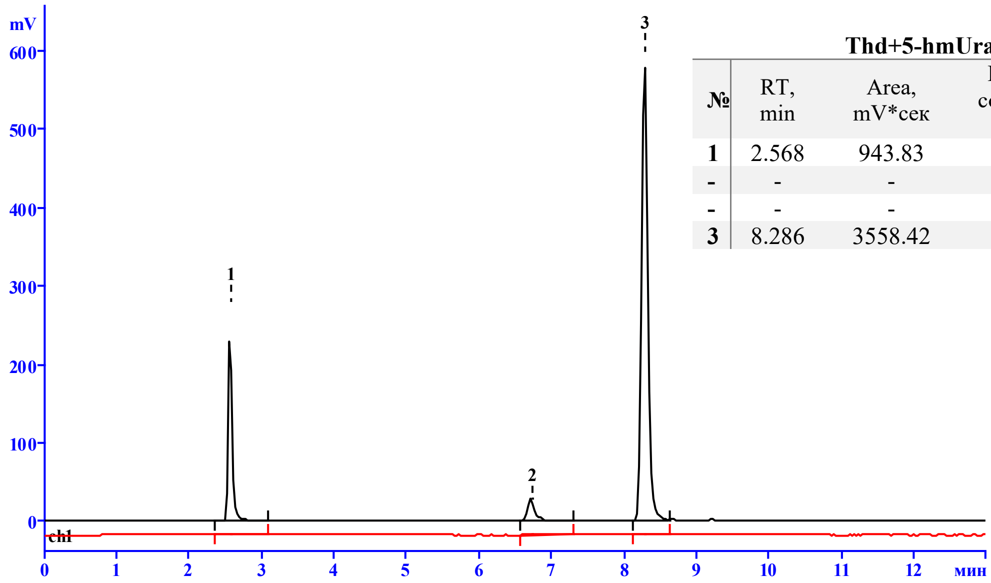

2) 7.8*10^{-3} U *LlNDT II*

$t_{initial}$

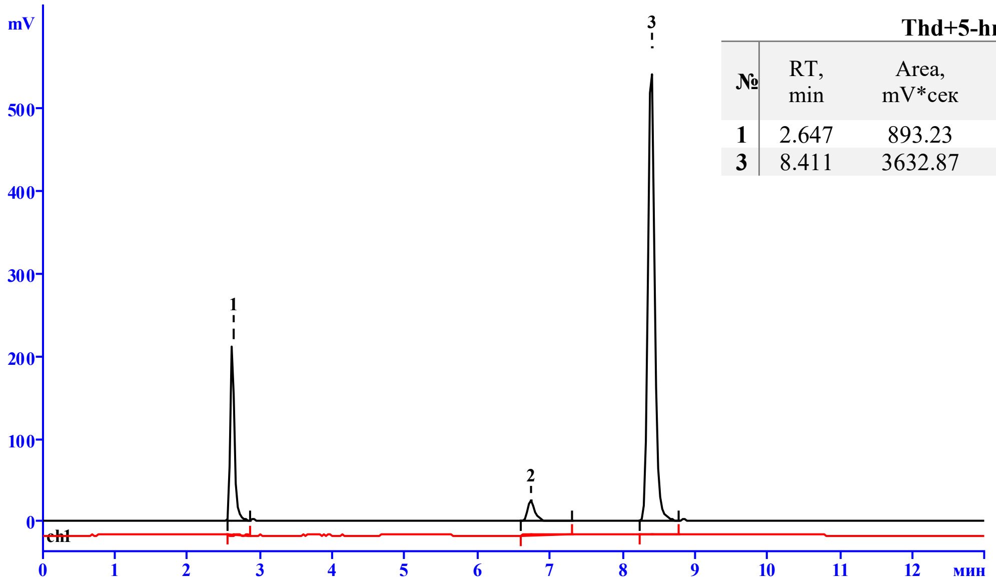

Thd+5-hmUra – 1:3 – t$_0$

№	RT, min	Area, mV*сек	Equilibrium concentration, µM	Name
**1**	2.647	893.23	100.00	5-hmUra
**3**	8.411	3632.87	300.00	Thd

$t_{equilibrium}$

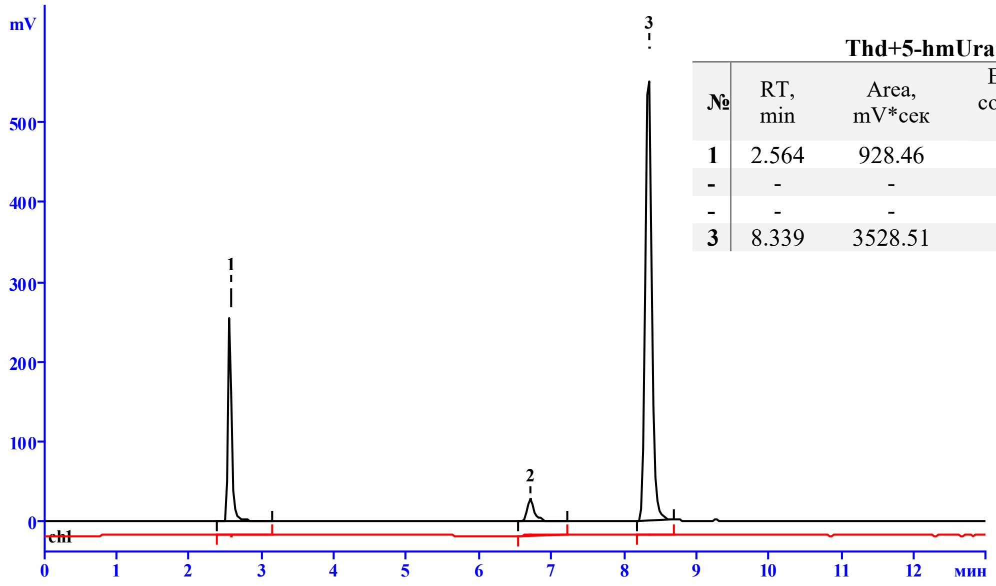

Thd+5-hmUra – 1:3– t $_{equilibrium}$

№	RT, min	Area, mV*сек	Equilibrium concentration, µM	Name
**1**	2.564	928.46	100.00	5-hmUra
**-**	-	-	-	Thy
**-**	-	-	-	5-hmdUrd
**3**	8.339	3528.51	300.00	Thd

2´-deoxycytidine from 7-methyldeoxyguanosine (7-MedGuo+Cyt, analytical method)

HPLC-analysis of transglycosylation: Cosmosil 5C18-MS-II, 4.6*150 mm, 5µm, 120 Å, (Nacalai Tesque, Inc. (Japan)), 20ºC, elution in modified mobile phase: linear gradient of acetonitrile in 10 mM NaOAc from 2 to 12% for 10 min (flushing with 12-80 % acetonitrile-10 mM NaOAc for 10-10.1 min, then 80-2% for 10.1-10.8 min) at flow rate 1 ml/min with UV detection at wavelength 266 nm, injection volume 20 µL.

Fugure S9.1. pH = 5.5, 7.8*10^{-3} U *LlNDT II*

t$_{initial}$

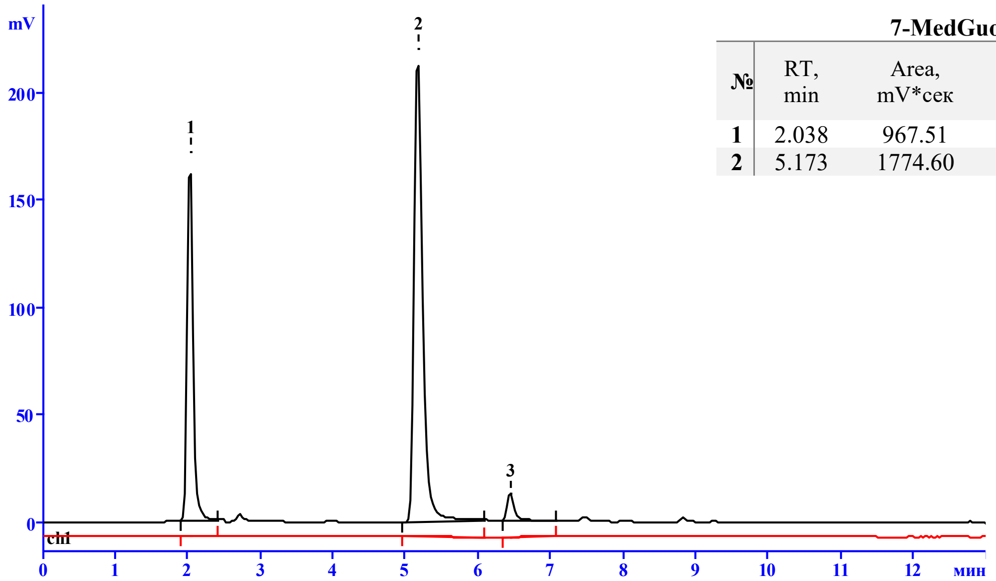

7-MedGuo+Cyt – 3:1 – t$_0$

№	RT, min	Area, mV*сек	Equilibrium concentration, µM	Name
**1**	2.038	967.51	100.00	Cyt
**2**	5.173	1774.60	300.00	7-MedGuo

t$_{equilibrium}$

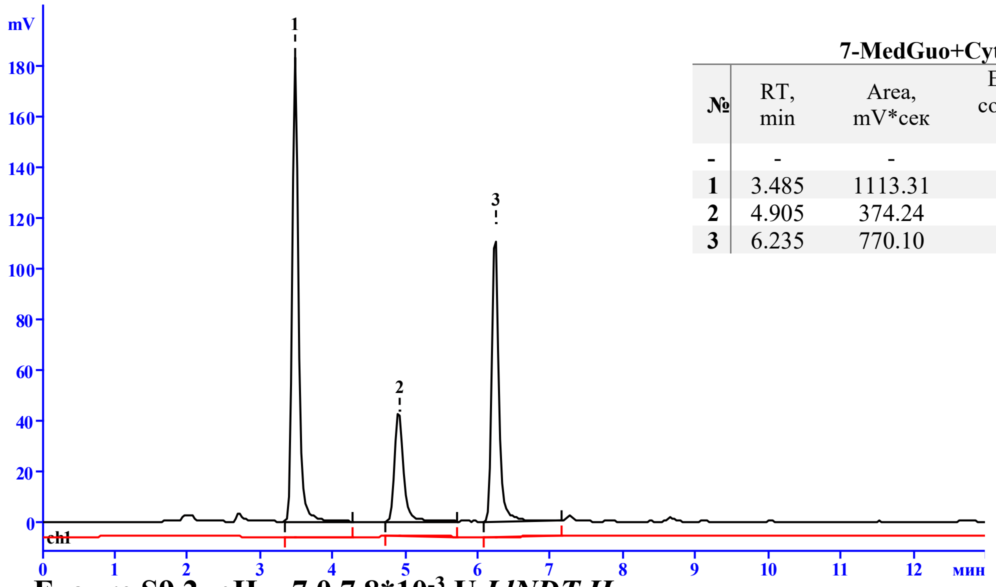

7-MedGuo+Cyt – 3:1– t $_{equilibrium}$

№	RT, min	Area, mV*сек	Equilibrium concentration, µM	Name
**-**	-	-	-	Cyt
**1**	3.485	1113.31	100.00	dCyd
**2**	4.905	374.24	63.27	7-MedGuo
**3**	6.235	770.10	236.73	7-MeGua

Fugure S9.2. pH = 7.0,7.8*10^{-3} U *LlNDT II*

7-MedGuo+Cyt – 3:1 – t$_0$

№	RT, min	Area, mV*сек	Equilibrium concentration, µM	Name
**1**	2.065	809.46	100.00	Cyt
**3**	5.361	3440.93	300.00	7-MedGuo

t$_{initial}$

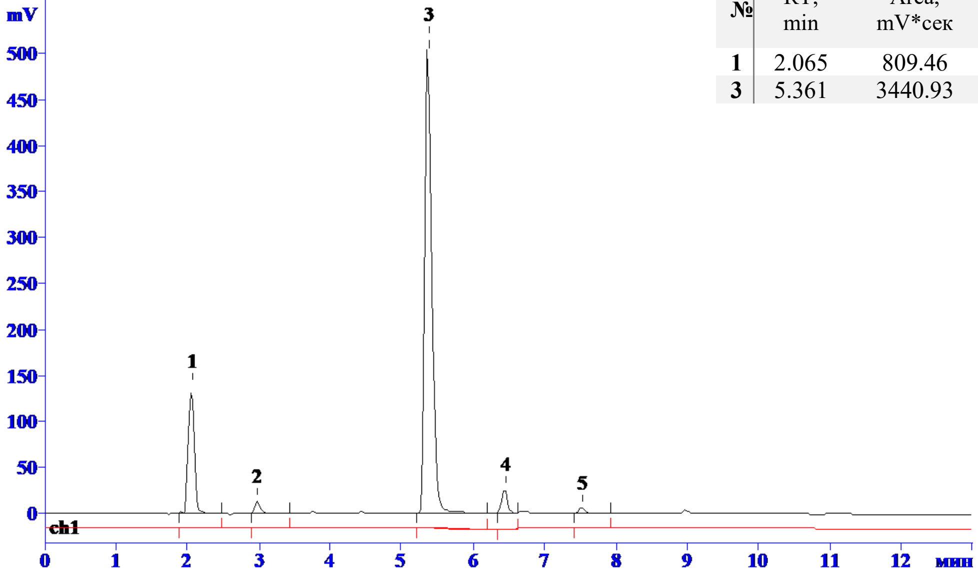

$t_{equilibrium}$

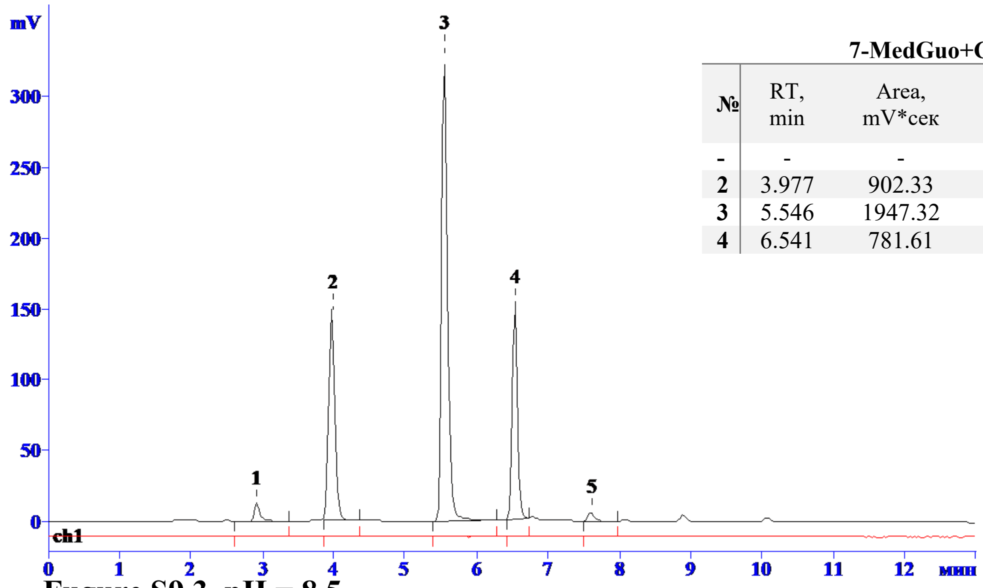

7-MedGuo+Cyt – 3:1– $t_{equilibrium}$

№	RT, min	Area, mV*сек	Equilibrium concentration, μM	Name
-	-	-	-	Cyt
2	3.977	902.33	100.00	dCyd
3	5.546	1947.32	169.78	7-MedGuo
4	6.541	781.61	130.22	7-MeGua

Fugure S9.3. pH = 8.5

1) 7.8*10^{-3} U *LlNDT II*

7-MedGuo+Cyt – 3:1 – t_0

№	RT, min	Area, mV*сек	Equilibrium concentration, μM	Name
1	2.148	867.22	100.00	Cyt
2	5.886	1665.93	300.00	7-MedGuo

$t_{initial}$

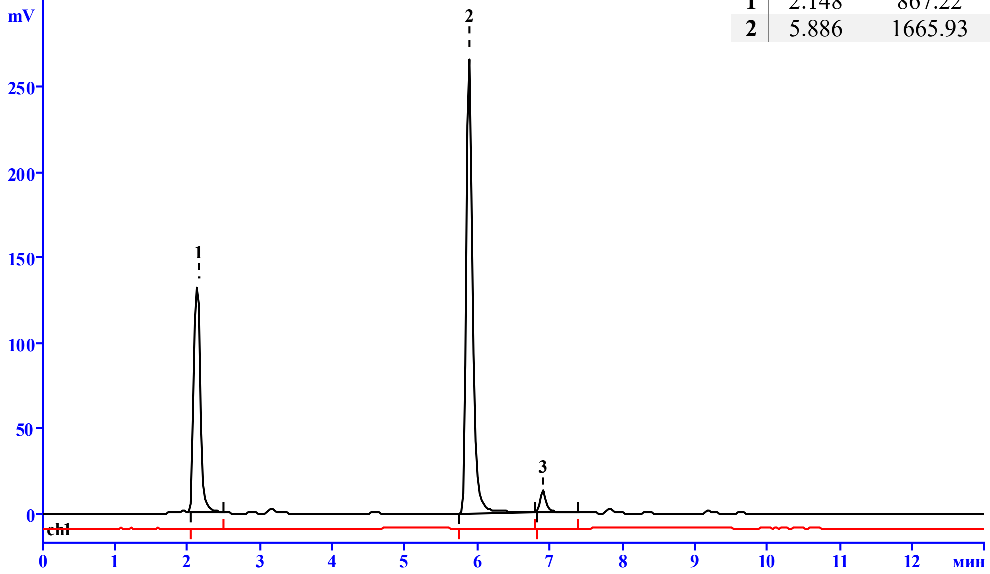

$t_{equilibrium}$

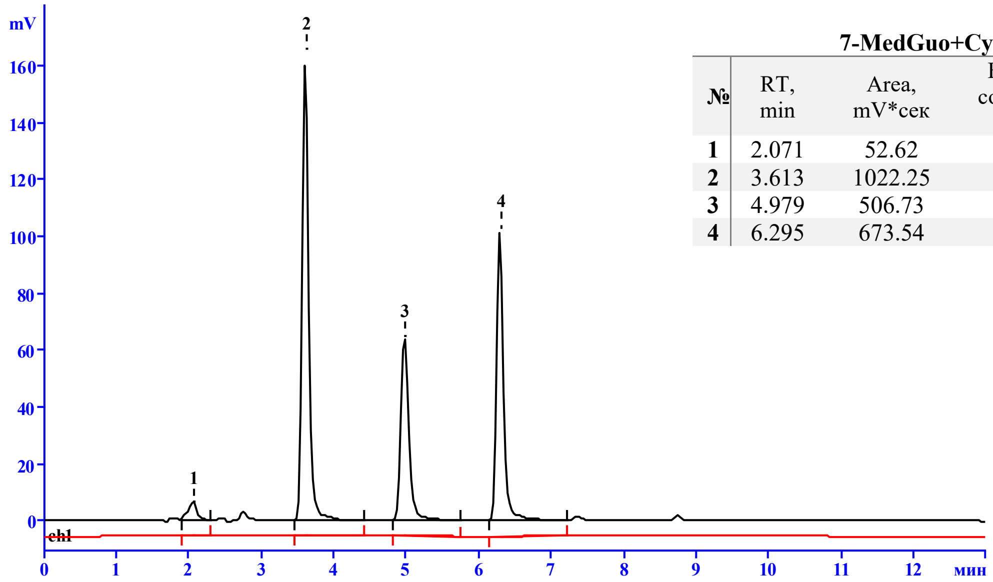

7-MedGuo+Cyt – 3:1– $t_{equilibrium}$

№	RT, min	Area, mV*сек	Equilibrium concentration, μM	Name
1	2.071	52.62	6.07	Cyt
2	3.613	1022.25	93.93	dCyd
3	4.979	506.73	91.25	7-MedGuo
4	6.295	673.54	208.75	7-MeGua

2) 26*10^{-3} U *LlNDT II*

$t_{initial}$

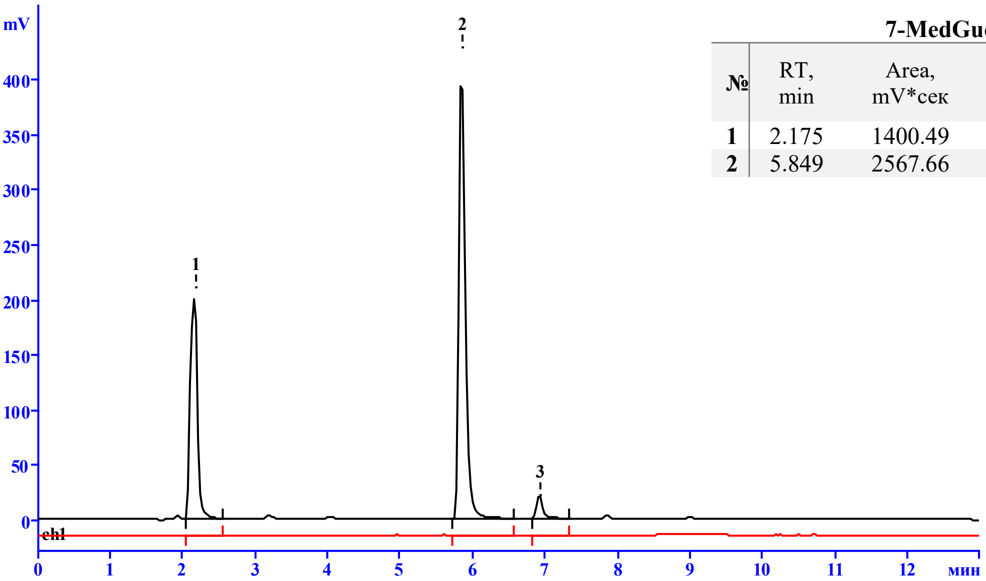

7-MedGuo+Cyt – 3:1 – t_0

№	RT, min	Area, mV*сек	Equilibrium concentration, μM	Name
**1**	2.175	1400.49	100.00	Cyt
**2**	5.849	2567.66	300.00	7-MedGuo

$t_{equilibrium}$

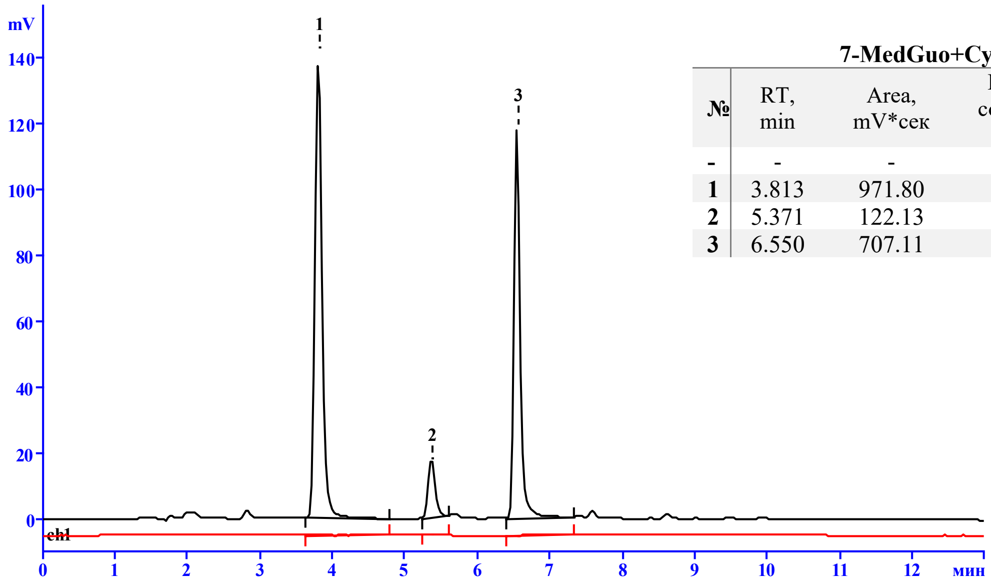

7-MedGuo+Cyt – 3:1– $t_{equilibrium}$

№	RT, min	Area, mV*сек	Equilibrium concentration, μM	Name
**-**	-	-	-	Cyt
**1**	3.813	971.80	100.00	dCyd
**2**	5.371	122.13	14.27	7-MedGuo
**3**	6.550	707.11	285.73	7-MeGua

2′-deoxyuridine, 2′-deoxthymidine, 5-vinyl-2'-deoxyuridine, deoxyinosine from 7-methyldeoxyguanosine (7-MedGuo+Ura, Thy, 5-VinUra, Hyp, analytical method)

HPLC-analysis of transglycosylation: Cosmosil 5C18-MS-II, 4.6*150 mm, 5μm, 120 Å, (Nacalai Tesque, Inc. (Japan)), 20ºC, elution in modified mobile phase: linear gradient of acetonitrile in 0.06% TFA from 2 to 12% for 10 min (flushing with 12-80 % acetonitrile-0.06% TFA for 10-10.1 min, then 80-2% for 10.1-10.8 min) at flow rate 1 ml/min with UV detection at wavelength 260 nm for Ura, Thy and Hyp, 281 nm for 5-VinUra, injection volume 20 μL.

Fugure S10.1. pH = 5.5, 7.8*10^{-3} U *LlNDT II*

a) $t_{initial}$

7-MedGuo+Ura – 3:1 – t_0

№	RT, min	Area, mV*сек	Equilibrium concentration, µM	Name
**2**	2.741	952.10	100.00	Ura
**4**	6.587	2320.35	300.00	7-MedGuo

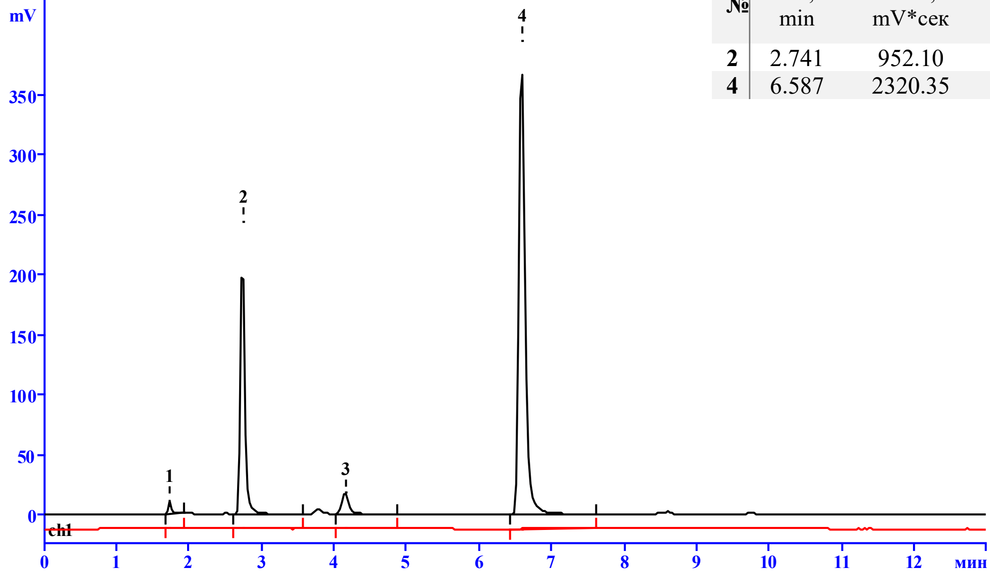

$t_{equilibrium}$

7-MedGuo+Ura – 3:1– t $_{equilibrium}$

№	RT, min	Area, mV*сек	Equilibrium concentration, µM	Name
**-**	-	-	-	Ura
**2**	4.103	1217.10	100.00	dUrd
**3**	5.758	1150.10	206.12	7-MeGua
**4**	6.580	726.08	93.88	7-MedGuo

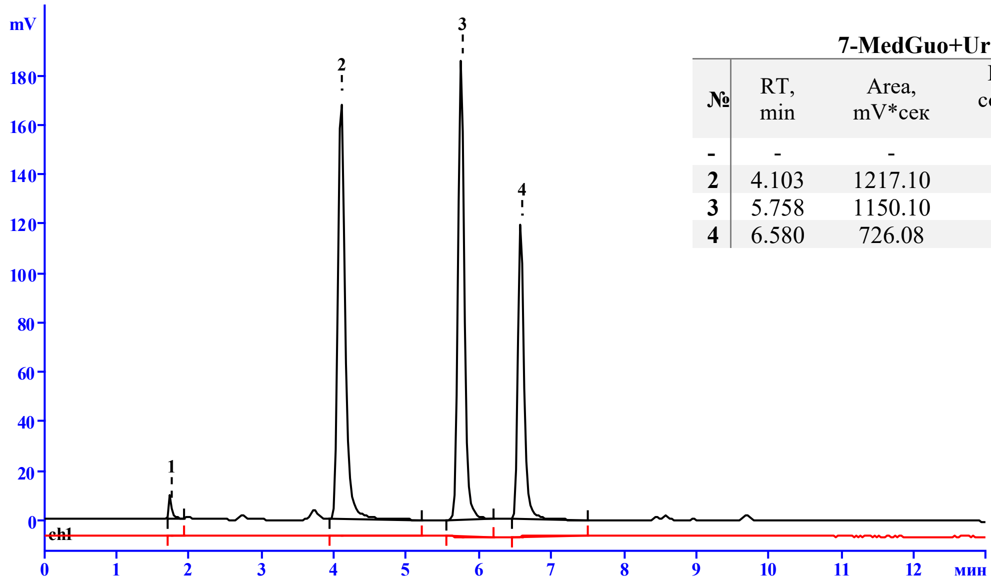

b) $t_{initial}$

7-MedGuo+Thy – 3:1 – t_0

№	RT, min	Area, mV*сек	Equilibrium concentration, µM	Name
**4**	5.297	1107.10	100.00	Thy
**5**	6.351	2837.73	300.00	7-MedGuo

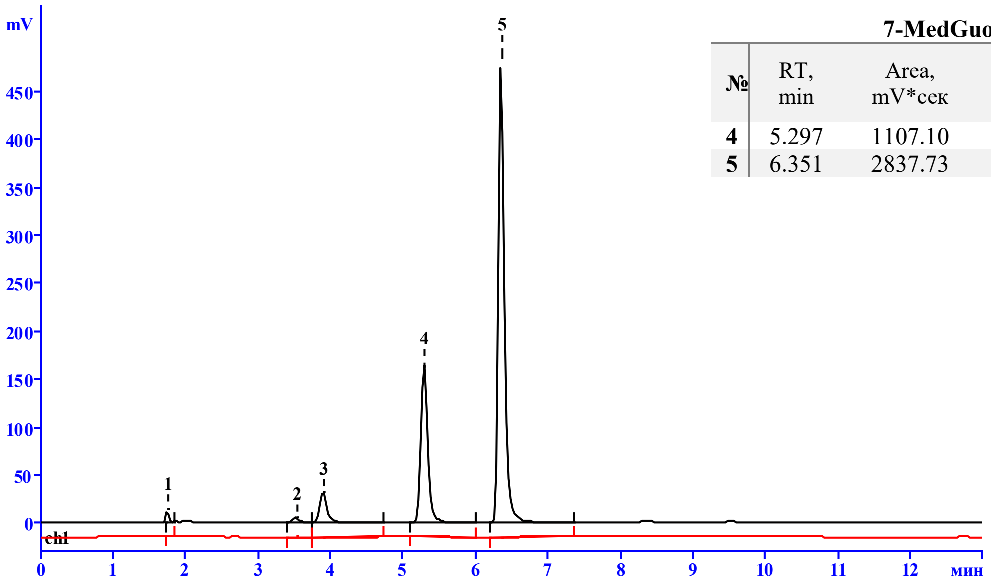

$t_{equilibrium}$

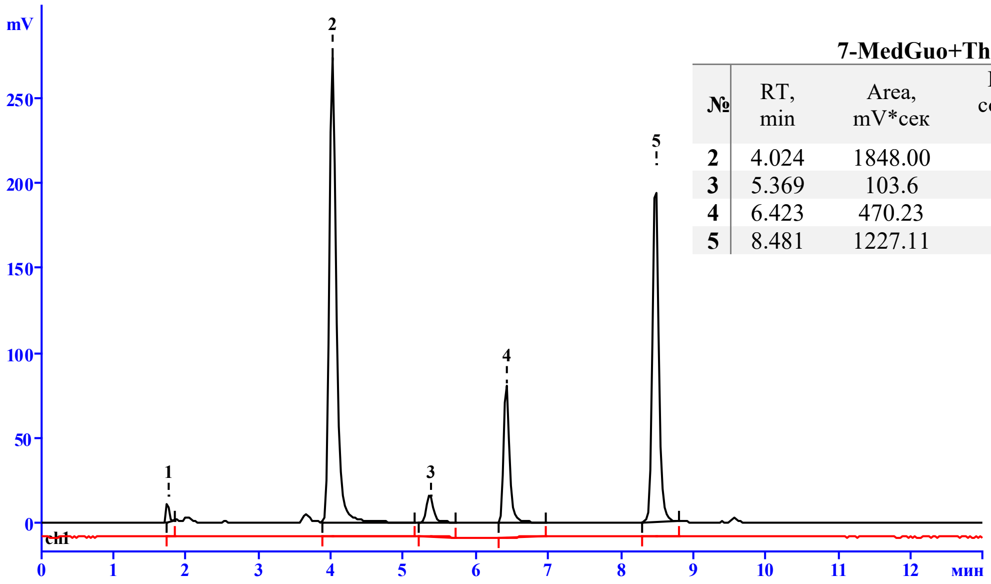

7-MedGuo+Thy – 3:1– $t_{equilibrium}$

№	RT, min	Area, mV*сек	Equilibrium concentration, µM	Name
2	4.024	1848.00	250.29	7-MeGua
3	5.369	103.6	9.36	Thy
4	6.423	470.23	49.71	7-MedGuo
5	8.481	1227.11	90.64	dThd

c) $t_{initial}$

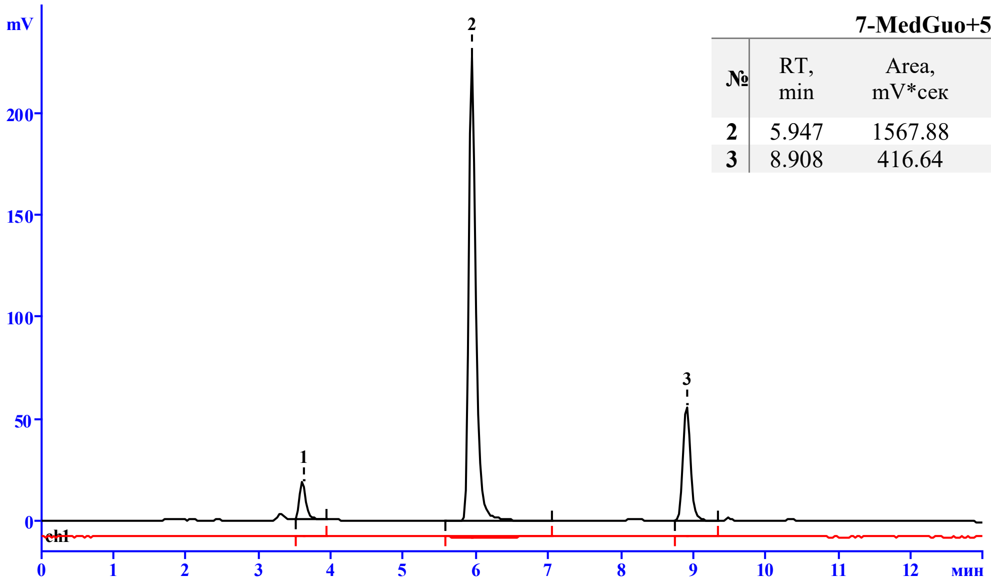

7-MedGuo+5-VinUra – 3:1 – t_0

№	RT, min	Area, mV*сек	Equilibrium concentration, µM	Name
2	5.947	1567.88	300.00	7-MedGuo
3	8.908	416.64	100.00	5-VinUra

$t_{equilibrium}$

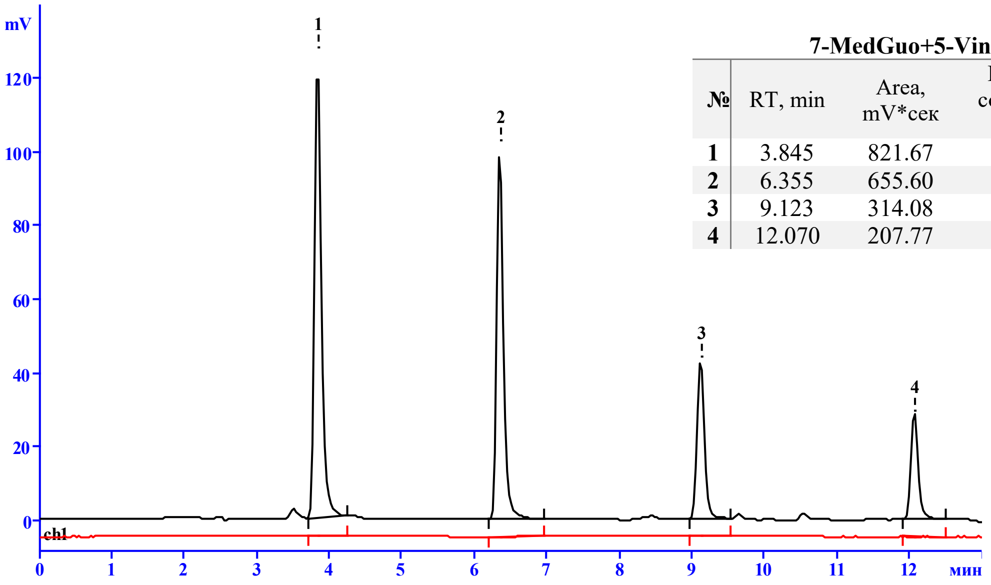

7-MedGuo+5-VinUra – 3:1– $t_{equilibrium}$

№	RT, min	Area, mV*сек	Equilibrium concentration, µM	Name
1	3.845	821.67	230.69	7-MeGua
2	6.355	655.60	69.31	7-MedGuo
3	9.123	314.08	75.38	5-VinUra
4	12.070	207.77	24.62	5-VindUrd

d) $t_{initial}$

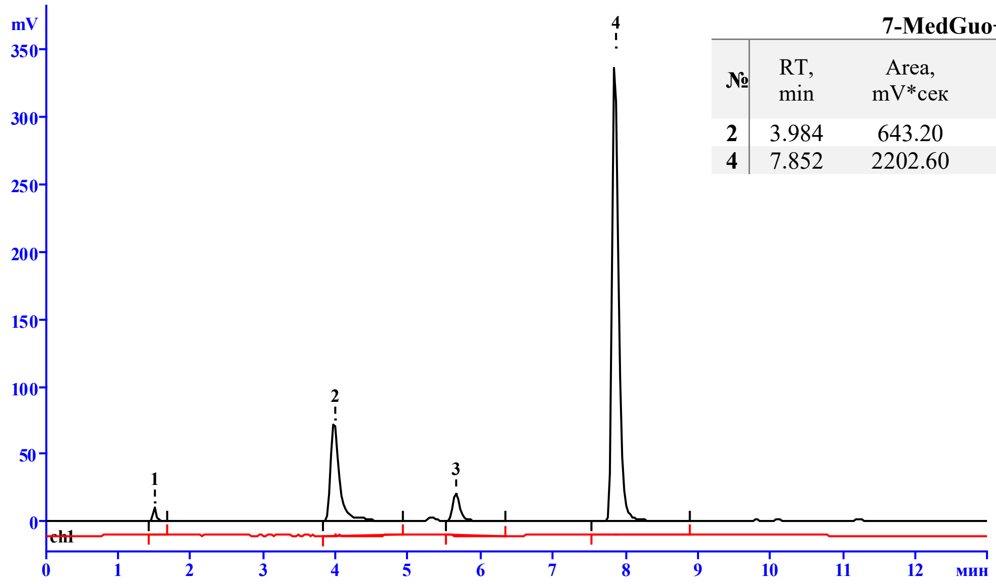

7-MedGuo+Hyp – 3:1 – t_0

№	RT, min	Area, mV*сек	Equilibrium concentration, µM	Name
**2**	3.984	643.20	100.00	Hyp
**4**	7.852	2202.60	300.00	7-MedGuo

$t_{equilibrium}$

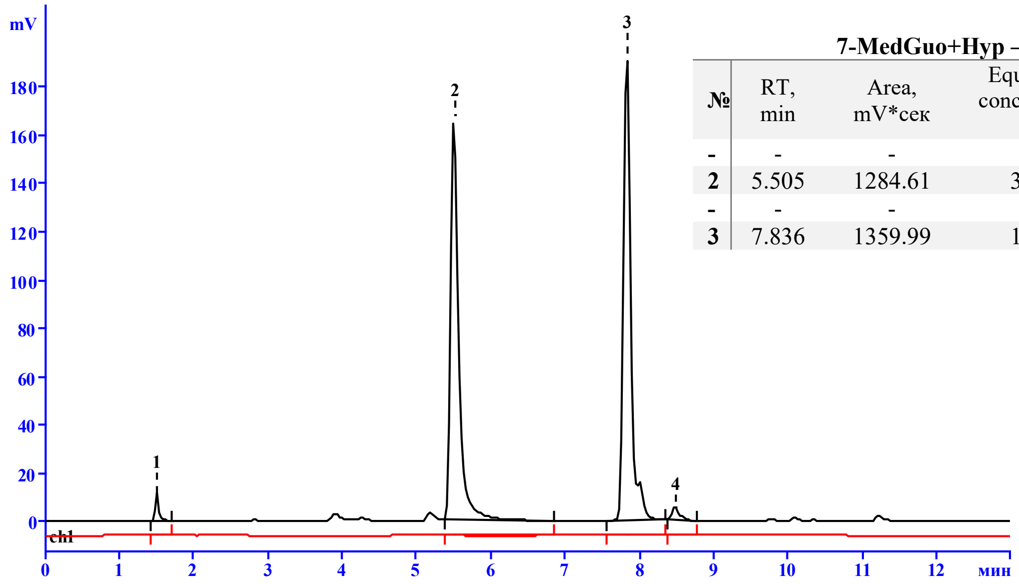

7-MedGuo+Hyp – 3:1– t equilibrium

№	RT, min	Area, mV*сек	Equilibrium concentration, µM	Name
**-**	-	-	-	Hyp
**2**	5.505	1284.61	300.00	7-MeGua
**-**	-	-	-	7-MedGuo
**3**	7.836	1359.99	100.00	Ino

Fugure S10.2. pH = 7.0, 7.8*10^{-3} U *LlNDT II*

7-MedGuo+Ura – 3:1 – t_0

№	RT, min	Area, mV*сек	Equilibrium concentration, µM	Name
**3**	2.675	946.25	100.00	Ura
**5**	6.408	2164.30	300.00	7-MedGuo

a) $t_{initial}$

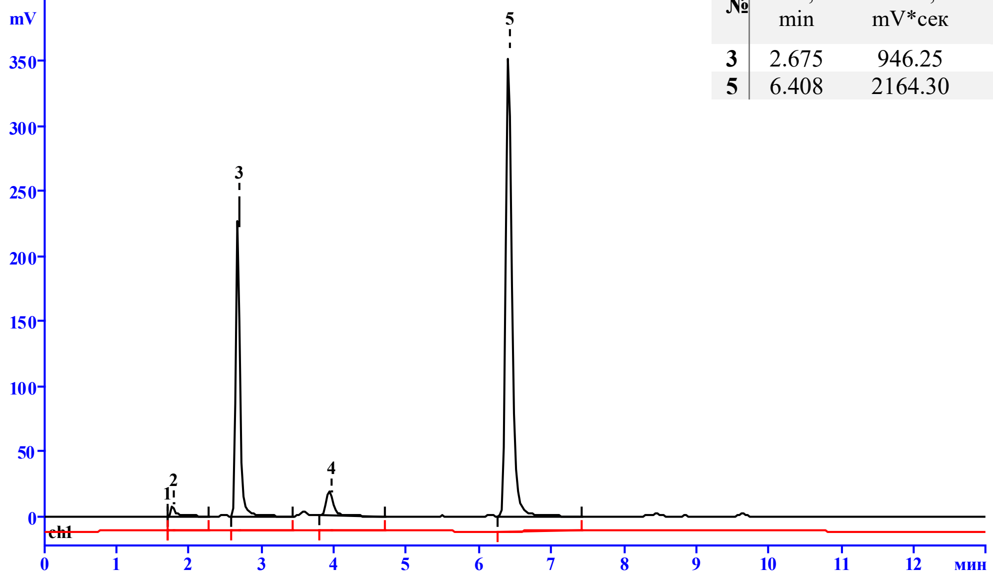

tequilibrium

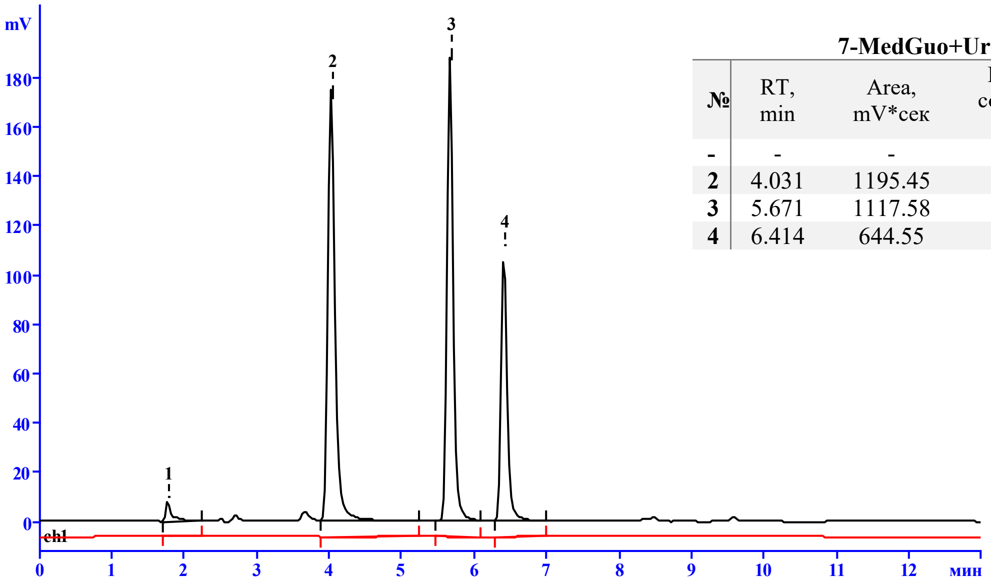

7-MedGuo+Ura – 3:1– t equilibrium

№	RT, min	Area, mV*сек	Equilibrium concentration, µM	Name
-	-	-	-	Ura
2	4.031	1195.45	100.00	dUrd
3	5.671	1117.58	210.66	7-MeGua
4	6.414	644.55	89.34	7-MedGuo

b) tinitial

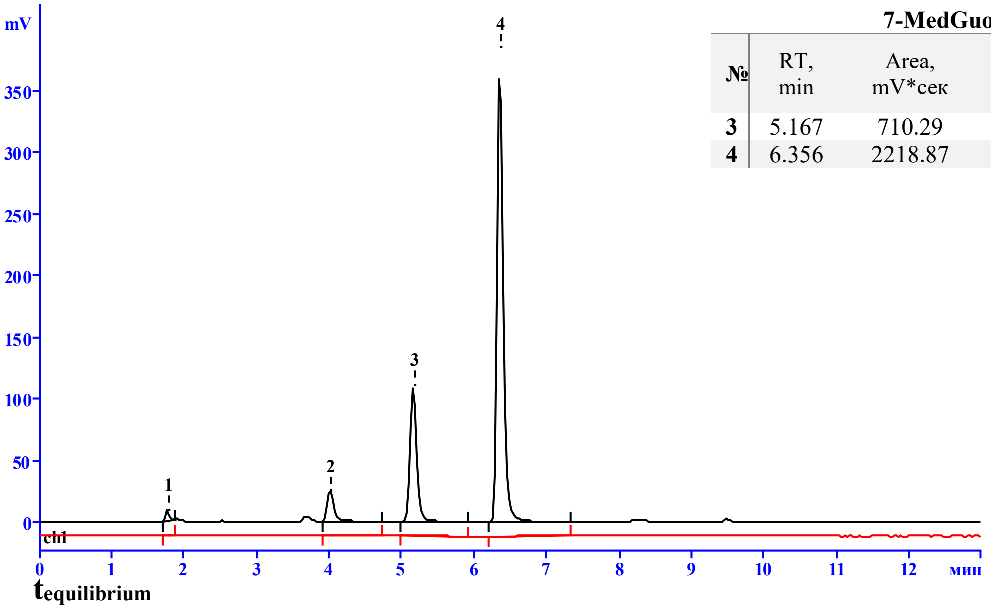

7-MedGuo+Thy – 3:1 – t_0

№	RT, min	Area, mV*сек	Equilibrium concentration, µM	Name
3	5.167	710.29	100.00	Thy
4	6.356	2218.87	300.00	7-MedGuo

tequilibrium

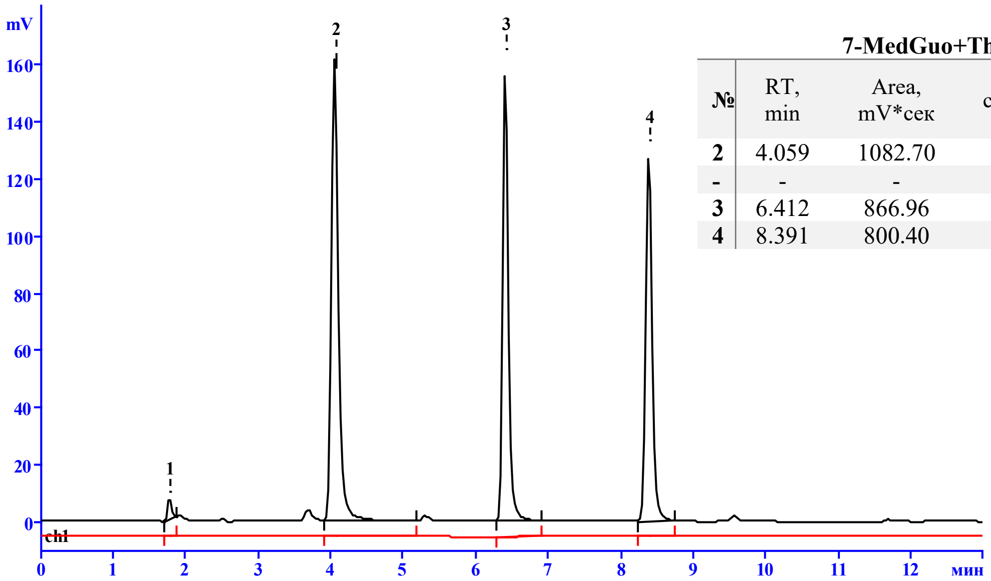

7-MedGuo+Thy – 3:1– t equilibrium

№	RT, min	Area, mV*сек	Equilibrium concentration, µM	Name
2	4.059	1082.70	182.78	7-MeGua
-	-	-	-	Thy
3	6.412	866.96	117.22	7-MedGuo
4	8.391	800.40	100.00	dThd

c) $t_{initial}$

7-MedGuo+5-VinUra – 3:1 – t_0

№	RT, min	Area, mV*сек	Equilibrium concentration, µM	Name
**2**	6.411	1556.30	300.00	7-MedGuo
**3**	9.125	445.63	100.00	5-VinUra

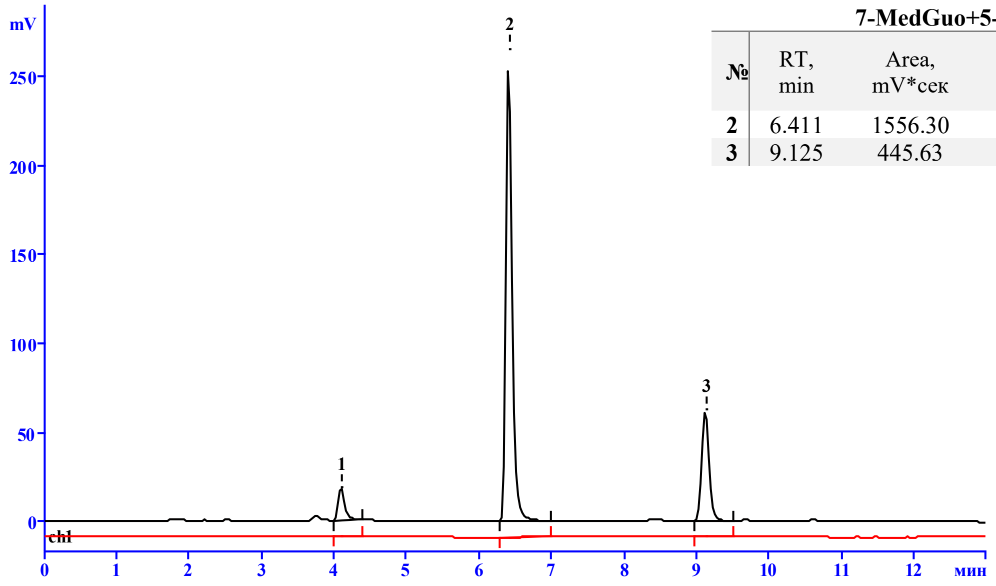

$t_{equilibrium}$

7-MedGuo+5-VinUra – 3:1– $t_{equilibrium}$

№	RT, min	Area, mV*сек	Equilibrium concentration, µM	Name
**1**	3.845	1087.41	300.00	7-MeGua
**-**	-	-	-	7-MedGuo
**2**	9.066	184.74	41.46	5-VinUra
**3**	12.090	241.46	58.54	5-VindUrd

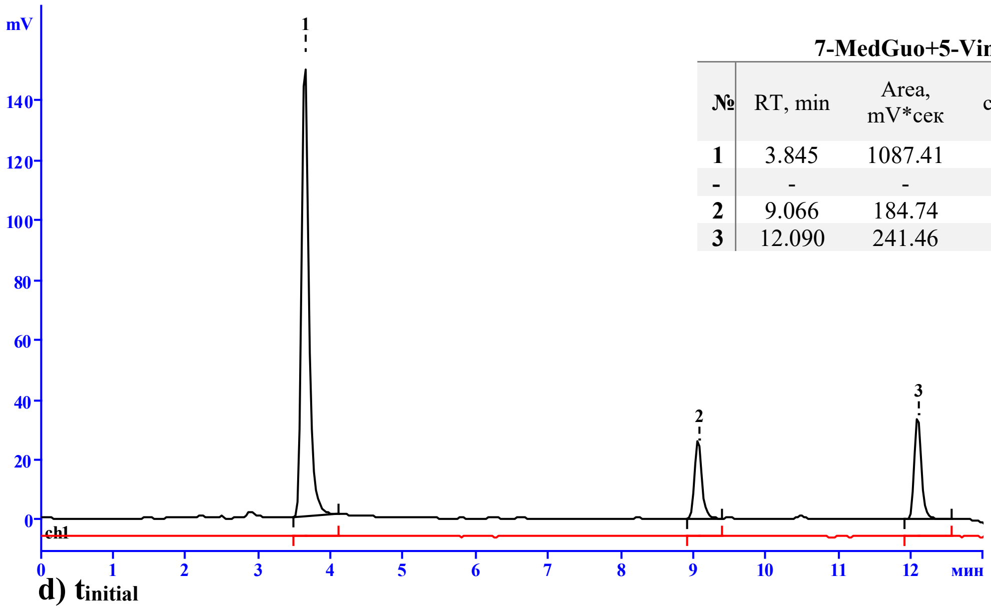

d) $t_{initial}$

7-MedGuo+Hyp – 1:1 – t_0

№	RT, min	Area, mV*сек	Equilibrium concentration, µM	Name
**2**	3.031	1556.08	200.00	Hyp
**5**	6.306	1406.01	200.00	7-MedGuo

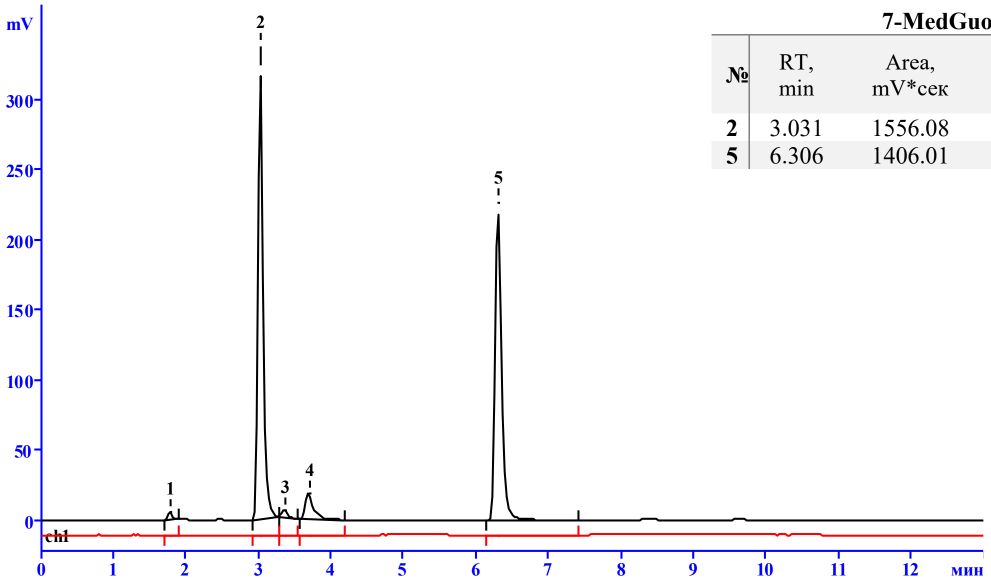

$t_{equilibrium}$

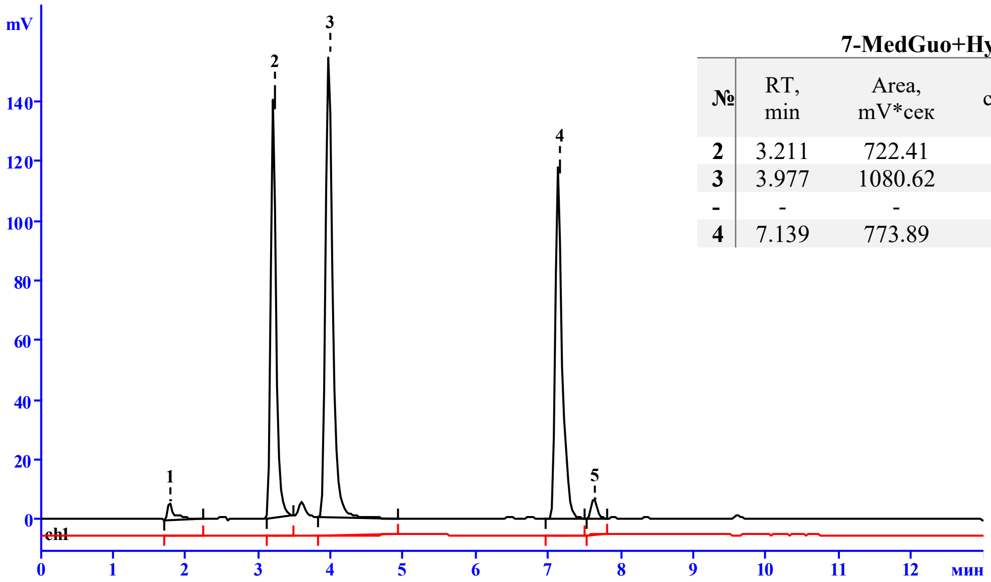

7-MedGuo+Hyp – 1:1– $t_{equilibrium}$

№	RT, min	Area, mV*сек	Equilibrium concentration, µM	Name
2	3.211	722.41	92,85	Hyp
3	3.977	1080.62	200.00	7-MeGua
-	-	-	-	7-MedGuo
4	7.139	773.89	107.15	Ino

d) $t_{initial}$

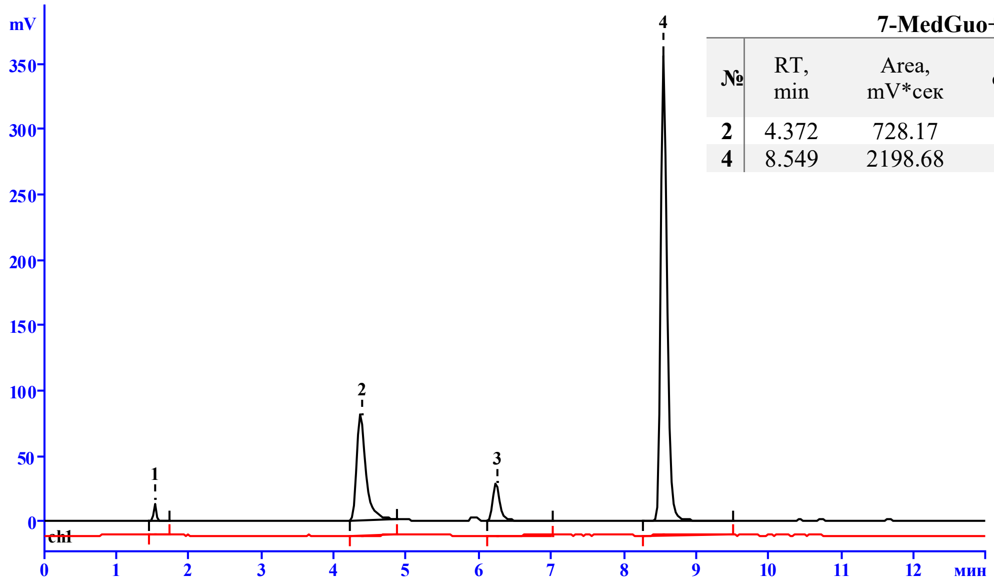

7-MedGuo+Hyp – 3:1 – t_0

№	RT, min	Area, mV*сек	Equilibrium concentration, µM	Name
2	4.372	728.17	100.00	Hyp
4	8.549	2198.68	300.00	7-MedGuo

$t_{equilibrium}$

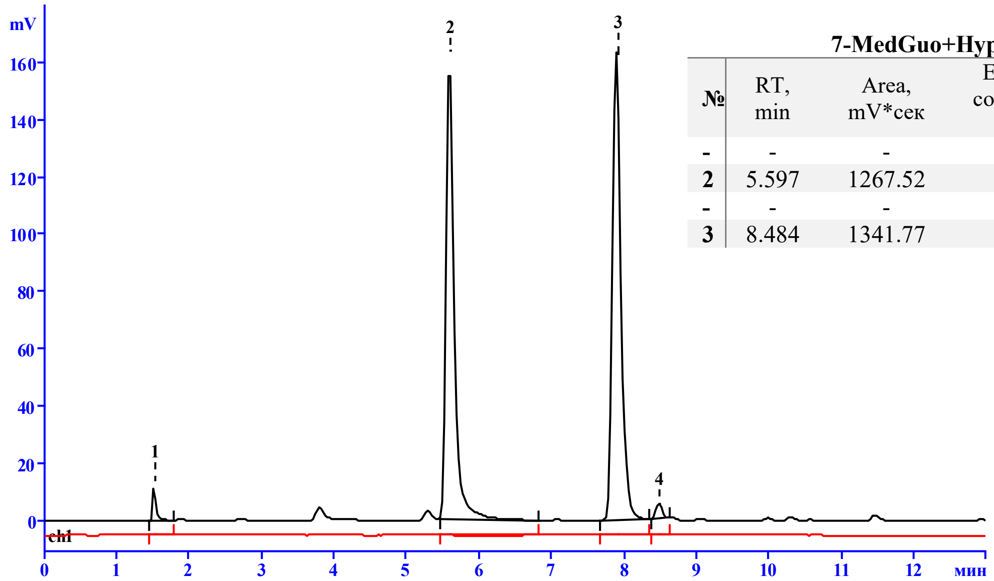

7-MedGuo+Hyp – 3:1– $t_{equilibrium}$

№	RT, min	Area, mV*сек	Equilibrium concentration, µM	Name
-	-	-	-	Hyp
2	5.597	1267.52	300.00	7-MeGua
-	-	-	-	7-MedGuo
3	8.484	1341.77	100.00	Ino

Fugure S10.3. pH = 8.5

1) 7.8*10^{-3} U *LlNDT II*

a) t$_{initial}$

7-MedGuo+Ura – 3:1 – t$_0$

№	RT, min	Area, mV*сек	Equilibrium concentration, µM	Name
2	2.764	945.29	100.00	Ura
4	6.478	2114.65	300.00	7-MedGuo

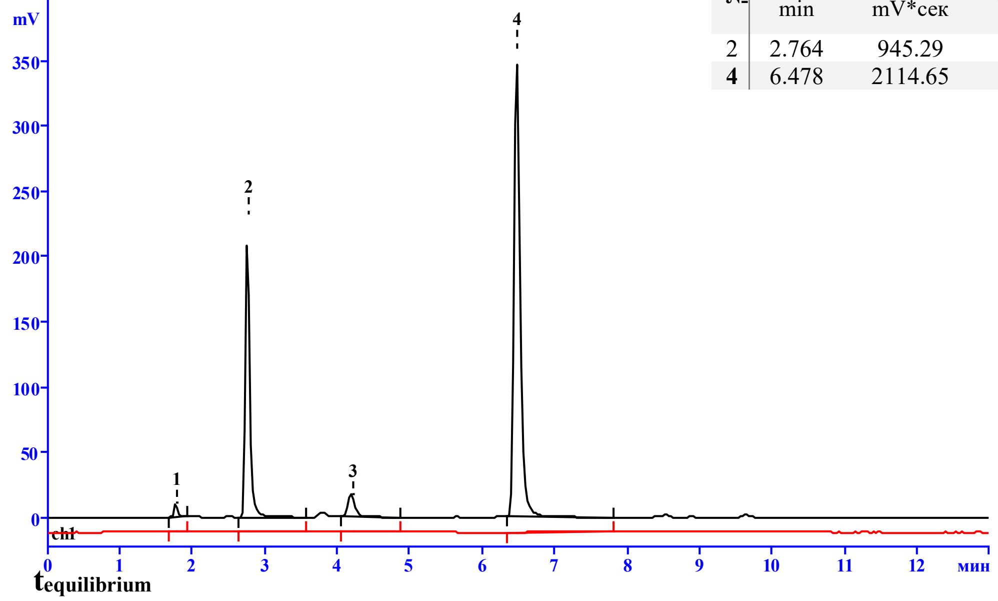

t$_{equilibrium}$

7-MedGuo+Ura – 3:1– t $_{equilibrium}$

№	RT, min	Area, mV*сек	Equilibrium concentration, µM	Name
2	2.749	763.35	80.75	Ura
3	4.016	315.13	19.25	dUrd
4	5.717	204.06	44.51	7-MeGua
5	6.504	1800.88	255.49	7-MedGuo

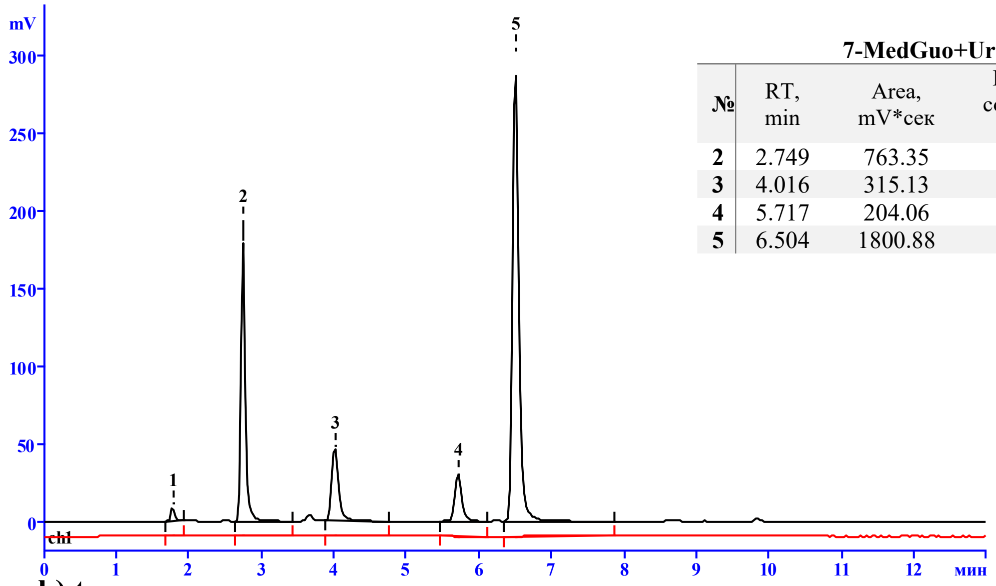

b) t$_{initial}$

7-MedGuo+Thy – 3:1 – t$_0$

№	RT, min	Area, mV*сек	Equilibrium concentration, µM	Name
3	5.281	713.06	100.00	Thy
4	6.452	2159.89	300.00	7-MedGuo

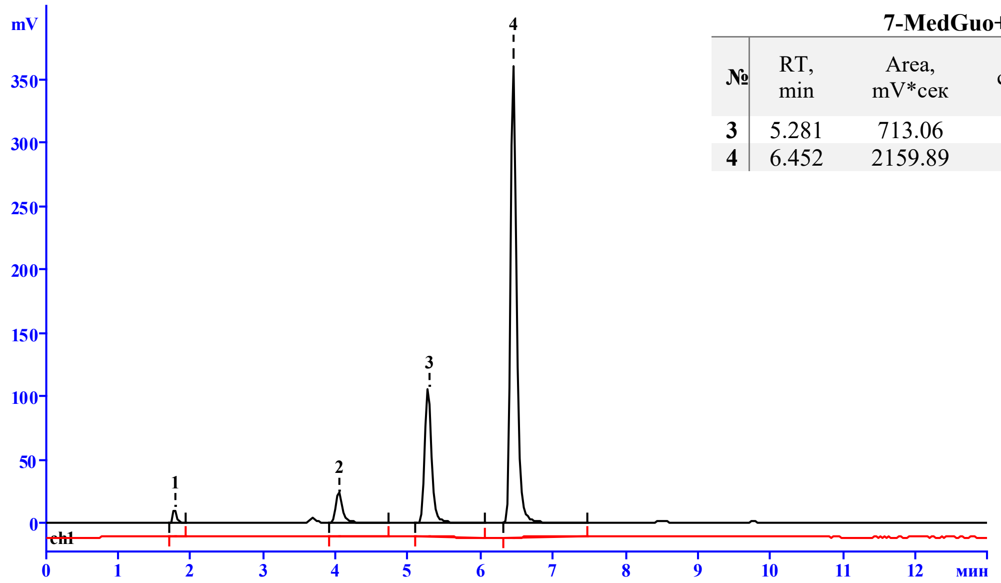

tequilibrium

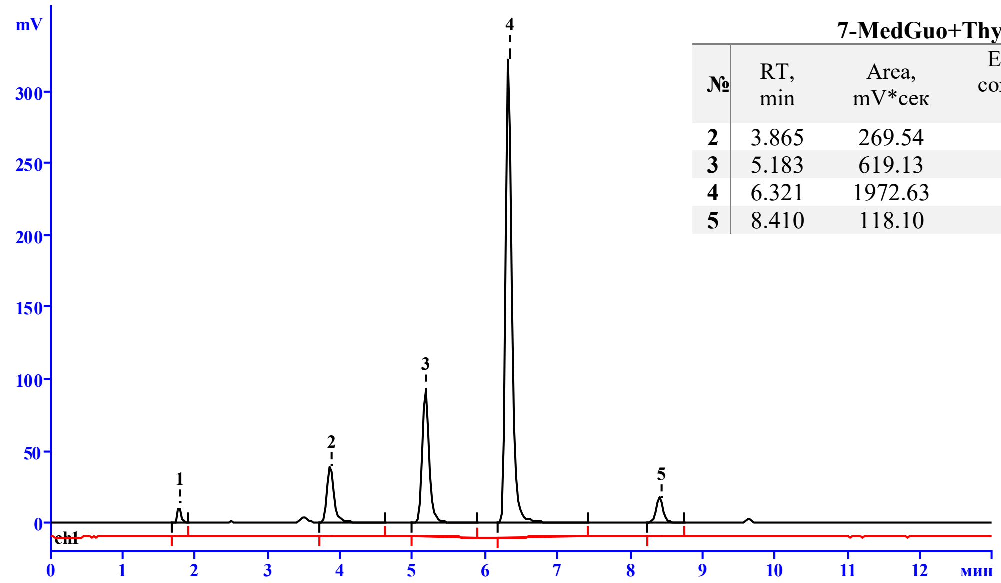

7-MedGuo+Thy – 3:1– t equilibrium

№	RT, min	Area, mV*сек	Equilibrium concentration, µM	Name
**2**	3.865	269.54	26.01	7-MeGua
**3**	5.183	619.13	86.83	Thy
**4**	6.321	1972.63	273.99	7-MedGuo
**5**	8.410	118.10	13.17	dThd

c) tinitial

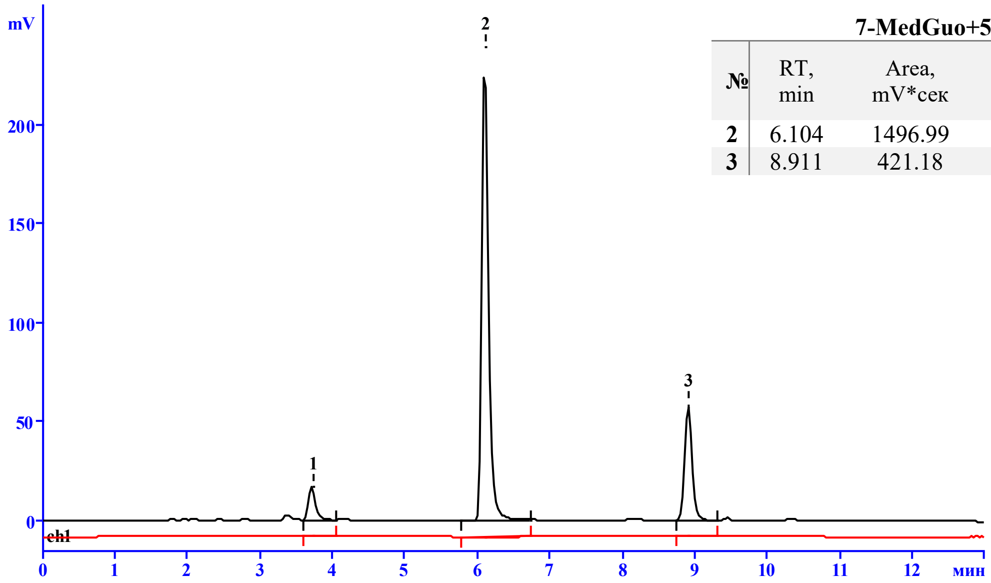

7-MedGuo+5-VinUra – 3:1 – t0

№	RT, min	Area, mV*сек	Equilibrium concentration, µM	Name
**2**	6.104	1496.99	300.00	7-MedGuo
**3**	8.911	421.18	100.00	5-VinUra

tequilibrium

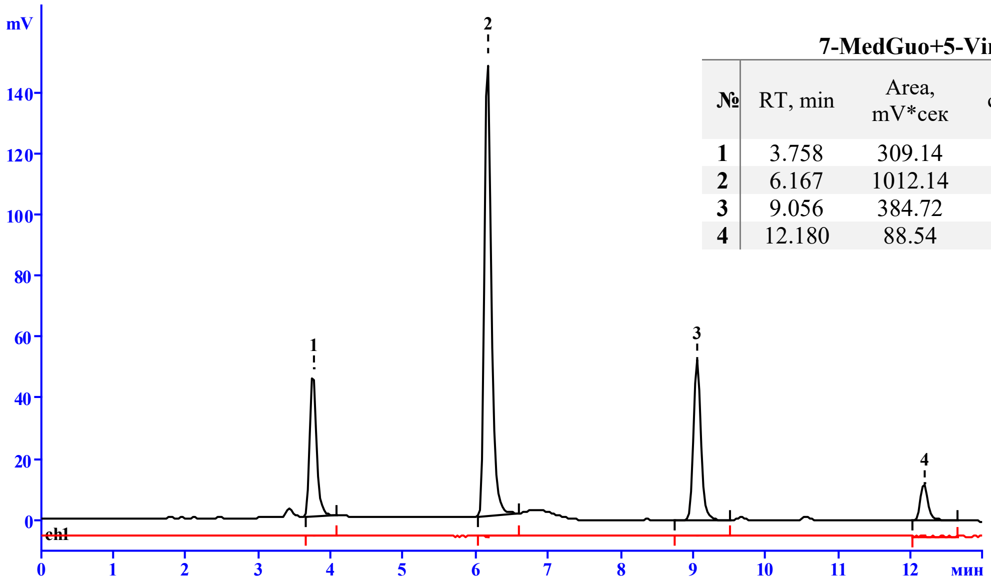

7-MedGuo+5-VinUra – 3:1– t equilibrium

№	RT, min	Area, mV*сек	Equilibrium concentration, µM	Name
**1**	3.758	309.14	97.16	7-MeGua
**2**	6.167	1012.14	202.84	7-MedGuo
**3**	9.056	384.72	91.34	5-VinUra
**4**	12.180	88.54	8.66	5-VindUrd

d) $t_{initial}$

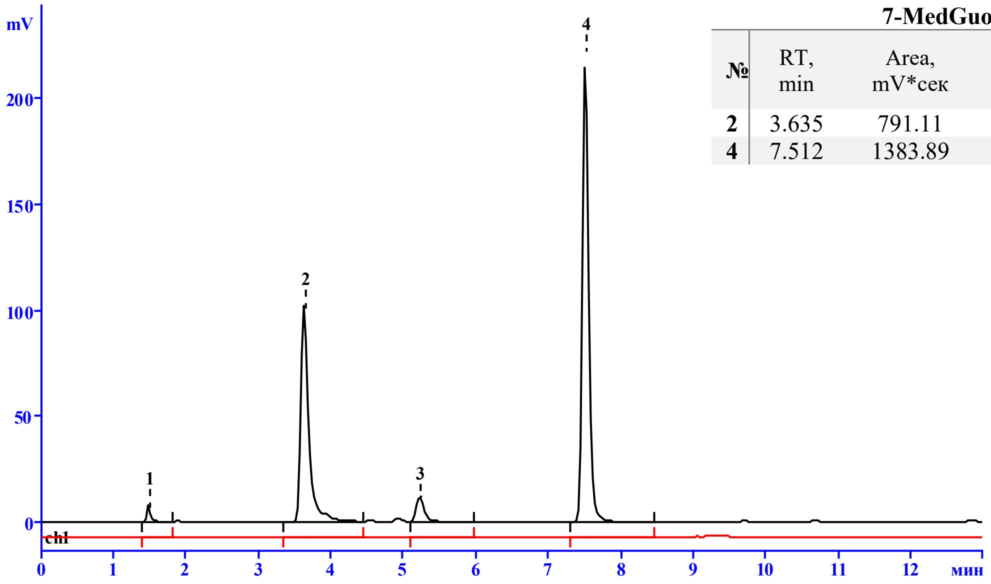

7-MedGuo+Hyp – 3:1 – t_0

№	RT, min	Area, mV*сек	Equilibrium concentration, µM	Name
**2**	3.635	791.11	100.00	Hyp
**4**	7.512	1383.89	300.00	7-MedGuo

$t_{equilibrium}$

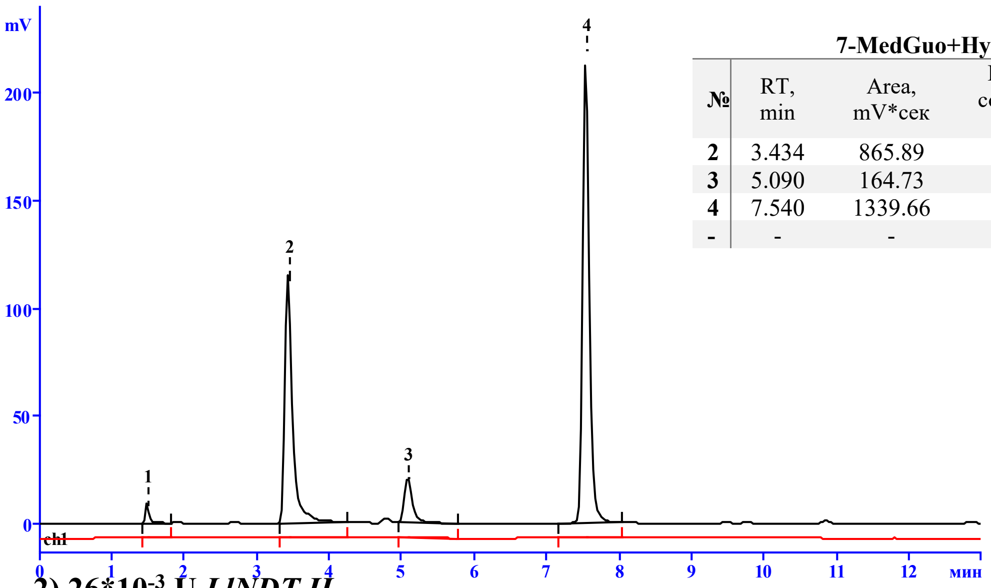

7-MedGuo+Hyp – 3:1– $t_{equilibrium}$

№	RT, min	Area, mV*сек	Equilibrium concentration, µM	Name
**2**	3.434	865.89	100.00	Hyp
**3**	5.090	164.73	290.41	7-MeGua
**4**	7.540	1339.66	9.59	7-MedGuo
**-**	-	-	-	Ino

2) 26*10^{-3} U *LlNDT II*

a) $t_{initial}$

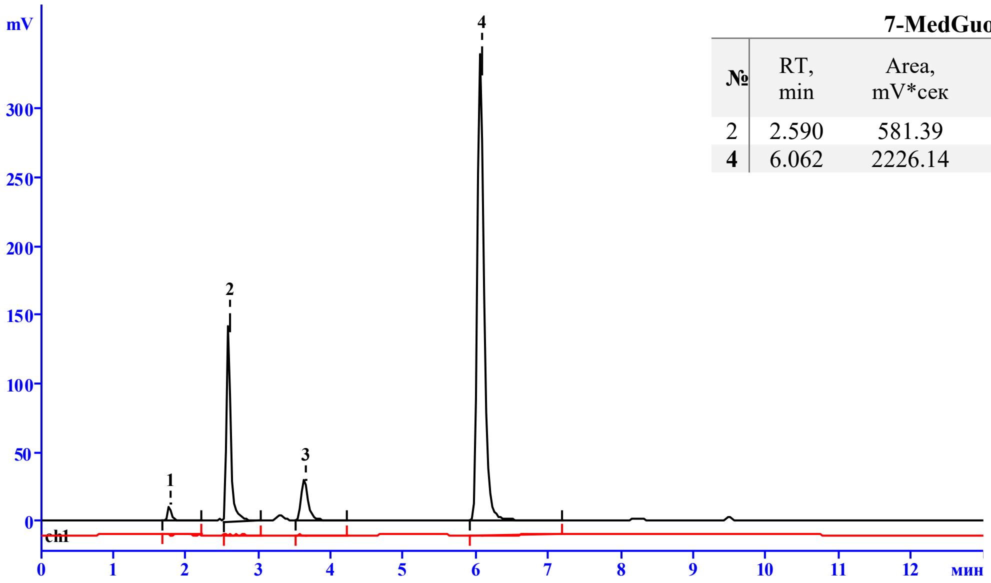

7-MedGuo+Ura – 3:1 – t_0

№	RT, min	Area, mV*сек	Equilibrium concentration, µM	Name
2	2.590	581.39	100.00	Ura
**4**	6.062	2226.14	300.00	7-MedGuo

$t_{equilibrium}$

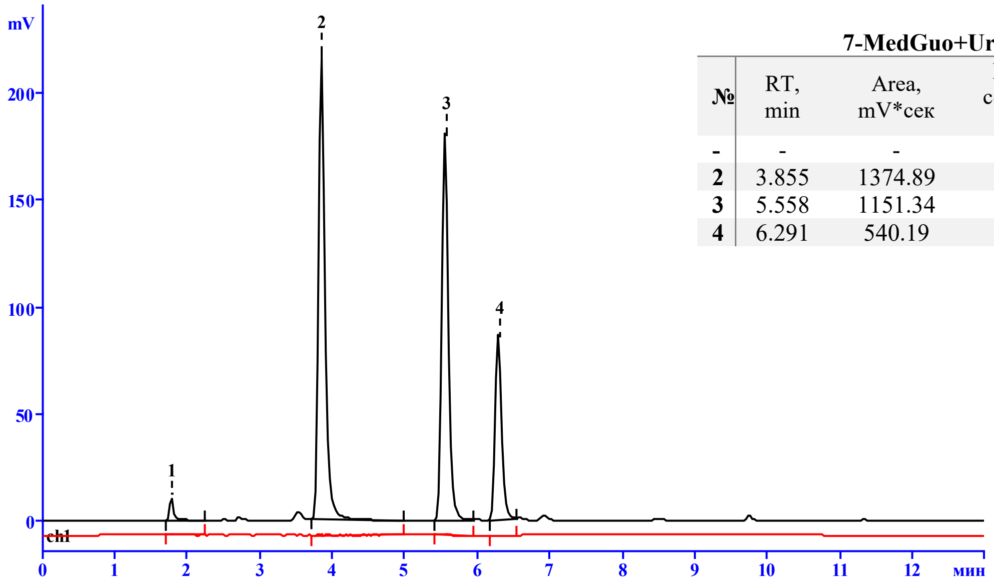

7-MedGuo+Ura – 3:1– t equilibrium

№	RT, min	Area, mV*сек	Equilibrium concentration, µM	Name
-	-	-	-	Ura
2	3.855	1374.89	100.00	dUrd
3	5.558	1151.34	227.20	7-MeGua
4	6.291	540.19	72.80	7-MedGuo

b) $t_{initial}$

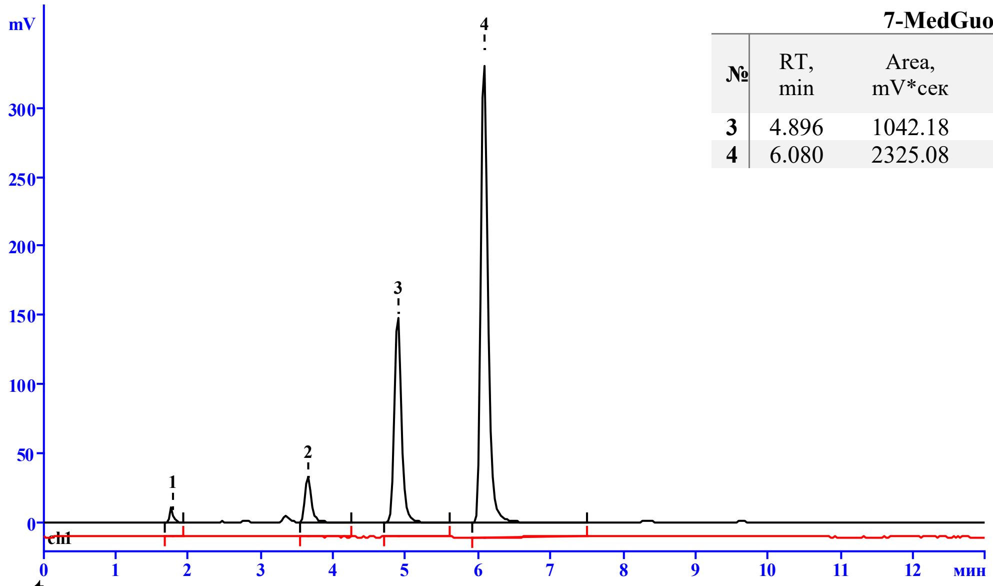

7-MedGuo+Thy – 3:1 – t₀

№	RT, min	Area, mV*сек	Equilibrium concentration, µM	Name
3	4.896	1042.18	100.00	Thy
4	6.080	2325.08	300.00	7-MedGuo

$t_{equilibrium}$

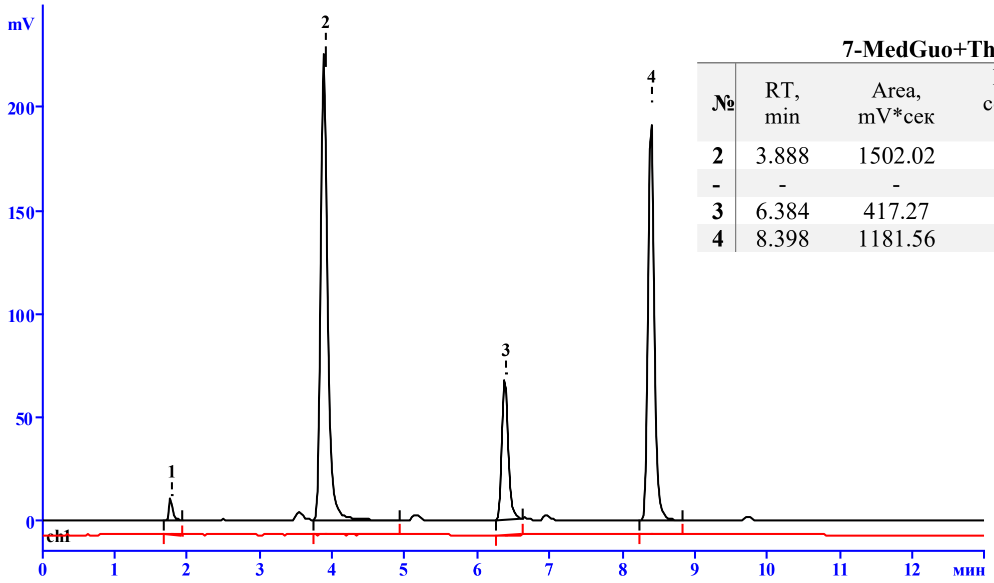

7-MedGuo+Thy – 3:1– t equilibrium

№	RT, min	Area, mV*сек	Equilibrium concentration, µM	Name
2	3.888	1502.02	246.16	7-MeGua
-	-	-	-	Thy
3	6.384	417.27	53.84	7-MedGuo
4	8.398	1181.56	100.00	dThd

c) $t_{initial}$

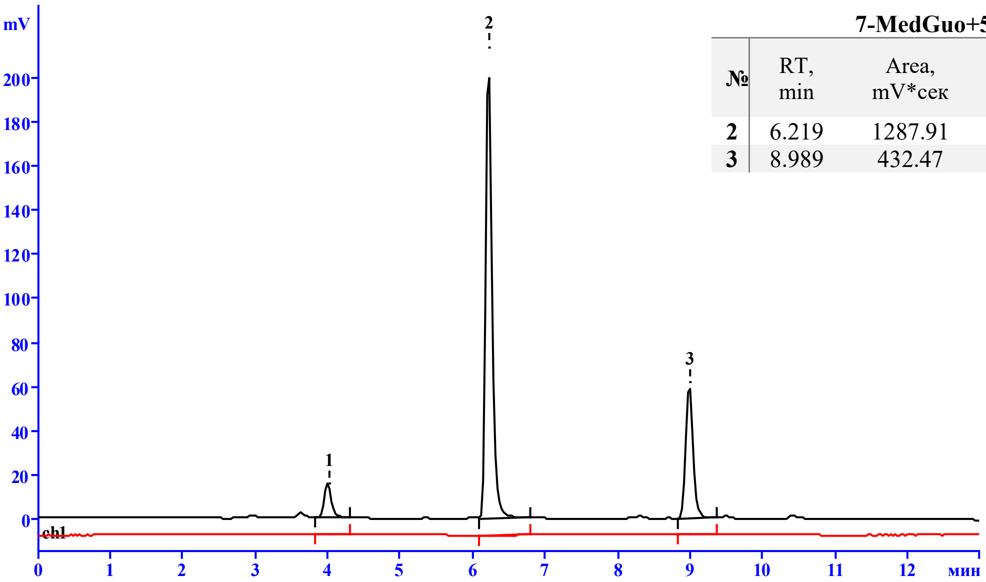

7-MedGuo+5-VinUra – 3:1 – t_0

№	RT, min	Area, mV*сек	Equilibrium concentration, µM	Name
**2**	6.219	1287.91	300.00	7-MedGuo
**3**	8.989	432.47	100.00	5-VinUra

$t_{equilibrium}$

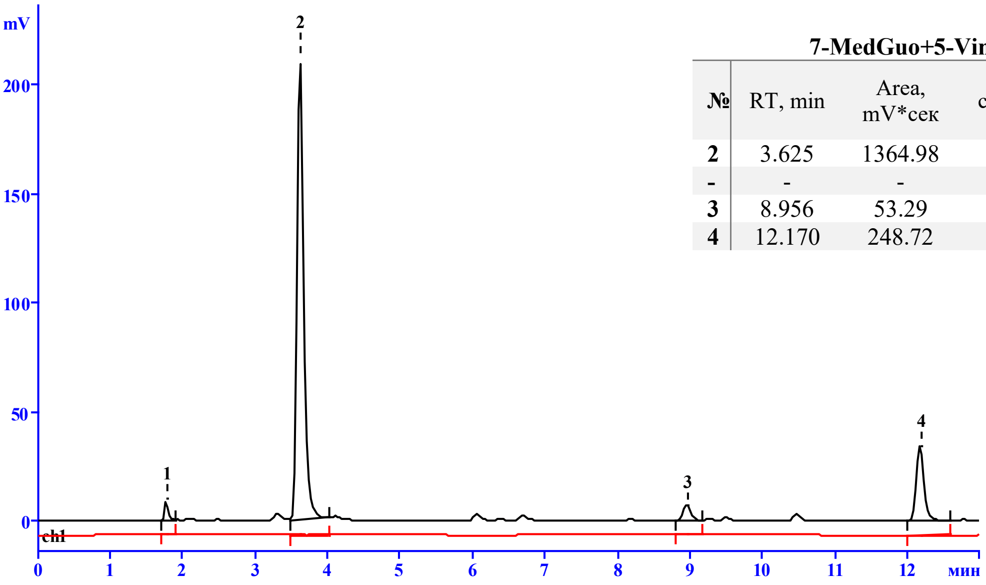

7-MedGuo+5-VinUra – 3:1– t $_{equilibrium}$

№	RT, min	Area, mV*сек	Equilibrium concentration, µM	Name
**2**	3.625	1364.98	300.00	7-MeGua
**-**	-	-	-	7-MedGuo
**3**	8.956	53.29	12.32	5-VinUra
**4**	12.170	248.72	87.68	5-VindUrd

d) $t_{initial}$

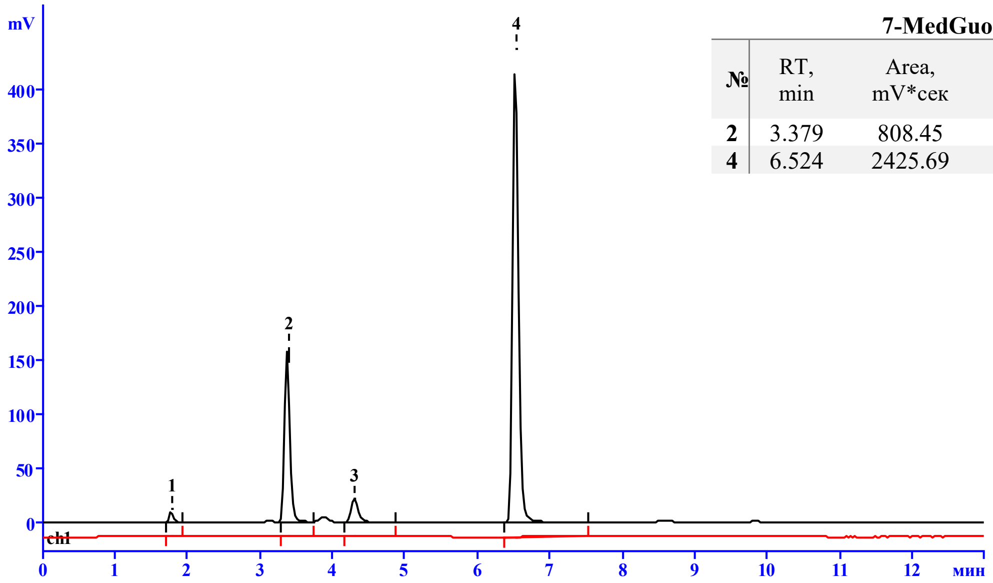

7-MedGuo+Hyp – 3:1 – t_0

№	RT, min	Area, mV*сек	Equilibrium concentration, µM	Name
**2**	3.379	808.45	100.00	Hyp
**4**	6.524	2425.69	300.00	7-MedGuo

$t_{equilibrium}$

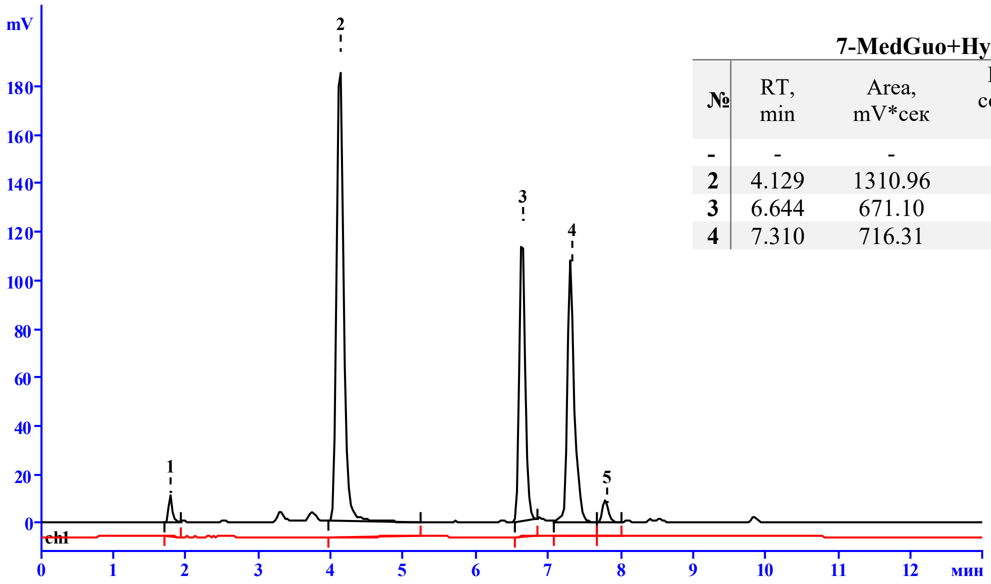

7-MedGuo+Hyp – 3:1– t equilibrium

№	RT, min	Area, mV*сек	Equilibrium concentration, µM	Name
-	-	-	-	Hyp
2	4.129	1310.96	217.00	7-MeGua
3	6.644	671.10	83.00	7-MedGuo
4	7.310	716.31	100.00	Ino

5-trifluoromethyl-2'-deoxyuridine, 6-oxomethylpurine-2'-deoxyriboside from 7-methyldeoxyguanosine (7-MedGuo+ 5-CF3Ura, 6-omPur, analytical method)

HPLC-analysis of transglycosylation: Cosmosil 5C18-MS-II, 4.6*150 mm, 5µm, 120 Å, (Nacalai Tesque, Inc. (Japan)), 20ºC, elution in modified mobile phase: linear gradient of acetonitrile in 0.06% TFA from 2 to 20% for 10 min (flushing with 20-80 % acetonitrile-0.06% TFA for 10-10.1 min, then 80-2% for 10.1-10.8 min) at flow rate 1 ml/min with UV detection at wavelength 260 nm, injection volume 20 µL.

Fugure S11.1. pH = 5.5, 7.8*10^{-3} U *LlNDT II*

7-MedGuo+5-CF$_3$Ura – 3:1 – t$_0$

№	RT, min	Area, mV*сек	Equilibrium concentration, µM	Name
3	6.353	1846.73	100.00	7-MedGuo
4	8.593	920.29	300.00	5-CF$_3$Ura

a) $t_{initial}$

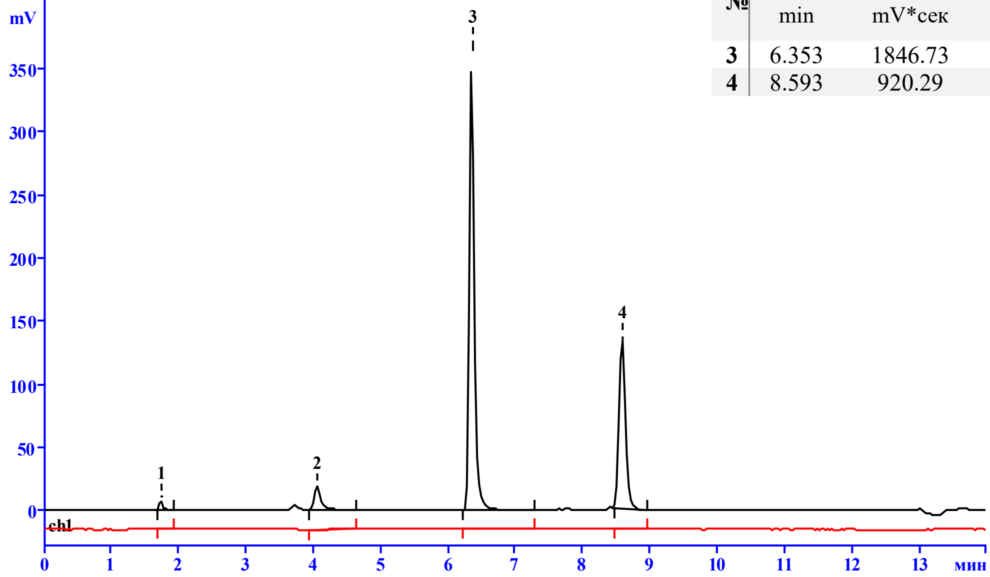

$t_{equilibrium}$

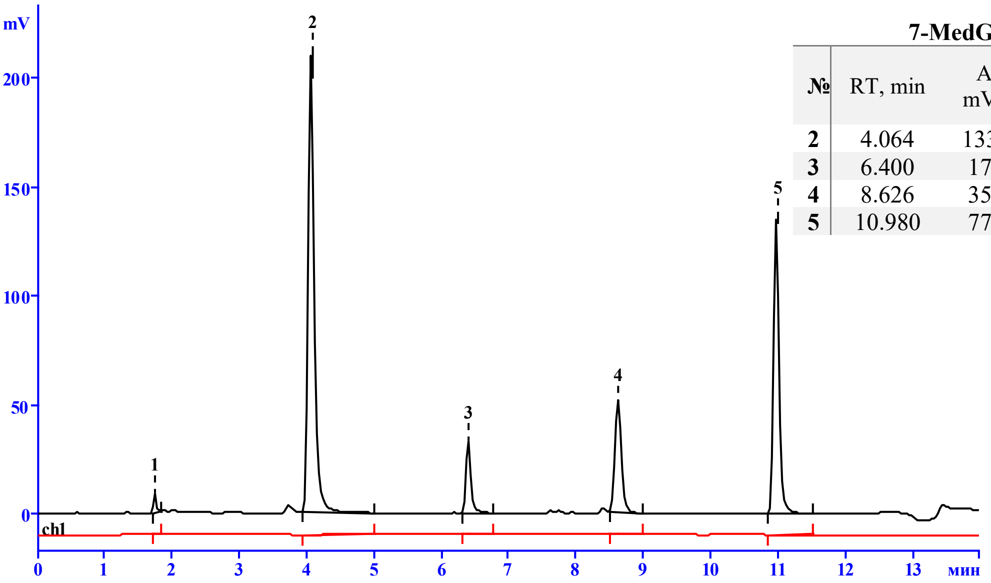

7-MedGuo+5-CF_3Ura – 3:1– $t_{equilibrium}$

№	RT, min	Area, mV*сек	Equilibrium concentration, µM	Name
2	4.064	1339.99	272.13	7-MeGua
3	6.400	171.54	27.87	7-MedGuo
4	8.626	354.74	38.55	5-CF_3Ura
5	10.980	772.61	61.45	5-CF3dUrd

b) $t_{initial}$

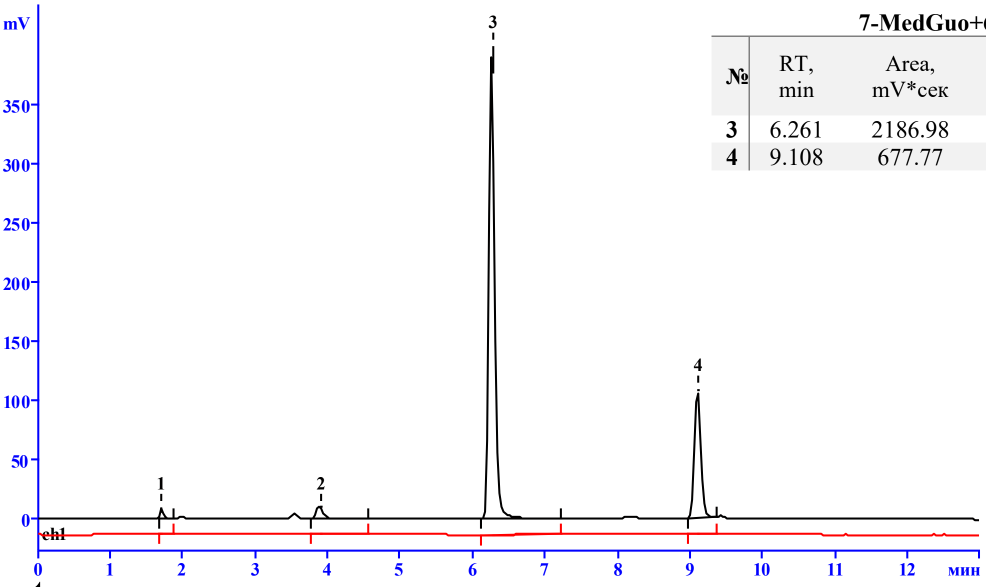

7-MedGuo+6-omPur – 3:1 – t_0

№	RT, min	Area, mV*сек	Equilibrium concentration, µM	Name
3	6.261	2186.98	100.00	7-MedGuo
4	9.108	677.77	300.00	6-omPur

$t_{equilibrium}$

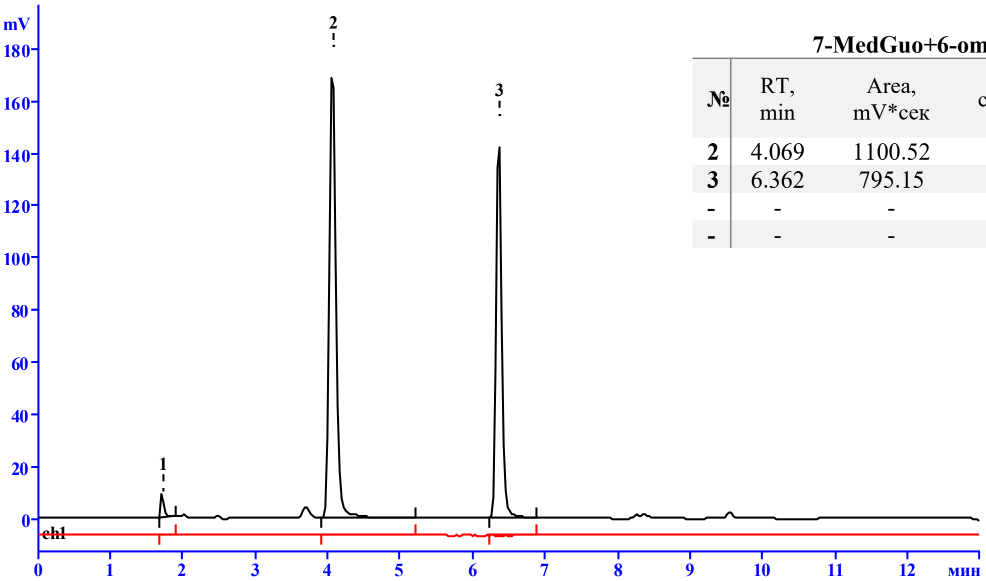

7-MedGuo+6-omPur – 3:1– $t_{equilibrium}$

№	RT, min	Area, mV*сек	Equilibrium concentration, µM	Name
2	4.069	1100.52	190.92	7-MeGua
3	6.362	795.15	109.08	7-MedGuo
-	-	-	-	6-omPur
-	-	-	100.00	6-omdPur

Fugure S11.2. **pH = 7.0, 7.8*10^{-3} U *LlNDT II***

a) t$_{initial}$

7-MedGuo+5-CF_3Ura – 3:1 – t$_0$

№	RT, min	Area, mV*сек	Equilibrium concentration, µM	Name
**3**	6.286	1836.18	100.00	7-MedGuo
**4**	8.624	825.17	300.00	5-CF_3Ura

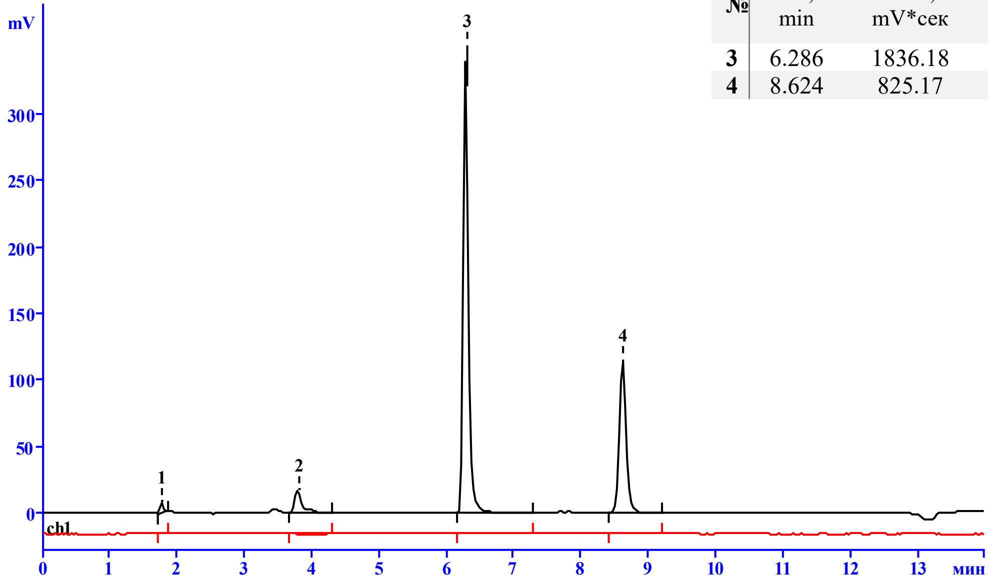

t$_{equilibrium}$

7-MedGuo+5-CF_3Ura – 3:1– t $_{equilibrium}$

№	RT, min	Area, mV*сек	Equilibrium concentration, µM	Name
**2**	3.870	1227.53	243.99	7-MeGua
**3**	6.284	342.80	56.01	7-MedGuo
**4**	8.602	353.29	42.81	5-CF_3Ura
**5**	10.980	779.59	57.19	5-CF3dUrd

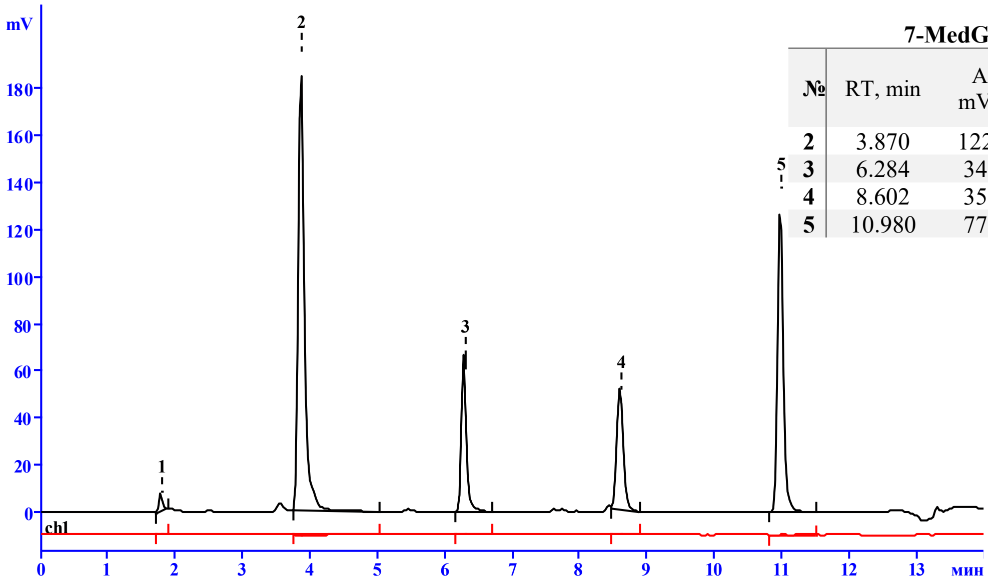

b) t$_{initial}$

7-MedGuo+6-omPur – 1:1 – t$_0$

№	RT, min	Area, mV*сек	Equilibrium concentration, µM	Name
**3**	6.663	1449.51	200.00	7-MedGuo
**4**	8.770	1352.69	200.00	6-omPur

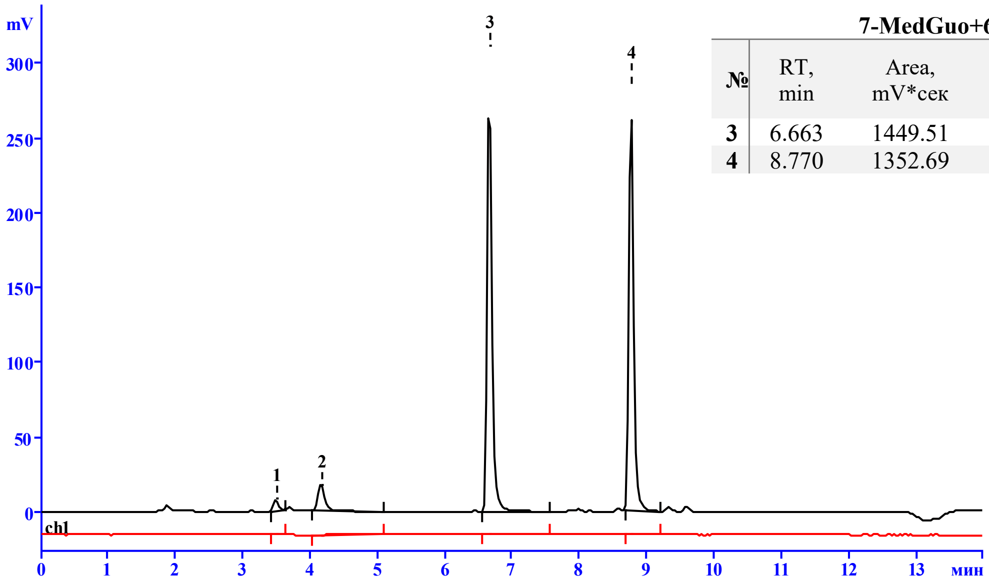

$t_{equilibrium}$

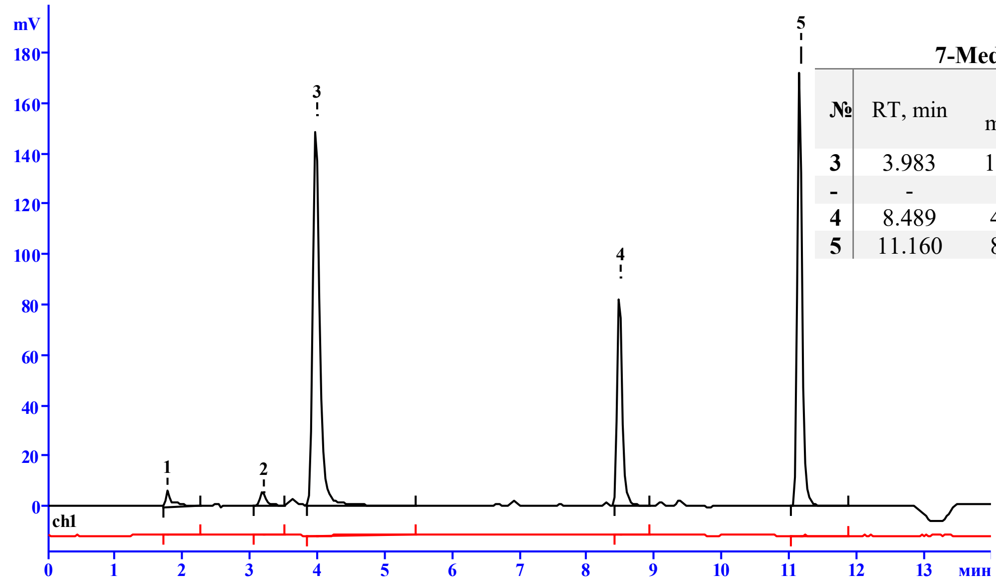

7-MedGuo+6-omPur – 1:1– t equilibrium

№	RT, min	Area, mV*сек	Equilibrium concentration, µM	Name
3	3.983	1087.58	200.00	7-MeGua
-	-	-	-	7-MedGuo
4	8.489	452.32	66.88	6-omPur
5	11.160	872.12	133.12	6-omdPur

$t_{initial}$

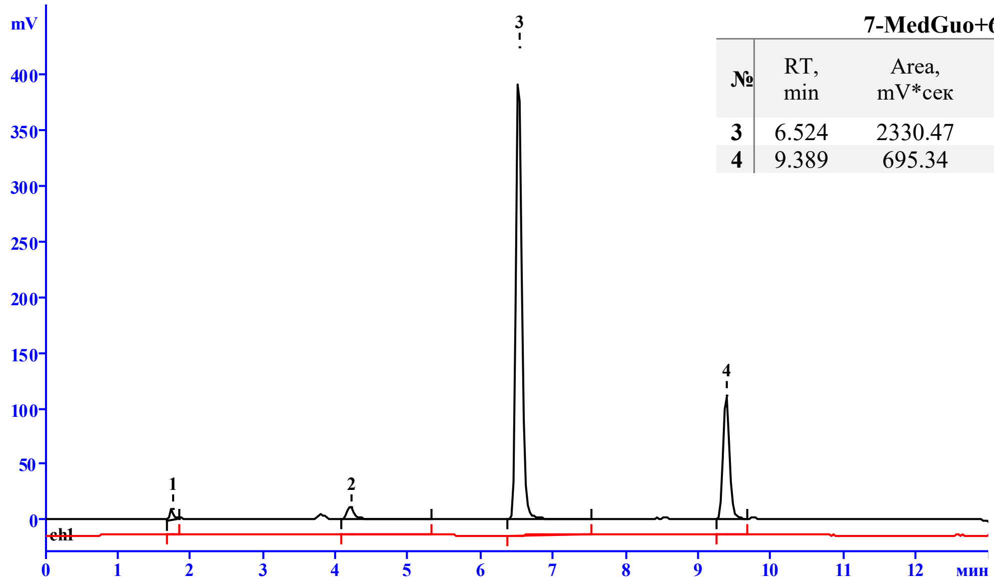

7-MedGuo+6-omPur – 3:1 – t_0

№	RT, min	Area, mV*сек	Equilibrium concentration, µM	Name
3	6.524	2330.47	100.00	7-MedGuo
4	9.389	695.34	300.00	6-omPur

$t_{equilibrium}$

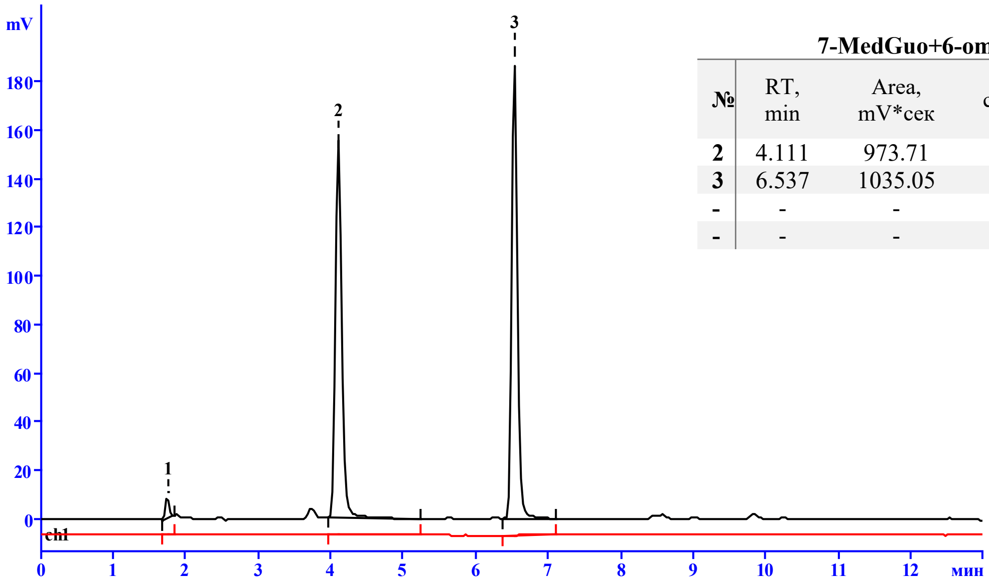

7-MedGuo+6-omPur – 3:1– t equilibrium

№	RT, min	Area, mV*сек	Equilibrium concentration, µM	Name
2	4.111	973.71	166.76	7-MeGua
3	6.537	1035.05	133.24	7-MedGuo
-	-	-	-	6-omPur
-	-	-	100.00	6-omdPur

Fugure S11.3. pH = 8.5

1) $7.8*10^{-3}$ U *LlNDT II*

a) $t_{initial}$

7-MedGuo+5-CF_3Ura – 3:1 – t_0

№	RT, min	Area, mV*сек	Equilibrium concentration, µM	Name
3	6.331	1841.06	100.00	7-MedGuo
4	8.581	889.91	300.00	5-CF_3Ura

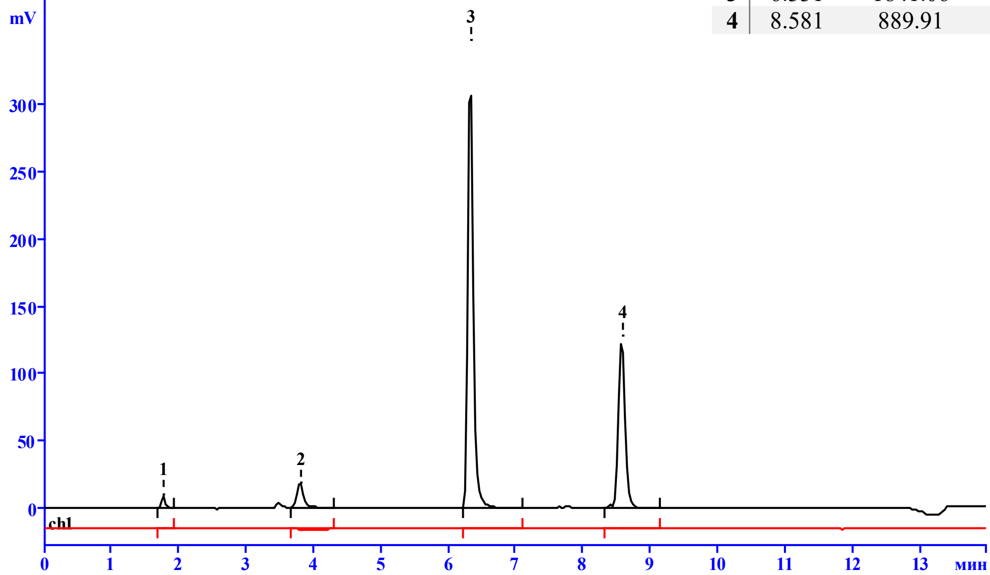

$t_{equilibrium}$

7-MedGuo+5-CF_3Ura – 3:1– t $_{equilibrium}$

№	RT, min	Area, mV*сек	Equilibrium concentration, µM	Name
2	4.166	141.52	10.86	7-MeGua
4	6.425	1774.42	289.14	7-MedGuo
5	8.662	903.49	100.00	5-CF_3Ura
-	-	-	-	5-CF3dUrd

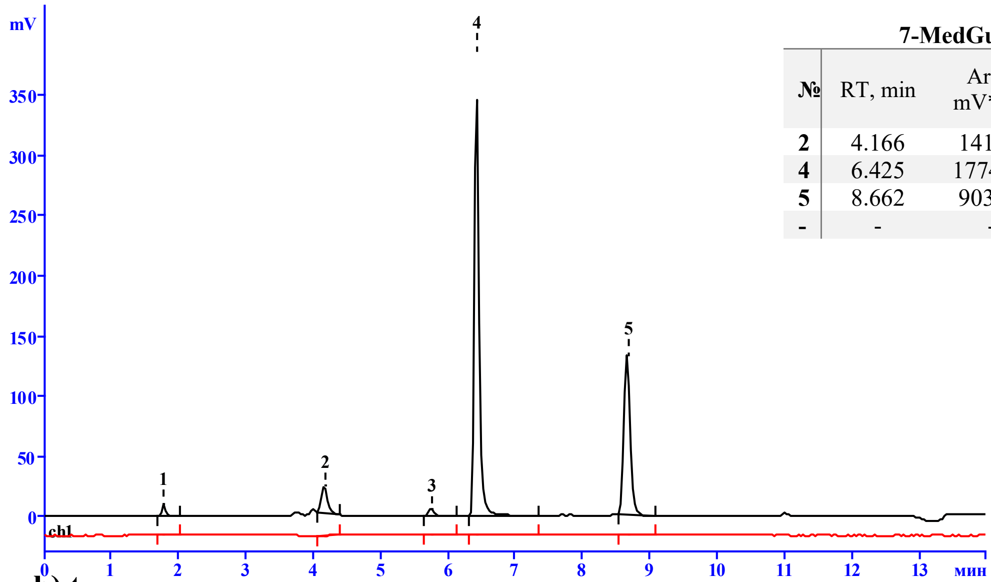

b) $t_{initial}$

7-MedGuo+6-omPur – 3:1 – t_0

№	RT, min	Area, mV*сек	Equilibrium concentration, µM	Name
3	7.364	1254.29	100.00	7-MedGuo
4	10.880	725.10	300.00	6-omPur

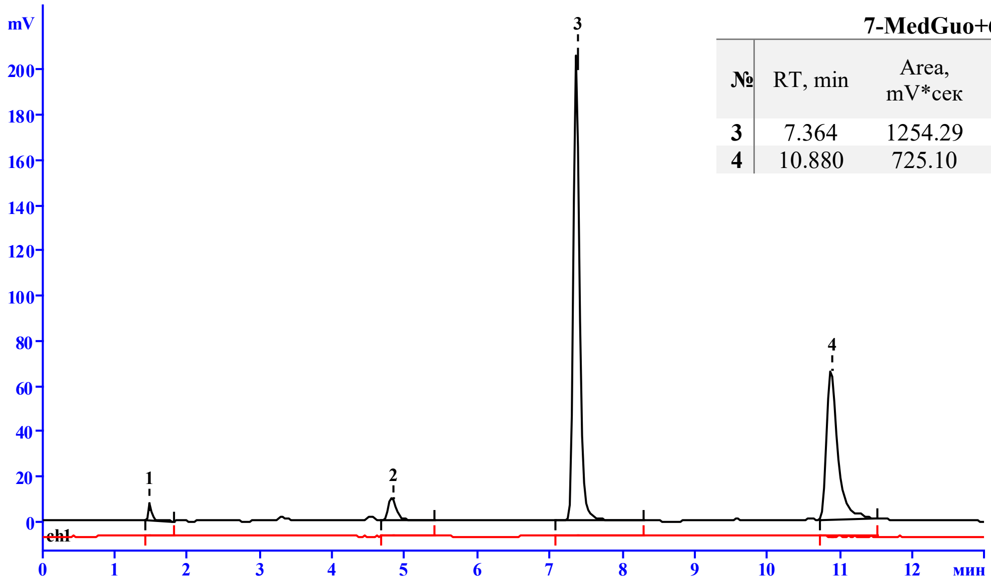

$t_{equilibrium}$

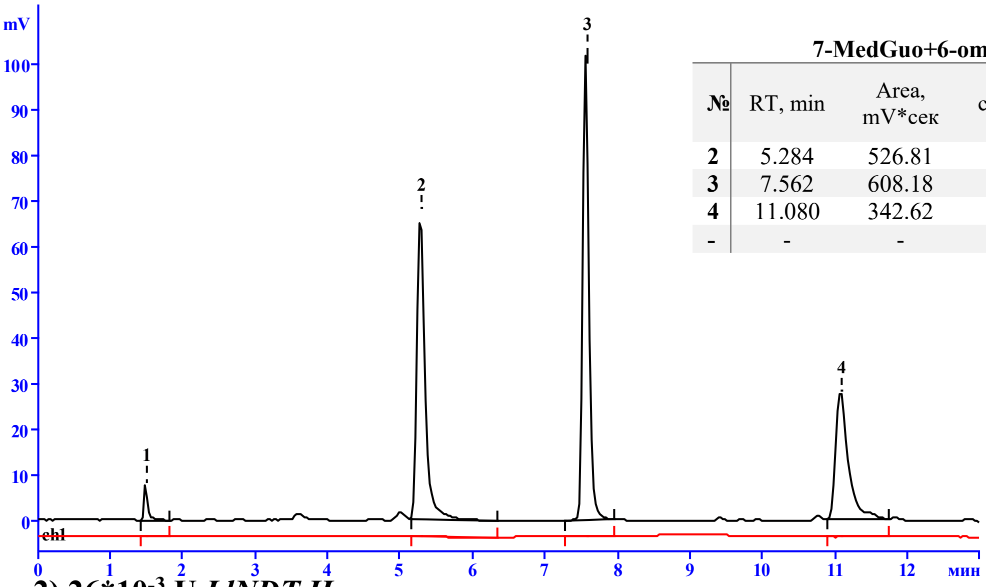

7-MedGuo+6-omPur – 3:1– $t_{equilibrium}$

№	RT, min	Area, mV*сек	Equilibrium concentration, μM	Name
**2**	5.284	526.81	154.54	7-MeGua
**3**	7.562	608.18	145.46	7-MedGuo
**4**	11.080	342.62	47.25	6-omPur
**-**	-	-	52.75	6-omdPur

2) 26*10^{-3} U *LlNDT II*

a) $t_{initial}$

7-MedGuo+5-CF_3Ura – 3:1 – t_0

№	RT, min	Area, mV*сек	Equilibrium concentration, μM	Name
**3**	6.387	1982.14	100.00	7-MedGuo
**4**	8.603	905.41	300.00	5-CF_3Ura

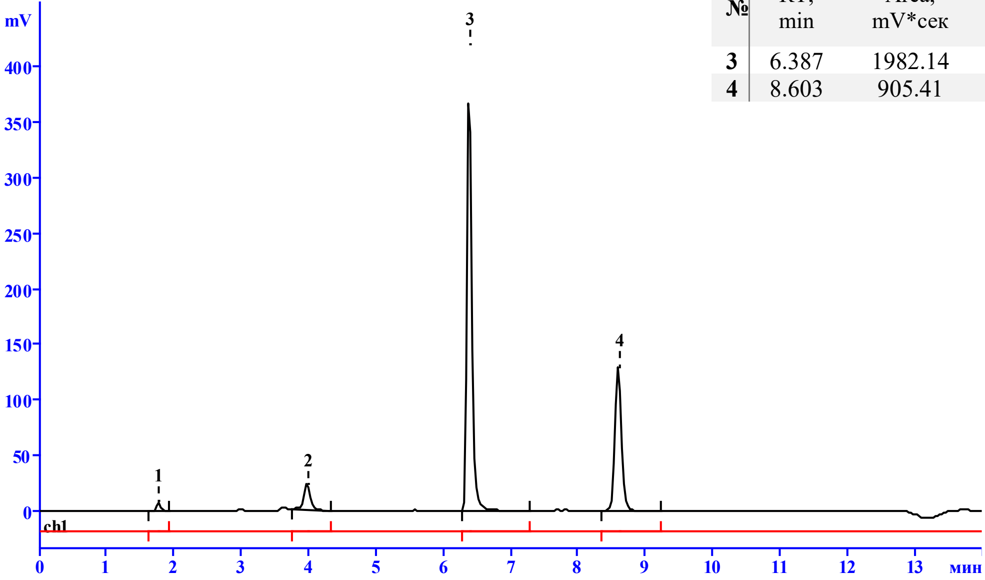

$t_{equilibrium}$

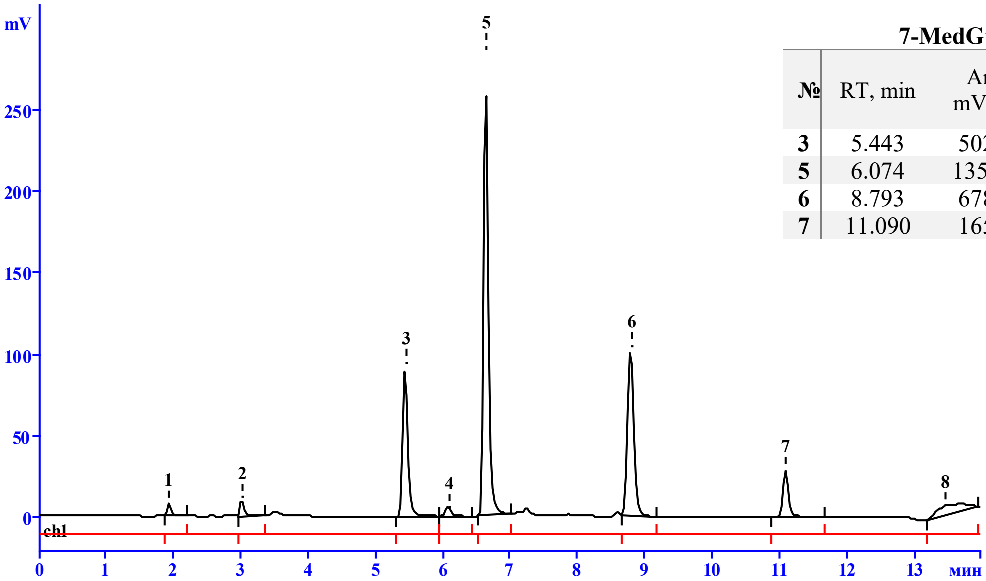

7-MedGuo+5-CF_3Ura – 3:1– $t_{equilibrium}$

№	RT, min	Area, mV*сек	Equilibrium concentration, μM	Name
**3**	5.443	502.46	95.54	7-MeGua
**5**	6.074	1350.89	204.46	7-MedGuo
**6**	8.793	678.16	74.90	5-CF_3Ura
**7**	11.090	165.25	25.10	5-CF3dUrd

b) $t_{initial}$

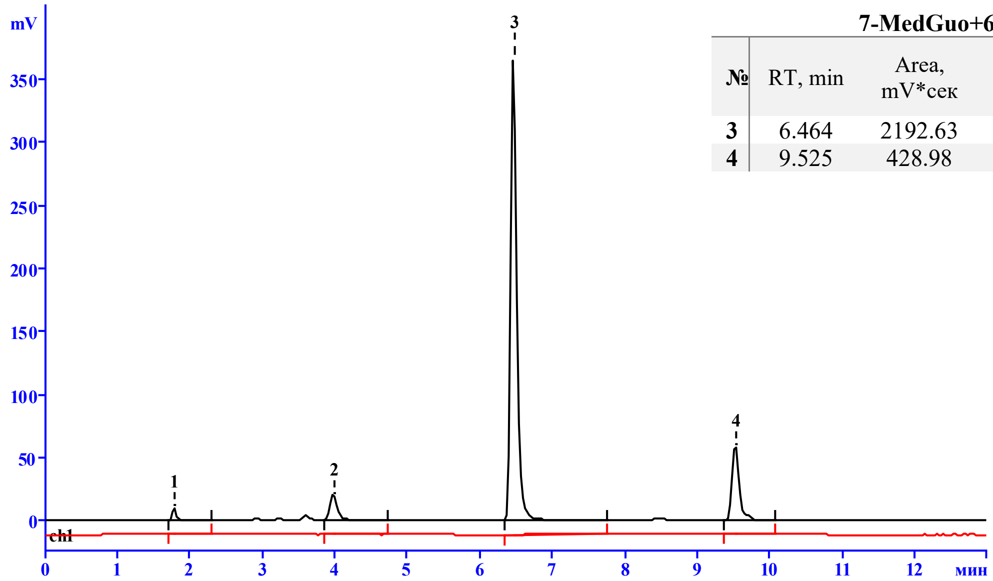

7-MedGuo+6-omPur – 3:1 – t_0

№	RT, min	Area, mV*сек	Equilibrium concentration, µM	Name
**3**	6.464	2192.63	100.00	7-MedGuo
**4**	9.525	428.98	300.00	6-omPur

$t_{equilibrium}$

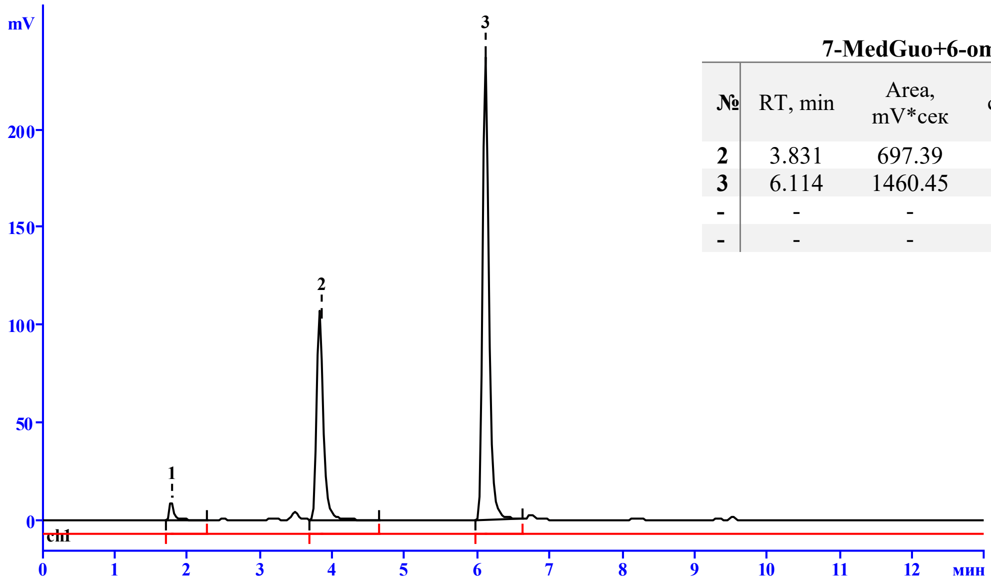

7-MedGuo+6-omPur – 3:1– $t_{equilibrium}$

№	RT, min	Area, mV*сек	Equilibrium concentration, µM	Name
**2**	3.831	697.39	100.18	7-MeGua
**3**	6.114	1460.45	199.82	7-MedGuo
**-**	-	-	-	6-omPur
**-**	-	-	100.00	6-omdPur

3) 52*10^{-3} U *LlNDT II*

$t_{initial}$

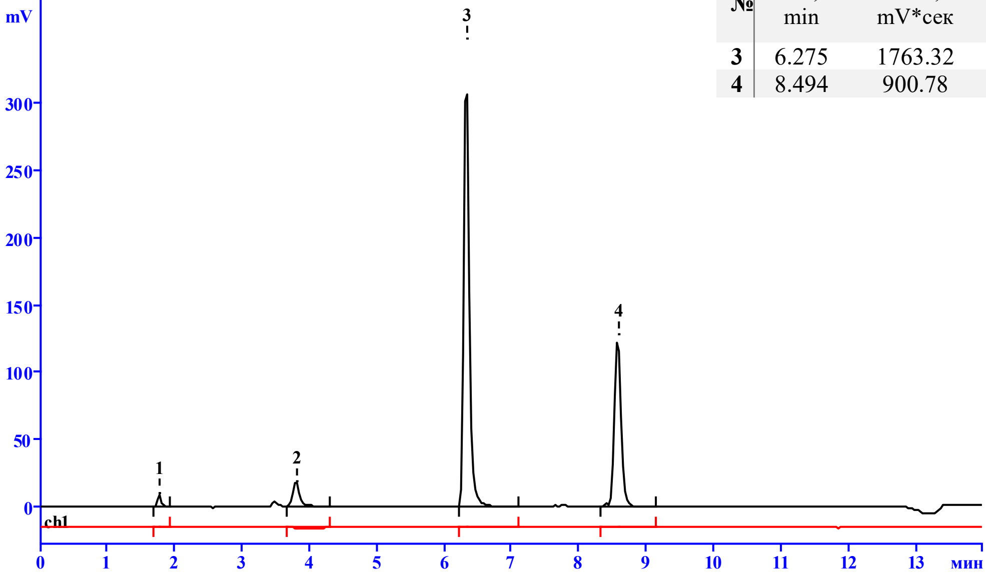

7-MedGuo+5-CF_3Ura – 3:1 – t_0

№	RT, min	Area, mV*сек	Equilibrium concentration, µM	Name
**3**	6.275	1763.32	300.00	7-MedGuo
**4**	8.494	900.78	100.00	5-CF_3Ura

$t_{equilibrium}$

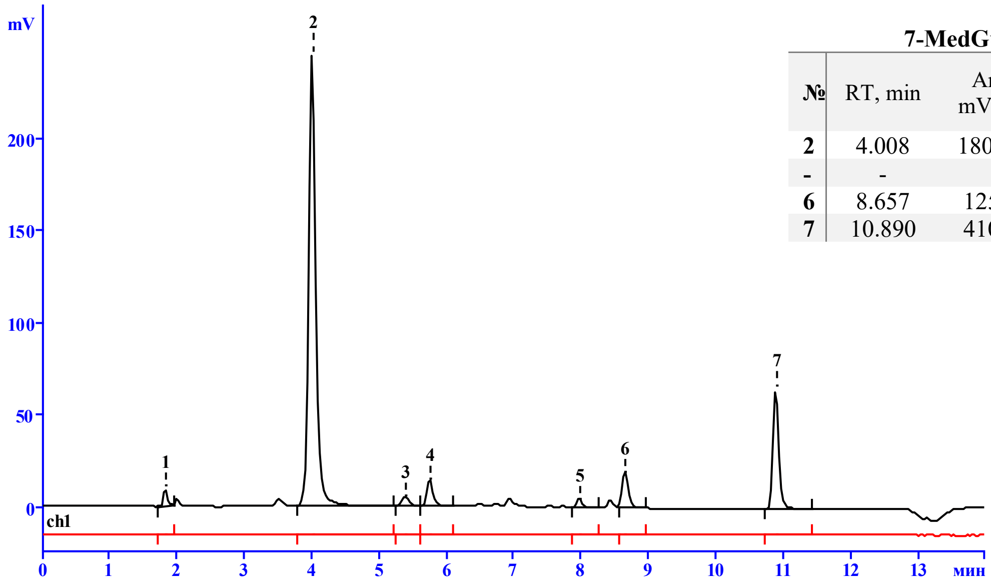

7-MedGuo+5-CF$_3$Ura – 3:1– t $_{equilibrium}$

№	RT, min	Area, mV*сек	Equilibrium concentration, µM	Name
**2**	4.008	1802.61	300.00	7-MeGua
**-**	-	-	-	7-MedGuo
**6**	8.657	125.18	13.90	5-CF$_3$Ura
**7**	10.890	410.60	86.10	5-CF3dUrd

<u>4-oxomethylpyrimidine-2'-deoxyriboside from 7-methyldeoxyguanosine (7-MedGuo+4-omPyr, analytical method)</u>

HPLC-analysis of transglycosylation: Cosmosil 5C18-MS-II, 4.6*150 mm, 5µm, 120 Å, (Nacalai Tesque, Inc. (Japan)), 20ºC, elution in modified mobile phase: linear gradient of acetonitrile in 0.06% TFA from 2 to 8% for 10 min (flushing with 8-80 % acetonitrile-0.06% TFA for 10-10.1 min, then 80-2% for 10.1-10.8 min) at flow rate 1 ml/min with UV detection at wavelength 268 nm, injection volume 20 µL.

Fugure S12.1. <u>pH = 5.5</u>, 7.8*10^{-3} U *LlNDT II*

$t_{initial}$

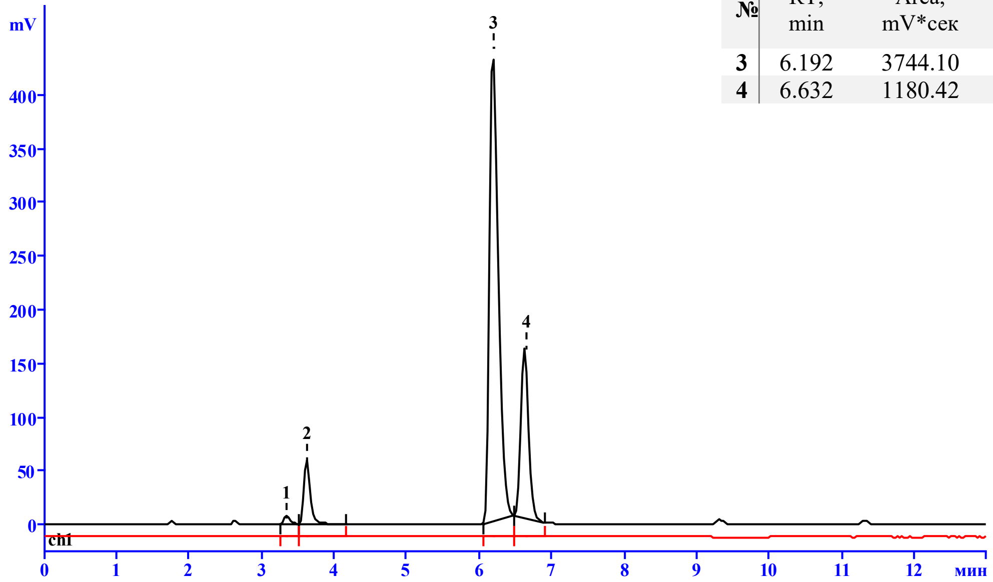

7-MedGuo+4-omPyr – 3:1 – t$_0$

№	RT, min	Area, mV*сек	Equilibrium concentration, µM	Name
**3**	6.192	3744.10	100.00	7-MedGuo
**4**	6.632	1180.42	300.00	4-omPyr

tequilibrium

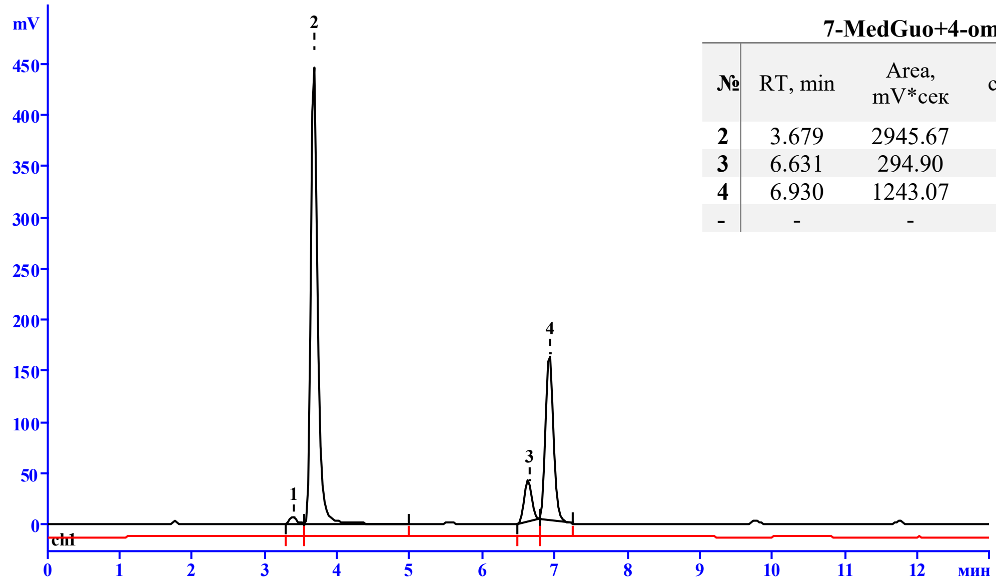

7-MedGuo+4-omPyr – 3:1– t equilibrium

№	RT, min	Area, mV*сек	Equilibrium concentration, µM	Name
2	3.679	2945.67	276.37	7-MeGua
3	6.631	294.90	23.63	7-MedGuo
4	6.930	1243.07	100.00	4-omPyr
-	-	-	0.00	4-omdPyr

Fugure S12.2. pH = 7.0, 7.8*10^{-3} U *LlNDT II*

tinitial

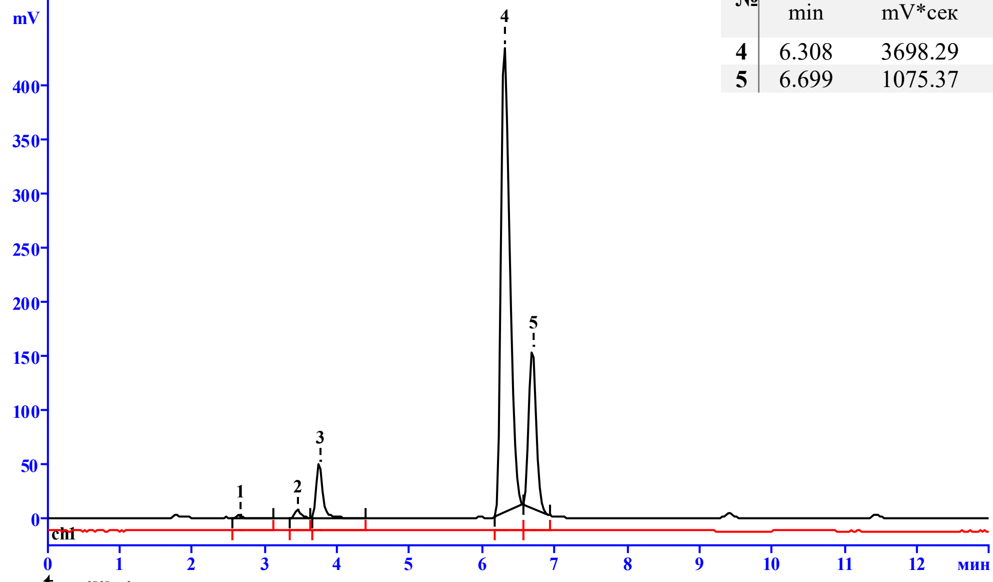

7-MedGuo+4-omPyr – 3:1 – t0

№	RT, min	Area, mV*сек	Equilibrium concentration, µM	Name
4	6.308	3698.29	100.00	7-MedGuo
5	6.699	1075.37	300.00	4-omPyr

tequilibrium

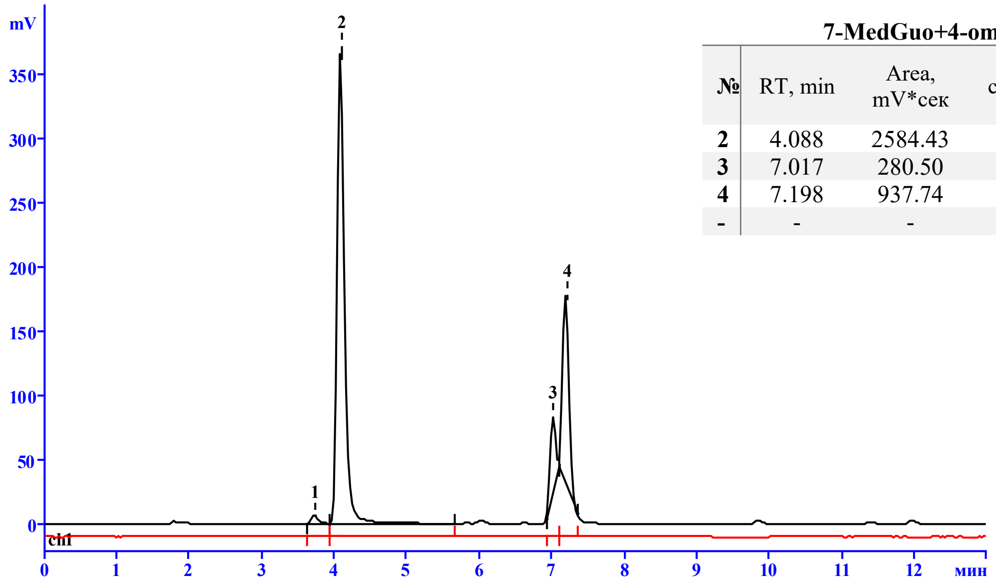

7-MedGuo+4-omPyr – 3:1– t equilibrium

№	RT, min	Area, mV*сек	Equilibrium concentration, µM	Name
2	4.088	2584.43	277.25	7-MeGua
3	7.017	280.50	22.75	7-MedGuo
4	7.198	937.74	100.00	4-omPyr
-	-	-	0.00	4-omdPyr

Fugure S12.3. <u>pH = 8.5</u>, 7.8*10^{-3} U *LlNDT II*

$t_{initial}$

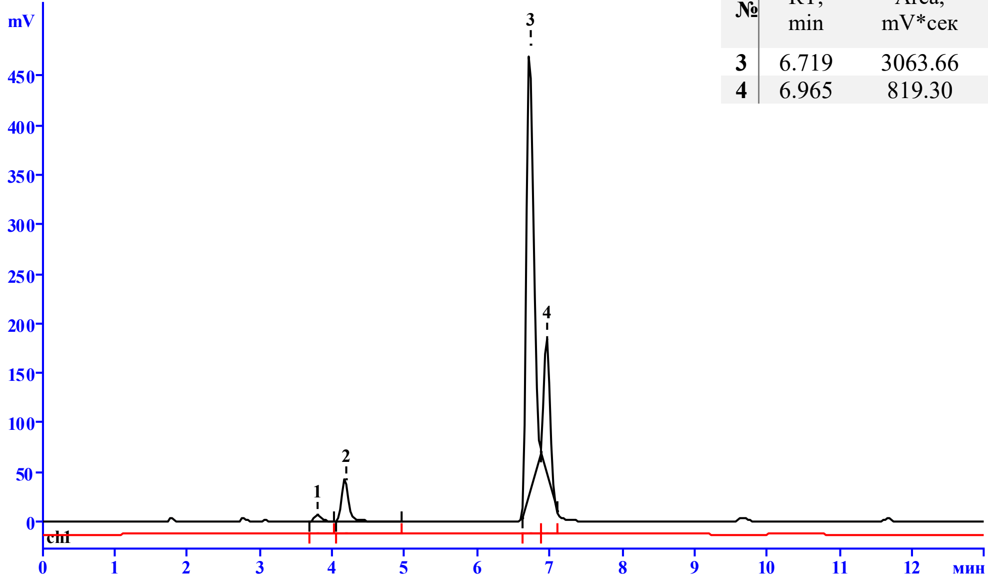

7-MedGuo+4-omPyr – 3:1 – t$_0$

№	RT, min	Area, mV*сек	Equilibrium concentration, µM	Name
**3**	6.719	3063.66	100.00	7-MedGuo
**4**	6.965	819.30	300.00	4-omPyr

$t_{equilibrium}$

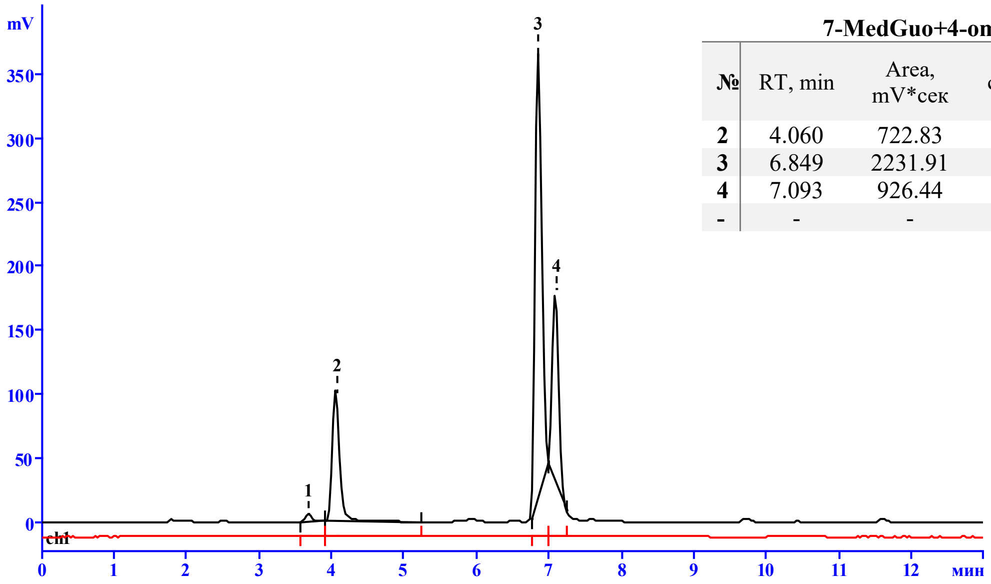

7-MedGuo+4-omPyr – 3:1– t $_{equilibrium}$

№	RT, min	Area, mV*сек	Equilibrium concentration, µM	Name
**2**	4.060	722.83	81.45	7-MeGua
**3**	6.849	2231.91	218.55	7-MedGuo
**4**	7.093	926.44	100.00	4-omPyr
**-**	-	-	0.00	4-omdPyr

<u>2´-deoxy-5-bromuridine, 2´-deoxy-5-fluorouridine from 7-methyldeoxyguanosine (7-MedGuo+5-BrUra, 5-FUra, analytical method)</u>

HPLC-analysis of transglycosylation: Synergi Hydro-RP, 4.6*150 mm, 4µm, 80 Å, (Nacalai Tesque, Inc. (Japan)), 20°C, elution in modified mobile phase: linear gradient of acetonitrile in 0.06% TFA from 2 to 12% for 10 min (flushing with 12-80 % acetonitrile-0.06% TFA for 10-10.1 min, then 80-2% for 10.1-10.8 min) at flow rate 1 ml/min with UV detection at wavelength 275 nm, injection volume 20 µL.

Fugure S13.1. pH = 5.5, 7.8*10^{-3} U *LlNDT II*

a) t$_{initial}$

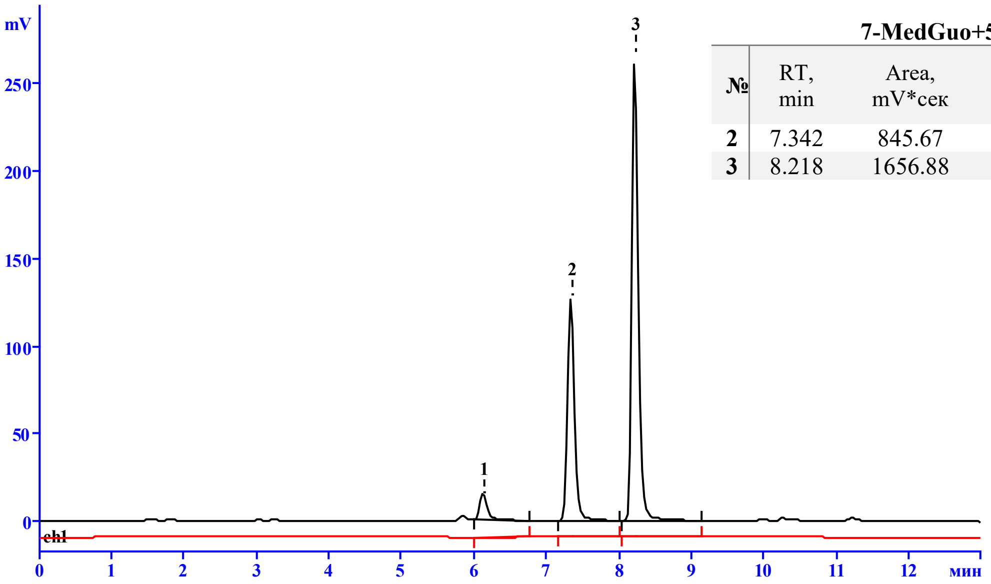

7-MedGuo+5-BrUra – 3:1 – t$_0$

№	RT, min	Area, mV*сек	Equilibrium concentration, µM	Name
2	7.342	845.67	100.00	5-BrUra
3	8.218	1656.88	300.00	7-MedGuo

t$_{equilibrium}$

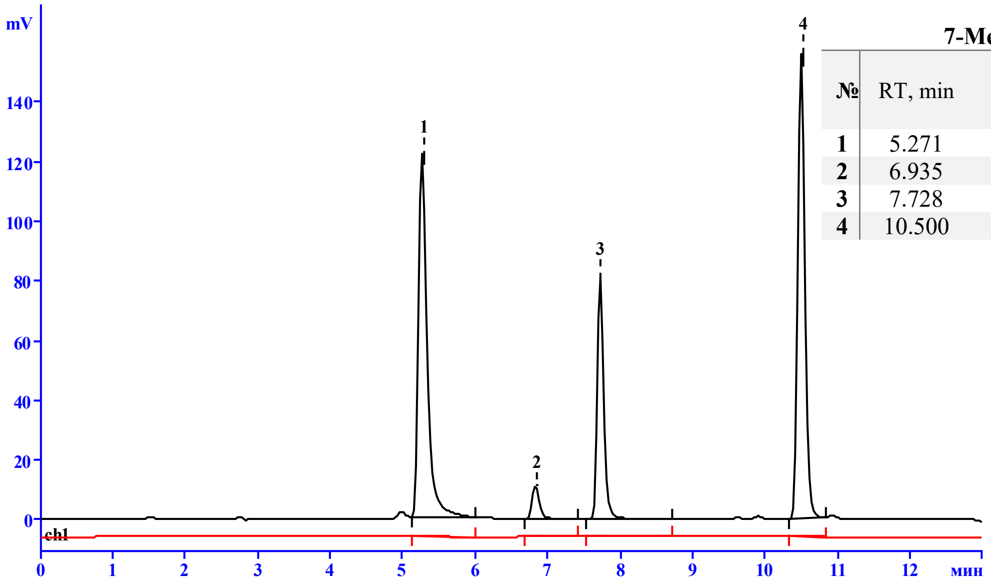

7-MedGuo+5-BrUra – 3:1– t $_{equilibrium}$

№	RT, min	Area, mV*сек	Equilibrium concentration, µM	Name
1	5.271	1032.45	207.15	7-MeGua
2	6.935	80.10	9.47	5-BrUra
3	7.728	512.82	92.85	7-MedGuo
4	10.500	1074.06	90.53	5-BrdUrd

b) t$_{initial}$

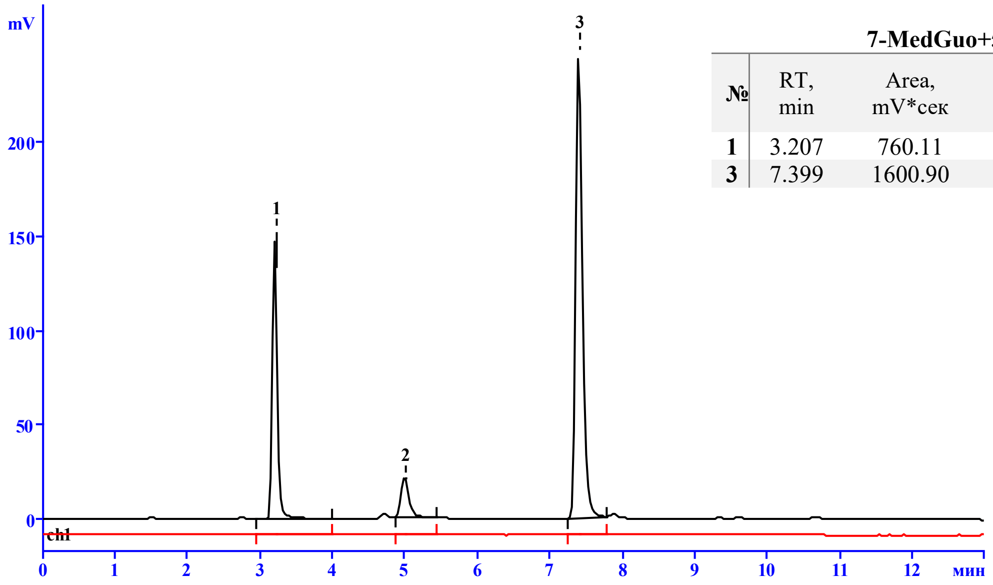

7-MedGuo+5-FUra – 3:1 – t$_0$

№	RT, min	Area, mV*сек	Equilibrium concentration, µM	Name
1	3.207	760.11	100.00	5-FUra
3	7.399	1600.90	300.00	7-MedGuo

tequilibrium

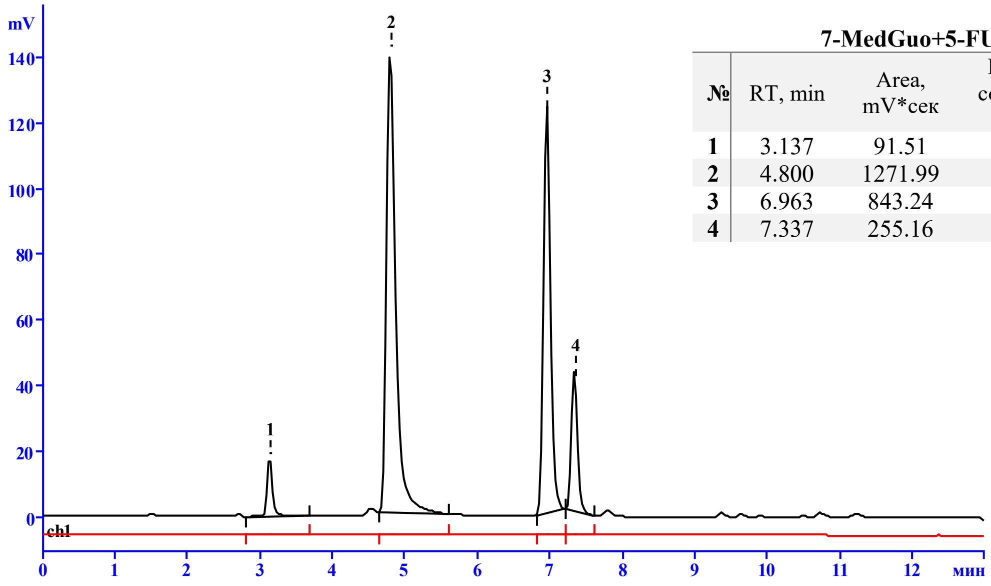

7-MedGuo+5-FUra – 3:1– t equilibrium

№	RT, min	Area, mV*сек	Equilibrium concentration, µM	Name
1	3.137	91.51	12.04	5-FUra
2	4.800	1271.99	252.18	7-MeGua
3	6.963	843.24	87.96	5-FdUrd
4	7.337	255.16	47.82	7-MedGuo

Fugure S13.2. <u>pH = 7.0</u>, 7.8*10^{-3} U *LlNDT II*

a) tinitial

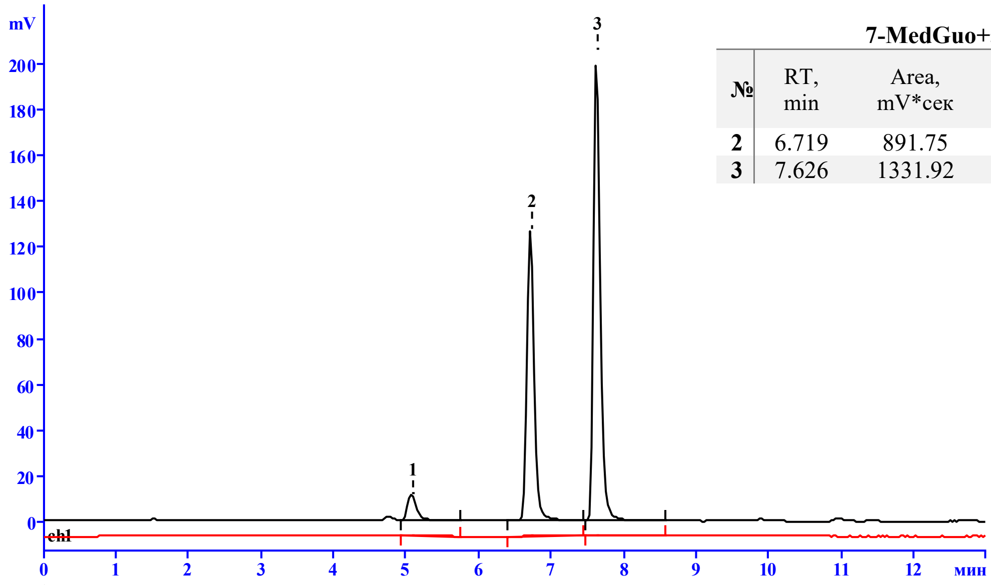

7-MedGuo+5-BrUra – 3:1 – t₀

№	RT, min	Area, mV*сек	Equilibrium concentration, µM	Name
2	6.719	891.75	100.00	5-BrUra
3	7.626	1331.92	300.00	7-MedGuo

tequilibrium

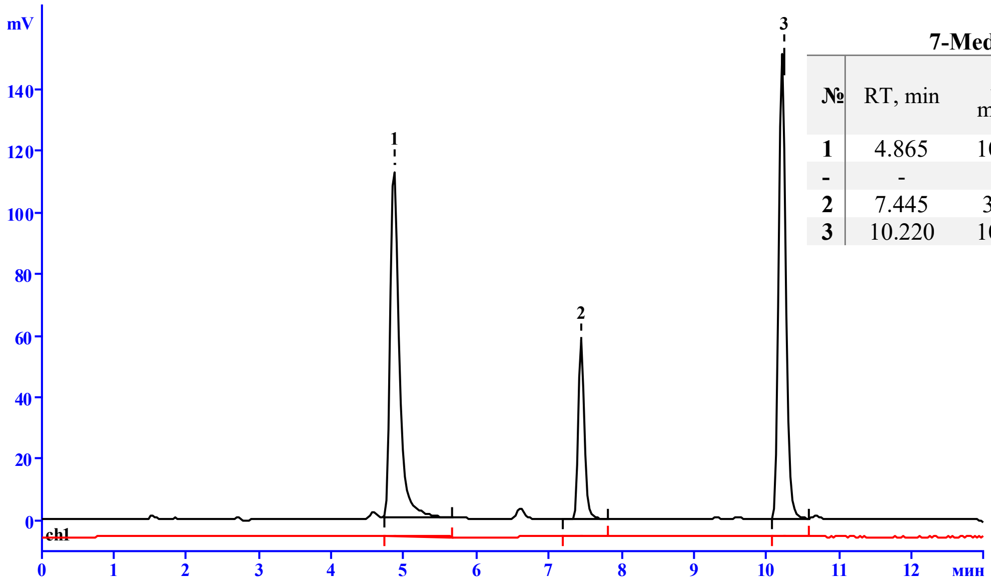

7-MedGuo+5-BrUra – 3:1– t equilibrium

№	RT, min	Area, mV*сек	Equilibrium concentration, µM	Name
1	4.865	1011.86	222.01	7-MeGua
-	-	-	-	5-BrUra
2	7.445	346.24	77.99	7-MedGuo
3	10.220	1051.76	100.00	5-BrdUrd

b) $t_{initial}$

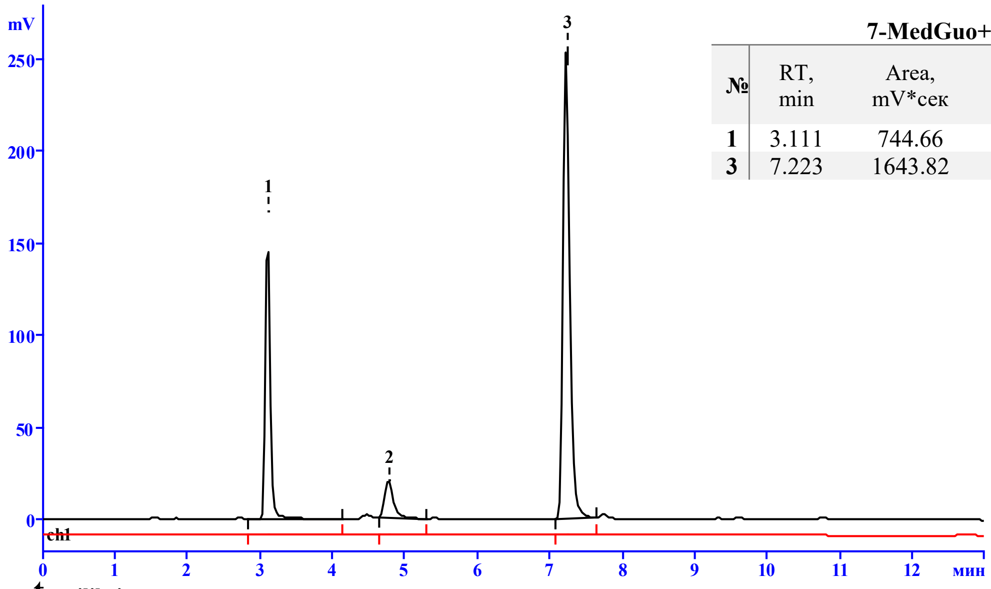

7-MedGuo+5-FUra – 3:1 – t_0

№	RT, min	Area, mV*сек	Equilibrium concentration, µM	Name
**1**	3.111	744.66	100.00	5-FUra
**3**	7.223	1643.82	300.00	7-MedGuo

$t_{equilibrium}$

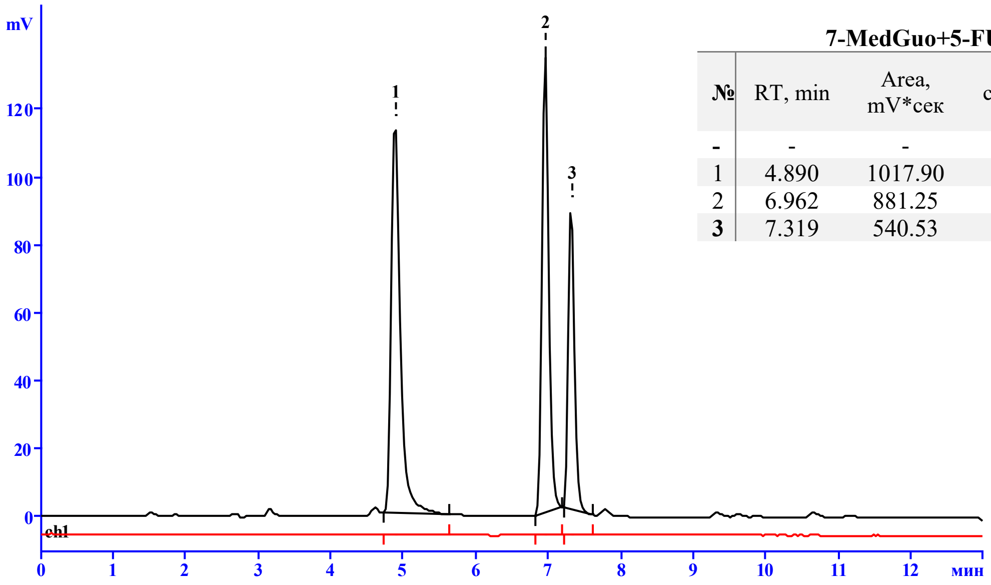

7-MedGuo+5-FUra – 3:1– t $_{equilibrium}$

№	RT, min	Area, mV*сек	Equilibrium concentration, µM	Name
**-**	-	-	-	5-FUra
1	4.890	1017.90	201.35	7-MeGua
2	6.962	881.25	100.00	5-FdUrd
**3**	7.319	540.53	98.65	7-MedGuo

Fugure S13.3. pH = 8.5

1) 7.8*10^{-3} U *LlNDT II*

a) $t_{initial}$

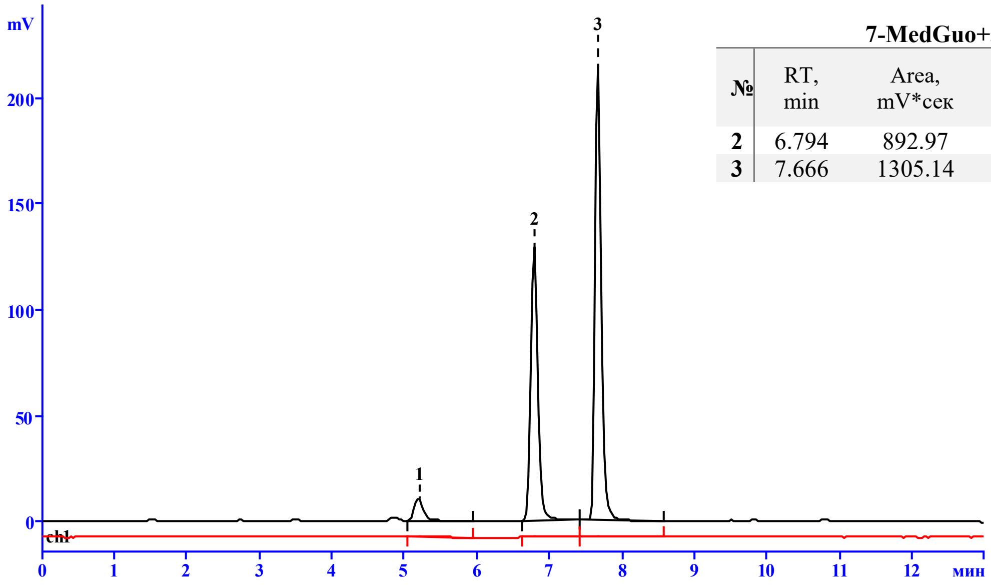

7-MedGuo+5-BrUra – 3:1 – t_0

№	RT, min	Area, mV*сек	Equilibrium concentration, µM	Name
**2**	6.794	892.97	100.00	5-BrUra
**3**	7.666	1305.14	300.00	7-MedGuo

$t_{equilibrium}$

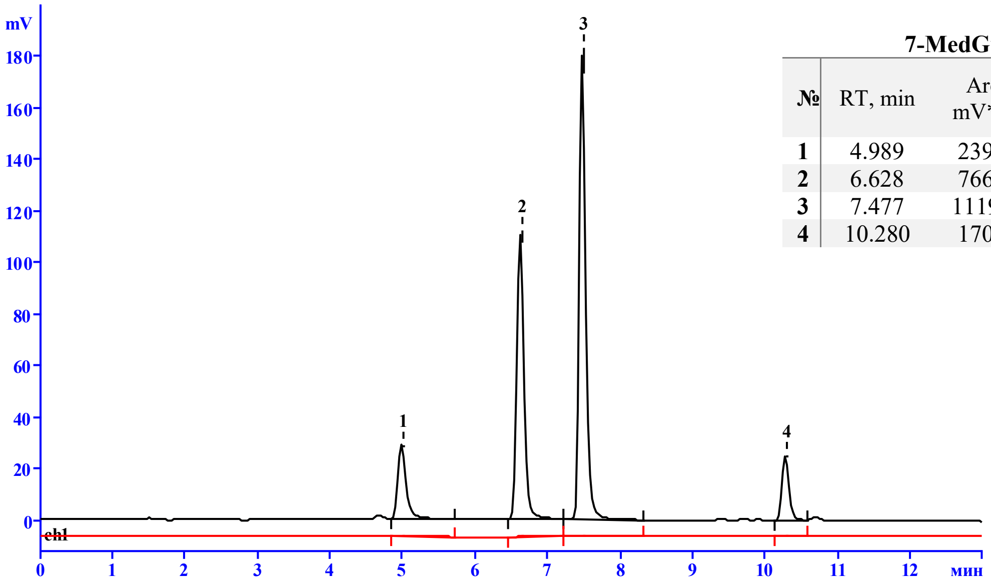

7-MedGuo+5-BrUra – 3:1– $t_{equilibrium}$

№	RT, min	Area, mV*сек	Equilibrium concentration, µM	Name
1	4.989	239.05	42.58	7-MeGua
2	6.628	766.13	85.80	5-BrUra
3	7.477	1119.91	257.42	7-MedGuo
4	10.280	170.82	14.20	5-BrdUrd

b) $t_{initial}$

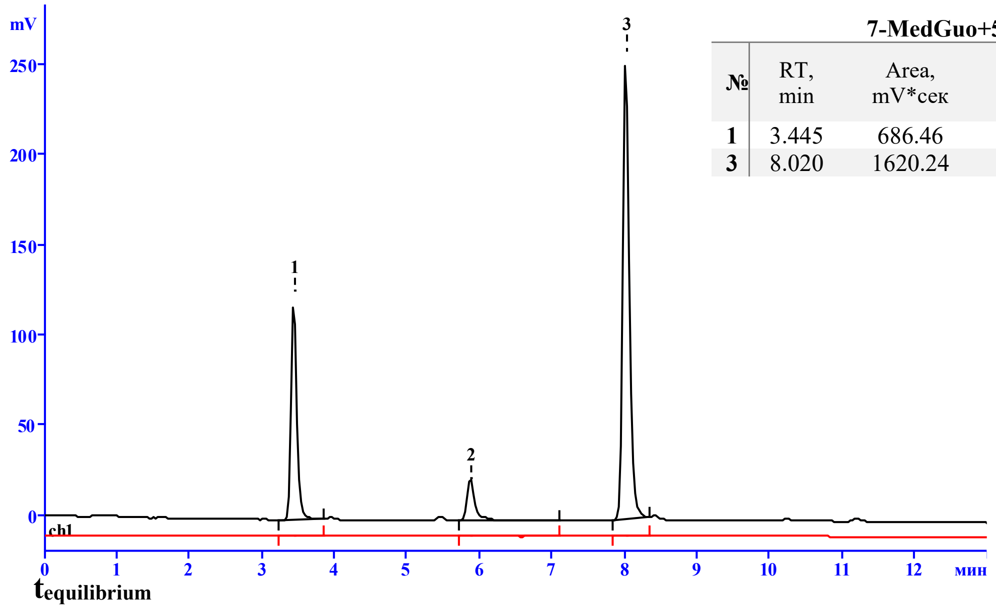

7-MedGuo+5-FUra – 3:1 – t_0

№	RT, min	Area, mV*сек	Equilibrium concentration, µM	Name
1	3.445	686.46	100.00	5-FUra
3	8.020	1620.24	300.00	7-MedGuo

$t_{equilibrium}$

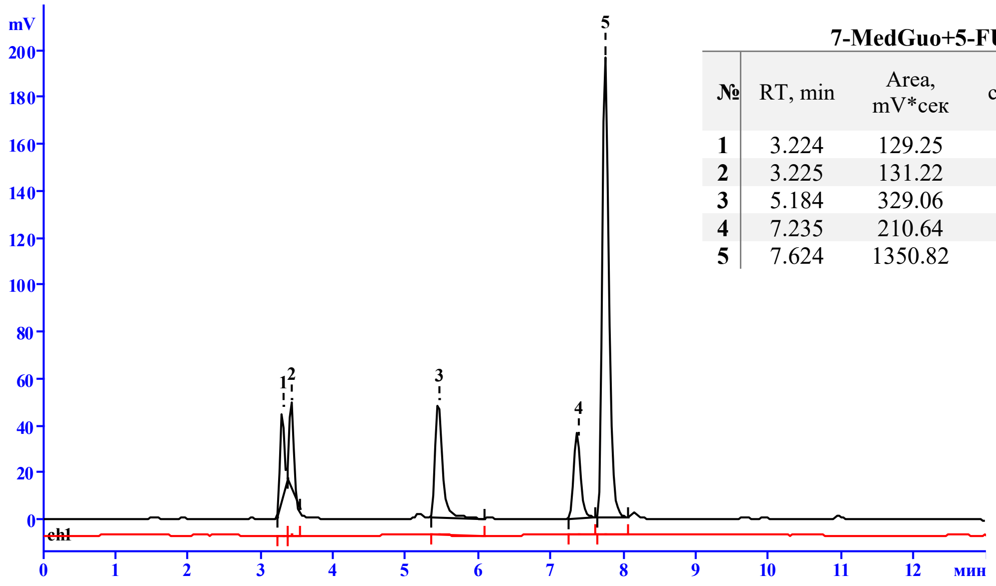

7-MedGuo+5-FUra – 3:1– $t_{equilibrium}$

№	RT, min	Area, mV*сек	Equilibrium concentration, µM	Name
1	3.224	129.25	36.72	5-FUra
2	3.225	131.22	37.28	5-FUra
3	5.184	329.06	49.89	7-MeGua
4	7.235	210.64	26.00	5-FdUrd
5	7.624	1350.82	250.11	7-MedGuo

2) 26*10^{-3} U *LlNDT II*

a) $t_{initial}$

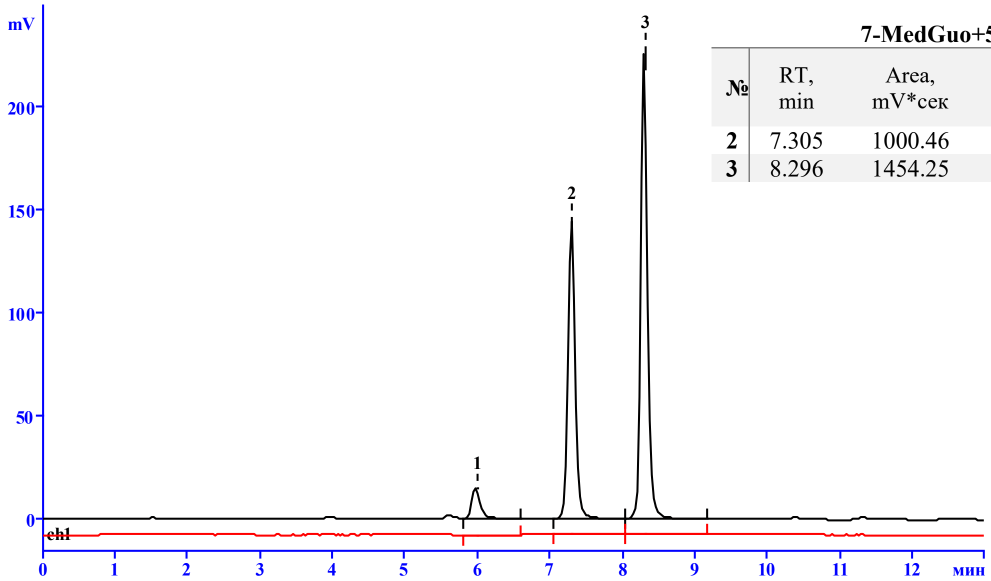

7-MedGuo+5-BrUra – 3:1 – t_0

№	RT, min	Area, mV*сек	Equilibrium concentration, µM	Name
**2**	7.305	1000.46	100.00	5-BrUra
**3**	8.296	1454.25	300.00	7-MedGuo

$t_{equilibrium}$

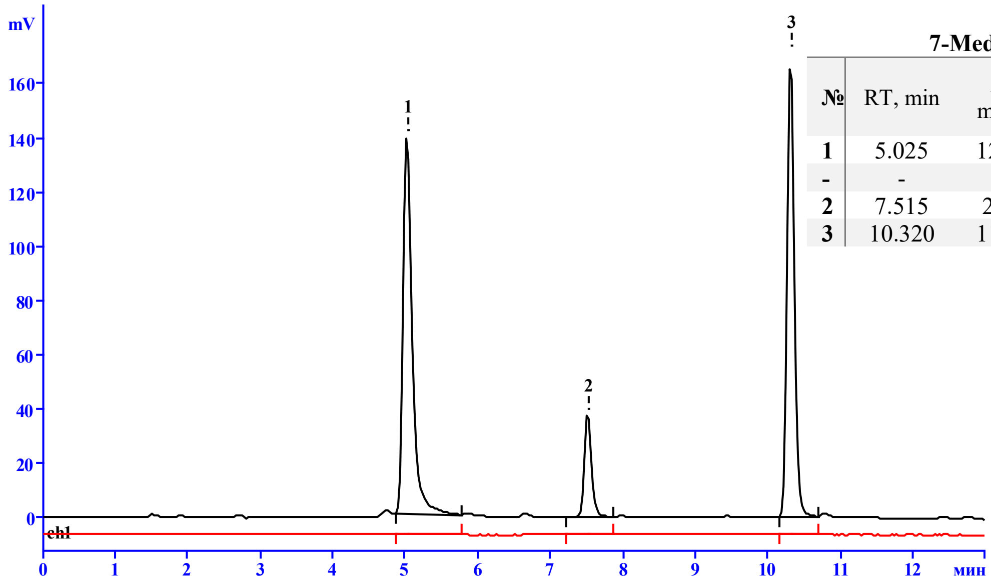

7-MedGuo+5-BrUra – 3:1– $t_{equilibrium}$

№	RT, min	Area, mV*сек	Equilibrium concentration, µM	Name
**1**	5.025	1247.16	245.95	7-MeGua
**-**	-	-	-	5-BrUra
**2**	7.515	261.99	54.05	7-MedGuo
**3**	10.320	1189.75	100.00	5-BrdUrd

b) $t_{initial}$

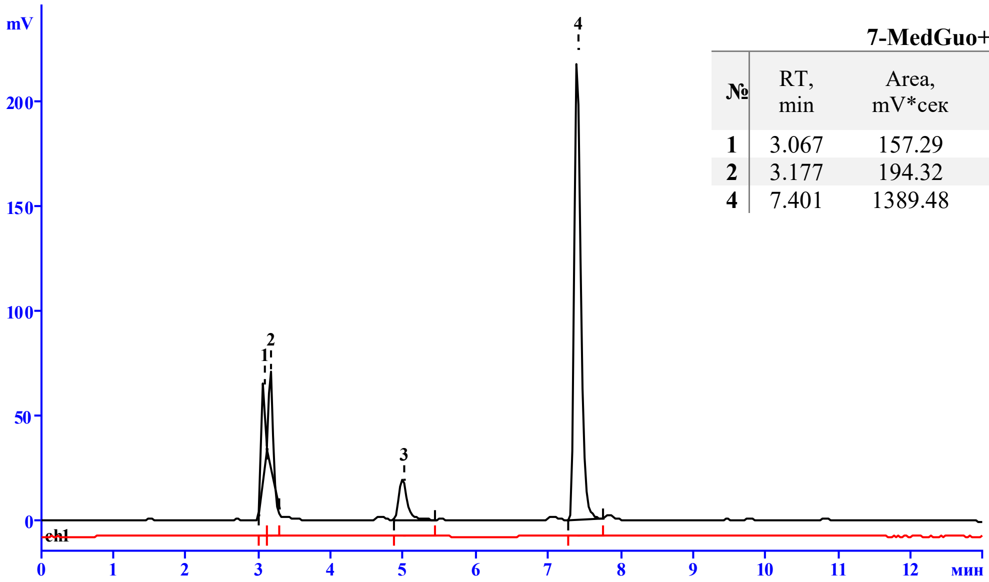

7-MedGuo+5-FUra – 3:1 – t_0

№	RT, min	Area, mV*сек	Equilibrium concentration, µM	Name
**1**	3.067	157.29	44.73	5-FUra
**2**	3.177	194.32	55.27	5-FUra
**4**	7.401	1389.48	300.00	7-MedGuo

t$_{equilibrium}$

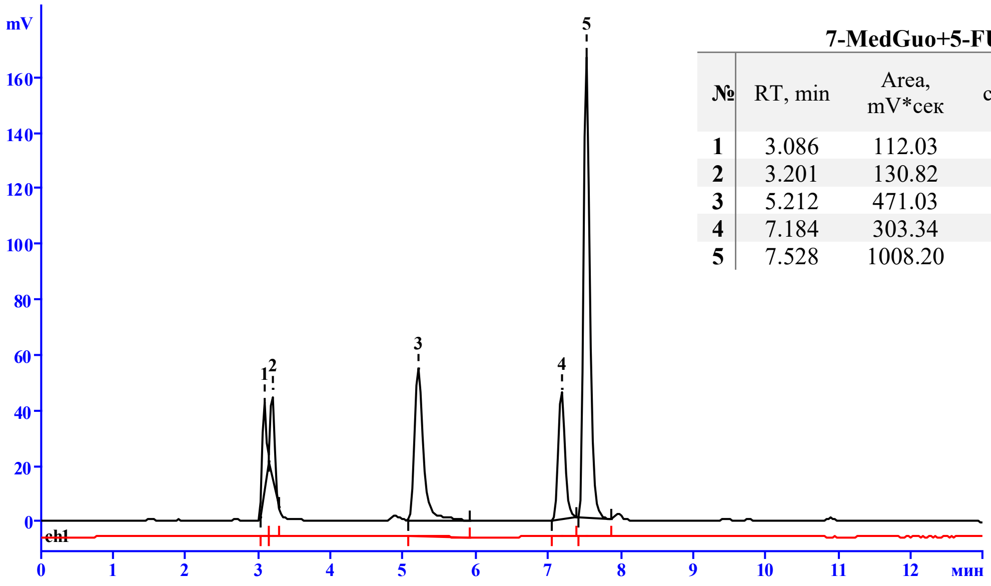

7-MedGuo+5-FUra – 3:1– t $_{equilibrium}$

№	RT, min	Area, mV*сек	Equilibrium concentration, µM	Name
1	3.086	112.03	31.83	5-FUra
2	3.201	130.82	37.17	5-FUra
3	5.212	471.03	82.32	7-MeGua
4	7.184	303.34	31.00	5-FdUrd
5	7.528	1008.20	217.68	7-MedGuo

3) 52*10^{-3} U *LlNDT II*

t$_{initial}$

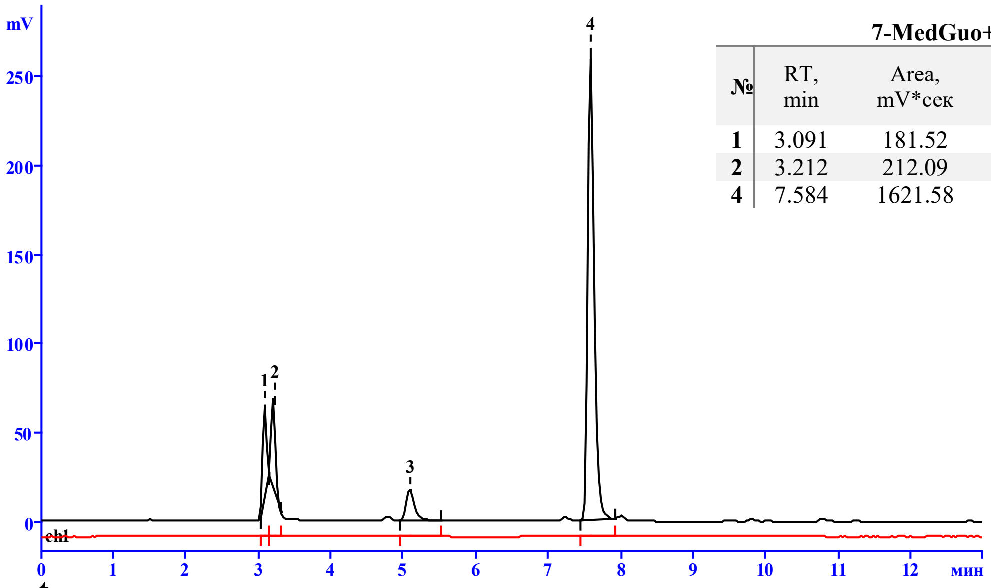

7-MedGuo+5-FUra – 3:1 – t$_0$

№	RT, min	Area, mV*сек	Equilibrium concentration, µM	Name
1	3.091	181.52	46,12	5-FUra
2	3.212	212.09	53.88	5-FUra
4	7.584	1621.58	300.00	7-MedGuo

t$_{equilibrium}$

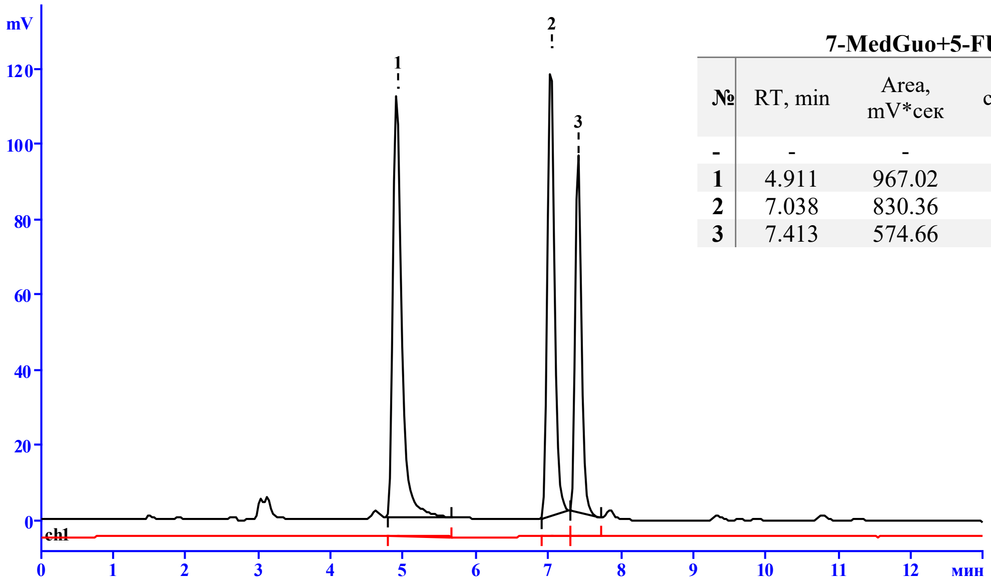

7-MedGuo+5-FUra – 3:1– t $_{equilibrium}$

№	RT, min	Area, mV*сек	Equilibrium concentration, µM	Name
-	-	-	-	5-FUra
1	4.911	967.02	193.69	7-MeGua
2	7.038	830.36	100.00	5-FdUrd
3	7.413	574.66	106.31	7-MedGuo

__2′-deoxyadenosine from 7-methyldeoxyguanosine (7-MedGuo+Ade, analytical method)__

HPLC-analysis of transglycosylation: Synergi Hydro-RP, 4.6*150 mm, 4µm, 80 Å, (Nacalai Tesque, Inc. (Japan)), 20ºC, elution in modified mobile phase: linear gradient of acetonitrile in 0.06% TFA from 2 to 8% for 10 min (flushing with 8-80 % acetonitrile-0.06% TFA for 10-10.1 min, then 80-2% for 10.1-10.8 min) at flow rate 1 ml/min with UV detection at wavelength 260 nm, injection volume 20 µL.

Fugure S14.1. __pH = 5.5__, 7.8*10^{-3} U *LlNDT II*

$t_{initial}$

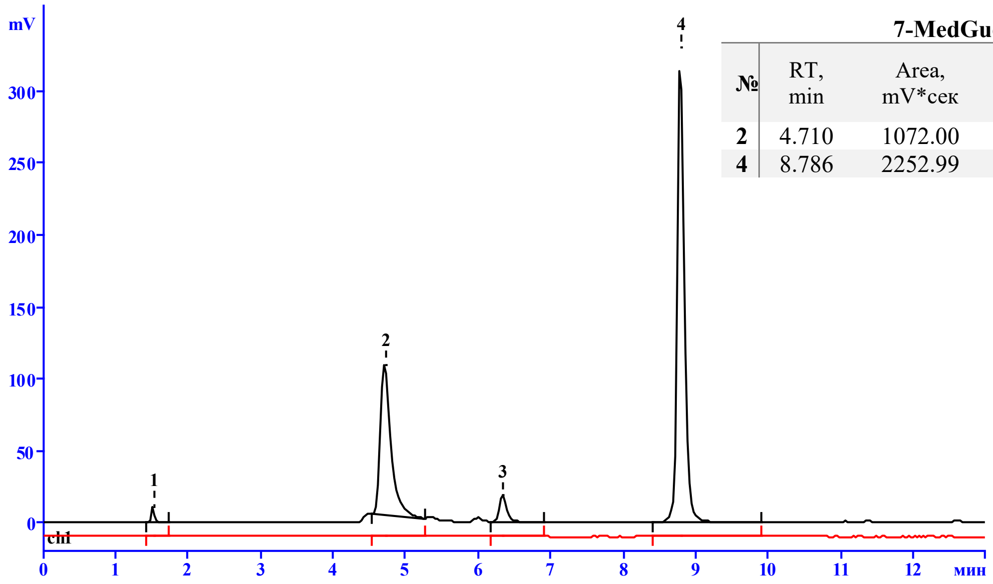

7-MedGuo+Ade – 3:1 – t_0

№	RT, min	Area, mV*сек	Equilibrium concentration, µM	Name
**2**	4.710	1072.00	100.00	Ade
**4**	8.786	2252.99	300.00	7-MedGuo

$t_{equilibrium}$

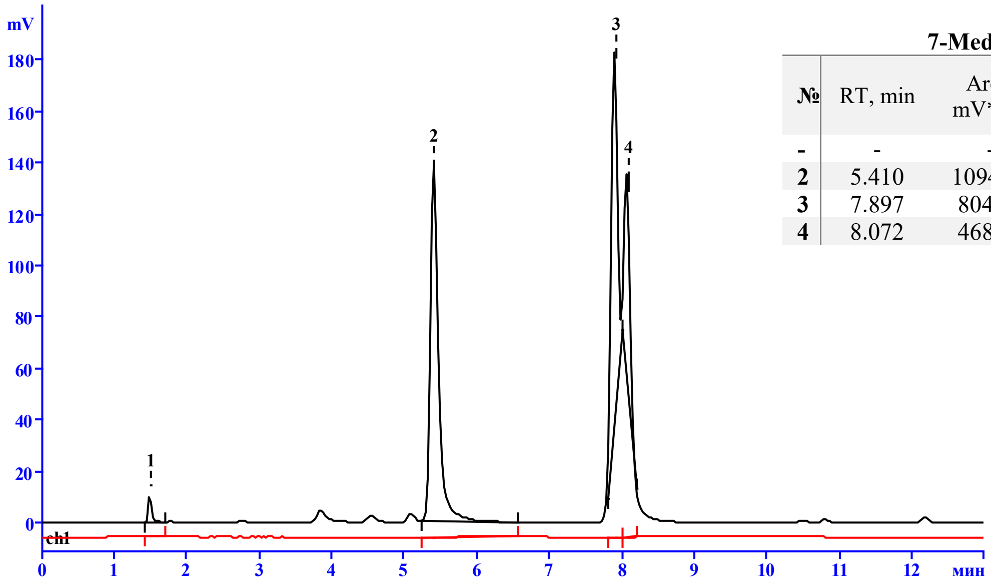

7-MedGuo+Ade – 3:1– $t_{equilibrium}$

№	RT, min	Area, mV*сек	Equilibrium concentration, µM	Name
**-**	-	-	-	Ade
**2**	5.410	1094.37	192.86	7-MeGua
**3**	7.897	804.61	107.14	7-MedGuo
**4**	8.072	468.21	100.00	Ado

Fugure S14.2. pH = 7.0, $7.8*10^{-3}$ U *LlNDT II*

tinitial

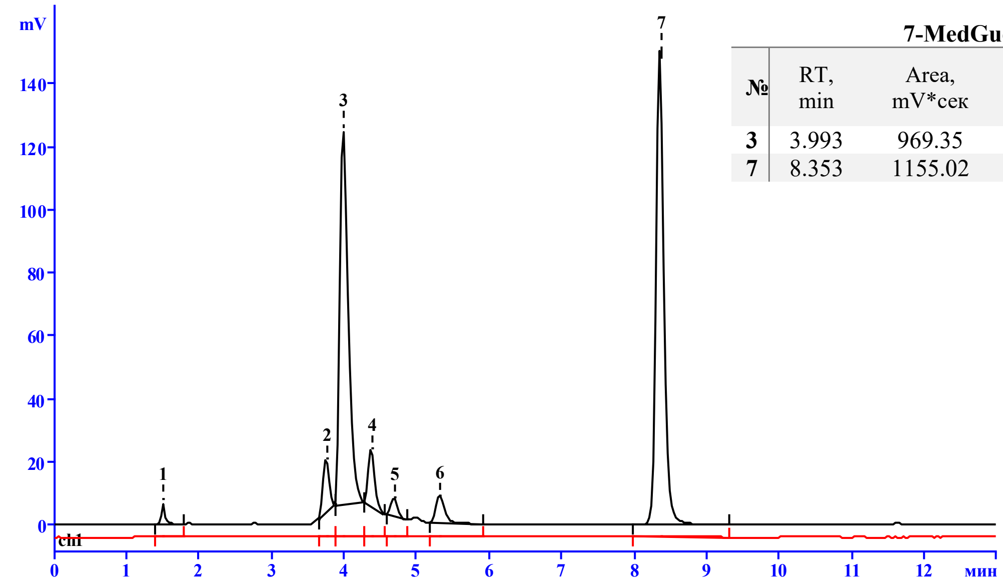

7-MedGuo+Ade – 1:1 – t_0

№	RT, min	Area, mV*сек	Equilibrium concentration, µM	Name
**3**	3.993	969.35	200.00	Ade
**7**	8.353	1155.02	200.00	7-MedGuo

tequilibrium

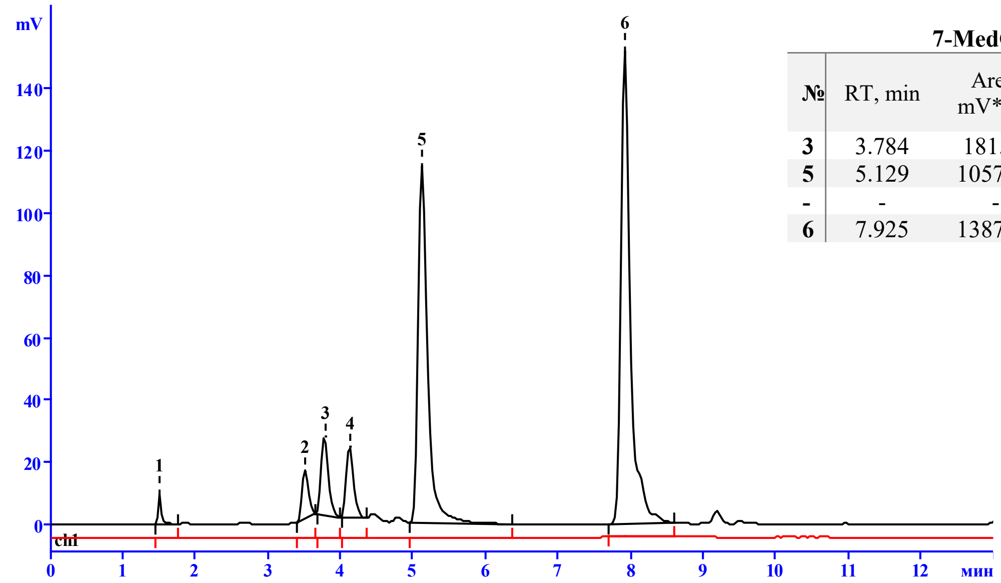

7-MedGuo+Ade – 1:1– t $_{equilibrium}$

№	RT, min	Area, mV*сек	Equilibrium concentration, µM	Name
**3**	3.784	181.22	37.39	Ade
**5**	5.129	1057.89	200.00	7-MeGua
**-**	-	-	-	7-MedGuo
**6**	7.925	1387.59	162.61	Ado

tinitial

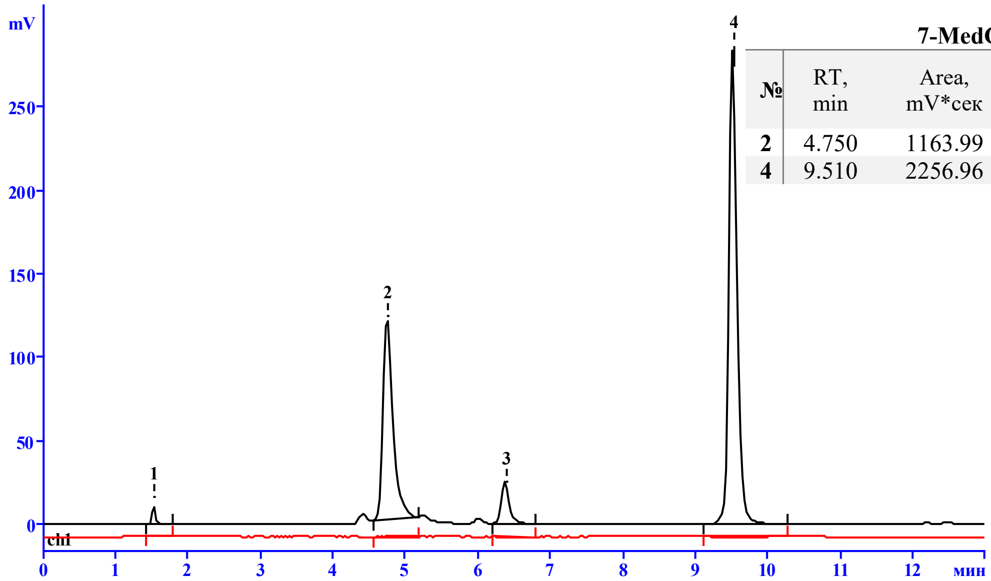

7-MedGuo+Ade – 3:1 – t_0

№	RT, min	Area, mV*сек	Equilibrium concentration, µM	Name
**2**	4.750	1163.99	100.00	Ade
**4**	9.510	2256.96	300.00	7-MedGuo

$t_{equilibrium}$

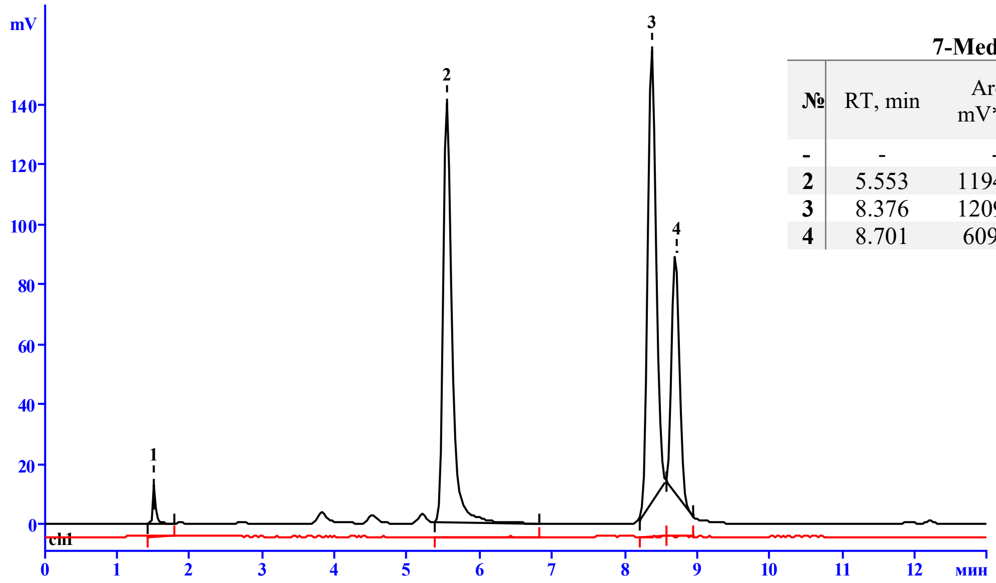

7-MedGuo+Ade – 3:1– t equilibrium

№	RT, min	Area, mV*сек	Equilibrium concentration, µM	Name
-	-	-	-	Ade
2	5.553	1194.60	139.18	7-MeGua
3	8.376	1209.86	160.82	7-MedGuo
4	8.701	609.63	100.00	Ado

Fugure S14.3. pH = 8.5, 7.8*10^{-3} U *LlNDT II*

$t_{initial}$

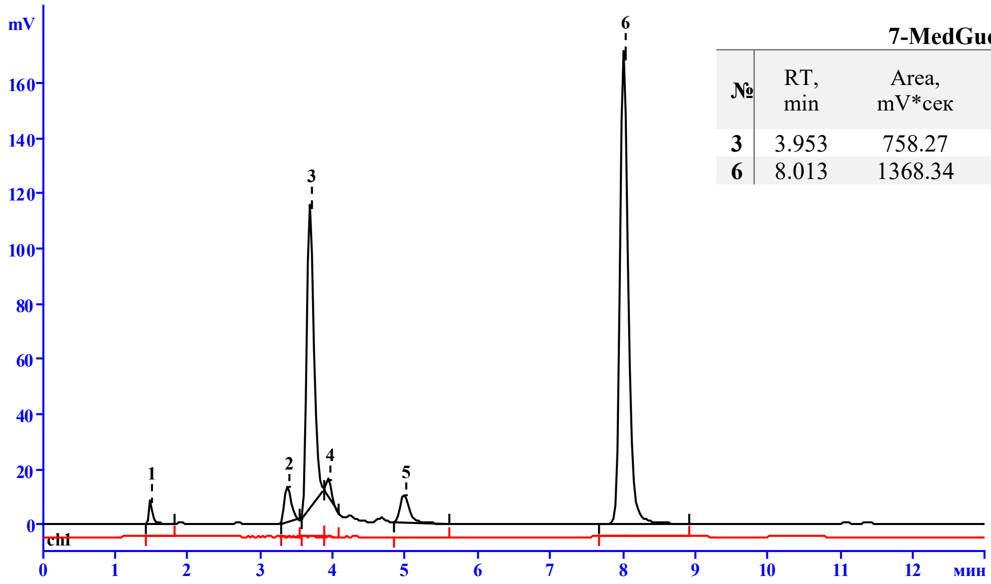

7-MedGuo+Ade – 3:1 – t_0

№	RT, min	Area, mV*сек	Equilibrium concentration, µM	Name
3	3.953	758.27	100.00	Ade
6	8.013	1368.34	300.00	7-MedGuo

$t_{equilibrium}$

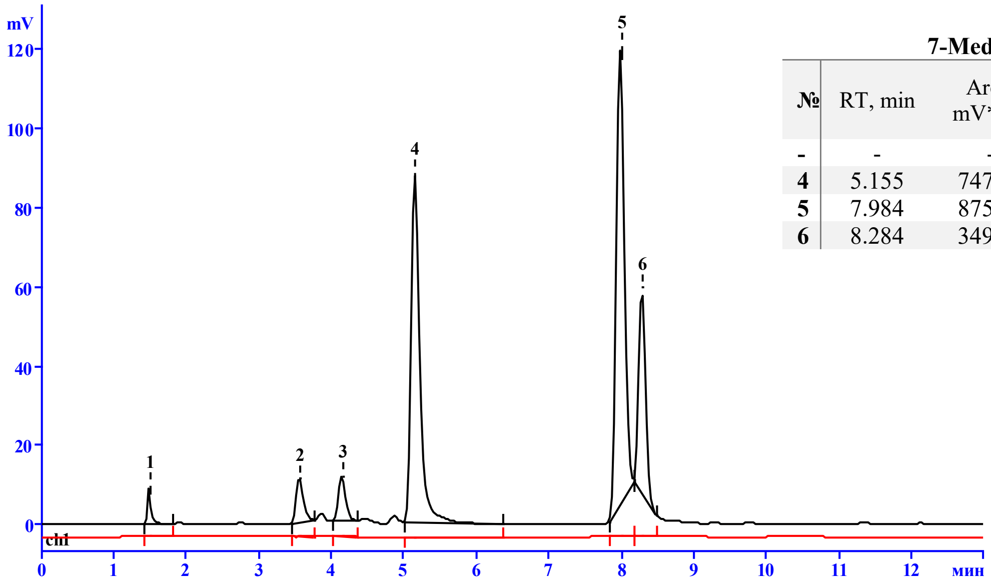

7-MedGuo+Ade – 3:1– t equilibrium

№	RT, min	Area, mV*сек	Equilibrium concentration, µM	Name
-	-	-	-	Ade
4	5.155	747.58	108.16	7-MeGua
5	7.984	875.02	191.84	7-MedGuo
6	8.284	349.66	100.00	Ado

Table S6. Purine transglycosylation reactions catalysed by NDT II *Lac. l.*[1].

Acceptor	Donor	Acceptor:Donor ratio	Yield, % (HPLC defined)[2]
Ade	7-mdGuo	1:1	81 %[4]
		1:3	100 %[3]
Hyp		1:1	54 %[4]
		1:3	100 %[3]
6-omPur		1:1	67 %[4]
		1:3	100 %[3]

[1] Reactions were carried out in 50 mM Tris-HCl, pH 7.0, r.t., *Lac. l.* NDT II, concentration of A:7-MedGuo 0.2 mM: 0.2 mM (1:1), 0.1 mM: 0.3 mM (1:3).

[2] The yield was calculated as the ratio of the equilibrium concentration of the target nucleoside to the initial concentration of the limiting substrate.

[3] No detectable peak signal of the heterocyclic base.

[4] No detectable peak signal of the 7-MedGuo (donor).

Table S7. Enzymatic synthesis of 5-hm-2′-dUrd using *Ll*NDTII at different pH values and enzyme concentrations. [a]

Entry	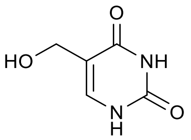	pH 5.5		7.0		8.5	
1	Enzyme, U	Time, min	Yield, %[b)]	Time, min	Yield, %[b)]	Time, min	Yield, %[b)]
2	$2.6 \cdot 10^{-3}$	1 d	57	1 d	55	–	–
3	$7.8 \cdot 10^{-3}$	70	74[c)]	170	84	–	–
4	$13 \cdot 10^{-3}$	50	75[c)]	100	85[c)]	160	14
5	$26 \cdot 10^{-3}$	35	70[c)]	80	82[c)]	1 d	87
6	$52 \cdot 10^{-3}$	15	74[c)]	35	81[c)]	7 h	82

[a] Reaction conditions: *Lac. l* NDT II., (0.1 mM Base: 0.3 mM 7-mdGuo (1:3)), 20°C, 50 mM NaOAc (pH 5.5); 50 mM Tris-HCl, (pH 7.0); 50 mM Tris-HCl, (pH 8.5). [b] The yield (HPLC) was calculated as the ratio of the target nucleoside equilibrium concentration to the limiting substrate initial concentration. [c] The yield is determined by the complete depletion of the donor.

Table S8. Enzymatic transglycosylation reactions of 5-hmUra and Thd, catalysed by NDT II *Lac. l.*, at different pH values[1].

Enzyme activity, U	pH	Time, min	Yield, % (HPLC defined)[2]
$2,6*10^{-3}$	5,5	1 d	62 %
	7,0	1 d	13 %
	8,5	–	–
$7,8*10^{-3}$	5,5	70	65 %
	7,0	180	74 %
	8,5	–	–

[1] Reactions were carried out in 50 мM NaOAc pH 5.5, 50 mM Tris-HCl pH 7.0/8,5, r.t., *Lac. l.* NDT II, concentration of 5-hmUra:Thd 0.1 mM: 0.3 mM (1:3).

[2] The yield was calculated as the ratio of the equilibrium concentration of the target nucleoside to the initial concentration of the limiting substrate.

<u>7-methylguanosine, 7-methyldeoxyguanosine, inosine, adenosine from 7-methylguanosine (7-MeGuo, 7-MedGuo, 7-MeGuo+Hyp, 7-MeGuo+Ade, analytical method)</u>

HPLC-analysis of transglycosylation: Cosmosil 5C18-MS-II, 4.6*150 mm, 5µm, 120 Å, (Nacalai Tesque, Inc. (Japan)), 37ºC, elution in modified mobile phase: linear gradient of acetonitrile in 0.06% TFA from 2 to 12% for 10 min (flushing with 12-80 % acetonitrile-0.06% TFA for 10-10.1 min, then 80-2% for 10.1-10.8 min) at flow rate 1 ml/min with UV detection at wavelength 260 nm, injection volume 20 µL.

Figure S15.

$t_{initial}$

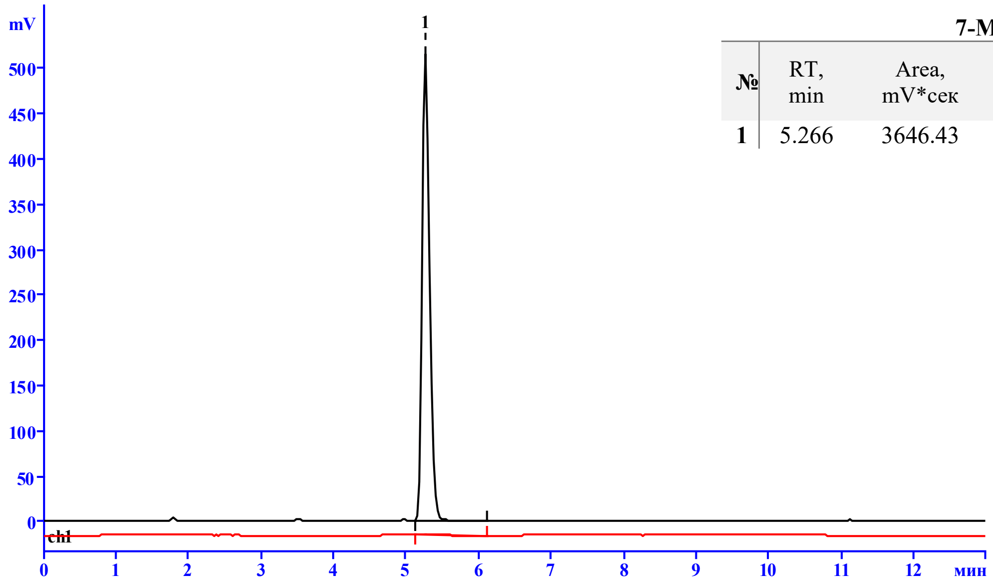

7-MeGuo – t_0

№	RT, min	Area, mV*сек	Equilibrium concentration, µM	Name
1	5.266	3646.43	300.00	7-MeGuo

1) **<u>pH = 5.5</u>**

$t_{initial+48h}$

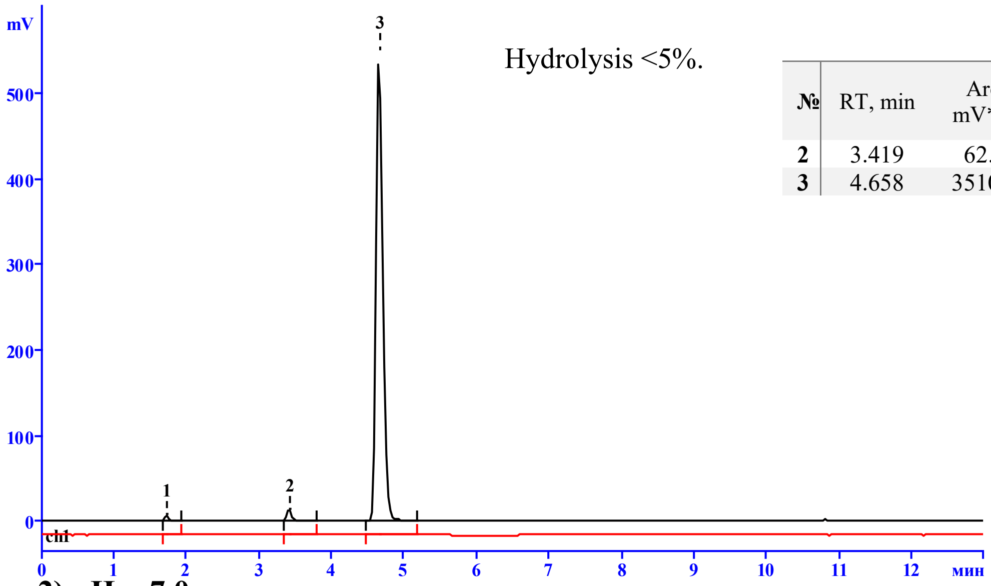

7-MeGuo – t_{0+48h}

№	RT, min	Area, mV*сек	Equilibrium concentration, µM	Name
**2**	3.419	62.73	11.22	7-MeGua
**3**	4.658	3510.05	288.78	7-MeGuo

2) **<u>pH = 7.0</u>**

$t_{initial+48h}$

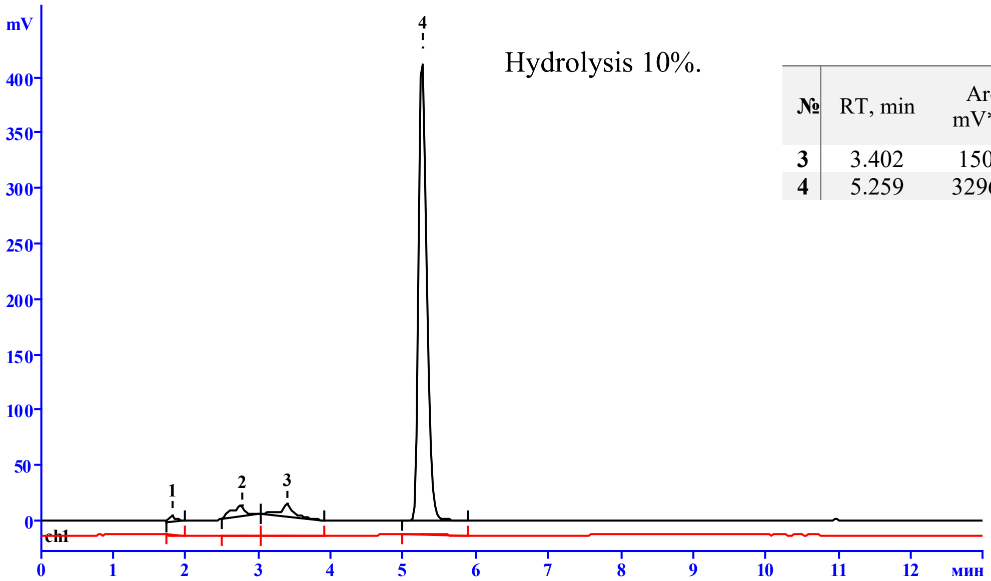

7-MeGuo – t_{0+48h}

№	RT, min	Area, mV*сек	Equilibrium concentration, µM	Name
**3**	3.402	150.43	14.78	7-MeGua
**4**	5.259	3296.90	271.24	7-MeGuo

3) **<u>pH = 8.5</u>**

$t_{initial+48h}$

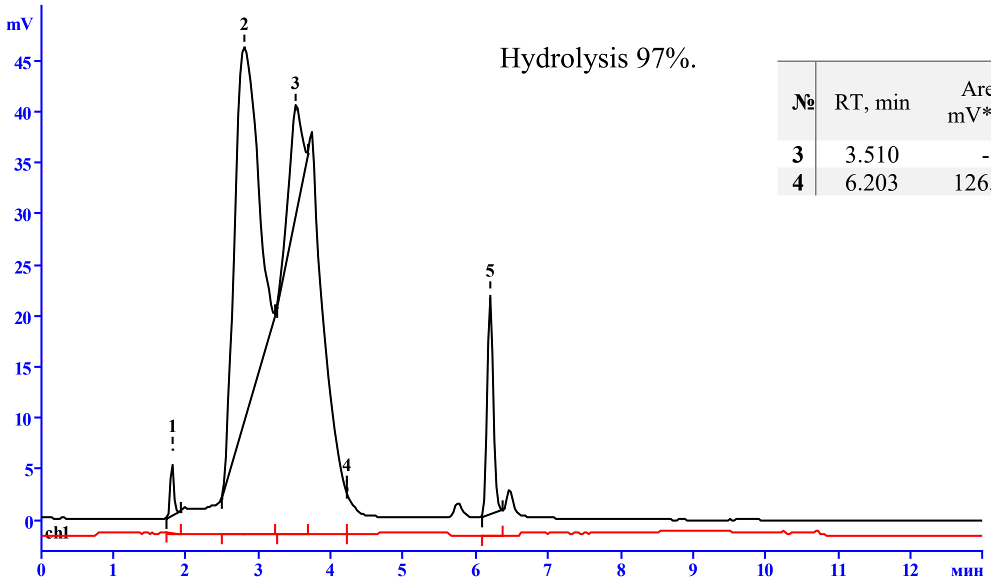

7-MeGuo – t_{0+48h}

№	RT, min	Area, mV*сек	Equilibrium concentration, µM	Name
**3**	3.510	-	-	7-MeGua
**4**	6.203	126.70	10.42	7-MeGuo

Figure S16.

$t_{initial}$

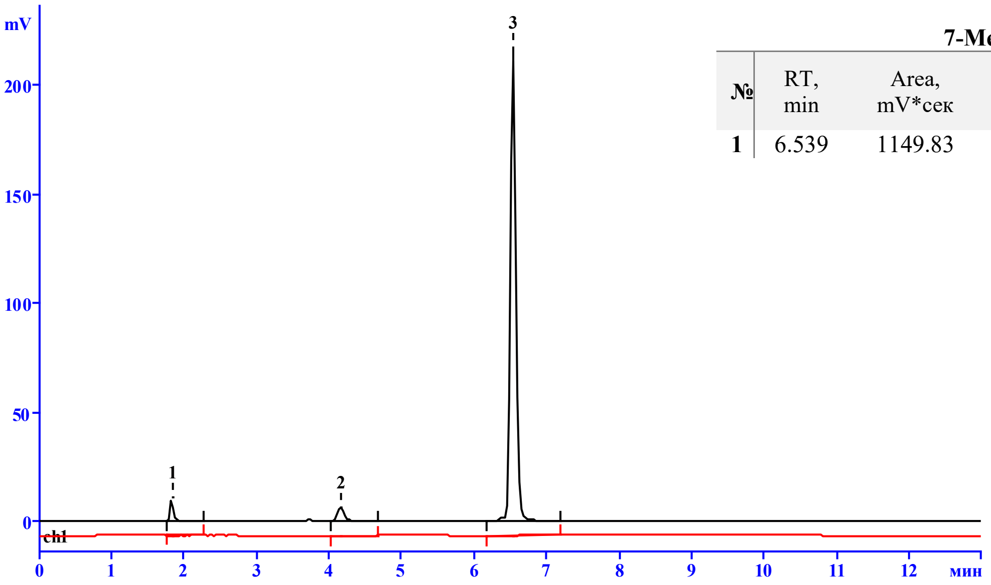

7-MedGuo – t_0

№	RT, min	Area, mV*сек	Equilibrium concentration, µM	Name
**1**	6.539	1149.83	300.00	7-MedGuo

1) <u>pH = 5.5</u>

$t_{initial+24h}$

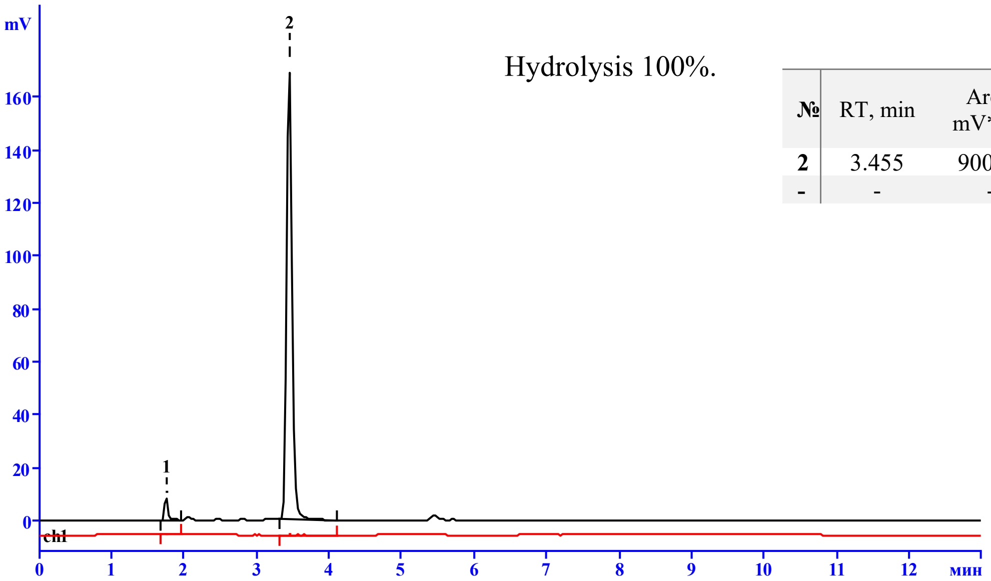

Hydrolysis 100%.

7-MedGuo – t_{0+24h}

№	RT, min	Area, mV*сек	Equilibrium concentration, µM	Name
**2**	3.455	900.88	300.00	7-MeGua
**-**	-	-	-	7-MedGuo

2) <u>pH = 7.0</u>

$t_{initial+24h}$

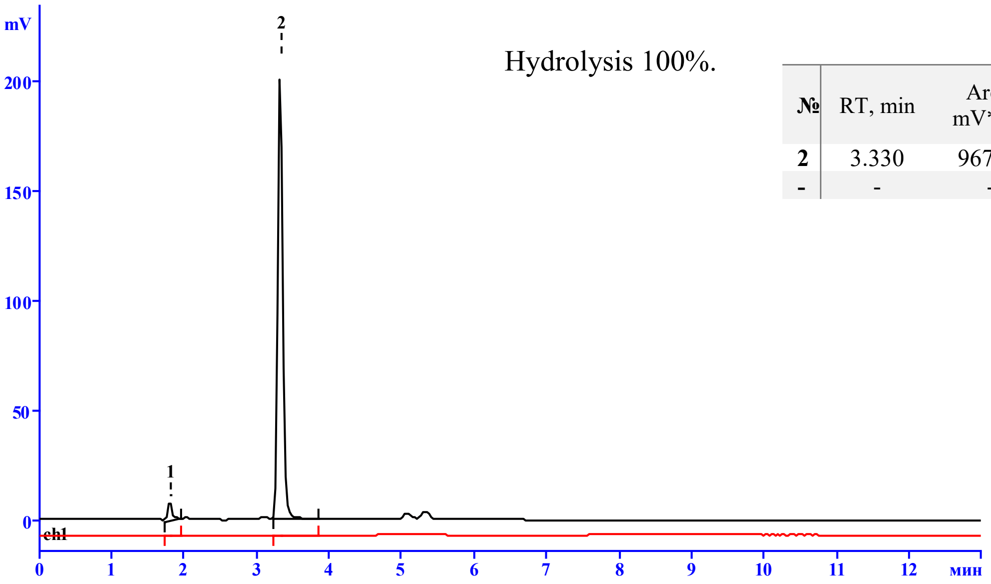

Hydrolysis 100%.

7-MedGuo – t_{0+24h}

№	RT, min	Area, mV*сек	Equilibrium concentration, µM	Name
**2**	3.330	967.73	300.00	7-MeGua
**-**	-	-	-	7-MedGuo

3) pH = 8.5

$t_{initial+24h}$

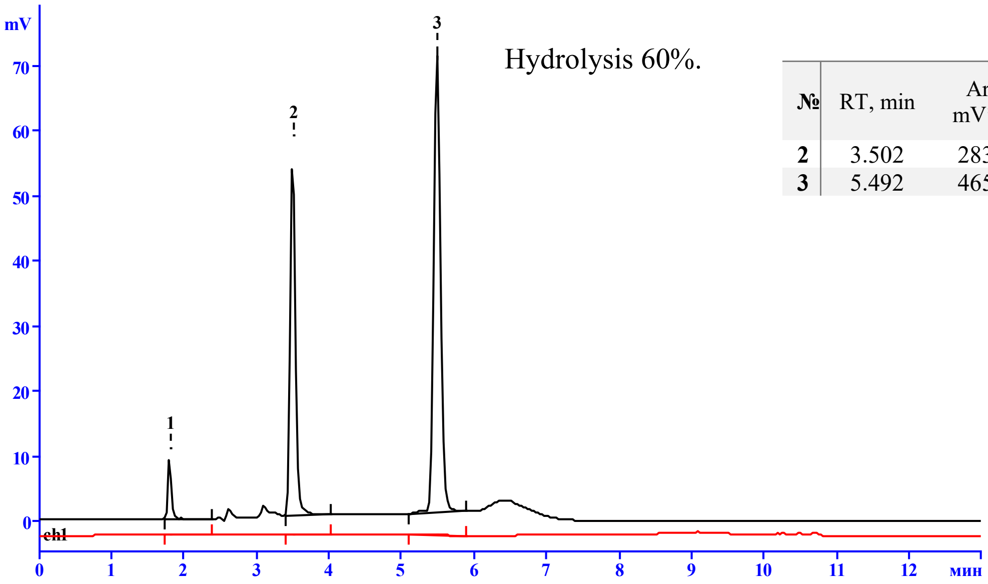

7-MedGuo – t_{0+24h}

№	RT, min	Area, mV*сек	Equilibrium concentration, µM	Name
2	3.502	283.58	178.53	7-MeGua
3	5.492	465.57	121.47	7-MedGuo

Figure S17. 50 мM citrate buffer pH = 5.5

$t_{initial}$

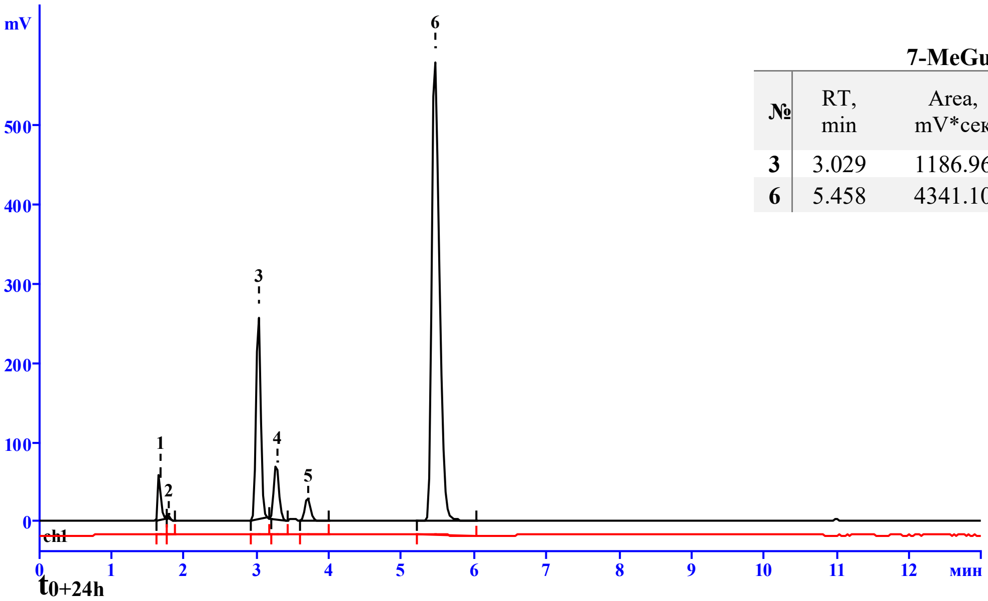

7-MeGuo+Hyp –1.5:1 – t_0

№	RT, min	Area, mV*сек	Equilibrium concentration, µM	Name
3	3.029	1186.96	200.00	Hyp
6	5.458	4341.10	300.00	7-MeGuo

t_{0+24h}

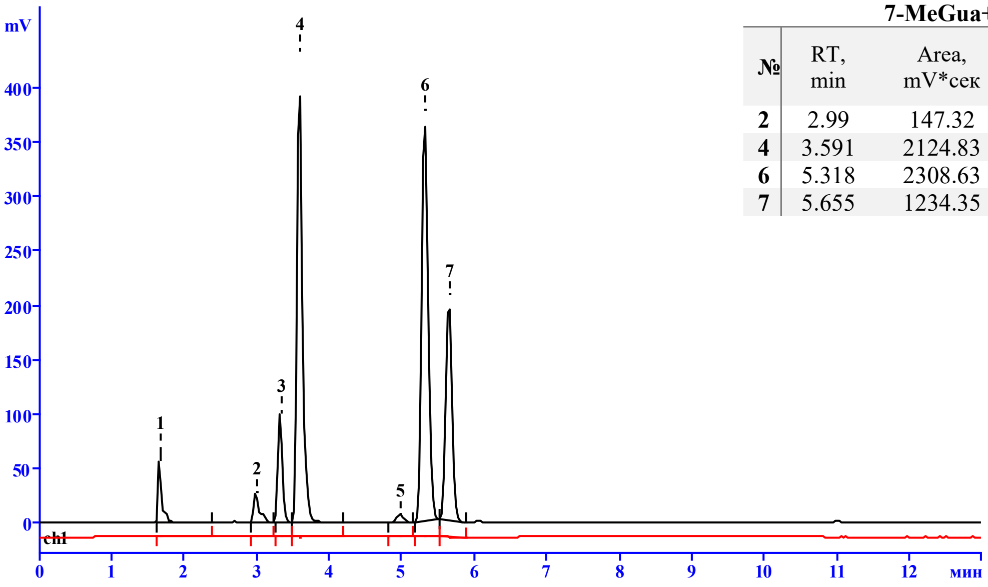

7-MeGua+Hyp – 1.5:1– t_{0+24h}

№	RT, min	Area, mV*сек	Equilibrium concentration, µM	Name
2	2.99	147.32	24.82	Hyp
4	3.591	2124.83	140.46	7-MeGua
6	5.318	2308.63	159.54	7-MeGuo
7	5.655	1234.35	175.18	Ino

Figure S18. PNP was incubated for 48h at 37°C

$t_{initial}$

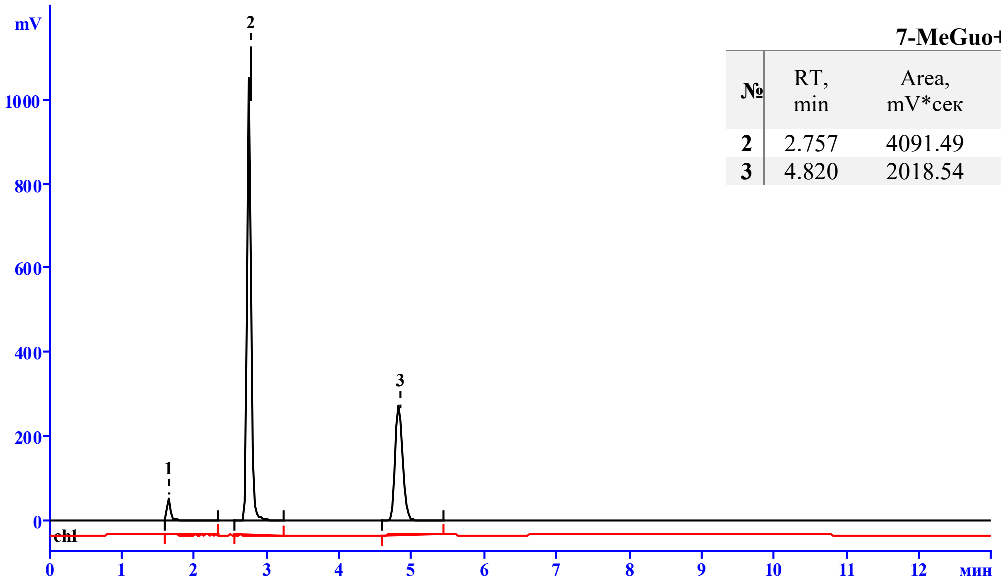

7-MeGuo+Ade – 1.5:1 – t_0

№	RT, min	Area, mV*сек	Equilibrium concentration, µM	Name
2	2.757	4091.49	200.00	Ade
3	4.820	2018.54	300.00	7-MeGuo

1) <u>pH = 5.5</u>

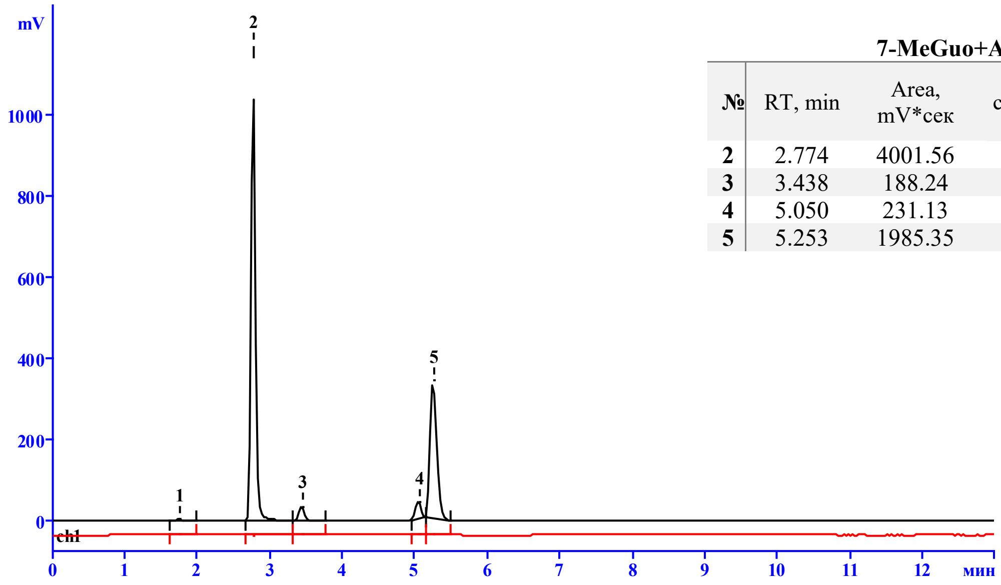

7-MeGuo+Ade – 1.5:1– t_{0+1h}

№	RT, min	Area, mV*сек	Equilibrium concentration, µM	Name
2	2.774	4001.56	195.60	Ade
3	3.438	188.24	4.93	7-MeGua
4	5.050	231.13	4.40	Ado
5	5.253	1985.35	295.07	7-MeGuo

2) <u>pH = 8.5</u>

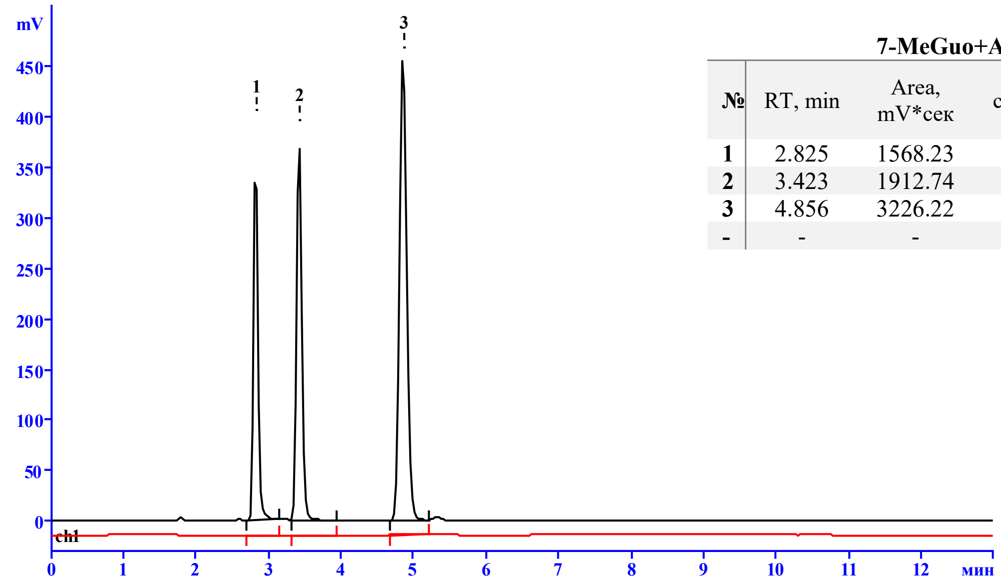

7-MeGuo+Ade – 1.5:1– t_{0+1h}

№	RT, min	Area, mV*сек	Equilibrium concentration, µM	Name
1	2.825	1568.23	76.66	Ade
2	3.423	1912.74	300.00	7-MeGua
3	4.856	3226.22	123.34	Ado
-	-	-	-	7-MeGuo

2′-deoxyadenosine from 7-methyldeoxyguanosine (7-MedGuo+Ade, analytical method)

HPLC-analysis of transglycosylation: Cosmosil 5C18-MS-II, 4.6*150 mm, 5µm, 120 Å, (Nacalai Tesque, Inc. (Japan)), 20ºC, elution in modified mobile phase: linear gradient of acetonitrile in 0.06% TFA from 2 to 8% for 10 min (flushing with 8-80 % acetonitrile-0.06% TFA for 10-10.1 min, then 80-2% for 10.1-10.8 min) at flow rate 1 ml/min with UV detection at wavelength 260 nm, injection volume 20 µL.

Figure S19. NDT II was incubated for 24h at r.t.

$t_{initial}$

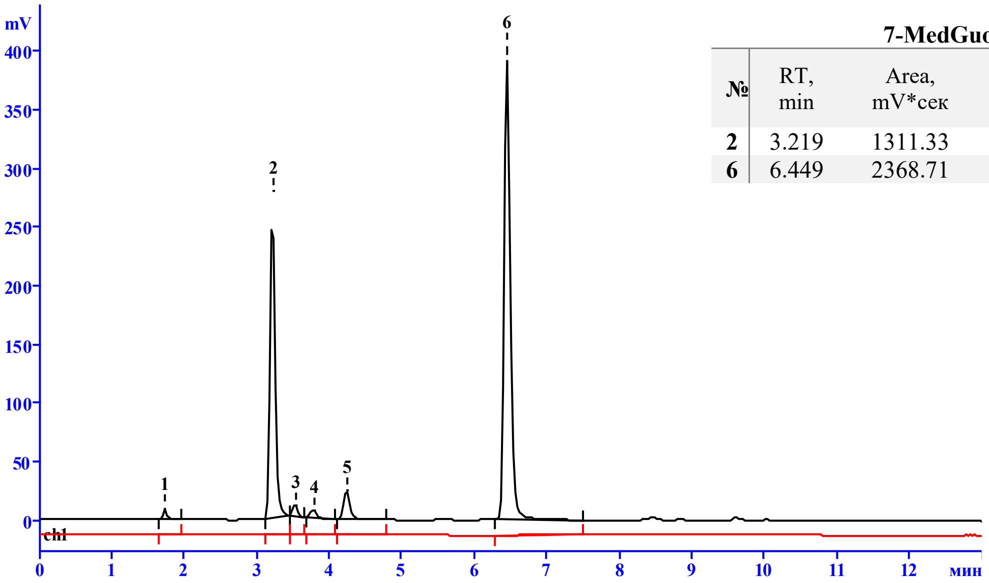

7-MedGuo+Ade – 3:1 – t_0

№	RT, min	Area, mV*сек	Equilibrium concentration, µM	Name
**2**	3.219	1311.33	100.00	Ade
**6**	6.449	2368.71	300.00	7-MedGuo

1) <u>pH = 5.5</u>

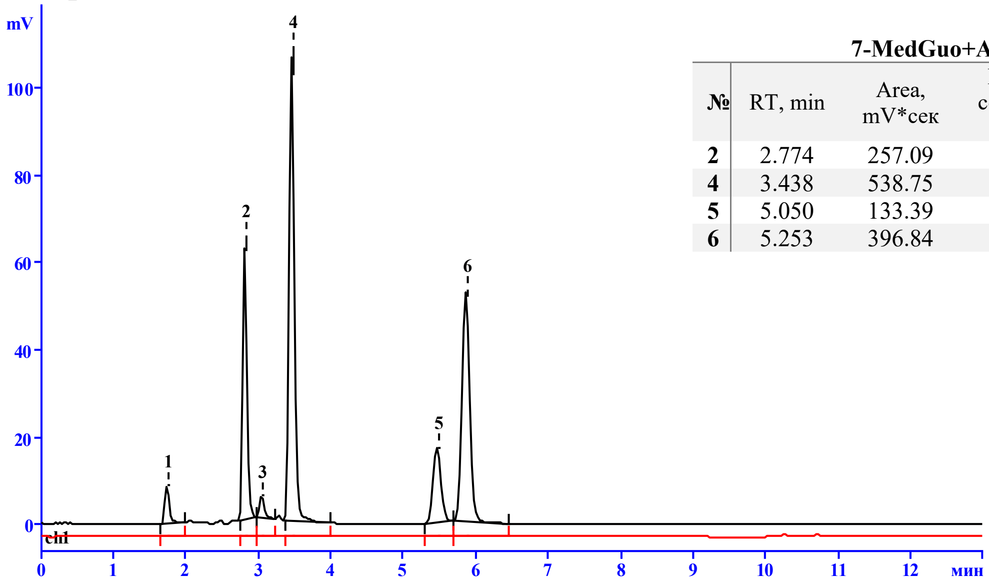

7-MedGuo+Ade – 3:1– $t_{0+20min}$

№	RT, min	Area, mV*сек	Equilibrium concentration, µM	Name
**2**	2.774	257.09	19.61	Ade
**4**	3.438	538.75	249.74	7-MeGua
**5**	5.050	133.39	80.39	7-MedGuo
**6**	5.253	396.84	50.26	dAdo

2) pH = 8.5

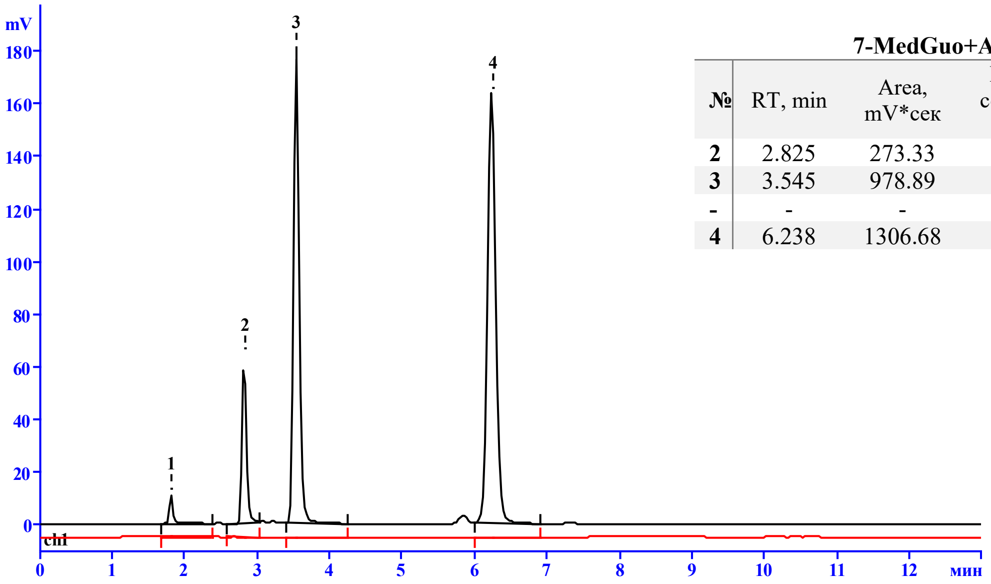

7-MedGuo+Ade – 3:1– $t_{0+20min}$

№	RT, min	Area, mV*сек	Equilibrium concentration, µM	Name
**2**	2.825	273.33	20.84	Ade
**3**	3.545	978.89	300.00	7-MeGua
**-**	-	-	-	7-MedGuo
**4**	6.238	1306.68	79.16	dAdo

Table S9. The effect of the duration of enzyme remains in the solution on transglycosylation reaction.

Enzyme	Reaction[1]	pH	Time the enzyme remains in the solution before the reaction, h	Yield, % (HPLC defined)[2]
*Ec*PNP	Ade+7-MeGuo	5,5	0	97.1
			48	<5
		8,5	0	100[3]
			48	61.7
*Ll*NDT II	Ade+7-MedGuo	5,5	0	100[3]
			24	80.4
		8,5	0	100[3]
			24	79.1

[1] Reaction conditions: *E.coli* PNP 0.2 U, (0.2 mM Ade: 0.3 mM 7-mGuo: 0.1 mM Pi (1:1.5:0.5)), 37°C, 1h; *Lac. l* NDT II. $7.8 \cdot 10^{-3}$ U, (0.1 mM Ade: 0.3 mM 7-mdGuo (1:3)), 20°C, 20min, 50 mM NaOAc (pH 5.5); 50 mM Tris-HCl, (pH 8.5).

[2] The yield was calculated as the ratio of the equilibrium concentration of the target nucleoside to the initial concentration of the limiting substrate.

[3] No detectable peak signal of the heterocyclic base.

Figure S20. Comparison of the effect of the buffer on the yield on transglycosylation reaction.

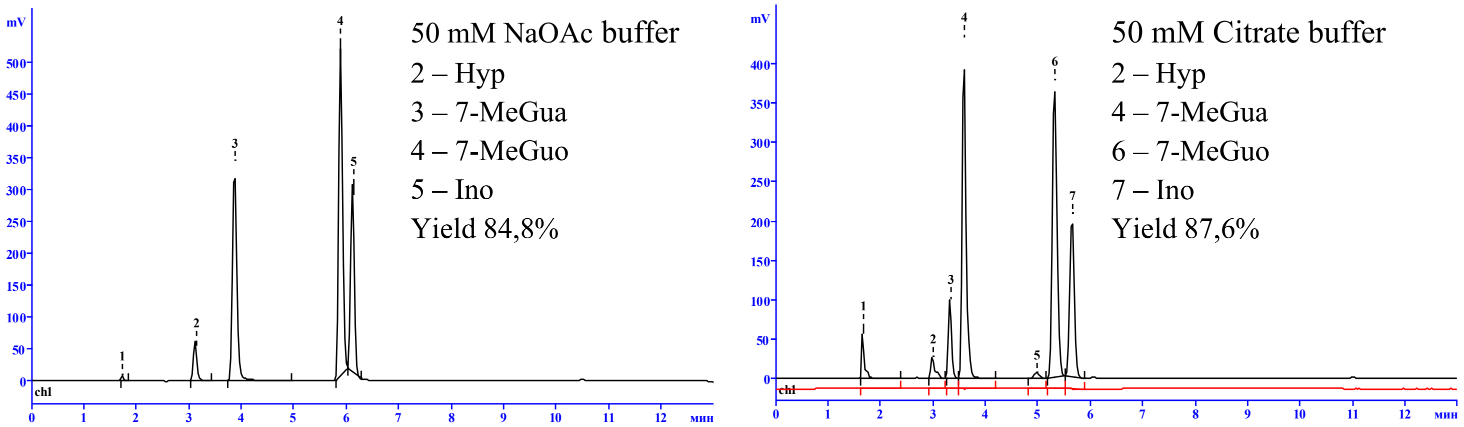

Reaction conditions: *E.coli* PNP 0.2 U, 0.2 mM Hyp: 0.3 mM 7-mGuo: 0.1 mM Pi (1:1.5:0.5), 37°C, 1d.

HPLC-analysis conditions: Cosmosil 5C18-MS-II, 4.6*150 mm, 5µm, 120 Å, (Nacalai Tesque, Inc. (Japan)), elution in modified mobile phase: linear gradient of acetonitrile in 0.06% TFA from 2 to 12% for 10 min (flushing with 12-80 % acetonitrile-0.06% TFA for 10-10.1 min, then 80-2% for 10.1-10.8 min) at flow rate 1 ml/min with UV detection at wavelength 260 nm, injection volume 20 µL.
